# The bovine oviductal environment and composition are negatively affected by elevated body energy reserves

**DOI:** 10.1371/journal.pone.0326138

**Published:** 2025-06-23

**Authors:** Natália Marins Bastos, Rodrigo Silva Goulart, Alessandra Bridi, Rosane Mazzarella, Luana Alves, Paola Maria da Silva Rosa, Ricardo de Francisco Strefezzi, Lindsay Baltel Paskoski, Ricardo Perecin Nociti, Juliano Rodrigues Sangalli, Schaienni Fontoura Saldanha, Camila Azzolin de Souza, Angélica Camargo dos Santos, Marcos Roberto Chiaratti, Guilherme Pugliesi, Flávio Vieira Meirelles, Felipe Perecin, Juliano Coelho da Silveira

**Affiliations:** 1 Department of Veterinary Medicine, College of Animal Science and Food Engineering, University of São Paulo, São Paulo, Brazil; 2 Department of Animal Science, College of Animal Science and Food Engineering, University of São Paulo, São Paulo, Brazil; 3 Department of Genetics and Evolution, Federal University of São Carlos, São Carlos, São Paulo, Brazil; 4 Department of Animal Reproduction, Faculty of Veterinary Medicine and Animal Science, University of São Paulo, São Paulo, Brazil; University of Hawai'i at Manoa, UNITED STATES OF AMERICA

## Abstract

To analyze the effects of high body energy reserve (BER) within the oviductal environment and its composition, Nellore cows were fed two different nutritional plans to obtain animals with moderate BER (MBER) and high BER (HBER). After obtaining the groups with different BERs, all animals were subjected to oestrus synchronization and artificial insemination, and 120 hours after ovulation induction, the cows were slaughtered, the reproductive tract was removed, and the ipsilateral oviduct to the corpus luteum was collected and dissected. Analyses were performed only for animals that had an 8-cell embryo in the isthmus. After embryo identification, we evaluated the molecular profiles of extracellular vesicles from oviductal flushing (OF-EVs) and luminal epithelial cells (OV-Cell) and performed histomorphological analysis of oviductal tissue from the ampullary and isthmic oviductal regions. The HBER group presented higher concentrations of ampullary extracellular vesicles (AMP-EVs) and larger sizers of isthmic extracellular vesicles (IST-EVs). The miRNA profile of AMP-EVs showed that the differentially expressed miRNAs were predicted to regulate pathways associated with cell growth, migration, differentiation and metabolism, with the HBER group being more susceptible to insulin modulation. The MBER animals showed greater ampullary vascularization than the HBER animals did. Additionally, the miRNA profile and differential gene expression (DEG) data obtained for ampullary (AMP-Cell) and isthmic (IST-Cell) luminal epithelial cells revealed pathways related to insulin metabolism. Thus, elevated BER may lead to oviductal insulin resistance, affecting normal functioning and, probably, embryo metabolism during early development, thus impacting gestational rates in these animals.

## Introduction

Until pregnancy establishment, gametes and embryos undergo several molecular, biochemical and morphological processes within the ovary and the oviduct before they reach the uterus for further development. Despite being a small anatomical structure, the oviduct has high relevance to reproductive function due to the dynamic and unique microenvironment important for reproductive events, such as final oocyte maturation, fertilization and early embryonic development [1]. The oviduct is composed of three different anatomical regions: the infundibulum, ampulla and isthmus and three cellular layers: mucosa, muscle and serosa [1,2]. The mucosa layer has ciliated and secretory cells [1,3] that, together with the oviductal fluid, allow gametic-embryonic transport and nourishment, composing the oviductal microenvironment [4–6].

Substrates and cofactors such as glucose, galactose, lactate, pyruvate, growth factors, amino acids, glycoproteins, serum albumin, enzymes and hormones [5,7,8] are components of the oviductal fluid produced by secretory epithelial cells, which is a transudate from systemic circulation in addition to follicular fluid resulting from ovulation [6,9]. Additionally, small extracellular vesicles (EVs) are present in oviductal fluid [10] and enable bidirectional cell communication between mothers (oviductal epithelial cells) and gametic-embryonic cells. EVs are biological nanoparticles that use extracellular fluids to diffuse and interact with target cells by transferring their cargo [11,12] and acting as biological vectors modulating receptor cell functions, delivering transcripts, microRNAs (miRNAs) and proteins that may affect target cells [13,14]. This is because EVs contain bioactive materials, such as proteins, lipids, mRNAs, and miRNAs, which partially represent the secretory cell content [15,16]. Thus, EVs can participate in gametic-embryonic development and play important roles in these processes [16–18]. Since EVs harboring miRNAs have powerful systemic access to various distant cells, they can have autocrine, paracrine and endocrine signaling functions [11] and may function as fine-tuners in reproductive events such as early embryo development.

All oviduct structures are under the influence of ovarian cyclicity. Hormonal changes across the estrous cycle result in alterations in histomorphology and epithelial cellular composition in the ampulla and isthmus [3,19] and modifications in EVs cargo from oviductal flushing [20,21]. In addition, the presence of an embryo modulates not only the molecular profile of oviductal epithelial cells but also the oviductal flushing and EVs content [8,22,23]. This indicates that external (ovarian cyclicity) and internal (embryo presence) processes modulate the response of the oviductal microenvironment [5]. Associated with external processes, nutritional management influences body energy reserves (BERs), which affect animal metabolism, physiology and the endocrine system [24–26]. Therefore, BER can modulate ovarian function, embryonic quality and further pregnancy establishment [27,28]. Compared to those with moderate BER, cows with elevated BERs have a greater reproductive failure rate and greater risk of metabolic diseases [29,30], suggesting that reproductive deficiency may be related to other anatomical structures in addition to the ovary. In a recent study performed by our group, HBER cows presented a lower ovulation rate and an even lower embryo recovery rate, possibly due to hyperinsulinemia [31]. Usually, high-genetic merit cows used as donors of oocytes and embryos are usually animals with high BERs and reproductive problems. The bovine can be used as a biological model for human studies due to its similar embryo development characteristics [32,33]. In addition, in modern human society, obesity and overweight are increasingly common among women of reproductive age. Studies aiming to understand the effects of high BER on the oviductal microenvironment and composition should be carried out to increase our understanding of the possible reproductive consequences in subsequent pregnancy. In this way, the hypothesis for this work is that increased BER alters the oviductal environment and composition, compared to moderated BER cows, providing a negative environment for embryo development.

## Materials and methods

The experiment was performed at the Laboratory of Morphophysiology and Molecular Development of the Department of Veterinary Medicine, both of which are located at the University of São Paulo (Campus of Pirassununga, SP, Brazil). All the experimental procedures were approved by the University of São Paulo Research Ethics Committee (protocol number: 1522231019). This study is reported in accordance with ARRIVE guidelines. All methods were performed in accordance with relevant guidelines and regulations.

### Animal model and sample collection

Nellore cows (n = 21) were randomly divided into two experimental groups and subjected to two different nutritional plans: moderate (n = 9) or high (n = 12) body energy reserve (BER), as previously described by Bastos et al. [31]. Briefly, 21 Nellore multiparous, non-lactating and not pregnant cows (510,67 ± 15,55 kg of body weight, 6,06 ± 0,54 years old and 1,44 ± 0,0083 m of withers height) with a mean body condition score (BCS) of 5,5 ± 0,21 (1–9 scale, according to NASEM [34]), were used in the experiment. The different BERs were achieved in 70 days, including an adaptation period to the finishing diet (21 days) using different feeding programs as previously described [31]. In order to monitor the maintenance and progression of energy reserves in MBER and HBER cows, the animals were weighted weekly as well as evaluated for fat thickness and serum metabolic hormones as previously described [31]. Before the end of the feedlot period animals were subjected to oestrus synchronization, artificial insemination approximately 120 hours after ovulation induction using semen from a single bull, as previously described [31]. Briefly, on the first day, Nellore cows received 2 mL of estradiol benzoate (Sincrodiol®, Ourofino Agronegócio) intramuscularly, 2 mL of PGF2α (Sincrocio®, Ourofino Agronegócio) intramuscularly and insertion of an intravaginal progesterone device (1g; Sincrogest®), Ourofino Agronegócio) which was withdrawn on the 8th day (D8). Still on D8 sync, animals received 2 mL of intramuscular PGF2α at the time of removal of the intravaginal progesterone device. In D10 sinc 2.5 mL of GnRH (Sincroforte®, Ourofino Agronegócio) was administered intramuscularly, the diameter of the dominant follicle (DF) was analyzed by ultrasound (MyLab Delta, Esaote, Italy), and after 12 hours of GnRH administration it was performed the artificial insemination. All animals were inseminated with semen from a single bull with previously known fertility. Confirmation of ovulation was performed 12 hours after fixed-time artificial insemination (FTAI) by ultrasound. Approximately 120 h after ovulation induction, cows were slaughtered and, upon slaughter the reproductive tract was removed and immediately transported to the laboratory [31]. Briefly, the ipsilateral oviducts to the corpus luteum were collected and dissected, and the oviductal portions of the ampulla and isthmus were separated through the ampullary-isthmic junction before oviductal flushing [23,31]. A total of 6 of the 21 cows (MBER: n = 9; HBER: n = 12) presented an 8-cell embryo within the flushing fluid; therefore, sample collection and analysis were performed at the MBER: n = 3 and the HBER: n = 3. Oviductal tissue, oviductal luminal epithelial cells and oviductal flushing fluid were collected for histopathological and molecular analysis, and extracellular vesicle isolation was performed for the ampullary and isthmic oviductal regions, respectively.

### Isolation of small extracellular vesicles from oviductal flushing

After the oviducts were obtained, the ampullary and isthmic portions were individually flushed with 1 mL of phosphate-saline solution calcium and magnesium free (1xPBS) and subsequently used to obtain oviductal small extracellular vesicles (OF-EVs) as previously described by Mazzarella et al. [23]. Briefly, the samples were centrifuged at 4°C and 300 × g for 10 minutes to remove cells, at 2000 × g for 10 minutes to remove cell debris, and at 16500 × g for 30 minutes to remove larger extracellular vesicles. To obtain a pellet enriched in small extracellular vesicles (smaller than 200 nm), 200 µl of the resulting supernatant was diluted in 1 × PBS to 1 mL, filtered through a 0.20 µm pore filter (Corning) and ultracentrifuged at 119700xg for 70 minutes at 4°C (Optima XE-90 Ultracentrifuge; 70 Ti rotor; Beckman Coulter). Then, the obtained pellet was washed in 1 × PBS and ultracentrifuged again at 119700xg for 70 minutes at 4°C. The pellet enriched with small extracellular vesicles was eluted in 20 µl of 1 × PBS for further characterization and miRNA content analysis.

### Characterization of small extracellular vesicles from oviductal flushing

OF-EVs characterization consisted of nanoparticle tracking analysis (NTA), transmission electron microscopy (TEM) and flow cytometry. To perform the TEM, slaughterhouse samples were used to avoid wasting OF-EVs from the experimental samples, which were subsequently subjected to NTA, flow cytometry and miRNA content analysis.

#### Nanoparticle tracking analysis.

OF-EVs ampullary (AMP-EVs) and isthmic (IST-EVs) small extracellular vesicles were isolated from 200 µl of oviductal isthmic flushing fluid. Upon AMP-EVs and IST-EVs isolation, 5 µl of the eluted pellet from both oviductal portions was diluted in 495 µl of 1x PBS each and used for particle size and concentration evaluation via a NanoSight device (NS300; NTA 3.4 Build 3.1.45; Malvern). Five 30-second videos were taken at a controlled temperature of 38.5°C and a camera level of 13 considering a threshold of 5. The size and concentration of each video were considered for statistical analysis.

#### Transmission electron microscopy.

After isolation by serial centrifugation, the EVs pellets were diluted in fixative solution (0.1 M cacodylate, 2.5% glutaraldehyde, and 4% paraformaldehyde; pH 7.2–7.4) for two hours before being ultracentrifuged again and resuspended in 20 μl of ultrapure Milli-Q water. The analyses were performed at the Multiuser Laboratory of Electronic Microscopy of the Department of Cellular and Molecular Biology, Faculty of Medicine of Ribeirão Preto, using a transmission electron microscope (FEI 200kV, model Tecnai 20, emitter LAB6).

#### Flow cytometry analysis.

After the EVs isolation protocol, samples were stained with the following antibodies as positive markers: PE-conjugated mouse monoclonal CD81 (ab81436; 1:20), mouse monoclonal Syntenin (sc-515538; 1:50), and Alexa fluor 488 goat anti-mouse polyclonal as secondary antibody (A11001; 1:2000). Calcein-AM (Sigma-Aldrich; 17783; 1uM) was used as a marker for cytoplasm containing nanoparticles. Calcein-AM positive events were used as inclusion factors for the analysis of CD81. As a marker to detect cell contamination in the isolated EVs, Calnexin (sc-23954; 1:50) was used. For sample preparation, pools of AMP-EVs and IST-EVs, from MBER and HBER group, were incubated with the antibodies for 2 h at room temperature in a shaker. For Syntenin and Calnexin, before the incubation the EVs samples were incubated with 0.001% Triton X- (X100, Sigma-Aldrich) solution for 15 min at room temperature. For Syntenin, the primary antibody was added and incubated with the samples for 30 minutes. Following, Alexa fluor 488-conjugated secondary antibody was added to the samples (100 µL) and incubated for further 1:30 h. After the incubation, samples were diluted in 200 µL of 3 × filtered PBS and analyzed by Cytoflex (Beckman Coulter). The flow cytometry instrument was optimized for nanoparticle detection by the violet SSC channel (V-SSC- 405/10) and for PE and FITC fluorescence, depending on the fluorophore conjugated to each antibody. The gain for V-SSC was 100, FITC 450 and PE 600. The threshold was set primarily for V-SSC at 500 and secondarily for FITC at 600. The number of events per seconds was maintained around 2000 and abortion rate was 8%. Approximated size of the nanoparticles was determinate using a mixture of fluorescent Megamix-Plus SSC and MegamixPlus FSC beads (BioCytex) which have different sizes (100, 160, 200, 240, 300, 500, 900 nm). Using the control (negative samples), gating was organized so unlabeled particles and negative samples were not detected. The acquisition was programmed to occur slowly (10uL/min) for 5 min/sample. Thus, the number of events within the set gates was used to determine the presence or absence of markers.

### Histomorphology analysis

After oviductal flushing, tissue samples from the ampulla and isthmus were collected and fixed in 10% buffered formalin for 48 hours. Then, the samples were routinely processed for histology. Four-micron cross-sections were obtained from each sample. Digital images of the ampullary and isthmic sections were obtained using a microscope coupled to a digital camera (Leica DM500 and Leica ICC50HD, Leica Microsystems; Heerbrugg, Switzerland). Masson’s trichrome stain was used to identify collagen fibers as blue. The mucosa total area and the collagen area were measured to calculate the collagen percentage (collagen area/total area). These values are presented as percentages (%). To determine vascularization, blood vessels were detected using immunohistochemistry (rabbit polyclonal anti-von Willebrand factor antibody (vWF), Abcam, code ab6994) at 40x magnification for both oviductal regions. The mucosal area without the lining epithelium was obtained, and the vessels positive for vWF were counted for vascularization determination (vessels/mm^2^). The luminal epithelial perimeter (mm) was obtained by drawing the luminal epithelial lining with the “trace-wand” tool in the Image-Pro Plus software (version 4.5 for Windows, Media Cybernetics; Silver Spring, MD, USA), as previously described by Gonella-Diaza [19], on the slides used to analyze the degree of collagen.

### Luminal epithelial cell collection for molecular analysis

Oviductal luminal epithelial cells (OV-Cell) from the ampulla (AMP-Cell) and isthmus (IST-Cell) were obtained by squeezing the tissue with a sterile glass slide [23]. The cells were immediately frozen in liquid nitrogen for further molecular analyses.

### Total RNA extraction

Total RNA was extracted from OF-EVs and OV-Cell using a miRNeasy Mini Kit (QIAGEN, Hilden, Germany) according to the manufacturer’s instructions. The RNA quantity and quality were analyzed via spectrometry (NanoDrop 2000, Thermo Fisher Scientific; absorbance ratio 260/280 nm), and the RNA was treated with DNaseI (Invitrogen; Carlsbad, CA) according to the manufacturer’s instructions. After extraction, the RNA was stored at −80°C until use.

### miRNA analysis

Total RNA was transformed into cDNA using the miScript II RT Kit (QIAGEN) and miScript HiSpec Buffer to obtain mature miRNAs in OV-Cells and into miScript HiFlex Buffer to obtain mature and precursor miRNAs in OF-EVs, similar to previous methods [18]. OV-cell reactions contained 100 ng of total RNA, while OF-EVs reactions were performed with 200 ng of total RNA. Both procedures were performed in a thermocycler (Life Technology) at 37°C for 60 minutes, followed by 95°C for 5 minutes. RT‒PCR was used to quantify the transcripts according to Da Silveira et al. [35], in which at least 0.2 ng of cDNA and 1 µl of forward primer were obtained from the mature bovine miRNA sequences available in the mirBase software (http://www.mirbase.org). The temperature was 95°C for 15 minutes, followed by 45 cycles of 94°C for 15 seconds, 55°C for 30 seconds and 70°C for 30 seconds. For each sample, an expression analysis of 383 bovine miRNAs was performed [35]. The miRNAs were considered to be present when they presented a cycle threshold (CT) lower than 37 in all biological repetitions and an appropriate melting curve. The CT data generated by amplification were normalized to the geometric means of bta-miR-99b, Hm/Ms/Rt T1 snRNA and RNT43 snoRNA for OV-Cells and bta-miR-99 for OF-EVs [17]. The miRNAs differentially expressed between groups were evaluated by miRWalk software version 3.0, and the predicted regulatory pathways were identified. Pathways were considered significant when the Benjamini–Hochberg (BH) adjustment was *P *< 0.05.

### RNA library preparation and sequencing

Sequencing libraries of OV-Cells were prepared using the TruSeq Stranded mRNA Kit (Illumina). In brief, poly-A RNAs were captured using poly-T oligo-attached magnetic beads. Poly-A RNAs were then fragmented, subjected to double-strand cDNA synthesis, ligated with dual-index adapters, PCR enriched and purified to create the final cDNA library. A High Sensitivity DNA Kit (Agilent) was used to confirm the library length (~300 bp) and lack of dimers. Finally, the libraries were quantified via quantitative PCR using the KAPA Library Quantification Kit (KAPA) and pooled, and the final library concentration was adjusted to 1 nM based on the Qubit dsDNA HS Assay Kit (Thermo Fisher Scientific). Sequencing was performed on a NextSeq 550 instrument (Illumina) using 1.8 pM of pooled libraries and a NextSeq 500/550 High Output Kit v2.5 (75 cycles).

The quality of the reads was assessed using FastQC (http://www.bioinformatics.babraham.ac.uk/projects/fastqc/). The 76 bp reads were mapped using star [36], and identification and quantification were performed using ARS-UCD1.2 (Ensembl and NCBI) as a reference genome and featureCounts implemented in the Rsuberead package [37,38] for gene count. Once the genes were identified, differential expression analysis was performed between groups using DESEq2 [39] considering a padj<0,10 and an absolute log2Folchange >0.5. Additionally, we considered genes to be differentially expressed if they were exclusive, expressed in one group (expressed in all samples from the same group) or not expressed in the other group (zero counts in all samples from the same group) within comparison and using the function filterByExpr from the edgeR package [40]. We estimated the hub genes using CeTF [41] based on the regulatory impact factor (RIF) and partial correlation and information theory (PCIT) [42,43]. Gene Ontology (GO) analysis was performed using clusterProfiler [44], and pathways were explored using Pathview [45]. The data were visualized using R software, in which we primarily observed the classification, intensity, and difference in expression between groups.

### Statistical analysis

The OF-EVs size and concentration, miRNA expression of OF-EVs and OV-Cells and histomorphology analysis data were analyzed using the fixed effect of the body energy reserve, whose means were adjusted by the least squares method and compared using the probability of difference determined via Student’s *t* test. All the analyses utilized the program “JMP” (version 7.01; Statistical Analysis Software Institute, SAS®, Inc., Cary, NC). A significant difference was defined as *P* < 0.05.

## Results

### The size and concentration of small extracellular vesicles in the oviductal region are affected by body energy reserves

**Fig 1:**
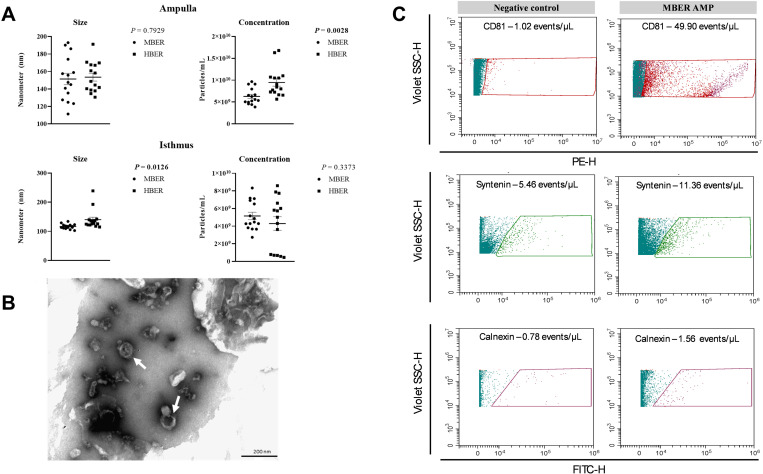
Oviductal flushing extracellular vesicles characterization from cows with different body energy reserve. **A.** Extracellular vesicles size and concentration in ampulla and isthmus analyzed by NTA. Mean ± standard error. P-value is on the right top of the figure. **B.** Transmission electron microscopy images show (white arrows) the extracellular vesicles presence in oviductal flushing. **C.** Flow cytometry representative results show positive events inside the gates created based on the unlabeled particles and negative control for each marker.

After oviductal flushing was obtained, to confirm the effectiveness of the EVs isolation protocol, AMP-EVs and IST-EVs were isolated and characterized. The size and concentration of the particles were determined via NTA analyses. For the ampulla, there was no difference in size (MBER: 151.41 ± 6.60 nm; HBER: 153.53 ± 4.47 nm, *P* = 0.7929; Fig 1A); however, the AMP-EVs concentration was greater in the HBER animals (MBER: 6.30 × 10^9 ^± 4.80 × 10^8^ particles/mL; HBER: 9.50 × 10^9^ ± 8.54 × 10^8^ particles/mL, *P* = 0.0028; Fig 1A). For the isthmus, the HBER IST-EVs were larger (MBER: 116.67 ± 2.16 nm; HBER: 140.05 ± 8.50 nm, *P* = 0.0126; Fig 1A), and there was no difference in particle concentration (MBER: 5.15 × 10^9 ^± 7.68 × 10^8^ particles/mL; HBER: 4.29 × 10^9^ ± 8.50 × 10^8^ particles/mL; *P* = 0.3373; Fig 1A). Transmission electron microscopy images showed the presence of OF-EVs (Fig 1B). Flow cytometry analyses verified the presence of specific proteins in OF-EVs. The CD81 and Syntenin proteins were found in the OF-EVs lysate with no significative events for negative control (S1A Fig), while the endoplasmic reticulum marker protein (Calnexin) was found only in oviductal cells (S1B Fig; S1 Table), confirming the use of the isolation protocol (Fig 1C).

### miRNA analyses of small extracellular vesicles from oviductal flushing fluid identified differences only in the ampullary region

**Fig 2:**
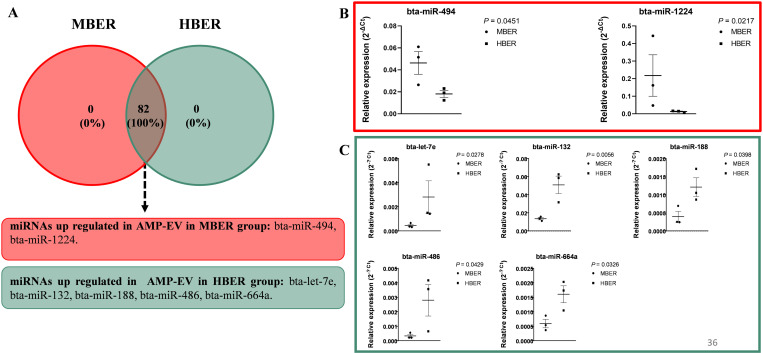
miRNAs expression in extracellular vesicles from ampullary flushing (AMP-EVs) from cows with different body energy reserve. **A.** Venn diagram demonstrating the 82 common miRNAs between groups which 2 were up regulated in MBER group and 5 up regulated in HBER group. **B.** Relative expression of up regulated miRNAs in AMP-EVs in MBER group. **C.** Relative expression of up regulated miRNAs in AMP-EVs in HBER group. Mean ± standard error. P-value is on the right top of the figure.

To understand the influence of BER on the oviductal environment, the OF-EVs miRNA content was analyzed with a 383 custom miRNA profiler plate (S2 and S3 Tables for AMP-EVs and IST-EVs, respectively). The AMP-EVs miRNA content presented a mean repeatability of 0.76 and 0.75 for the MBER and HBER, respectively. There were 82 common miRNAs between the groups (Fig 2A; S4 Table), two of which were upregulated in the MBER animals (bta-miR-494 and bta-miR-1224; Figs 2A and 2B), and five were upregulated in the HBER animals (bta-let-7e, bta-miR-132, bta-miR-188, bta-miR-486, and bta-miR-664a; Figs 2A and 2C). For the IST-EVs, the mean repeatability of the samples was 0.80 and 0.85 for the MBER and HBER, respectively. There were 150 common miRNAs between the groups; however, we were not able to detect significant differences in the expression of these miRNAs (S5 Table).

### Enrichment analysis of differentially expressed miRNAs from AMP-EVs associated with metabolism and cell-to-cell interactions

**Fig 3:**
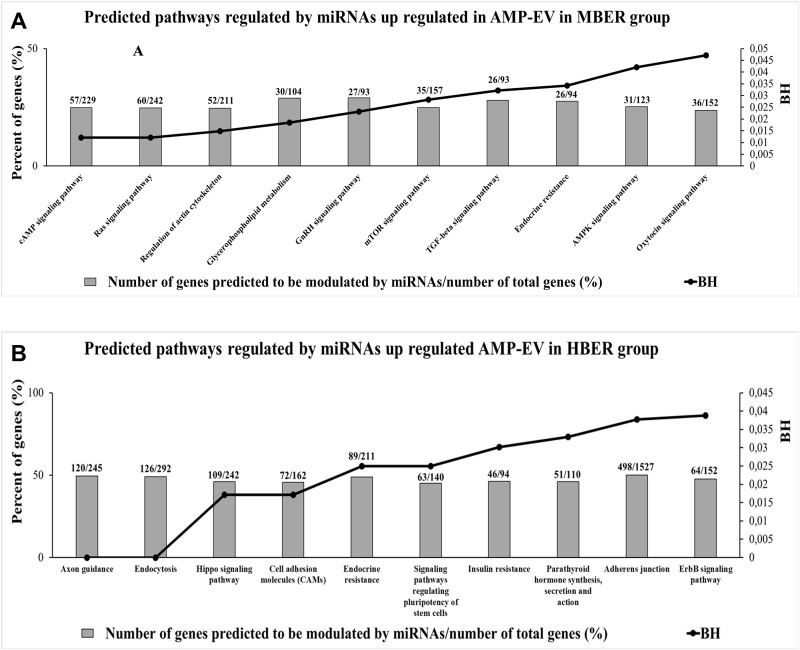
Enrichment analysis performed in miRWalk 3.0 software of predicted pathways modulated by miRNAs differentially expressed in small extracellular vesicles from ampullary oviductal flushing (AMP-EVs). **A.** The 10 predicted pathways with highest percent of genes predicted to be modulated by miRNAs up regulated in AMP-EVs in MBER group. **B.** The 10 predicted pathways with highest percent of genes predicted to be modulated by miRNAs up regulated in AMP-EVs in HBER group. The Y-axis in left represents the percent of genes (%) predicted to be modulated by miRNAs and the Y-axis in right shows the BH (BH < 0.05).

To determine the predicted biological functions regulated by miRNAs differentially expressed in AMP-EVs, we performed bioinformatics analysis. Among the 283 predicted pathways (S6 Table) regulated by the two miRNAs upregulated in the MBER group, 51 were significant, 10 of which were involved, with the highest percentage of genes predicted to be modulated by those miRNAs, as shown in Fig 3A. Among the five miRNAs upregulated in the HBER group, there were 313 total predicted pathways (S7 Table), 42 of which were significant, and the 10 pathways related to the genes with the highest percentage of genes predicted to be modulated by those miRNAs are represented in Fig 3B. Our results demonstrated that the miRNA-containing AMP-EVs cargo in the MBER group was predicted to regulate among others pathways such as glycerophospholipid metabolism, regulation of the actin cytoskeleton, GnRH signaling and TGF-beta signaling pathways, while the miRNAs present in the AMP-EVs cargo in the HBER group were involved in endocytosis, insulin resistance, the Hippo and ErbB signaling pathways and cell-to-cell interaction pathways.

### Histomorphology revealed greater vascularization in the MBER animals

**Fig 4:**
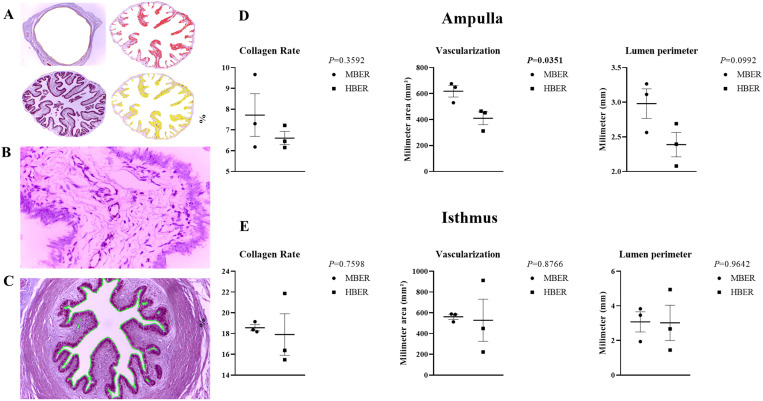
Oviductal histomorphological analysis to obtain **A.** Collagen rate, by red and yellow colors segmentation of the image. **B.** Vascularization (blood vessels/mm^2^; white arrows). **C.** Luminal perimeter (micrometer; µm) from cows with different body energy reserve. **D.** Histomorphological analysis in ampullary region. **E.** Histomorphological analysis in isthmic region. Mean ± standard error. P-value is on the right top of the figure.

Histomorphology analyses were performed to determine the collagen concentration (Fig 4A), degree of vascularization (Fig 4B) and luminal perimeter (Fig 4C) of the ampullary (Fig 4D) and isthmic (Fig 4E) oviductal regions. In the ampulla, there were no differences between the groups in terms of the collagen percentage or luminal perimeter; however, the MBER animals had greater ampullary vascularization than the HBER animals did (*P *= 0.0351; Fig 4D). The collagen percentage, vascularization and luminal perimeter did not differ among the groups at the isthmus.

### miRNA analyses of oviductal epithelial cells demonstrated the effects of increased body energy reserve

**Fig 5:**
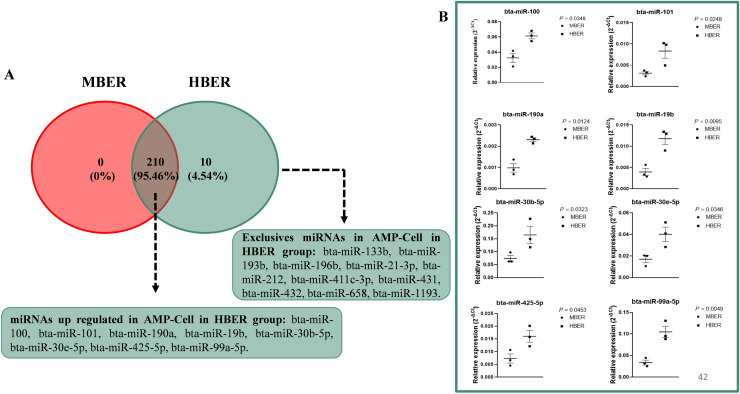
miRNAs expression in ampullary luminal epithelial cells (AMP-Cell) from cows with different body energy reserve. **A.** Venn diagram demonstrating the 210 common miRNAs between groups and 10 exclusives miRNAs in HBER group. Of the 210 common miRNAs, 8 were up regulated in HBER group **B.** Relative expression of up regulated miRNAs in AMP-Cell in HBER group. Mean ± standard error. P-value is on the right top of the figure.

To determine the influence of BER on oviductal epithelial cells and OV-cell miRNA levels, we analyzed 383 miRNAs in each sample (S8 and S9 Tables for AMP-EVs and IST-EVs, respectively). The AMP-cell miRNA contents presented mean repeatability values of 0.90 and 0.90 for the MBER and HBER, respectively. There were a total of 210 common miRNAs between the groups (Fig 5A; S10 Table), and 10 exclusive miRNAs were found only in HBER animals (bta-miR-133b, bta-miR-193b, bta-miR-196b, bta-miR-21-3p, bta-miR-212, bta-miR-411c-3p, bta-miR-431, bta-miR-432, bta-miR-658, and bta-miR-1193; Fig 5A). Among the common miRNAs between the groups, a total of eight miRNAs were upregulated in HBER animals (bta-miR-100, bta-miR-101, bta-miR-190a, bta-miR-19b, bta-miR-30b-5p, bta-miR-30e-5p, bta-miR-425-5p, and bta-miR-99a-5p; Figs 5A and 5B) compared to those in MBER animals.

**Fig 6:**
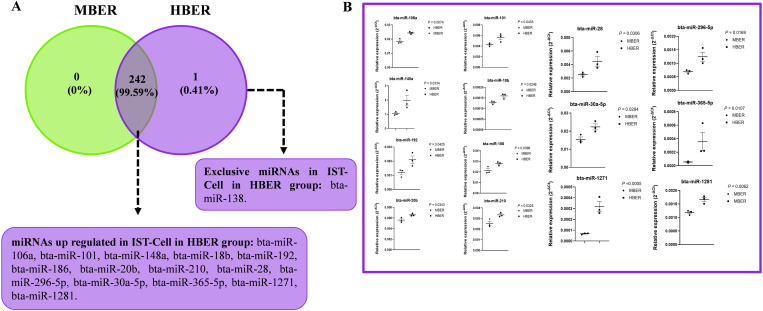
miRNAs expression in isthmic luminal epithelial cells (IST-Cell) from cows with different body energy reserve. **A.** Venn diagram demonstrating the 242 common miRNAs between groups and 1 exclusive miRNAs in HBER group. Of the 243 common miRNAs, 14 were up regulated in HBER group **B.** Relative expression of up regulated miRNAs in IST-Cell in HBER group. Mean ± standard error. P-value is on the right top of the figure.

For the IST-Cell, the mean repeatability of the samples was 0.80 and 0.85 for the MBER and HBER, respectively. There were 242 common miRNAs between the groups (Fig 6A; S11 Table), and one exclusive miRNA (bta-miR-138) was present only in the HBER animals. Among the common miRNAs between groups, a total of 13 were upregulated in HBER animals (bta-miR-106a, bta-miR-101, bta-miR-148a, bta-miR-18b, bta-miR-192, bta-miR-186, bta-miR-20b, bta-miR-210, bta-miR-28, bta-miR-296-5p, bta-miR-30a-5p, bta-miR-365-5p, and bta-miR-1271; Figs 6A and 6B) compared to those in MBER animals.

### Enrichment analysis of miRNAs differentially expressed in oviductal cells predicted that they regulate pathways involved in the insulin response

**Fig 7:**
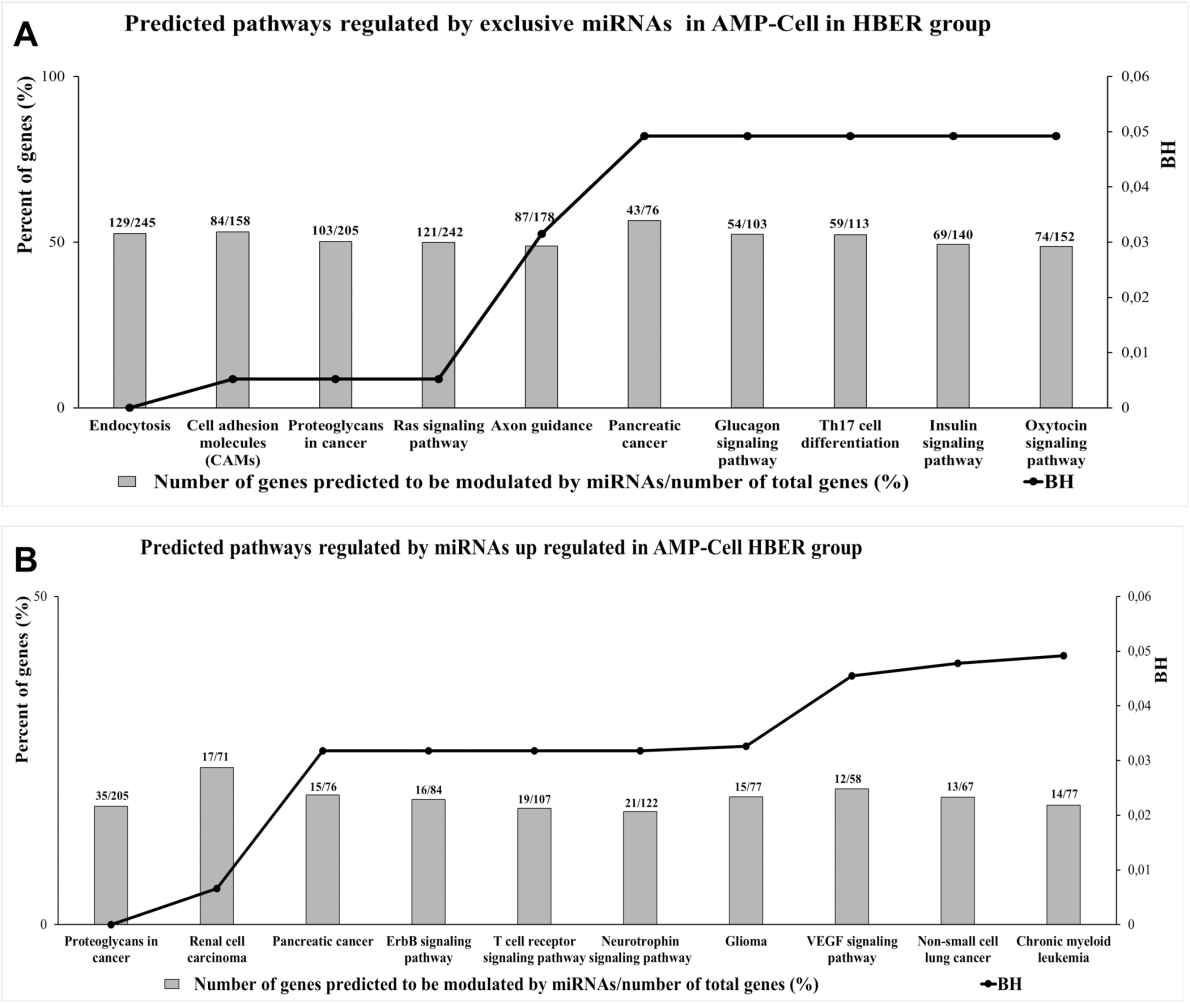
Enrichment analysis performed in miRWalk 3.0 software of predicted pathways modulated by miRNAs exclusives and differentially expressed in ampullary luminal epithelial cells (AMP-Cell) from HBER group. **A**. The 10 predicted pathways with highest percent of genes predicted to be modulated by exclusives miRNAs in AMP-Cell in HBER group. **B.** The 10 predicted pathways with highest percent of genes predicted to be modulated by miRNAs up regulated AMP-Cells in HBER group. The Y-axis in left represents the percent of genes (%) predicted to be modulated by miRNAs and the Y-axis in right shows the BH (BH < 0.05).

To determine the predicted biological functions regulated by miRNAs differentially expressed in OV-Cell, we performed bioinformatics analysis of the unique and differentially expressed miRNAs. In AMP-Cell, a total of 315 predictive pathways were regulated by the 10 exclusive miRNAs in the HBER group (S12 Table), 16 of which were significantly different according to BH values, and 10 were selected as pathways with the highest percentage of genes predicted to be modulated by those miRNAs (Fig 7A). Among the 199 pathways predicted to be regulated by the 8 miRNAs upregulated in the HBER cohort (S13 Table), 19 were significantly different according to BH values, and 10 of those genes were predicted to be modulated the most (Fig 7B). Our results demonstrated that the exclusive miRNAs in HBER AMP-Cell are predicted to regulate pathways such as the glucagon, insulin and oxytocin signaling pathways, while the upregulated miRNAs in HBER AMP-Cell are predicted to be involved in proteoglycans in cancer and ErbB and VEGF signaling pathways.

**Fig 8:**
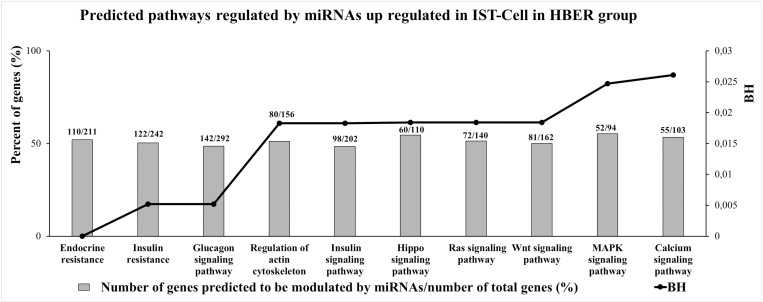
Enrichment analysis performed in miRWalk 3.0 software of predicted pathways modulated by miRNAs differentially expressed in isthmic luminal epithelial cells (IST-Cell) from HBER group. The 10 predicted pathways with highest percent of genes predicted to be modulated by miRNAs up regulated IST-Cells in HBER group. The Y-axis in left represents the percent of genes (%) predicted to be modulated by miRNAs and the Y-axis in right shows the BH (BH < 0.05).

The 14 miRNAs upregulated in the HBER IST-Cell cohort were predicted to regulate 314 pathways (S14 Table), 48 of which were significantly different according to their BH values, and 10 of which had the highest percentage of genes predicted to be modulated by those miRNAs (Fig 8). The upregulated miRNAs in the HBER IST-Cell line were related to endocrine resistance, insulin resistance and the insulin signaling pathway.

### Differentially expressed genes in oviductal epithelial cells modulate metabolism and hormone response

With the aim of understanding the influence of BER on the RNA profile and biological processes, RNA-seq was performed in OV-Cell. We evaluated differentially expressed genes (DEGs) between different comparisons, and our results revealed a differential RNA profile in OV-Cell from cows with different BERs. Briefly, in AMP-Cell, there were 11 DEGs, 6 of which were upregulated in the MBER group (ENSBTAG00000049543, PDYN, EPPK1, NQO2, SCG5 and IQCE; S2A Fig, red), and 5 of which were upregulated in the HBER group (KRT80, TPMT, SFMBT2, MFAP2 and CA10; S2A Fig, green dots). Heatmap analysis demonstrated the differences within samples within each group (S2B Fig). The DEGs were involved in biological pathways such as neuroactive ligand‒receptor, cytokine‒cytokine receptor interaction, glycine, serine and threonine metabolism, cysteine and methionine metabolism, the Hedgehog signaling pathway and the Wnt signaling pathway (S2C Fig).

In the IST-Cell cohort, there were a total of 17 DEGs, 8 of which were upregulated in the MBER group (LOC112444164, BCL2L14, CLDC, D2HGDH, TBC1D8, VCAN, NTS, and ALAD; S3A Fig, green dots), and 9 of which were upregulated in the HBER group (LOC78706, ENSBTAG00000049543, TMEM246, VNN2, LOC112447728, EPPK1, ADH6, AIM2, and PDYN; S3A Fig, purple dots). Heatmap analysis demonstrated the differences within samples within each group (S3B Fig). These DEGs were involved in biological pathways such as neuroactive ligand‒receptor interaction; the calcium signaling pathway; the cAMP signaling pathway; vitamin digestion and absorption; alanine, aspartate and glutamate metabolism; carbohydrate digestion and absorption; the estrogen signaling pathway; and vascular smooth muscle contraction (S3C Fig).

## Discussion

Genetic donor cattle, which can have high BERs, frequently suffer from reproductive problems. In another context, due to modern human lifestyles, the number of women of childbearing age who are overweight or obese has significantly increased over the years, representing an important problem to be solved [46]. These metabolic conditions affect women’s health and reproductive function, which may negatively affect fetal outcomes [47,48]. However, the biological and molecular causes of metabolism and, consequently, the BER responsible for reproductive disorders and the consequences for embryo development in animals and humans have not yet been fully elucidated. Once the oviduct is a dynamic structure that, under ovarian hormone control, provides the ideal microenvironment for fertilization and embryo development, this work aimed to evaluate the composition of the oviduct environment as well as epithelial cells from cows with different BERs. To our knowledge, this is the first study connecting BER with oviductal effects as well as evaluating the different oviduct regions (ampulla and isthmus). To do that, we subjected Nellore cows from the same herd to a feedlot period to obtain cows with moderate BER and elevated BER. Once the animals had different BERs, the cows were subjected to oestrus synchronization and slaughter, and the ipsilateral oviducts to the corpus luteum were collected and dissected; only from animals that had an 8-cell embryo were the samples collected. Thus, we evaluated the oviductal (ampulla and isthmus) environment through the use of oviductal flushing extracellular vesicles, histopathology and oviductal epithelial cell miRNAs and mRNAs to predict the biological pathways involved in this phenotype. It is important to declare that in the present work our sample number seems to be small. However, samples used in the present manuscript are from a larger sample size and only samples from pregnant animals containing an 8-cells embryo were used. Thus, the samples used here allow us to determine the physiological and molecular response related to the phenotype.

Extracellular vesicles, which compose the oviductal fluid, are key mediators of oviductal dynamism and communication between mothers and embryos, suggesting their important role in reproductive function. In the present work, the size and concentration of OF-EVs were evaluated, as was their miRNA content. We found that HBER induced the increase in AMP-EVs concentration and IST-EVs size. At this point, we suggest that the body’s energy reserve influences EVs concentration and size, and its effects remain unknown. Cells under stress conditions as oxidative and metabolic stress secretes higher EVs amounts [49,50]. Women embryos produced i*n vitro* with positive pregnancy outcomes secreted fewer EVs compared to non-pregnant women embryos [51]. The authors also observed that EVs secreted from non-pregnant women embryos were bigger that EVs from positive pregnancy outcomes [51]. The EVs size is negatively correlated with embryo quality, which increased EVs size is related to poorly embryo quality [52]. Additionally, in regard to differences in concentration, a previous study conducted by our group has demonstrated that in vitro derived embryos produce a larger number of EVs/ml in comparison to in vivo produced embryos [53]. In our case we don’t believe that the number of EVs is affected by the quality of the embryo but by the HBER environment, which is inducing an increase in the EVs in the ampulla region of the oviduct. Similarly, in another manuscript investigating the number of EVs secreted by an embryo exposed to 20% or 5% oxygen tension, we observed that in 5% O_2_ D3 embryos secreted a higher number of EVs in comparison to a 20% O_2_ environment, suggesting that the environment can influence EVs secretion [54]. Thus, due to the low vascularization in the HBER ampulla we can postulate that oviductal cells are affected by the lower O_2_ apport and are secreting a higher amount of EVs in the lumen. This finding suggested that the HBER may promote a stressful ampullary environment and influence embryonic quality in the isthmus, where the 8-cell embryo is located. Additionally, BER induced changes in the AMP-EVs miRNA content but not in the miRNA content in IST-EVs. Despite the physiological importance of oviductal EVs in reproduction, the understanding of oviductal EVs cargo from different oviductal regions is still limited. Importantly, previous studies have demonstrated that during the estrous cycle [20,21,23] and in the presence of embryos, the miRNA cargo from oviductal EVs can be modulated and that oviductal EVs can modulate metabolism-related genes in embryos upon supplementation in vitro [55]. Our data showed that BER changes the miRNA cargo in AMP-EVs. The functional enrichment analysis of differentially expressed miRNAs in AMP-EVs predicted that these miRNAs regulate pathways related to cell growth, migration, differentiation and metabolism. The upregulated miRNAs in HBER AMP-EVs (bta-miR-664a) are predicted to regulate transcripts such as INSR and GLUT4 (MirWalk version 3.0), which play important roles in insulin signaling and insulin resistance pathways. Thus, these data suggest that insulin pathway disturbance in the ampulla of HBER animals could impact oocyte fertilization and early embryo development. Importantly, analysis of oviductal EVs from the isthmus region did not reveal any differences in the levels of these miRNAs, thus supporting the idea that the major disturbance might occur in the ampulla region. Once the bovine embryo also secretes EVs [53,54], in our study the 8-cell stage embryo presence in both groups may masked a metabolic effect coming from BER in isthmus. However, EVs are composed by many other biological molecules that were not analyzed in this study, and it is possible that their profile is altered by BER.

In women and other animals, metabolic problems such as high DMI, elevated BER and hyperinsulinemia can cause remodeling in many biological tissues [56–58]. The main alteration related to metabolic problems is vascular dysfunction affecting peripheral vascular resistance and substrate delivery [57]. This is probably due to the extracellular matrix, which acts as a physical barrier for metabolite diffusion and can remodel itself in diverse situations. In response to elevated BER and in hyperinsulinemia conditions, the extracellular matrix can remodel itself and accumulate collagen deposition, increasing physical barriers to glucose, insulin and fatty acid transport and decreasing vascular delivery [58]. Additionally, maternal obesity impairs placental angiogenesis and blood vessel density, promoting hypoxia, hypoglycemia and hypoinsulinemia in fetuses [56,59–61]. However, data showing the effects of BER through oviductal histomorphology are still limited. Our data revealed that the HBER impaired ampullary vascularization. Thus, the HBER negatively affects vascularization in the ampullar region which may contribute to a harmful environment.

The cell molecular profile that composes the oviductal epithelium became an interesting type of sample for understanding the possible ways in which metabolism affects this tissue. The molecular profile of oviductal epithelial cells can be altered through the estrous cycle [62], by ovarian hormones [63], by the presence of embryos [22,23] and by lactation [64,65]. Additionally, the nutritional plan influenced the ampullary epithelial cell protein profile in goats [66]. In this way, we evaluated the miRNA content in OV-Cell to investigate the effects of BER on the molecular architecture of oviductal epithelial cells. When analyzing the OV-cell miRNA profile, we observed that BER changed miRNA expression in the ampulla and isthmus cells. There were a total of 10 miRNAs whose expression was exclusively in the HBER AMP-Cell cohort. The predicted pathways related to those miRNAs are mainly associated with metabolism, such as the glucagon and insulin signaling pathways. Interestingly, the exclusively detected bta-miR-21-3p and bta-miR-432 were predicted to regulate the INSR transcript, and miR-432 was also predicted to regulate the GLUT4 transcript (MirWalk 3.0 version), suggesting that HBER may regulate AMP-cell insulin metabolism. Our data revealed that, in the HBER ampulla, the miRNA-containing EVs are involved in insulin resistance, similar to what has been observed in AMP-Cell, and we suggest that the insulin pathway is disrupted within the ampulla of HBER animals. Briefly, according to the literature, insulin is important to maintain glucose homeostasis and regulate carbohydrate, lipid and protein metabolism influencing these macronutrients stores [67,68]. Thus, insulin signaling regulates important biological processes such as synthesis and uptake of glucose, gluconeogenesis, lipid metabolism, protein synthesis, cell growth and differentiation [69,70]. Furthermore, the expression of a total of 8 miRNAs was elevated in the HBER cohort; these miRNAs are predicted to regulate pathways related to cell proliferation, differentiation and vascularization. The VEGF signaling pathway is associated with vascular development in which its family members are glycoproteins known to regulate vasculogenesis and angiogenesis processes in embryo development and pathological conditions [71]. In bovine oviduct, the VEGF property appears to be related to vascular permeability, epithelial cell secretion and motility providing the ideal supply of factors and nutrients and gametes/embryo transport, acting as a fine regulator of the oviductal environment [72,73]. The main modulator of the VEGF expression is insulin [74], and the increase in BER is also kwon do down regulate the VEGF signaling pathway [75]. This suggests that the impaired vascularization related to the down regulation of the VEGF signaling may be associated with an insulin resistance condition. Additionally, these data corroborate our histomorphology results showing decreased vascularization in the HBER ampulla. Thus, we speculate that an impaired insulin pathway has an important role in ampullary physiology in HBER animals.

In IST-Cell, 14 miRNAs, such as those involved in endocrine resistance, insulin resistance, and insulin and glucagon signaling, were upregulate HBERs. bta-miR-20b and bta-miR-28 are predicted to regulate the INSR transcript, while bta-miR-30a-5p and bta-miR-365-5p are predicted to regulate the GLUT4 transcript (MirWalk version 3.0). This finding suggested that the regulation of insulin effects may play an important role in HBER isthmus cells. Additionally, the bta-miR-138 present only in HBER IST-Cell is predicted to be involved in proliferation inhibition [76], apoptosis induction [77], and inflammation [78]. Despite these well-established functions, studies have reported that miR-138 may be related to alterations in glycolysis [79,80]. Additionally, miR-138 expression is related to carcinogenesis due to elevated BER [81]. It is important to emphasize that the isthmus samples collected in this study had a single 8-cell embryo. At this development stage, dramatic changes occur within the embryo. Once major gene activation occurs in 8-cell embryos [82], as well as metabolic changes involved in support embryonic development. Thus, simultaneously, early embryos using pyruvate for oxidative phosphorylation slowly switch to glycolytic metabolism as the mitochondria mature [83,84]. Since these are important ultrastructural changes that can interfere with later developmental stages, the metabolic status of the developing embryo and the environment surrounding this embryo may have important contributions to the following steps.

To increase our understanding of the effects of elevated BER in OV-Cell, we used RNAseq analysis to identify DEGs. Previous studies identified a significant number of DEGs modulated by ovarian hormones [63] and embryo presence [22]. Our data revealed 11 and 17 DEGs in AMP-Cell and IST-Cells, respectively, suggesting that BER has a slight effect on the DEGs in OV-Cell. However, the impact and effects of these processes must be considered. In AMP-treated cells, the biological pathways affected by DEGs are related to cell metabolism, proliferation and development and are associated with the molecular response to the oviductal environment. According to its physiological role, after ovulation, the oviductal structure prepares itself, generating an extremely secretory environment [19]. Given the time at which the samples were collected (120 hours after ovulation induction), we expected the ampulla to change its cellular constitution, decreasing the number and activity of secretory cells and increasing the number and activity of ciliated cells [3]. However, under high BER, the natural physiological responses to these processes may be altered due to changes in the molecular response of the epithelium. Like in AMP-Cell samples, in isthmus samples, the DEGs were involved in biological pathways associated with molecular responses focused on cell metabolism. Analysis of the DEGs in IST-Cell suggested that the carbohydrate digestion and absorption pathway is affected by BER, possibly leading to alterations in sugar metabolism caused mainly by insulin, as already noted by miRNA analysis, at an important developmental period for embryonic metabolism transition [85]. In addition, for IST-Cell, the DEGs influenced pathways related to embryo transport, such as the estrogen signaling pathway and vascular smooth muscle contraction. Besides all the important functions in the reproductive tract controlled by ovarian hormones, the oviduct is responsible for the gametes and embryo transport through smooth muscle contractions, ciliated cells beating, and oviduct fluid flow [4]. Thus, estrogen can induce muscle contractions, faster ciliary beat and increase in oviductal fluid in order to advance the transport [19,86]. However, these oviductal functions must be orchestrated along with the embryo development, otherwise, the lack of synchrony between oviduct and embryo may result in ectopic pregnancy or failure in embryo development [3,4,87]. Thus, we speculate that even with a small number of DEGs in oviductal epithelial cells under different BERs, these genes might have a relevant impact on the normal physiological function of the oviduct. Therefore, our data reveal that elevated BER may alter oviductal metabolism, possibly leading to local insulin resistance, affecting normal function and, probably, embryo metabolism during early development, impacting gestational rates in these animals.

## Conclusions

HBER alters the oviductal environment and cell contents, possibly compromising oviduct physiological function. Elevated BER may impair normal embryonic development and later pregnancy stages. Our experiment’s analysis demonstrated that elevated BER induces changes in EVs size and concentration, as well as vasculature, miRNAs and transcripts within the different regions of the oviduct. Importantly, the changes appear to be associated with the time after ovulation and oviduct location. In conclusion elevated BER can negatively impact reproductive performance based due to significant changes in the oviduct.

## Supporting information

S1 Fig Oviductal flushing extracellular vesicles characterization by flow cytometry from cows with High (HBER) and moderate body energy reserve (MBER). **A.** EVs samples from different regions of oviduct, ampulla (AMP) and isthmus (IST), stained with antibodies as positive (Syntenin and CD81) and negative markers (Calnexin); the positive events are shown inside the gates created based on the unlabeled particles and negative control for each marker. **B.** Positive control with permeabilized oviductal cells; Hoechst (nuclear marker) positive events were used as inclusion factor for the analysis of Calnexin (endoplasmic reticulum marker).
(TIF)

S2 Fig Differential gene expression (DEG) in ampullary luminal epithelial cells (AMP-Cell) from cows with different body energy reserve. **A.** Volcano and smir plot representing the variation in DEGs in AMP-Cell for MBER and HBER group **B.** Heatmap showing the variation in the DEGs in AMP-Cell for MBER and HBER group. **C.** Biological pathways affected by DEGs in AMP-Cell.
(TIF)

S3 Fig Differential gene expression (DEG) in isthmic luminal epithelial cells (IST-Cell) from cows with different body energy reserve. **A.** Volcano and smear plot representing the variation in DEGs in IST-Cell for MBER and HBER group **B.** Heatmap showing the variation in the DEGs in IST-Cell for MBER and HBER group. **C.** Biological pathways affected by DEGs in IST-Cell.
(TIF)

S1 Table Flow cytometry analyses for oviductal flushing extracellular vesicles characterization from cows with different body energy reserve. (DOCX)

S2 Table Raw cycle threshold levels of the 383 miRNAs profile in ampullary extracellular vesicles (AMP-EVs) of cows with different body energy reserve. (DOCX)

S3 Table Raw cycle threshold levels of the 383 miRNAs profile in isthmic extracellular vesicles (IST-EVs) of cows with different body energy reserve. (DOCX)

S4 Table Normalized data of the 82 miRNAs commonly detected in ampullary extracellular vesicles (AMP-EVs) of cows with different body energy reserve. (DOCX)

S5 Table Normalized data of the 150 miRNAs commonly detected in isthmic extracellular vesicles (IST-EVs) of cows with different body energy reserve. (DOCX)

S6 Table Biological patwhays predicted as modulated by miRNAs up regulated in ampullary extracellular vesicles (AMP-EVs) in moderated body energy reserve (MBER) group. (DOCX)

S7 Table Biological patwhays predicted as modulated by miRNAs up regulated in isthimic extracellular vesicles (IST-EVs) in high body energy reserve (HBER) group. (DOCX)

S8 Table Raw cycle threshold levels of the 383 miRNAs profile in ampullary luminal epithelial cells (AMP-Cell) of cows with different body energy reserve. (DOCX)

S9 Table Raw cycle threshold levels of the 383 miRNAs profile in isthmic luminal epithelial cells (IST-Cell) of cows with different body energy reserve. (DOCX)

S10 Table Normalized data of the 210 miRNAs commonly detected in ampullary luminal epithelial cells (AMP-Cell) of cows with different body energy reserve. (DOCX)

S11 Table Normalized data of the 242 miRNAs commonly detected in isthmic luminal epithelial cells (IST-Cell) of cows with different body energy reserve. (DOCX)

S12 Table Biological patwhays predicted as modulated by exclusive miRNAs in ampullary luminal epithelial cells (AMP-Cell) in high body energy reserve (HBER) group. (DOCX)

S13 Table Biological patwhays predicted as modulated by miRNAs up regulated in ampullary luminal epithelial cells (AMP-Cell) in high body energy reserve (HBER) group. (DOCX)

S14 Table Biological patwhays predicted as modulated by miRNAs up regulated in isthmic luminal epithelial cells (IST-Cell) in high body energy reserve (HBER) group. (DOCX)
