# Supplementary figures and images for: The bovine oviductal environment and composition are negatively affected by elevated body energy reserves
Source: PLoS One. 2025 Jun 23;20(6):e0326138. doi: 10.1371/journal.pone.0326138 (PMC12184905; doi:10.1371/journal.pone.0326138)

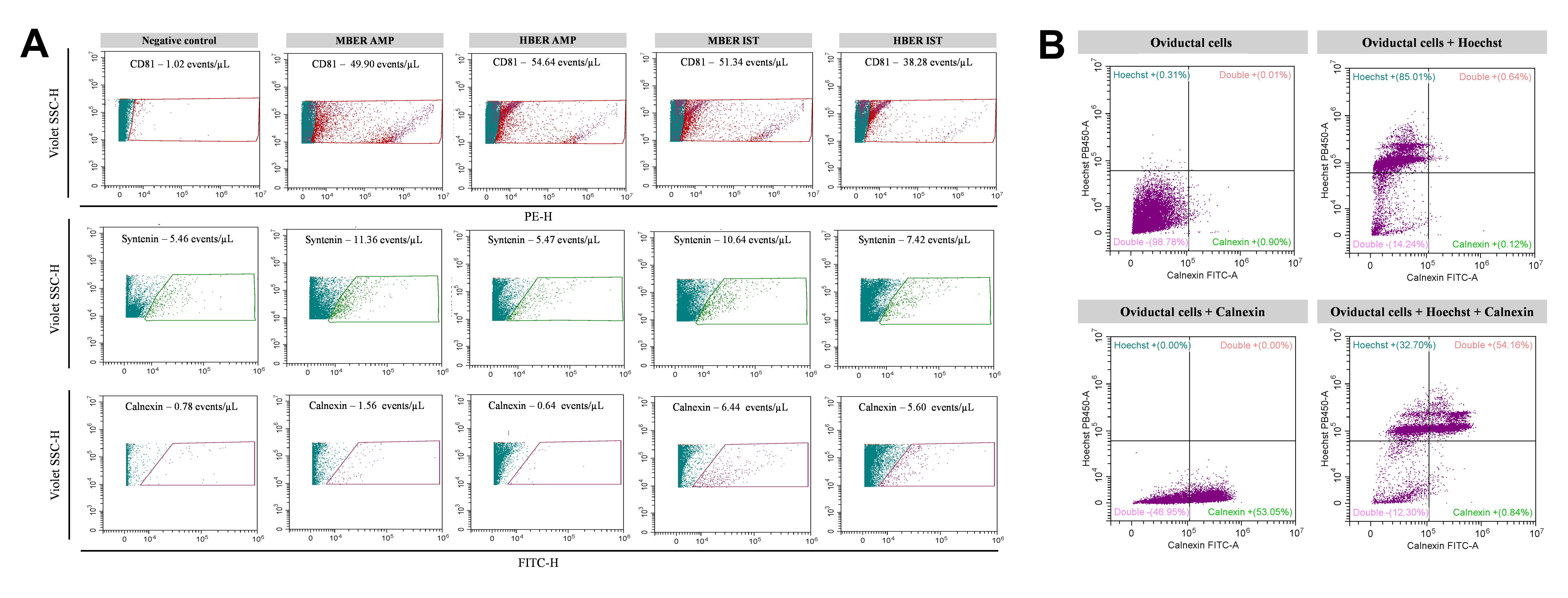

Supplement: S1 Fig — A. EVs samples from different regions of oviduct, ampulla (AMP) and isthmus (IST), stained with antibodies as positive (Syntenin and CD81) and negative markers (Calnexin); the positive events are shown inside the gates created based on the unlabeled particles and negative control for each marker. B. Positive control with permeabilized oviductal cells; Hoechst (nuclear marker) positive events were used as inclusion factor for the analysis of Calnexin (endoplasmic reticulum marker). (TIF) [file pone.0326138.s001.tif]

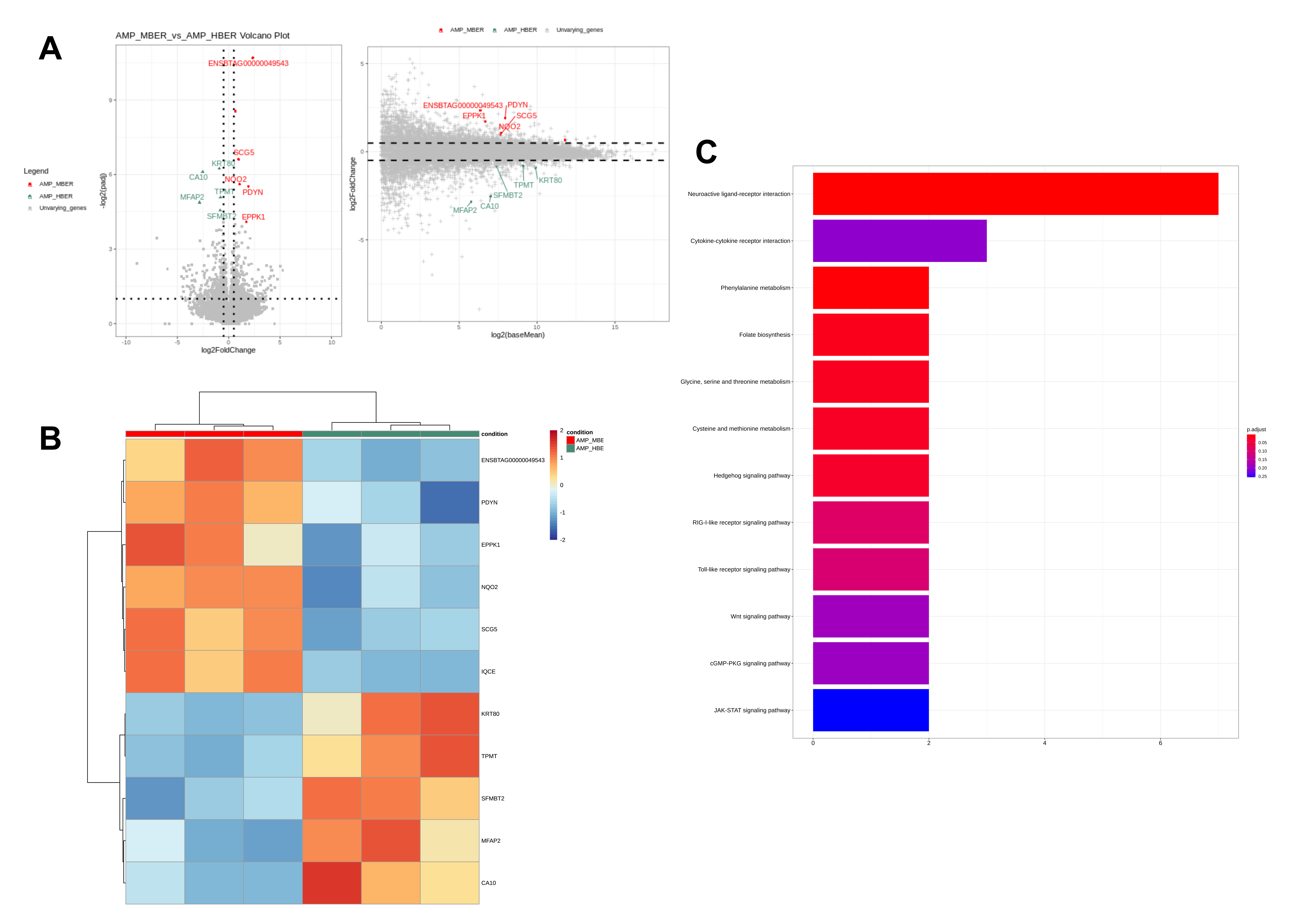

Supplement: S2 Fig — A. Volcano and smir plot representing the variation in DEGs in AMP-Cell for MBER and HBER group B. Heatmap showing the variation in the DEGs in AMP-Cell for MBER and HBER group. C. Biological pathways affected by DEGs in AMP-Cell. (TIF) [file pone.0326138.s002.tif]

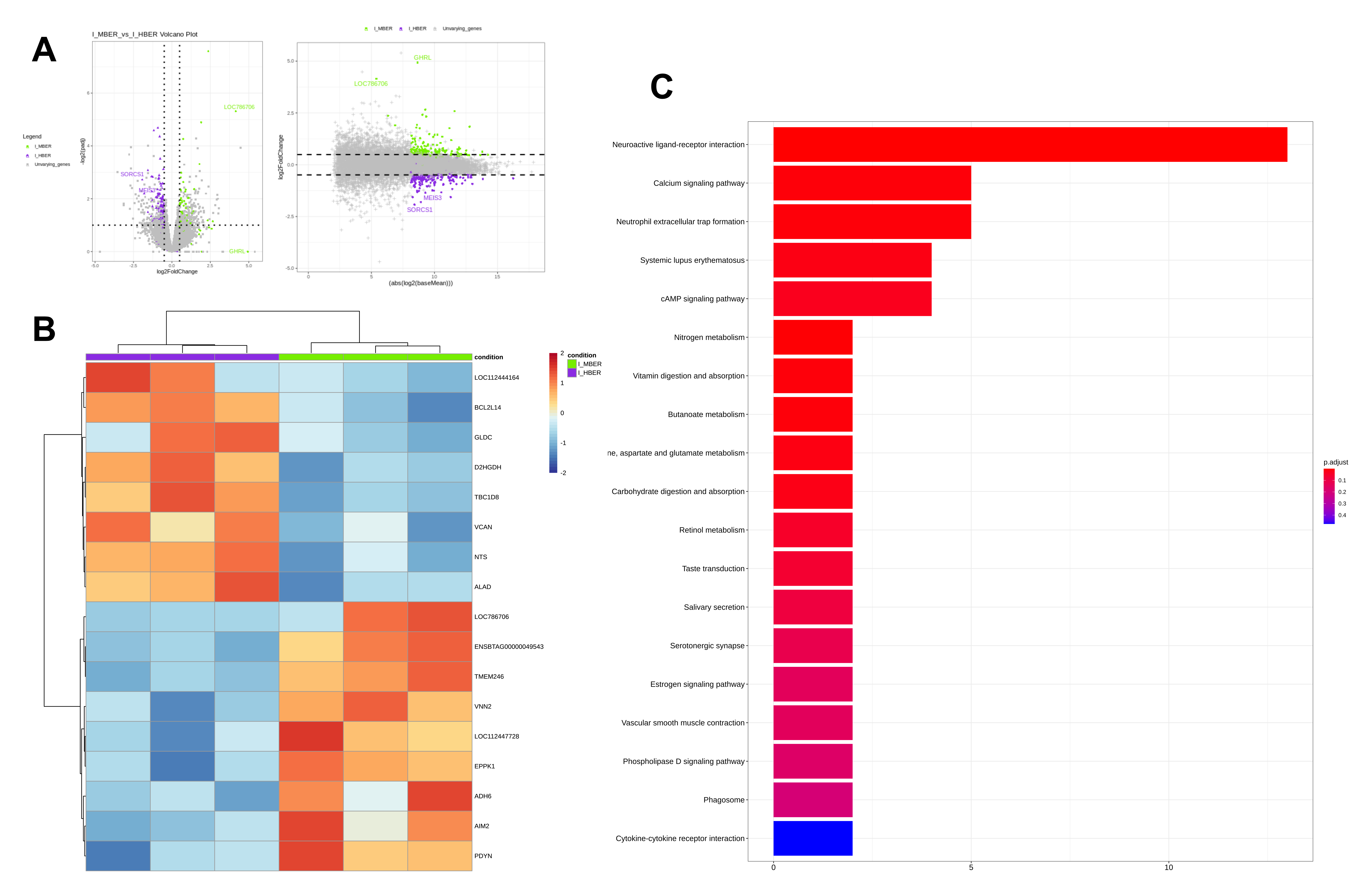

Supplement: S3 Fig — A. Volcano and smear plot representing the variation in DEGs in IST-Cell for MBER and HBER group B. Heatmap showing the variation in the DEGs in IST-Cell for MBER and HBER group. C. Biological pathways affected by DEGs in IST-Cell. (TIF) [file pone.0326138.s003.tif]
