# Supplementary material for: The bovine oviductal environment and composition are negatively affected by elevated body energy reserves
Source: PLoS One. 2025 Jun 23;20(6):e0326138. doi: 10.1371/journal.pone.0326138 (PMC12184905; doi:10.1371/journal.pone.0326138)
Supplement: S1 Table — (DOCX) [file pone.0326138.s004.docx]

| **Supplementary Table 1.** Flow cytometry analyses for oviductal flushing extracellular vesicles characterization from cows with different body energy reserve. | | | | | | |
| --- | --- | --- | --- | --- | --- | --- |
| **Antibodies^1^** | **Events/µL^2^** |  | **Body energy reserve^3^** | | | |
|  |  |  | **MBER** | | **HBER** | |
|  |  | **Negative control** | **Ampulla** | **Isthmus** | **Ampulla** | **Isthmus** |
| **CD81** | **Total** | 2072.52 | 2785.66 | 3388.79 | 3862.48 | 2708.17 |
|  | **100-240 nm** | 242.09 | 1471.52 | 595.55 | 374.43 | 332.22 |
|  | **FITC positive** | 2.48 | 19.32 | 19.64 | 24.14 | 18.20 |
|  | **PE positive** | 1.02 | 49.90 | 54.64 | 51.34 | 38.28 |
| Syntenin | **Total** | 4497.26 | 7129.54 | 7271.03 | 7808.96 | 7866.60 |
|  | **100-240 nm** | 125.33 | 275.30 | 442.51 | 425.51 | 676.66 |
|  | **FITC positive** | 5.46 | 11.36 | 5.47 | 10.64 | 7.42 |
|  | **PE positive** | 0.94 | 1.52 | 1.06 | 1.86 | 1.40 |
| Calnexin | **Total** | 5611.80 | 7650.54 | 6949.91 | 7484.09 | 7110.92 |
|  | **100-240 nm** | 44.46 | 110.40 | 317.87 | 182.00 | 368.53 |
|  | **FITC positive** | 0.78 | 1.56 | 0.64 | 6.44 | 5.60 |
|  | **PE positive** | 0.22 | 0.54 | 0.12 | 3.76 | 1.20 |
| ^1^Antigens: CD81: Specific extracellular vesicles protein; Syntenin: Specific extracellular vesicles protein; Calnexin: Cell protein, used as a positive control. ^2^Events/µl: Total: Total events per µl; 100-200 nm: Total events registered in 100-200 nm diameter; FITC positive: Events positive for fluorescein isothiocyanate; PE positive: Events positive for phycoerythrin.  ^3^Body energy reserve: MBER: Cows with moderated body energy reserve; HBER: Cows with high body energy reserve. | | | | | | |
