# Supplementary material for: The bovine oviductal environment and composition are negatively affected by elevated body energy reserves
Source: PLoS One. 2025 Jun 23;20(6):e0326138. doi: 10.1371/journal.pone.0326138 (PMC12184905; doi:10.1371/journal.pone.0326138)
Supplement: S2 Table — (DOCX) [file pone.0326138.s005.docx]

| **Supplementary Table 2.** Raw cycle threshold levels of the 383 miRNAs profile in ampullary extracellular vesicles (AMP-EVs) of cows with different body energy reserve. | | | | | | |
| --- | --- | --- | --- | --- | --- | --- |
| **miRNA** | **Body energy reserve^1^** | | | | | |
|  | **MBER** | | | **HBER** | | |
|  | **1** | **2** | **3** | **1** | **2** | **3** |
| bta-let-7a-3p | . | . | . | . | . | . |
| bta-miR-103 | . | . | 34.518 | 36.761 | 33.676 | . |
| bta-let-7a-5p | . | . | 34.751 | 35.297 | . | 35.264 |
| bta-miR-105a | . | . | . | 35.080 | 32.600 | . |
| bta-let-7b | 34.632 | 34.202 | 32.937 | 33.431 | . | 32.930 |
| bta-miR-105b | . | 35.565 | . | . | 30.540 | 32.957 |
| bta-let-7c | 34.790 | 36.019 | 32.699 | 32.809 | 34.623 | 35.017 |
| bta-miR-106a | . | 34.985 | 35.851 | 35.357 | 32.993 | 35.940 |
| bta-let-7d | . | . | . | 33.883 | . | . |
| bta-miR-106b | . | 34.129 | . | . | 33.942 | . |
| bta-let-7e | 35.329 | 33.640 | 34.926 | 33.393 | 30.727 | 33.186 |
| bta-miR-107 | . | . | . | . | 34.961 | . |
| bta-let-7f | . | 34.687 | 36.295 | . | . | . |
| bta-miR-10a | . | 34.762 | . | 33.858 | 30.475 | 33.736 |
| bta-let-7g | . | . | . | . | . | 34.903 |
| bta-miR-10b | . | . | . | . | 32.743 | 32.906 |
| bta-let-7i | . | . | . | . | 34.529 | . |
| bta-miR-122 | . | . | . | . | 32.729 | . |
| bta-miR-1 | . | . | . | 33.554 | . | . |
| bta-miR-124a | 33.884 | . | . | . | 33.742 | . |
| bta-miR-100 | . | 33.772 | 34.756 | . | 33.779 | . |
| bta-miR-124b | 32.678 | 36.117 | . | 35.023 | 33.607 | . |
| bta-miR-101 | . | 34.564 | . | . | . | . |
| bta-miR-125a | . | 32.708 | . | . | 30.781 | 33.541 |
| bta-miR-125b | . | 33.864 | 34.422 | 33.907 | . | . |
| bta-miR-133b | . | . | . | . | 34.662 | . |
| bta-miR-126-3p | 32.809 | 31.971 | 35.146 | 32.834 | 28.605 | 31.443 |
| bta-miR-133c | 33.906 | 33.865 | 35.011 | . | 29.726 | 33.203 |
| bta-miR-126-5p | 36.018 | 30.753 | 33.542 | 36.788 | 28.562 | 30.509 |
| bta-miR-134 | . | 31.277 | 38.317 | 32.777 | 28.111 | 30.157 |
| bta-miR-127 | 34.320 | 33.201 | 33.627 | 34.885 | 30.837 | 33.986 |
| bta-miR-135a | . | 36.908 | 38.325 | . | 33.855 | . |
| bta-miR-128 | . | . | . | . | . | . |
| bta-miR-135b | . | . | . | . | 28.941 | . |
| bta-miR-129 | . | . | 36.358 | . | 35.028 | 35.715 |
| bta-miR-136 | . | . | . | . | 36.421 | . |
| bta-miR-129-3p | 34.812 | 32.888 | 33.547 | . | 33.598 | 35.347 |
| bta-miR-137 | . | . | . | . | 35.272 | . |
| bta-miR-129-5p | 34.596 | 34.041 | 34.093 | 33.895 | 32.798 | 35.052 |
| bta-miR-138 | . | 27.830 | . | 30.972 | 26.587 | 28.720 |
| bta-miR-130a | 33.872 | 33.970 | 33.623 | 35.025 | 32.912 | 33.231 |
| bta-miR-139 | . | 31.929 | 34.402 | 32.300 | 30.733 | 33.900 |
| bta-miR-130b | 32.559 | 33.756 | 33.328 | 33.750 | 31.402 | 32.405 |
| bta-miR-140 | . | 33.941 | . | . | 31.731 | 33.088 |
| bta-miR-132 | 29.716 | 29.560 | 29.695 | 28.014 | 27.314 | 28.714 |
| bta-miR-141 | . | 39.979 | 34.637 | 33.199 | 31.548 | 32.648 |
| bta-miR-133a | . | 31.881 | 32.788 | 39.090 | 27.951 | 30.797 |
| bta-miR-142-3p | . | 35.518 | . | . | 32.677 | 34.256 |
| bta-miR-142-5p | . | . | . | . | 32.733 | 37.078 |
| bta-miR-151-3p | . | 34.106 | 34.480 | . | 30.729 | 34.091 |
| bta-miR-143 | 31.694 | 33.880 | 30.784 | 32.027 | 29.712 | 30.962 |
| bta-miR-151-5p | . | 34.498 | 35.275 | . | 34.965 | . |
| bta-miR-144 | . | . | . | . | 31.463 | 34.269 |
| bta-miR-152 | 34.451 | 32.912 | . | . | 28.908 | 31.946 |
| bta-miR-145 | . | 32.509 | 32.644 | 36.472 | 29.280 | 31.860 |
| bta-miR-153 | . | . | . | . | . | . |
| bta-miR-146a | . | . | . | . | 32.533 | . |
| bta-miR-154a | . | 32.610 | 31.097 | 34.090 | 28.698 | 31.404 |
| bta-miR-146b | 35.090 | . | . | . | 32.316 | 34.639 |
| bta-miR-154b | 33.962 | 28.671 | 29.749 | 32.834 | 26.066 | 28.890 |
| bta-miR-147 | . | . | . | . | . | . |
| bta-miR-154c | . | 34.182 | . | . | 31.365 | 33.571 |
| bta-miR-148a | . | 34.769 | 34.239 | . | . | . |
| bta-miR-155 | . | 33.973 | 34.289 | 37.831 | 32.094 | 34.951 |
| bta-miR-148b | . | 36.953 | 33.938 | . | . | . |
| bta-miR-15a | . | . | 34.719 | . | 34.810 | . |
| bta-miR-149-3p | 30.392 | 30.528 | 30.585 | 32.399 | 28.617 | 31.402 |
| bta-miR-15b | . | . | . | . | . | . |
| bta-miR-149-5p | . | 32.538 | 32.571 | 33.898 | 28.556 | 30.995 |
| bta-miR-16a | . | 32.928 | 33.807 | . | 31.158 | 34.747 |
| bta-miR-150 | . | 33.188 | 33.401 | 36.229 | 29.754 | 32.898 |
| bta-miR-16b | . | 32.324 | 32.825 | 34.973 | 29.709 | 32.185 |
| bta-miR-17-3p | . | . | . | . | . | . |
| bta-miR-188 | 35.693 | 35.103 | 34.003 | 33.905 | 32.407 | 33.910 |
| bta-miR-17-5p | . | . | 34.757 | 35.774 | 31.878 | . |
| bta-miR-18a | . | . | . | . | 35.337 | . |
| bta-miR-181a | . | . | . | . | . | . |
| bta-miR-18b | . | . | . | . | . | . |
| bta-miR-181b | . | 34.102 | 34.090 | 33.974 | 31.618 | . |
| bta-miR-190a | . | 36.050 | . | . | 33.984 | . |
| bta-miR-181c | . | . | . | . | . | . |
| bta-miR-190b | . | 33.857 | 35.140 | 33.929 | 33.893 | . |
| bta-miR-181d | . | 31.800 | 32.887 | 33.840 | 29.492 | 32.177 |
| bta-miR-191 | 35.361 | 31.593 | 31.861 | 31.185 | 29.095 | 30.597 |
| bta-miR-182 | . | 36.843 | 36.750 | . | 31.094 | 33.962 |
| bta-miR-192 | 35.904 | 34.097 | 34.809 | 38.528 | 32.949 | 33.507 |
| bta-miR-183 | . | 33.580 | 35.110 | . | 30.244 | 32.651 |
| bta-miR-193a | . | . | . | . | 35.995 | . |
| bta-miR-184 | . | 33.531 | 34.727 | . | 30.222 | 33.770 |
| bta-miR-193a-3p | . | . | 38.056 | . | . | . |
| bta-miR-185 | . | . | . | . | 33.972 | . |
| bta-miR-193a-5p | . | 34.095 | 33.888 | 31.866 | 32.194 | 34.729 |
| bta-miR-186 | . | 33.348 | 34.705 | . | 29.625 | 33.876 |
| bta-miR-193b | . | . | 35.586 | 38.128 | . | . |
| bta-miR-187 | 34.646 | 32.549 | 31.584 | 33.449 | 29.652 | 32.872 |
| bta-miR-194 | . | . | . | . | 34.933 | . |
| bta-miR-195 | . | 34.738 | 33.253 | . | 31.690 | . |
| bta-miR-200c | . | 32.946 | 32.412 | 33.148 | 31.071 | 31.490 |
| bta-miR-196a | 35.362 | 34.114 | 32.431 | 33.436 | 29.029 | 30.860 |
| bta-miR-202 | . | . | . | . | 34.386 | . |
| bta-miR-196b | 35.467 | 33.675 | 33.923 | 34.912 | 31.739 | 34.601 |
| bta-miR-204 | . | 34.996 | 33.093 | 34.941 | 29.013 | 32.000 |
| bta-miR-197 | 32.567 | 30.683 | 31.110 | 33.945 | 27.747 | 30.743 |
| bta-miR-205 | . | 33.749 | 32.887 | 36.855 | 29.355 | 31.845 |
| bta-miR-199a-3p | . | . | 33.591 | 34.772 | . | . |
| bta-miR-206 | . | 31.123 | 33.343 | . | 28.055 | 30.710 |
| bta-miR-199a-5p | . | . | . | . | . | . |
| bta-miR-208a | . | . | . | . | 35.597 | . |
| bta-miR-199b | . | . | . | . | 33.931 | 34.307 |
| bta-miR-208b | . | 33.219 | . | . | . | . |
| bta-miR-199c | . | 34.152 | 32.568 | 33.815 | 29.823 | 31.933 |
| bta-miR-20a | . | 32.590 | 33.731 | 34.376 | 30.673 | 33.131 |
| bta-miR-19a | . | . | . | 36.619 | 32.003 | 34.667 |
| bta-miR-20b | . | . | 35.438 | . | 32.885 | 35.963 |
| bta-miR-19b | . | 36.717 | . | . | 33.277 | 35.839 |
| bta-miR-21-3p | . | . | . | . | 34.875 | . |
| bta-miR-200a | . | 36.919 | 34.778 | . | . | . |
| bta-miR-21-5p | . | . | . | . | 33.986 | . |
| bta-miR-200b | 32.223 | 31.572 | 29.893 | 29.276 | 31.022 | 30.984 |
| bta-miR-210 | 35.530 | 34.741 | 34.564 | 32.655 | 31.713 | . |
| bta-miR-211 | . | 32.115 | 31.463 | 33.708 | 29.750 | 31.878 |
| bta-miR-22-5p | 36.890 | . | 34.284 | 34.022 | . | 35.219 |
| bta-miR-212 | 34.781 | 34.989 | . | . | 31.651 | 33.783 |
| bta-miR-221 | . | . | 35.060 | 35.213 | 29.682 | . |
| bta-miR-214 | 32.729 | 36.796 | 34.230 | 35.600 | 32.539 | . |
| bta-miR-222 | 34.237 | 32.668 | 33.787 | 32.882 | 30.300 | 32.856 |
| bta-miR-215 | . | 33.415 | 33.115 | . | 32.965 | . |
| bta-miR-223 | . | 34.381 | 32.058 | . | 30.180 | 34.721 |
| bta-miR-216a | . | . | . | 35.238 | 38.024 | . |
| bta-miR-224 | . | . | . | . | . | 35.260 |
| bta-miR-216b | . | . | 34.539 | . | 34.164 | . |
| bta-miR-23a | 35.373 | 32.240 | 30.806 | 31.137 | 32.236 | 32.729 |
| bta-miR-217 | . | . | . | 36.704 | . | . |
| bta-miR-23b-3p | . | 34.087 | 32.462 | 34.386 | 30.677 | 34.619 |
| bta-miR-218 | . | 34.053 | . | . | 32.294 | 36.501 |
| bta-miR-23b-5p | . | 34.976 | . | 34.466 | 33.068 | 34.320 |
| bta-miR-219 | 35.825 | 31.870 | 31.530 | 32.779 | 28.649 | 30.540 |
| bta-miR-24 | . | . | . | . | 34.979 | . |
| bta-miR-219-3p | . | 34.834 | 34.864 | 35.272 | 31.839 | 33.848 |
| bta-miR-24-3p | . | 34.437 | . | 29.709 | . | 33.971 |
| bta-miR-219-5p | . | . | . | . | . | . |
| bta-miR-25 | . | 33.335 | 34.567 | 35.430 | 33.846 | . |
| bta-miR-22-3p | 2.823 | 2.682 | 2.766 | 2.810 | 2.679 | 2.393 |
| bta-miR-26a | . | 32.832 | 30.825 | 31.558 | 29.076 | 30.261 |
| bta-miR-26b | . | 35.845 | . | 33.859 | 32.779 | . |
| bta-miR-29d-3p | . | . | . | 35.871 | . | . |
| bta-miR-26c | . | . | . | . | . | . |
| bta-miR-29d-5p | . | 35.334 | 35.060 | . | . | . |
| bta-miR-27a-3p | . | 31.744 | 30.704 | 32.107 | 28.431 | 30.156 |
| bta-miR-29e | . | . | . | . | . | . |
| bta-miR-27a-5p | 8.599 | 8.632 | 8.796 | 8.373 | 8.489 | 8.489 |
| bta-miR-301a | . | 33.694 | 38.287 | . | 34.381 | . |
| bta-miR-27b | . | . | 33.220 | . | 32.815 | 34.369 |
| bta-miR-301b | . | 39.946 | . | . | . | . |
| bta-miR-28 | . | . | 34.334 | . | . | . |
| bta-miR-302a | . | . | . | . | 33.745 | . |
| bta-miR-296-3p | 33.682 | 31.755 | 30.402 | 38.177 | 30.638 | 35.254 |
| bta-miR-302b | . | 34.651 | . | . | 33.799 | . |
| bta-miR-296-5p | . | 32.384 | 31.797 | 33.059 | 28.886 | 32.743 |
| bta-miR-302c | . | 33.970 | 33.125 | . | 30.383 | 33.602 |
| bta-miR-299 | . | 35.043 | . | . | 31.876 | 33.650 |
| bta-miR-302d | . | . | . | . | . | . |
| bta-miR-29a | 34.439 | 34.195 | 34.838 | 30.683 | 34.928 | 33.792 |
| bta-miR-3064 | . | 34.333 | . | . | 32.645 | 44.188 |
| bta-miR-29b | . | . | . | . | 33.723 | . |
| bta-miR-30a-5p | . | 32.717 | 33.732 | 34.674 | 31.440 | 33.496 |
| bta-miR-29c | 34.590 | 36.829 | 35.026 | 32.695 | 35.435 | . |
| bta-miR-30b-3p | 35.722 | . | . | . | . | . |
| bta-miR-30b-5p | . | 31.214 | 35.043 | 43.045 | 30.817 | 34.700 |
| bta-miR-328 | 30.869 | 29.200 | 30.608 | 35.022 | 29.259 | 31.623 |
| bta-miR-30c | . | 33.031 | 32.816 | . | 29.721 | 32.161 |
| bta-miR-329a | . | . | . | . | . | . |
| bta-miR-30d | . | 32.829 | 34.145 | 36.060 | 31.156 | . |
| bta-miR-329b | . | . | . | . | . | . |
| bta-miR-30e-5p | 34.184 | 36.391 | 33.854 | 38.028 | 31.908 | 33.158 |
| bta-miR-330 | . | 36.537 | 33.625 | 36.068 | 32.705 | . |
| bta-miR-30f | . | 32.793 | 33.576 | . | 29.885 | 32.112 |
| bta-miR-331-3p | . | . | . | . | 35.560 | . |
| bta-miR-31 | 35.062 | 32.330 | 32.574 | 35.623 | 32.746 | . |
| bta-miR-331-5p | 34.364 | 31.107 | 33.186 | 35.655 | 29.065 | 32.858 |
| bta-miR-32 | . | . | . | . | 36.281 | 42.902 |
| bta-miR-335 | . | 33.797 | 35.109 | . | 32.865 | 36.335 |
| bta-miR-320a | 30.649 | 30.113 | 30.799 | 29.176 | 30.712 | 32.180 |
| bta-miR-338 | . | . | . | . | . | . |
| bta-miR-320b | 33.929 | 32.810 | 33.063 | 33.680 | 30.256 | 31.930 |
| bta-miR-339a | . | 36.786 | . | . | 30.383 | 33.624 |
| bta-miR-323 | 19.181 | 18.600 | 19.330 | 18.984 | 18.811 | 19.004 |
| bta-miR-339b | . | 31.239 | 31.743 | 32.867 | 28.721 | 32.854 |
| bta-miR-324 | . | 32.585 | 32.024 | 35.473 | 29.676 | 33.667 |
| bta-miR-33a | . | 34.938 | 34.186 | 36.925 | . | . |
| bta-miR-326 | . | 36.577 | 34.473 | . | 32.167 | 36.784 |
| bta-miR-33b | 35.327 | . | 36.042 | . | 36.035 | 36.724 |
| bta-miR-340 | . | 32.152 | 31.527 | . | 28.964 | 34.189 |
| bta-miR-365-3p | . | . | . | . | 35.726 | . |
| bta-miR-342 | . | 31.364 | 32.643 | 35.056 | 29.617 | 34.491 |
| bta-miR-365-5p | . | 33.532 | 36.340 | 33.306 | 30.597 | 34.215 |
| bta-miR-345-3p | . | . | 34.240 | 34.100 | 33.930 | . |
| bta-miR-367 | . | . | . | 36.864 | . | . |
| bta-miR-345-5p | 34.100 | 33.457 | 32.945 | 34.193 | 30.762 | 32.618 |
| bta-miR-369-3p | . | . | . | . | . | . |
| bta-miR-346 | . | 31.858 | 32.800 | 31.833 | 30.373 | 33.459 |
| bta-miR-369-5p | . | 34.343 | 33.523 | . | 29.791 | 32.440 |
| bta-miR-34a | . | 33.341 | . | . | 32.882 | . |
| bta-miR-370 | 35.330 | 32.018 | 34.650 | 34.816 | 32.127 | 35.014 |
| bta-miR-34b | . | 34.776 | . | . | 33.481 | . |
| bta-miR-371 | . | 34.451 | . | . | 31.637 | . |
| bta-miR-34c | . | . | 34.809 | . | 34.938 | . |
| bta-miR-374a | . | . | . | 35.620 | 35.830 | . |
| bta-miR-361 | . | . | 34.877 | 36.962 | 35.669 | . |
| bta-miR-374b | . | 34.941 | 35.533 | 34.851 | 33.299 | 34.408 |
| bta-miR-362-3p | . | . | . | . | . | . |
| bta-miR-375 | 34.110 | 33.401 | 31.936 | 32.485 | 27.805 | 34.220 |
| bta-miR-362-5p | . | . | 34.980 | . | 32.940 | . |
| bta-miR-376a | . | 33.790 | . | . | 30.122 | 33.050 |
| bta-miR-363 | . | . | 34.801 | . | 32.561 | 33.597 |
| bta-miR-376b | . | . | . | . | . | . |
| bta-miR-376c | . | . | . | . | . | . |
| bta-miR-382 | 33.637 | 30.578 | 30.409 | 33.798 | 28.176 | 31.447 |
| bta-miR-376d | . | 33.771 | 34.470 | . | 31.843 | . |
| bta-miR-383 | . | 33.847 | 31.886 | 38.380 | 29.533 | 32.895 |
| bta-miR-376e | . | . | . | . | 34.870 | . |
| bta-miR-409a | 37.538 | 35.430 | 35.388 | . | 33.540 | . |
| bta-miR-377 | . | . | . | . | . | 36.175 |
| bta-miR-409b | . | 33.078 | . | . | 30.729 | 35.215 |
| bta-miR-378 | . | 32.781 | 31.821 | 33.560 | 28.483 | 33.669 |
| bta-miR-410 | . | 34.352 | 34.072 | . | 32.887 | . |
| bta-miR-378b | 34.250 | 31.997 | 31.924 | . | 28.652 | 31.842 |
| bta-miR-411a | 33.323 | 31.613 | 30.634 | 33.638 | 28.168 | 30.897 |
| bta-miR-378c | 36.738 | 36.238 | 33.426 | . | 34.139 | . |
| bta-miR-411b | 34.200 | 32.872 | 31.547 | 33.710 | 30.386 | 34.346 |
| bta-miR-378d | 33.996 | 32.417 | 32.316 | 34.915 | 29.740 | 31.571 |
| bta-miR-411c-3p | . | . | . | . | 36.054 | . |
| bta-miR-379 | . | . | . | . | 35.395 | . |
| bta-miR-411c-5p | . | . | . | . | 36.877 | . |
| bta-miR-380-3p | . | . | . | . | 31.058 | . |
| bta-miR-412 | . | 34.581 | . | . | 32.210 | . |
| bta-miR-380-5p | 31.810 | 31.800 | 29.693 | 33.849 | 27.925 | 30.749 |
| bta-miR-421 | 36.488 | 31.866 | 32.158 | 32.658 | 28.586 | 31.757 |
| bta-miR-381 | . | . | . | . | . | . |
| bta-miR-423-3p | . | 36.066 | 33.736 | 34.106 | 34.183 | . |
| bta-miR-423-5p | . | . | . | 34.977 | 35.532 | . |
| bta-miR-449c | . | 33.190 | 34.393 | 35.607 | 31.739 | 33.803 |
| bta-miR-424-3p | . | . | . | . | . | . |
| bta-miR-449d | . | 33.107 | 32.767 | 33.384 | 31.749 | 33.686 |
| bta-miR-424-5p | . | . | . | 33.532 | . | . |
| bta-miR-450a | . | . | . | . | . | . |
| bta-miR-425-3p | 32.793 | 31.796 | 30.714 | 30.752 | 28.890 | 31.219 |
| bta-miR-450b | . | . | 36.498 | . | . | . |
| bta-miR-425-5p | . | . | 34.875 | 34.549 | 35.365 | . |
| bta-miR-451 | . | 33.261 | . | 32.664 | . | . |
| bta-miR-429 | 30.733 | 31.330 | 29.953 | 31.620 | 28.882 | 30.780 |
| bta-miR-452 | . | . | 33.070 | . | 31.613 | 34.539 |
| bta-miR-431 | . | . | 36.117 | 34.627 | 33.817 | . |
| bta-miR-4523 | . | 32.482 | 29.862 | 34.823 | 31.866 | 36.992 |
| bta-miR-432 | 33.855 | 30.780 | 30.588 | 36.427 | 28.130 | 30.258 |
| bta-miR-453 | 35.348 | 34.947 | 35.474 | 34.669 | 32.819 | 36.060 |
| bta-miR-433 | 33.232 | 29.087 | 28.215 | 31.149 | 26.494 | 29.849 |
| bta-miR-454 | . | 31.582 | 31.204 | . | 23.887 | 32.807 |
| bta-miR-448 | . | . | . | . | 33.740 | . |
| bta-miR-455-3p | . | 32.872 | 32.841 | . | 29.684 | 33.954 |
| bta-miR-449a | 34.088 | 35.318 | . | . | 33.919 | . |
| bta-miR-455-5p | . | . | . | . | 34.240 | . |
| bta-miR-449b | . | 35.995 | 35.009 | . | 35.641 | . |
| bta-miR-483 | . | . | . | . | 32.517 | . |
| bta-miR-484 | . | . | . | . | 34.851 | . |
| bta-miR-496 | 35.496 | . | 34.547 | . | 30.935 | . |
| bta-miR-485 | . | . | 34.549 | 34.562 | 32.739 | 36.730 |
| bta-miR-497 | . | . | . | . | 36.494 | 34.507 |
| bta-miR-486 | 35.856 | 33.890 | 35.702 | 31.922 | 31.354 | 34.315 |
| bta-miR-499 | . | . | . | . | . | . |
| bta-miR-487a | . | . | 34.945 | . | 33.235 | . |
| bta-miR-500 | 33.934 | 36.710 | 34.000 | 32.257 | 35.486 | 35.591 |
| bta-miR-487b | . | 35.579 | 35.879 | . | 33.246 | 34.732 |
| bta-miR-502a | . | . | . | . | 33.900 | . |
| bta-miR-488 | . | 32.888 | 33.530 | . | 30.717 | 34.964 |
| bta-miR-502b | 33.980 | 32.034 | 32.262 | . | 31.891 | 33.590 |
| bta-miR-489 | 33.877 | 33.146 | 33.276 | 33.896 | 32.981 | 33.381 |
| bta-miR-503-3p | 34.723 | 31.661 | 31.091 | 33.310 | 28.727 | 31.622 |
| bta-miR-490 | . | 34.365 | 33.511 | . | 30.758 | . |
| bta-miR-503-5p | 8.329 | 34.852 | 34.368 | 33.872 | 33.386 | . |
| bta-miR-491 | . | 34.146 | 33.789 | 34.889 | 30.201 | 32.919 |
| bta-miR-504 | . | . | 34.851 | . | 34.400 | . |
| bta-miR-493 | . | 31.725 | 31.840 | 33.591 | 30.514 | 31.754 |
| bta-miR-505 | 34.398 | 32.924 | 32.553 | 36.508 | 30.624 | . |
| bta-miR-494 | 28.972 | 27.123 | 27.790 | 29.456 | 28.938 | 30.083 |
| bta-miR-532 | 36.666 | 32.197 | 31.438 | . | 29.507 | 32.040 |
| bta-miR-495 | . | . | . | . | . | . |
| bta-miR-539 | . | 33.263 | 34.583 | . | 30.611 | . |
| bta-miR-541 | 33.191 | 31.179 | 31.098 | 31.128 | 28.791 | 31.756 |
| bta-miR-582 | . | . | . | . | . | . |
| bta-miR-542-5p | . | 34.399 | . | . | 31.742 | . |
| bta-miR-584 | 28.706 | 28.825 | 27.752 | 25.796 | 29.385 | 28.652 |
| bta-miR-543 | . | . | . | . | . | . |
| bta-miR-592 | . | 36.846 | . | . | 32.947 | . |
| bta-miR-544a | . | . | . | . | . | . |
| bta-miR-599 | . | . | . | . | . | . |
| bta-miR-544b | . | . | . | . | . | . |
| bta-miR-615 | 12.365 | 12.258 | 12.393 | 12.197 | 12.459 | 12.531 |
| bta-miR-545-3p | . | . | . | . | . | . |
| bta-miR-628 | 35.184 | 31.803 | 31.220 | . | 29.181 | 33.615 |
| bta-miR-545-5p | . | 34.393 | . | . | 35.390 | . |
| bta-miR-631 | 20.314 | 19.721 | 20.427 | 20.540 | 19.749 | 20.220 |
| bta-miR-551a | . | 35.301 | . | . | 33.608 | 34.681 |
| bta-miR-652 | . | . | 36.886 | 35.497 | 31.730 | 39.695 |
| bta-miR-551b | . | . | . | 35.046 | 33.164 | . |
| bta-miR-653 | . | . | . | . | 33.046 | . |
| bta-miR-562 | . | . | . | . | . | . |
| bta-miR-654 | . | . | . | 34.714 | 31.859 | 37.725 |
| bta-miR-568 | . | . | . | . | . | . |
| bta-miR-655 | . | 34.890 | 32.877 | . | 30.810 | 34.911 |
| bta-miR-574 | 32.918 | 30.661 | 29.318 | 27.965 | 30.375 | 32.000 |
| bta-miR-656 | 35.891 | 31.834 | 30.824 | 34.303 | 29.280 | 32.817 |
| bta-miR-658 | . | . | 36.969 | 33.987 | 31.670 | 35.942 |
| bta-miR-758 | . | 33.444 | 33.478 | . | 32.245 | 35.743 |
| bta-miR-660 | . | 32.795 | 31.778 | . | 29.214 | 32.834 |
| bta-miR-759 | . | . | . | . | . | . |
| bta-miR-664a | 33.899 | 34.488 | 34.366 | 32.957 | 32.393 | 33.635 |
| bta-miR-760-3p | 34.233 | 34.958 | 33.355 | 31.791 | 32.448 | 33.917 |
| bta-miR-664b | 34.797 | 34.890 | 34.017 | 32.798 | 32.422 | . |
| bta-miR-760-5p | 33.865 | 30.813 | 29.752 | 33.512 | 28.016 | 30.881 |
| bta-miR-665 | . | 34.901 | 33.942 | 32.358 | 31.380 | 33.469 |
| bta-miR-761 | . | . | 34.115 | 36.049 | 35.886 | . |
| bta-miR-669 | . | 32.149 | 31.530 | 34.211 | 30.799 | . |
| bta-miR-763 | . | 31.824 | 32.124 | 34.913 | 30.597 | 33.951 |
| bta-miR-670 | . | 32.922 | 34.274 | . | 33.633 | 34.925 |
| bta-miR-764 | . | 32.948 | 35.039 | . | 31.727 | . |
| bta-miR-671 | . | . | 36.110 | . | . | . |
| bta-miR-767 | 33.929 | 33.170 | 32.071 | 34.002 | 32.519 | 34.099 |
| bta-miR-677 | . | 36.714 | 35.609 | 43.593 | . | 36.388 |
| bta-miR-769 | 36.128 | 33.614 | 33.226 | . | 30.811 | . |
| bta-miR-7 | . | . | . | . | . | . |
| bta-miR-873 | . | 32.369 | 32.899 | . | 30.957 | 33.813 |
| bta-miR-708 | 34.596 | 31.987 | 31.805 | 36.149 | 30.657 | 32.899 |
| bta-miR-874 | 33.127 | 33.544 | 32.950 | 31.781 | 34.826 | . |
| bta-miR-744 | 38.312 | . | 33.107 | 39.681 | 32.855 | 36.974 |
| bta-miR-875 | . | 35.406 | 35.484 | . | 32.326 | . |
| bta-miR-876 | . | . | 33.551 | . | 33.807 | . |
| bta-miR-98 | . | . | 34.604 | . | . | . |
| bta-miR-877 | 31.907 | 31.066 | 30.680 | 30.845 | 30.515 | 31.880 |
| bta-miR-99a-3p | . | 31.817 | 30.612 | 33.633 | 30.175 | 33.553 |
| bta-miR-885 | . | 31.090 | 30.709 | 31.599 | 29.550 | 32.504 |
| bta-miR-99a-5p | . | 34.616 | 32.201 | 33.811 | 33.782 | 36.117 |
| bta-miR-9-3p | . | . | 36.824 | . | . | . |
| bta-miR-99b | 23.718 | 23.178 | 23.734 | 24.117 | 23.310 | 23.718 |
| bta-miR-9-5p | 34.481 | 33.938 | 32.573 | 35.125 | 31.872 | 34.317 |
| bta-miR-1179 | . | 36.699 | 32.979 | . | 31.780 | 34.051 |
| bta-miR-92a | 34.996 | 31.881 | 30.770 | 33.697 | 31.175 | . |
| bta-miR-1185 | . | . | . | . | . | . |
| bta-miR-92b | 31.773 | 29.291 | 29.156 | 30.845 | 28.447 | 30.085 |
| bta-miR-1193 | . | . | . | . | . | . |
| bta-miR-93 | 34.769 | 33.808 | 32.861 | 32.673 | 34.493 | 34.805 |
| bta-miR-1197 | . | 34.306 | 32.950 | . | 33.452 | 34.881 |
| bta-miR-935 | . | 32.886 | 31.508 | 34.026 | 29.793 | 32.328 |
| bta-miR-122 | . | 34.587 | . | . | . | . |
| bta-miR-940 | 32.009 | 30.705 | 31.479 | 29.604 | 31.829 | 32.893 |
| bta-miR-1224 | 24.900 | 25.713 | 27.910 | 29.959 | 29.310 | 30.647 |
| bta-miR-95 | . | . | 33.438 | . | 32.925 | . |
| bta-miR-1225-3p | 33.011 | 32.420 | 31.850 | 31.898 | 31.047 | 32.988 |
| bta-miR-96 | . | . | . | . | . | . |
| bta-miR-1246 | 23.722 | 24.838 | 26.603 | 25.687 | 27.098 | 28.811 |
| bta-miR-1247-3p | 32.742 | 31.118 | 28.761 | 29.821 | 28.145 | 30.858 |
| bta-miR-1296 | . | 33.039 | 30.471 | 36.794 | 29.795 | 33.482 |
| bta-miR-1247-5p | 32.937 | 32.258 | 31.801 | 31.993 | 32.896 | 35.039 |
| bta-miR-1298 | . | 34.731 | . | . | . | . |
| bta-miR-1248 | . | . | . | . | 35.369 | . |
| bta-miR-1301 | . | 34.466 | 35.030 | 34.912 | 37.031 | . |
| bta-miR-1249 | . | 33.911 | 35.021 | . | 32.989 | . |
| bta-miR-1306 | . | 31.871 | 30.615 | 35.299 | 29.533 | 32.304 |
| bta-miR-1260b | 27.401 | 25.752 | 23.817 | 26.847 | 27.211 | 27.710 |
| bta-miR-1307 | 34.822 | 32.335 | 31.669 | 32.587 | 31.158 | 33.927 |
| bta-miR-1271 | . | . | . | . | 32.903 | . |
| bta-miR-1343-3p | 32.661 | 31.478 | 29.529 | 32.661 | 30.365 | 32.447 |
| bta-miR-1277 | . | . | . | . | . | . |
| bta-miR-1343-5p | 31.741 | 31.325 | 31.284 | 30.624 | 30.797 | 33.935 |
| bta-miR-1281 | 30.822 | 31.613 | 29.405 | 30.435 | 28.264 | 31.439 |
| bta-miR-1388-3p | 32.396 | 33.790 | 31.570 | 34.049 | 31.379 | 33.982 |
| bta-miR-1282 | . | . | 36.964 | 35.906 | . | . |
| RNT43 snoRNA | 36.013 | . | 32.870 | 30.826 | 32.828 | . |
| bta-miR-1284 | . | . | . | . | 33.310 | . |
| Hm/Ms/Rt T1 snRNA | 22.121 | 23.108 | 24.833 | 24.953 | 24.086 | 27.795 |
| bta-miR-1287 | 35.394 | 33.569 | 33.108 | 33.396 | 32.945 | 36.599 |
| bta-miR-99b | 23.738 | 22.997 | 23.288 | 23.916 | 23.135 | 23.751 |
| bta-miR-1291 | . | 35.553 | 32.033 | 35.101 | . | . |
| Negative control | . | . | . | . | . | . |
| ^1^Body energy reserve: MBER: Cows with moderated body energy reserve; HBER: Cows with high body energy reserve. | | | | | | |
