# Supplementary material for: The bovine oviductal environment and composition are negatively affected by elevated body energy reserves
Source: PLoS One. 2025 Jun 23;20(6):e0326138. doi: 10.1371/journal.pone.0326138 (PMC12184905; doi:10.1371/journal.pone.0326138)
Supplement: S3 Table — (DOCX) [file pone.0326138.s006.docx]

| **Supplementary Table 3.** Raw cycle threshold levels of the 383 miRNAs profile in isthmic extracellular vesicles (IST-EVs) of cows with different body energy reserve. | | | | | | |
| --- | --- | --- | --- | --- | --- | --- |
| **miRNA** | **Body energy reserve^1^** | | | | | |
|  | **MBER** | | | **HBER** | | |
|  | **1** | **2** | **3** | **1** | **2** | **3** |
| bta-let-7a-3p | 30.912 | . | . | . | . | . |
| bta-miR-103 | 34.695 | 34.376 | 32.239 | 36.857 | 32.349 | 34.130 |
| bta-let-7a-5p | 29.729 | 30.657 | 29.096 | 30.277 | 30.139 | 31.654 |
| bta-miR-105a | . | 34.168 | . | . | 38.184 | . |
| bta-let-7b | 30.701 | 31.168 | 29.565 | 29.822 | 29.711 | 32.994 |
| bta-miR-105b | 35.809 | 36.879 | 33.952 | . | 33.951 | . |
| bta-let-7c | 29.545 | 29.777 | 29.345 | 29.695 | . | 31.467 |
| bta-miR-106a | . | 34.753 | 32.686 | 34.743 | 32.941 | 35.383 |
| bta-let-7d | 30.784 | 32.269 | 31.683 | 33.311 | . | 34.876 |
| bta-miR-106b | 32.899 | 31.484 | 33.108 | 31.789 | 32.746 | 33.322 |
| bta-let-7e | 29.193 | 30.203 | 29.262 | 30.011 | 31.875 | 31.728 |
| bta-miR-107 | . | . | . | . | . | . |
| bta-let-7f | 30.925 | 32.137 | 30.481 | 33.456 | 31.259 | 34.192 |
| bta-miR-10a | 34.560 | 34.911 | 31.090 | 32.899 | 31.893 | 33.817 |
| bta-let-7g | 35.953 | 34.366 | 32.417 | . | 32.868 | . |
| bta-miR-10b | 33.910 | 36.377 | 32.862 | . | 31.194 | 34.382 |
| bta-let-7i | . | 32.861 | 32.842 | 36.339 | 31.982 | . |
| bta-miR-122 | . | 34.927 | . | 35.472 | . | . |
| bta-miR-1 | 31.381 | 30.412 | 25.670 | 33.345 | 28.407 | 30.427 |
| bta-miR-124a | 33.134 | 32.259 | 32.713 | 32.818 | 33.430 | 32.244 |
| bta-miR-100 | 30.732 | 33.559 | 30.750 | 31.874 | 31.286 | 34.945 |
| bta-miR-124b | 31.935 | 32.089 | 32.832 | 33.840 | 32.927 | 32.530 |
| bta-miR-101 | . | 34.438 | 31.036 | . | 32.397 | 34.782 |
| bta-miR-125a | 33.828 | 34.730 | 32.908 | 33.883 | 32.209 | . |
| bta-miR-125b | 30.839 | 30.711 | 29.306 | 31.847 | 28.407 | 32.690 |
| bta-miR-133b | 35.110 | 34.110 | 31.440 | . | 32.356 | 34.896 |
| bta-miR-126-3p | 32.327 | 30.676 | 28.736 | 31.532 | 28.063 | 32.289 |
| bta-miR-133c | . | 35.506 | . | 34.290 | 35.054 | . |
| bta-miR-126-5p | 33.241 | 32.046 | 29.562 | 32.205 | 29.383 | 32.929 |
| bta-miR-134 | 34.045 | 33.503 | 36.551 | 32.766 | 35.060 | 33.456 |
| bta-miR-127 | 30.698 | 29.990 | 30.832 | 31.253 | 30.471 | 30.777 |
| bta-miR-135a | 33.087 | 36.448 | 35.600 | 33.547 | 33.241 | . |
| bta-miR-128 | . | 34.320 | . | . | . | 34.672 |
| bta-miR-135b | 36.649 | . | . | 35.694 | 35.661 | . |
| bta-miR-129 | 32.882 | 31.593 | 33.799 | 31.468 | 33.966 | 31.858 |
| bta-miR-136 | . | . | . | . | . | . |
| bta-miR-129-3p | . | . | . | 35.504 | . | . |
| bta-miR-137 | . | . | . | . | . | 36.088 |
| bta-miR-129-5p | 32.862 | 31.351 | 35.156 | 30.795 | 32.851 | 32.876 |
| bta-miR-138 | 33.688 | 33.884 | 35.629 | 32.914 | 34.689 | 33.930 |
| bta-miR-130a | 30.892 | 31.074 | 31.505 | 30.870 | 30.800 | 30.712 |
| bta-miR-139 | 30.724 | 28.718 | 31.915 | 29.816 | 31.903 | 31.036 |
| bta-miR-130b | 30.586 | 30.537 | 30.445 | 30.574 | 30.292 | 30.683 |
| bta-miR-140 | . | 35.341 | . | 33.952 | 36.051 | . |
| bta-miR-132 | 31.482 | 29.091 | 31.824 | 29.167 | 30.841 | 31.505 |
| bta-miR-141 | 32.845 | 33.491 | . | 33.882 | 34.175 | 36.953 |
| bta-miR-133a | 31.838 | 31.478 | 26.602 | 34.220 | 28.585 | 30.747 |
| bta-miR-142-3p | 34.903 | . | . | . | 38.475 | . |
| bta-miR-142-5p | . | 34.890 | . | . | . | . |
| bta-miR-151-3p | 35.104 | . | 33.895 | 34.125 | 33.851 | . |
| bta-miR-143 | 32.295 | 30.747 | 30.695 | 31.619 | 30.655 | 32.480 |
| bta-miR-151-5p | 33.870 | 33.854 | 31.844 | 33.852 | 30.750 | 35.784 |
| bta-miR-144 | . | . | . | 35.693 | . | . |
| bta-miR-152 | 36.266 | 36.291 | 35.855 | 36.369 | 35.379 | . |
| bta-miR-145 | 33.028 | . | 32.440 | 31.826 | 30.868 | . |
| bta-miR-153 | . | . | . | . | . | . |
| bta-miR-146a | . | . | . | . | 34.831 | . |
| bta-miR-154a | 33.507 | 34.855 | 35.202 | 35.191 | 34.086 | 36.299 |
| bta-miR-146b | . | . | . | . | 36.720 | . |
| bta-miR-154b | 29.499 | 30.229 | 30.132 | 29.731 | 29.597 | 30.387 |
| bta-miR-147 | . | . | 35.549 | . | 35.038 | 23.662 |
| bta-miR-154c | 34.420 | 35.268 | 34.925 | 35.043 | 33.391 | 33.390 |
| bta-miR-148a | 32.932 | 32.297 | 31.767 | 33.738 | 30.474 | 34.967 |
| bta-miR-155 | . | 33.826 | . | 33.955 | . | 36.801 |
| bta-miR-148b | 33.964 | 33.892 | 32.963 | 32.844 | 31.014 | 34.349 |
| bta-miR-15a | 33.627 | . | 31.548 | 34.752 | 31.745 | 34.654 |
| bta-miR-149-3p | 29.739 | 30.380 | 30.240 | 29.824 | 30.227 | 29.725 |
| bta-miR-15b | . | 35.026 | 32.828 | 35.054 | 36.544 | 34.925 |
| bta-miR-149-5p | . | 34.133 | . | . | . | 35.396 |
| bta-miR-16a | 33.278 | 33.737 | 29.811 | 33.619 | 30.024 | 33.948 |
| bta-miR-150 | . | . | . | . | 32.450 | . |
| bta-miR-16b | 31.955 | 32.821 | 29.831 | 32.267 | 30.241 | 34.607 |
| bta-miR-17-3p | 34.842 | 34.971 | 39.653 | . | 34.447 | . |
| bta-miR-188 | 32.890 | 32.089 | 33.239 | 31.509 | 32.182 | 32.819 |
| bta-miR-17-5p | 34.471 | 32.681 | 34.297 | 33.044 | 32.943 | 33.882 |
| bta-miR-18a | . | 33.908 | 35.887 | . | 34.866 | 34.913 |
| bta-miR-181a | . | 34.270 | 32.836 | 34.912 | 32.866 | . |
| bta-miR-18b | . | . | . | . | . | . |
| bta-miR-181b | 33.868 | 31.974 | 32.039 | 32.412 | 32.300 | 34.420 |
| bta-miR-190a | . | . | 35.973 | . | . | . |
| bta-miR-181c | . | . | 32.891 | . | 34.001 | . |
| bta-miR-190b | 35.525 | . | . | . | 34.870 | . |
| bta-miR-181d | 33.034 | 30.958 | 32.761 | 31.900 | 31.901 | 33.402 |
| bta-miR-191 | 31.756 | 29.103 | 30.907 | 28.777 | 30.663 | 31.365 |
| bta-miR-182 | . | 35.822 | . | . | . | . |
| bta-miR-192 | 36.807 | 33.480 | 33.951 | 33.491 | 35.025 | 33.973 |
| bta-miR-183 | . | . | . | . | . | . |
| bta-miR-193a | . | 35.050 | . | . | . | 35.837 |
| bta-miR-184 | 35.467 | 32.229 | . | 32.637 | 33.984 | . |
| bta-miR-193a-3p | . | . | . | 35.861 | 35.579 | 34.773 |
| bta-miR-185 | . | . | . | . | 33.555 | . |
| bta-miR-193a-5p | 30.793 | 28.699 | 32.234 | 30.763 | 29.738 | 31.168 |
| bta-miR-186 | . | 39.685 | 33.886 | . | 33.531 | . |
| bta-miR-193b | 32.847 | 31.836 | 31.817 | 32.469 | 32.767 | 32.981 |
| bta-miR-187 | 32.455 | 30.668 | 30.326 | 30.753 | 30.913 | 31.409 |
| bta-miR-194 | 35.993 | . | 34.476 | . | 33.941 | . |
| bta-miR-195 | 33.123 | 31.588 | 31.564 | 32.513 | 30.224 | 35.083 |
| bta-miR-200c | 31.798 | 32.190 | 33.795 | 31.797 | 31.090 | 32.932 |
| bta-miR-196a | . | . | 33.999 | 36.654 | . | . |
| bta-miR-202 | . | . | . | 34.948 | . | . |
| bta-miR-196b | . | . | 33.868 | . | . | . |
| bta-miR-204 | 35.948 | 33.545 | 35.715 | . | 33.573 | . |
| bta-miR-197 | . | 30.807 | 34.912 | 32.402 | 32.745 | . |
| bta-miR-205 | 34.048 | 34.214 | 33.622 | 35.054 | 34.759 | 33.599 |
| bta-miR-199a-3p | . | 32.774 | 31.906 | . | 30.737 | . |
| bta-miR-206 | 32.934 | 31.780 | 29.310 | 32.836 | 30.689 | 35.163 |
| bta-miR-199a-5p | 34.744 | 33.317 | 33.167 | . | 32.607 | . |
| bta-miR-208a | 35.103 | 36.603 | . | . | . | . |
| bta-miR-199b | . | 34.549 | 32.910 | . | 33.546 | 34.760 |
| bta-miR-208b | . | . | 33.523 | . | 36.124 | . |
| bta-miR-199c | 32.684 | 31.939 | 31.059 | 32.580 | 29.677 | 33.972 |
| bta-miR-20a | 33.647 | 35.902 | 33.073 | 34.957 | 31.746 | 33.129 |
| bta-miR-19a | . | 35.907 | . | . | 34.548 | . |
| bta-miR-20b | 33.975 | 32.938 | 33.846 | . | 33.429 | . |
| bta-miR-19b | . | 35.012 | . | . | 33.928 | 34.542 |
| bta-miR-21-3p | 34.540 | 32.707 | . | 34.988 | 35.560 | 35.608 |
| bta-miR-200a | 33.916 | 36.139 | 33.798 | . | 32.863 | 33.984 |
| bta-miR-21-5p | . | . | . | . | . | 36.532 |
| bta-miR-200b | 29.869 | 30.353 | 31.851 | 31.379 | 29.835 | 31.333 |
| bta-miR-210 | 33.799 | 33.098 | 33.984 | 33.232 | 33.099 | 32.856 |
| bta-miR-211 | 35.820 | . | . | 34.449 | 33.955 | . |
| bta-miR-22-5p | 33.187 | 34.224 | 30.160 | 33.620 | 32.772 | 32.163 |
| bta-miR-212 | 28.074 | 27.476 | 31.761 | 31.302 | 30.859 | 29.730 |
| bta-miR-221 | 32.747 | 32.702 | 32.481 | 32.521 | . | 32.916 |
| bta-miR-214 | 33.856 | 33.604 | 32.934 | 32.272 | 33.040 | 33.056 |
| bta-miR-222 | 34.934 | 33.328 | 32.951 | 34.762 | 32.980 | 34.936 |
| bta-miR-215 | 38.749 | . | . | . | 35.740 | . |
| bta-miR-223 | . | . | 35.559 | 34.302 | 34.717 | . |
| bta-miR-216a | . | . | . | 35.944 | . | 34.393 |
| bta-miR-224 | 34.158 | . | . | . | . | . |
| bta-miR-216b | . | . | . | . | 34.597 | . |
| bta-miR-23a | 30.830 | 31.515 | 29.366 | 31.155 | 28.649 | 31.622 |
| bta-miR-217 | . | 41.657 | . | . | . | . |
| bta-miR-23b-3p | 33.888 | 34.274 | 32.213 | 34.968 | 31.775 | 33.882 |
| bta-miR-218 | 34.751 | . | . | . | . | . |
| bta-miR-23b-5p | . | . | . | . | . | . |
| bta-miR-219 | 31.040 | 29.592 | 31.841 | 30.692 | 30.658 | 29.326 |
| bta-miR-24 | . | . | . | . | . | . |
| bta-miR-219-3p | 36.101 | 36.430 | . | 35.075 | 35.103 | 36.966 |
| bta-miR-24-3p | 31.610 | 30.754 | 29.416 | 32.029 | 29.371 | 32.237 |
| bta-miR-219-5p | . | . | . | . | . | . |
| bta-miR-25 | 34.914 | 32.812 | 32.759 | 32.860 | 32.154 | 34.838 |
| bta-miR-22-3p | 2.953 | 2.836 | . | 3.157 | 3.057 | 2.795 |
| bta-miR-26a | 30.824 | 31.496 | 28.752 | 32.782 | 29.285 | 32.639 |
| bta-miR-26b | 35.908 | 32.536 | 30.760 | 34.317 | 31.277 | . |
| bta-miR-29d-3p | 38.858 | 33.223 | 30.653 | 34.496 | 31.874 | 34.745 |
| bta-miR-26c | . | . | . | . | . | . |
| bta-miR-29d-5p | 35.140 | 35.073 | 33.848 | 34.272 | 35.984 | 35.408 |
| bta-miR-27a-3p | 35.418 | 31.886 | 29.930 | 34.288 | 30.725 | 34.635 |
| bta-miR-29e | . | 36.627 | 34.884 | . | 35.210 | . |
| bta-miR-27a-5p | . | 36.884 | . | 36.307 | 29.751 | 18.623 |
| bta-miR-301a | . | 34.780 | 35.986 | . | 36.863 | . |
| bta-miR-27b | 32.827 | 34.875 | 30.230 | 35.042 | 29.823 | 32.999 |
| bta-miR-301b | 36.974 | . | . | . | 33.977 | . |
| bta-miR-28 | . | . | 42.951 | . | 33.314 | . |
| bta-miR-302a | . | . | . | . | . | . |
| bta-miR-296-3p | 32.824 | 34.090 | 35.343 | 29.873 | 36.886 | 33.805 |
| bta-miR-302b | . | . | . | . | . | . |
| bta-miR-296-5p | 33.004 | 33.887 | 30.810 | 35.788 | 33.895 | 29.245 |
| bta-miR-302c | . | 34.568 | . | . | . | . |
| bta-miR-299 | . | 34.876 | . | . | . | . |
| bta-miR-302d | . | . | 35.565 | 34.342 | . | . |
| bta-miR-29a | 31.895 | 31.901 | 29.748 | . | 29.802 | . |
| bta-miR-3064 | . | . | . | . | . | . |
| bta-miR-29b | 33.867 | 32.199 | 31.935 | . | 32.866 | 32.871 |
| bta-miR-30a-5p | 33.910 | 34.280 | 31.352 | . | 30.711 | 32.726 |
| bta-miR-29c | 31.180 | 32.611 | 29.835 | 32.525 | 29.549 | 32.944 |
| bta-miR-30b-3p | . | . | 33.730 | . | . | . |
| bta-miR-30b-5p | 32.908 | 32.023 | 33.376 | 34.550 | 33.032 | 35.838 |
| bta-miR-328 | 33.822 | 31.444 | 33.769 | 31.793 | 33.510 | 33.300 |
| bta-miR-30c | 32.713 | 31.817 | 31.660 | 32.292 | 30.693 | 33.882 |
| bta-miR-329a | . | . | . | . | . | . |
| bta-miR-30d | 34.718 | 32.972 | 32.245 | . | 31.745 | 33.841 |
| bta-miR-329b | . | . | . | . | . | . |
| bta-miR-30e-5p | 33.819 | . | 31.471 | . | 32.057 | 34.938 |
| bta-miR-330 | 36.480 | 33.307 | 33.875 | 34.821 | 33.270 | . |
| bta-miR-30f | 35.281 | 33.919 | 32.428 | 35.033 | 33.539 | 38.461 |
| bta-miR-331-3p | . | . | 35.431 | 35.944 | . | . |
| bta-miR-31 | 31.934 | 32.390 | 34.792 | 33.815 | 32.491 | 32.892 |
| bta-miR-331-5p | 31.856 | 30.429 | 32.749 | 29.923 | 31.866 | 31.833 |
| bta-miR-32 | . | . | . | . | . | . |
| bta-miR-335 | 36.598 | 34.518 | . | . | 34.897 | 36.140 |
| bta-miR-320a | 28.197 | 27.892 | 28.711 | 28.451 | 28.046 | 27.969 |
| bta-miR-338 | . | . | 34.898 | . | . | . |
| bta-miR-320b | 31.919 | 31.488 | 32.930 | 31.873 | 31.835 | 32.008 |
| bta-miR-339a | 34.793 | 31.654 | 33.064 | 32.935 | 32.712 | 32.923 |
| bta-miR-323 | 16.848 | 16.762 | 17.303 | 16.799 | 16.512 | 16.828 |
| bta-miR-339b | 34.947 | 31.369 | 32.897 | 31.847 | 34.136 | 33.063 |
| bta-miR-324 | 34.905 | 35.101 | 33.950 | 34.326 | 34.292 | 34.283 |
| bta-miR-33a | . | . | . | . | . | 36.438 |
| bta-miR-326 | . | . | 35.343 | . | 35.019 | 35.620 |
| bta-miR-33b | 33.988 | 32.893 | 33.818 | 34.372 | 33.277 | 33.896 |
| bta-miR-340 | . | . | . | . | 35.177 | . |
| bta-miR-365-3p | 33.521 | . | 30.054 | . | 30.629 | 34.709 |
| bta-miR-342 | . | 34.932 | . | 34.788 | 33.561 | . |
| bta-miR-365-5p | . | 33.948 | 33.518 | 33.770 | 35.516 | 33.418 |
| bta-miR-345-3p | 34.350 | 32.798 | 35.027 | 34.764 | 36.352 | 34.753 |
| bta-miR-367 | . | . | . | . | . | 38.216 |
| bta-miR-345-5p | 32.841 | 32.257 | 33.302 | 32.350 | 32.524 | 32.805 |
| bta-miR-369-3p | . | 36.111 | . | . | 35.568 | . |
| bta-miR-346 | 32.666 | 31.406 | 34.940 | 31.856 | 32.827 | 32.927 |
| bta-miR-369-5p | 35.281 | . | 36.386 | . | . | . |
| bta-miR-34a | 34.859 | 33.883 | 32.868 | 33.408 | . | 34.995 |
| bta-miR-370 | 34.869 | 33.718 | 36.698 | 33.924 | 33.923 | 34.900 |
| bta-miR-34b | . | 34.483 | . | . | 34.464 | 35.727 |
| bta-miR-371 | . | 33.512 | . | 32.978 | 34.934 | 36.988 |
| bta-miR-34c | 34.160 | 34.281 | . | . | . | 35.638 |
| bta-miR-374a | . | . | 36.131 | . | 36.524 | . |
| bta-miR-361 | . | 34.442 | . | 35.361 | 35.055 | . |
| bta-miR-374b | . | . | . | . | 33.887 | . |
| bta-miR-362-3p | . | . | 34.604 | . | . | . |
| bta-miR-375 | 31.811 | 31.920 | 32.692 | 30.759 | 32.262 | 31.858 |
| bta-miR-362-5p | . | 35.168 | 35.068 | . | 34.048 | 36.933 |
| bta-miR-376a | . | 35.566 | . | 36.689 | . | . |
| bta-miR-363 | 36.871 | 36.137 | 36.206 | 35.316 | . | . |
| bta-miR-376b | . | . | . | . | . | . |
| bta-miR-376c | . | . | . | . | . | . |
| bta-miR-382 | 29.562 | 29.022 | 29.850 | 28.833 | 29.821 | 29.711 |
| bta-miR-376d | . | . | . | . | . | . |
| bta-miR-383 | 34.266 | 32.393 | 36.044 | 31.800 | 33.273 | 32.876 |
| bta-miR-376e | . | . | . | . | . | . |
| bta-miR-409a | . | 35.121 | 36.454 | 36.521 | 34.420 | . |
| bta-miR-377 | . | . | . | . | . | . |
| bta-miR-409b | . | . | . | . | . | . |
| bta-miR-378 | 33.442 | 31.811 | 30.296 | 35.416 | 31.856 | 33.004 |
| bta-miR-410 | 34.435 | 32.662 | 35.648 | 32.919 | 35.599 | . |
| bta-miR-378b | 34.541 | 33.334 | 30.283 | 35.692 | 31.819 | 33.806 |
| bta-miR-411a | 31.906 | 31.829 | 33.098 | 31.534 | 32.568 | 32.664 |
| bta-miR-378c | . | 34.506 | 32.954 | 32.767 | 33.962 | 34.505 |
| bta-miR-411b | 34.464 | 33.528 | 36.051 | 34.764 | 33.462 | 34.129 |
| bta-miR-378d | 33.267 | 35.121 | 34.123 | 32.852 | 34.272 | 36.093 |
| bta-miR-411c-3p | . | 36.699 | 37.809 | 34.761 | . | . |
| bta-miR-379 | . | . | 35.306 | . | . | . |
| bta-miR-411c-5p | . | . | 34.925 | . | . | . |
| bta-miR-380-3p | . | 33.105 | . | 36.746 | . | . |
| bta-miR-412 | . | . | . | . | . | . |
| bta-miR-380-5p | 35.793 | 34.577 | 34.394 | 35.104 | 35.268 | . |
| bta-miR-421 | 30.127 | 26.887 | 29.802 | 29.099 | 29.894 | 30.235 |
| bta-miR-381 | . | . | . | 35.095 | 34.408 | . |
| bta-miR-423-3p | 34.969 | 32.258 | 33.384 | 32.315 | 34.893 | 34.702 |
| bta-miR-423-5p | 33.155 | 32.921 | 34.000 | 32.200 | 32.792 | 35.814 |
| bta-miR-449c | 35.472 | 34.984 | 36.190 | 36.139 | 36.098 | 34.476 |
| bta-miR-424-3p | . | 33.916 | 33.786 | 35.445 | . | . |
| bta-miR-449d | 33.340 | 32.066 | 33.649 | 32.562 | 34.877 | 32.918 |
| bta-miR-424-5p | 36.874 | . | 32.253 | 34.977 | 31.011 | . |
| bta-miR-450a | . | . | . | . | . | . |
| bta-miR-425-3p | 28.567 | 27.743 | 28.899 | 28.282 | 28.885 | 28.789 |
| bta-miR-450b | . | . | . | . | . | . |
| bta-miR-425-5p | . | . | 36.004 | 33.576 | 33.298 | 35.426 |
| bta-miR-451 | . | 34.968 | 32.164 | . | 31.193 | 34.683 |
| bta-miR-429 | 29.161 | 28.776 | 29.585 | 29.673 | 29.390 | 29.284 |
| bta-miR-452 | . | 36.963 | . | 35.044 | . | . |
| bta-miR-431 | . | 33.583 | . | 34.471 | . | 36.346 |
| bta-miR-4523 | 33.322 | 32.292 | 35.671 | 27.756 | 32.799 | 33.300 |
| bta-miR-432 | 36.329 | 34.144 | 35.529 | 34.465 | 35.368 | . |
| bta-miR-453 | 32.945 | 32.125 | 33.515 | 32.282 | 32.873 | 33.532 |
| bta-miR-433 | 30.055 | 30.088 | 29.724 | 29.246 | 29.897 | 29.961 |
| bta-miR-454 | . | . | . | . | . | . |
| bta-miR-448 | . | . | . | . | . | . |
| bta-miR-455-3p | . | . | . | . | . | . |
| bta-miR-449a | 34.028 | 35.205 | . | 34.985 | 35.106 | 33.783 |
| bta-miR-455-5p | . | . | . | . | . | . |
| bta-miR-449b | . | 33.885 | 35.032 | . | 35.625 | 34.301 |
| bta-miR-483 | . | 32.882 | 33.895 | 34.532 | 32.754 | . |
| bta-miR-484 | 35.226 | 33.164 | 33.333 | 33.892 | 36.448 | . |
| bta-miR-496 | 36.012 | 35.934 | . | 36.090 | . | . |
| bta-miR-485 | 36.627 | 35.341 | . | 34.629 | 33.421 | 35.085 |
| bta-miR-497 | . | 36.027 | 34.555 | . | 34.052 | . |
| bta-miR-486 | 29.766 | 29.778 | 29.652 | 30.954 | 29.541 | 29.374 |
| bta-miR-499 | . | . | 33.184 | . | 34.015 | . |
| bta-miR-487a | . | . | . | . | . | . |
| bta-miR-500 | 26.271 | 23.834 | 26.762 | 24.358 | 25.554 | 25.895 |
| bta-miR-487b | . | 35.280 | . | . | . | . |
| bta-miR-502a | . | . | . | . | . | . |
| bta-miR-488 | . | . | . | . | . | . |
| bta-miR-502b | . | 35.511 | . | 34.478 | . | . |
| bta-miR-489 | 31.722 | 31.787 | 31.635 | 30.677 | 31.845 | 31.794 |
| bta-miR-503-3p | 31.973 | 31.630 | 31.237 | 31.932 | 32.040 | 31.584 |
| bta-miR-490 | . | 33.587 | 40.638 | 35.298 | 34.970 | . |
| bta-miR-503-5p | . | 35.633 | . | 35.169 | 6.299 | 36.056 |
| bta-miR-491 | 35.752 | 35.671 | 35.166 | . | 33.903 | 35.036 |
| bta-miR-504 | . | 36.823 | . | . | . | . |
| bta-miR-493 | 31.888 | 30.456 | 32.766 | 31.142 | 30.647 | 31.088 |
| bta-miR-505 | 31.819 | 29.806 | 31.414 | 31.269 | 30.460 | 31.152 |
| bta-miR-494 | 21.884 | 22.199 | 22.810 | 22.776 | 22.804 | 21.759 |
| bta-miR-532 | 35.667 | 32.596 | . | 32.942 | 33.862 | 34.546 |
| bta-miR-495 | . | 36.432 | . | 35.743 | . | . |
| bta-miR-539 | . | 34.921 | 34.082 | . | . | . |
| bta-miR-541 | 29.918 | 29.711 | 30.472 | 29.272 | 29.821 | 30.010 |
| bta-miR-582 | . | . | . | . | . | . |
| bta-miR-542-5p | . | 36.564 | . | . | 36.552 | . |
| bta-miR-584 | 29.708 | 28.592 | 30.800 | 30.158 | 29.727 | 29.706 |
| bta-miR-543 | . | 36.330 | . | 33.399 | . | . |
| bta-miR-592 | 36.790 | 33.441 | 34.741 | 33.409 | 34.989 | 35.037 |
| bta-miR-544a | . | . | . | . | 35.267 | . |
| bta-miR-599 | . | . | . | . | . | . |
| bta-miR-544b | . | . | . | . | . | . |
| bta-miR-615 | 9.446 | 9.728 | 9.552 | 9.426 | 9.499 | 9.341 |
| bta-miR-545-3p | . | . | . | . | . | . |
| bta-miR-628 | 34.785 | . | . | . | . | . |
| bta-miR-545-5p | . | . | . | . | . | . |
| bta-miR-631 | 19.250 | 19.267 | 19.343 | 19.132 | 18.990 | 19.307 |
| bta-miR-551a | . | . | . | 33.878 | . | . |
| bta-miR-652 | 34.443 | 32.294 | 32.904 | 33.099 | 33.475 | 34.983 |
| bta-miR-551b | . | 35.745 | . | 35.642 | 36.023 | . |
| bta-miR-653 | . | . | . | . | . | . |
| bta-miR-562 | . | . | . | . | . | . |
| bta-miR-654 | . | . | 33.918 | . | . | . |
| bta-miR-568 | . | . | . | . | . | . |
| bta-miR-655 | . | . | . | 36.124 | . | 36.607 |
| bta-miR-574 | 26.798 | 27.533 | 26.760 | 28.002 | 27.831 | 26.869 |
| bta-miR-656 | 31.849 | 30.794 | 31.229 | 30.739 | 30.985 | 31.303 |
| bta-miR-658 | 34.876 | 35.812 | 35.741 | 35.997 | 36.413 | 35.045 |
| bta-miR-758 | . | 43.649 | . | . | . | . |
| bta-miR-660 | . | 34.133 | 35.626 | 34.223 | 33.329 | 36.997 |
| bta-miR-759 | 36.797 | . | . | 36.789 | . | . |
| bta-miR-664a | 32.847 | 32.915 | 34.084 | 31.817 | 31.011 | 33.120 |
| bta-miR-760-3p | 35.011 | 34.021 | 35.274 | 33.499 | 34.068 | 33.955 |
| bta-miR-664b | . | . | 33.772 | 33.874 | 32.248 | 36.437 |
| bta-miR-760-5p | 32.821 | 32.528 | 30.697 | 32.043 | 31.224 | 32.865 |
| bta-miR-665 | 32.643 | 29.298 | 31.813 | 29.714 | 30.658 | 30.612 |
| bta-miR-761 | . | 33.070 | 34.860 | 32.945 | . | . |
| bta-miR-669 | 31.842 | 31.358 | 32.745 | 32.465 | 31.840 | 32.053 |
| bta-miR-763 | 33.214 | 33.217 | 33.503 | 31.125 | 34.180 | 33.891 |
| bta-miR-670 | . | . | . | 35.026 | . | . |
| bta-miR-764 | 35.596 | 35.453 | . | . | . | 36.771 |
| bta-miR-671 | . | . | . | 35.570 | . | 36.555 |
| bta-miR-767 | 30.807 | 30.930 | 30.775 | 30.889 | 30.876 | 30.987 |
| bta-miR-677 | 36.989 | 33.108 | 31.884 | 35.316 | 32.351 | 33.777 |
| bta-miR-769 | . | . | . | 34.560 | . | . |
| bta-miR-7 | . | 34.924 | 33.820 | . | 36.050 | . |
| bta-miR-873 | . | 34.989 | 36.058 | . | . | 33.963 |
| bta-miR-708 | 35.873 | 33.826 | 32.958 | . | 33.890 | . |
| bta-miR-874 | 31.882 | 29.699 | 30.890 | 30.752 | 31.588 | 30.688 |
| bta-miR-744 | 36.682 | 36.467 | . | 34.828 | . | . |
| bta-miR-875 | . | 36.951 | . | 36.638 | . | . |
| bta-miR-876 | . | 34.375 | 36.874 | . | 35.304 | . |
| bta-miR-98 | . | . | 34.574 | . | . | . |
| bta-miR-877 | 31.219 | 29.820 | 31.445 | 30.105 | 30.539 | 30.365 |
| bta-miR-99a-3p | . | . | 36.088 | . | 34.573 | 33.827 |
| bta-miR-885 | 34.301 | 28.281 | 34.549 | 29.973 | 31.931 | 32.899 |
| bta-miR-99a-5p | 32.367 | 33.877 | 30.954 | 33.754 | 30.249 | . |
| bta-miR-9-3p | . | . | . | . | . | . |
| bta-miR-99b | 23.789 | 23.279 | 23.829 | 23.520 | 23.671 | 23.838 |
| bta-miR-9-5p | . | 34.142 | 34.957 | 36.065 | 35.966 | . |
| bta-miR-1179 | . | 35.305 | . | . | . | . |
| bta-miR-92a | 30.715 | 29.097 | 30.769 | 29.679 | 30.506 | 31.774 |
| bta-miR-1185 | . | . | . | . | . | . |
| bta-miR-92b | 28.976 | 28.137 | 27.970 | 28.434 | 28.714 | 28.738 |
| bta-miR-1193 | . | . | . | . | . | . |
| bta-miR-93 | . | . | . | 32.583 | 32.271 | 33.908 |
| bta-miR-1197 | . | 36.989 | . | . | . | . |
| bta-miR-935 | 33.970 | 31.947 | 32.998 | 32.728 | 32.963 | 34.294 |
| bta-miR-122 | 35.518 | 35.547 | . | 34.556 | . | . |
| bta-miR-940 | 27.816 | 27.350 | 27.119 | 27.962 | 27.811 | 25.940 |
| bta-miR-1224 | 27.264 | 27.739 | 28.098 | 27.191 | 26.566 | 27.746 |
| bta-miR-95 | . | . | . | . | . | . |
| bta-miR-1225-3p | 29.641 | 29.131 | 28.887 | 28.842 | 29.791 | 28.127 |
| bta-miR-96 | . | . | . | . | . | . |
| bta-miR-1246 | 22.725 | 24.781 | 23.757 | 24.731 | 24.603 | 23.892 |
| bta-miR-1247-3p | 31.768 | 30.837 | 32.030 | 31.129 | . | 31.663 |
| bta-miR-1296 | 34.580 | 33.897 | 33.524 | 32.333 | . | 34.034 |
| bta-miR-1247-5p | 29.776 | 29.676 | 30.572 | 28.070 | 30.475 | 29.924 |
| bta-miR-1298 | . | . | 33.697 | . | . | . |
| bta-miR-1248 | 34.766 | 33.957 | 30.762 | 33.825 | 34.528 | 36.635 |
| bta-miR-1301 | 35.652 | 33.773 | . | 33.949 | 36.676 | 34.968 |
| bta-miR-1249 | . | 34.615 | 34.871 | . | 33.913 | 33.373 |
| bta-miR-1306 | 34.969 | 33.665 | 34.740 | 35.254 | 33.991 | 36.515 |
| bta-miR-1260b | 26.184 | 25.703 | 22.348 | 26.404 | 23.837 | 26.254 |
| bta-miR-1307 | 27.374 | 27.434 | 28.195 | 27.826 | 28.665 | 27.632 |
| bta-miR-1271 | . | . | . | . | . | . |
| bta-miR-1343-3p | 31.899 | 30.196 | 32.034 | 30.578 | 30.744 | 31.995 |
| bta-miR-1277 | . | . | . | . | . | . |
| bta-miR-1343-5p | 28.548 | 28.941 | 28.706 | 28.789 | 28.480 | 28.654 |
| bta-miR-1281 | 30.284 | 30.658 | 29.368 | 31.017 | 30.493 | 28.494 |
| bta-miR-1388-3p | 32.077 | 30.502 | 31.580 | 30.699 | 32.184 | 33.441 |
| bta-miR-1282 | 33.850 | . | 32.655 | 35.668 | 36.681 | 32.918 |
| RNT43 snoRNA | 31.708 | 29.973 | 30.693 | 32.919 | 30.410 | 31.315 |
| bta-miR-1284 | . | . | . | . | . | . |
| Hm/Ms/Rt T1 snRNA | 20.780 | 22.578 | 20.722 | 20.857 | 21.073 | 22.287 |
| bta-miR-1287 | 33.305 | 30.752 | 31.736 | 31.880 | 32.466 | 32.866 |
| bta-miR-99b | 23.250 | 23.681 | 23.591 | 23.228 | 23.400 | 23.637 |
| bta-miR-1291 | 35.168 | 32.169 | . | 33.554 | 34.192 | 35.562 |
| Negative control | . | . | . | . | . | . |
| ^1^Body energy reserve: MBER: Cows with moderated body energy reserve; HBER: Cows with high body energy reserve. | | | | | | |
