# Supplementary material for: The bovine oviductal environment and composition are negatively affected by elevated body energy reserves
Source: PLoS One. 2025 Jun 23;20(6):e0326138. doi: 10.1371/journal.pone.0326138 (PMC12184905; doi:10.1371/journal.pone.0326138)
Supplement: S4 Table — (DOCX) [file pone.0326138.s007.docx]

| **Supplementary Table 4.** Normalized data of the 82 miRNAs commonly detected in ampullary extracellular vesicles (AMP-EVs) of cows with different body energy reserve. | | | | | | | |
| --- | --- | --- | --- | --- | --- | --- | --- |
| **miRNA** | **Body energy reserve^1^** | | | | | | **P – Value^2^** |
|  | **MBER** | | | **HBER** | | |  |
|  | **1** | **2** | **3** | **1** | **2** | **3** |  |
| bta-let-7c | 11.0621 | 12.932 | 9.18838 | 8.79235 | 11.4008 | 11.2821 | 0.7002 |
| bta-let-7e | 11.6012 | 10.5522 | 11.415 | 9.37628 | 7.50493 | 9.45158 | **0.0278** |
| bta-miR-126-3p | 9.08123 | 8.88399 | 11.6352 | 8.81788 | 5.38234 | 7.70872 | 0.1294 |
| bta-miR-126-5p | 12.2904 | 7.6655 | 10.0312 | 12.7715 | 5.33977 | 6.77431 | 0.5544 |
| bta-miR-127 | 10.5917 | 10.1137 | 10.1161 | 10.8689 | 7.61446 | 10.2515 | 0.5291 |
| bta-miR-129-5p | 10.8675 | 10.954 | 10.5817 | 9.87842 | 9.57595 | 11.3171 | 0.3777 |
| bta-miR-130a | 10.1439 | 10.8825 | 10.1123 | 11.0089 | 9.68973 | 9.49678 | 0.5901 |
| bta-miR-130b | 8.83089 | 10.6688 | 9.81708 | 9.73361 | 8.17945 | 8.67004 | 0.2639 |
| bta-miR-132 | 5.9878 | 6.47301 | 6.18405 | 3.99811 | 4.09169 | 4.97969 | **0.0056** |
| bta-miR-143 | 7.96597 | 10.7928 | 7.27338 | 8.011 | 6.48937 | 7.2275 | 0.2847 |
| bta-miR-154b | 10.2338 | 5.58387 | 6.23785 | 8.81732 | 2.84346 | 5.15574 | 0.4840 |
| bta-miR-149-3p | 6.66376 | 7.44094 | 7.0742 | 8.38257 | 5.39473 | 7.66739 | 0.9286 |
| bta-miR-188 | 11.9648 | 12.0152 | 10.4921 | 9.88893 | 9.18494 | 10.1757 | **0.0398** |
| bta-miR-191 | 11.6329 | 8.50585 | 8.35005 | 7.16905 | 5.87247 | 6.86252 | 0.0658 |
| bta-miR-192 | 12.1762 | 11.0094 | 11.2977 | 14.512 | 9.72696 | 9.77262 | 0.9276 |
| bta-miR-187 | 10.9184 | 9.46205 | 8.07356 | 9.43272 | 6.42917 | 9.13785 | 0.4126 |
| bta-miR-196a | 11.6335 | 11.0261 | 8.91992 | 9.41997 | 5.806 | 7.12542 | 0.0831 |
| bta-miR-196b | 11.7392 | 10.5872 | 10.4118 | 10.896 | 8.51673 | 10.8669 | 0.4100 |
| bta-miR-197 | 8.83907 | 7.59514 | 7.59913 | 9.92843 | 4.5243 | 7.00817 | 0.6237 |
| bta-miR-200b | 8.49529 | 8.48439 | 6.38252 | 5.25968 | 7.79942 | 7.2493 | 0.3845 |
| bta-miR-222 | 10.5093 | 9.58057 | 10.2765 | 8.86577 | 7.07774 | 9.12184 | 0.0653 |
| bta-miR-23a | 11.6449 | 9.15274 | 7.29485 | 7.12093 | 9.01353 | 8.99435 | 0.5215 |
| bta-miR-219 | 12.0973 | 8.78258 | 8.01917 | 8.76235 | 5.4264 | 6.80554 | 0.1712 |
| bta-miR-22-3p | -20.905 | -20.406 | -20.745 | -21.206 | -20.544 | -21.341 | 0.2953 |
| bta-miR-27a-5p | -15.129 | -14.456 | -14.715 | -15.643 | -14.733 | -15.246 | 0.2505 |
| bta-miR-296-3p | 9.95352 | 8.6679 | 6.89117 | 14.1605 | 7.41522 | 11.519 | 0.3057 |
| bta-miR-29a | 10.7105 | 11.1072 | 11.3269 | 6.66653 | 11.7053 | 10.057 | 0.3521 |
| bta-miR-328 | 7.14054 | 6.11301 | 7.0974 | 11.0058 | 6.03677 | 7.88802 | 0.3630 |
| bta-miR-30e-5p | 10.4556 | 13.3034 | 10.3432 | 14.0121 | 8.68574 | 9.42369 | 0.7492 |
| bta-miR-331-5p | 10.6358 | 8.01999 | 9.67472 | 11.6381 | 5.84218 | 9.12341 | 0.7705 |
| bta-miR-320a | 6.92081 | 7.02597 | 7.28813 | 5.15997 | 7.48946 | 8.44566 | 0.9644 |
| bta-miR-320b | 10.2005 | 9.72285 | 9.55215 | 9.66412 | 7.03392 | 8.19534 | 0.1237 |
| bta-miR-323 | -4.5472 | -4.4872 | -4.1813 | -5.0323 | -4.4111 | -4.7305 | 0.2067 |
| bta-miR-345-5p | 10.3723 | 10.3695 | 9.43375 | 10.1764 | 7.53952 | 8.88375 | 0.2210 |
| bta-miR-370 | 11.6017 | 8.93063 | 11.1389 | 10.7995 | 8.90479 | 11.2795 | 0.8448 |
| bta-miR-375 | 10.3824 | 10.314 | 8.42493 | 8.46894 | 4.58295 | 10.4853 | 0.3706 |
| bta-miR-382 | 9.90872 | 7.49054 | 6.89775 | 9.78149 | 4.95354 | 7.71239 | 0.7314 |
| bta-miR-411a | 9.59504 | 8.52523 | 7.12279 | 9.62146 | 4.94539 | 7.16202 | 0.4862 |
| bta-miR-411b | 10.4723 | 9.78499 | 8.03618 | 9.69325 | 7.16376 | 10.6116 | 0.8380 |
| bta-miR-378d | 10.2675 | 9.32972 | 8.80507 | 10.8991 | 6.51737 | 7.83601 | 0.4852 |
| bta-miR-380-5p | 8.08176 | 8.71244 | 6.18232 | 9.8324 | 4.70248 | 7.0141 | 0.7894 |
| bta-miR-421 | 12.7602 | 8.77852 | 8.64749 | 8.64186 | 5.36335 | 8.02264 | 0.1814 |
| bta-miR-425-3p | 9.06518 | 8.70843 | 7.2035 | 6.73594 | 5.66731 | 7.48394 | 0.0943 |
| bta-miR-429 | 7.00543 | 8.24254 | 6.44189 | 7.60376 | 5.65993 | 7.04501 | 0.5892 |
| bta-miR-432 | 10.1271 | 7.69254 | 7.07727 | 12.4105 | 4.90779 | 6.52308 | 0.8933 |
| bta-miR-453 | 11.6197 | 11.8591 | 11.9636 | 10.6526 | 9.59657 | 12.325 | 0.2985 |
| bta-miR-433 | 9.5037 | 5.99969 | 4.70409 | 7.13223 | 3.27165 | 6.11463 | 0.5408 |
| bta-miR-486 | 12.128 | 10.8021 | 12.1915 | 7.90527 | 8.13105 | 10.5804 | **0.0430** |
| bta-miR-500 | 10.2064 | 13.6221 | 10.4891 | 8.24091 | 12.2639 | 11.8563 | 0.7181 |
| bta-miR-489 | 10.1489 | 10.0588 | 9.76537 | 9.87999 | 9.7588 | 9.64645 | 0.1622 |
| bta-miR-503-3p | 10.9951 | 8.57308 | 7.58061 | 9.29317 | 5.50423 | 7.88695 | 0.3774 |
| bta-miR-494 | 5.24436 | 4.0354 | 4.27906 | 5.43921 | 5.71526 | 6.34817 | **0.0451** |
| bta-miR-541 | 9.46329 | 8.09198 | 7.58742 | 7.1116 | 5.56874 | 8.02098 | 0.1787 |
| bta-miR-584 | 4.97765 | 5.7377 | 4.24138 | 1.77982 | 6.16257 | 4.91763 | 0.6377 |
| bta-miR-615 | -11.363 | -10.83 | -11.118 | -11.82 | -10.763 | -11.203 | 0.6676 |
| bta-miR-631 | -3.4143 | -3.3663 | -3.0837 | -3.4765 | -3.474 | -3.5148 | 0.1263 |
| bta-miR-574 | 9.19042 | 7.5736 | 5.80692 | 3.94818 | 7.15248 | 8.26524 | 0.5460 |
| bta-miR-656 | 12.1634 | 8.74656 | 7.31314 | 10.2865 | 6.05749 | 9.0827 | 0.6513 |
| bta-miR-664a | 10.1708 | 11.4007 | 10.855 | 8.94062 | 9.17005 | 9.90061 | **0.0326** |
| bta-miR-760-3p | 10.5051 | 11.8708 | 9.84443 | 7.77466 | 9.2257 | 10.1824 | 0.1419 |
| bta-miR-760-5p | 10.1365 | 7.72575 | 6.24114 | 9.49587 | 4.79339 | 7.14682 | 0.6417 |
| bta-miR-767 | 10.2007 | 10.0826 | 8.55982 | 9.98548 | 9.29688 | 10.3648 | 0.6850 |
| bta-miR-708 | 10.8677 | 8.89958 | 8.29393 | 12.1322 | 7.43417 | 9.16491 | 0.8942 |
| bta-miR-877 | 8.17861 | 7.9789 | 7.169 | 6.82851 | 7.29244 | 8.14586 | 0.5142 |
| bta-miR-9-5p | 10.7526 | 10.851 | 9.06167 | 11.109 | 8.64983 | 10.5827 | 0.9147 |
| bta-miR-92b | 8.04497 | 6.2034 | 5.6455 | 6.82875 | 5.22439 | 6.35031 | 0.5973 |
| bta-miR-93 | 11.0407 | 10.7207 | 9.35035 | 8.65664 | 11.2708 | 11.0708 | 0.9713 |
| bta-miR-940 | 8.28086 | 7.61757 | 7.96802 | 5.58729 | 8.606 | 9.15872 | 0.8864 |
| bta-miR-1224 | 1.17228 | 2.62523 | 4.39874 | 5.94262 | 6.08771 | 6.91198 | **0.0217** |
| bta-miR-1225-3p | 9.28329 | 9.33284 | 8.33943 | 7.88148 | 7.82493 | 9.2532 | 0.3064 |
| bta-miR-1246 | -0.0058 | 1.75044 | 3.09178 | 1.67041 | 3.8758 | 5.07595 | 0.2238 |
| bta-miR-1247-3p | 9.01418 | 8.0301 | 5.24986 | 5.80438 | 4.92255 | 7.12389 | 0.3169 |
| bta-miR-1247-5p | 9.20904 | 9.17074 | 8.29065 | 7.97692 | 9.67395 | 11.3045 | 0.4913 |
| bta-miR-1260b | 3.67273 | 2.66492 | 0.30634 | 2.83056 | 3.98884 | 3.97571 | 0.2651 |
| bta-miR-1307 | 11.0942 | 9.24795 | 8.15813 | 8.57067 | 7.93526 | 10.1929 | 0.6107 |
| bta-miR-1343-3p | 8.93341 | 8.39081 | 6.01836 | 8.64445 | 7.14289 | 8.71285 | 0.7273 |
| bta-miR-1343-5p | 8.01281 | 8.23736 | 7.77357 | 6.60803 | 7.57402 | 10.2005 | 0.9173 |
| bta-miR-1281 | 7.09441 | 8.52587 | 5.89419 | 6.41905 | 5.0412 | 7.70427 | 0.5090 |
| bta-miR-1388-3p | 8.66794 | 10.7026 | 8.05964 | 10.0327 | 8.15634 | 10.2473 | 0.7630 |
| Hm/Ms/Rt T1 snRNA | -1.6069 | 0.02083 | 1.32196 | 0.9367 | 0.86333 | 4.06024 | 0.2056 |
| bta-miR-1287 | 11.666 | 10.4819 | 9.59731 | 9.37915 | 9.72279 | 12.8647 | 0.9561 |
| bta-miR-99b | 0.01028 | -0.0906 | -0.2231 | -0.1005 | -0.0878 | 0.01659 | 0.5998 |
| ^1^Body energy reserve: MBER: Cows with moderated body energy reserve; HBER: Cows with high body energy reserve; ^2^P-value: P value between animals with different body energy reserve. | | | | | | | |
