# Supplementary material for: The bovine oviductal environment and composition are negatively affected by elevated body energy reserves
Source: PLoS One. 2025 Jun 23;20(6):e0326138. doi: 10.1371/journal.pone.0326138 (PMC12184905; doi:10.1371/journal.pone.0326138)
Supplement: S5 Table — (DOCX) [file pone.0326138.s008.docx]

| **Supplementary Table 5.** Normalized data of the 150 miRNAs commonly detected in isthmic extracellular vesicles (IST-EVs) of cows with different body energy reserve. | | | | | | | |
| --- | --- | --- | --- | --- | --- | --- | --- |
| **miRNA** | **Body energy reserve^1^** | | | | | | **P-value^2^** |
|  | **MBER** | | | **HBER** | | |  |
|  | **1** | **2** | **3** | **1** | **2** | **3** |  |
| bta-miR-103 | 11.17591 | 10.89641 | 8.865318 | 13.4834 | 8.81394 | 10.39238 | 0.7259 |
| bta-let-7a-5p | 6.209712 | 7.177778 | 5.721577 | 6.90333 | 6.603207 | 7.916258 | 0.2570 |
| bta-let-7b | 7.182044 | 7.688715 | 6.190813 | 6.448496 | 6.175463 | 9.256462 | 0.8127 |
| bta-miR-106b | 9.37997 | 8.004498 | 9.73423 | 8.414736 | 9.210135 | 9.584236 | 0.9642 |
| bta-let-7e | 5.67361 | 6.723055 | 5.887817 | 6.636835 | 8.33988 | 7.990408 | 0.0627 |
| bta-let-7f | 7.406226 | 8.657598 | 7.107239 | 10.0822 | 7.72377 | 10.45418 | 0.1578 |
| bta-miR-10a | 11.04091 | 11.4319 | 7.715961 | 9.525482 | 8.357744 | 10.07971 | 0.5942 |
| bta-miR-1 | 7.861703 | 6.932756 | 2.296492 | 9.971348 | 4.871855 | 6.689949 | 0.5511 |
| bta-miR-124a | 9.614612 | 8.779001 | 9.338577 | 9.444054 | 9.89451 | 8.506268 | 0.9411 |
| bta-miR-100 | 7.213111 | 10.07969 | 7.375648 | 8.500069 | 7.750546 | 11.20747 | 0.5435 |
| bta-miR-124b | 8.415971 | 8.609091 | 9.458015 | 10.46604 | 9.392008 | 8.792458 | 0.2843 |
| bta-miR-125b | 7.319982 | 7.231377 | 5.931747 | 8.473083 | 4.871719 | 8.952972 | 0.6803 |
| bta-miR-126-3p | 8.808276 | 7.196796 | 5.361818 | 8.157825 | 4.527639 | 8.55196 | 0.9801 |
| bta-miR-126-5p | 9.722304 | 8.566599 | 6.188192 | 8.831066 | 5.847662 | 9.191471 | 0.8982 |
| bta-miR-134 | 10.52569 | 10.02379 | 13.17724 | 9.39164 | 11.52422 | 9.718689 | 0.4324 |
| bta-miR-127 | 7.178924 | 6.510365 | 7.458509 | 7.879025 | 6.935253 | 7.039467 | 0.5970 |
| bta-miR-129 | 9.362922 | 8.113626 | 10.42503 | 8.093742 | 10.43033 | 8.120363 | 0.7030 |
| bta-miR-129-5p | 9.342723 | 7.871872 | 11.78217 | 7.420998 | 9.315248 | 9.138828 | 0.4652 |
| bta-miR-138 | 10.16926 | 10.40484 | 12.25554 | 9.539974 | 11.15303 | 10.19297 | 0.4681 |
| bta-miR-130a | 7.372909 | 7.594852 | 8.1308 | 7.496222 | 7.264242 | 6.974224 | 0.1687 |
| bta-miR-139 | 7.205222 | 5.238153 | 8.540672 | 6.442274 | 8.367054 | 7.298517 | 0.7525 |
| bta-miR-130b | 7.067014 | 7.057287 | 7.071411 | 7.199686 | 6.756367 | 6.945711 | 0.4882 |
| bta-miR-132 | 7.96256 | 5.611607 | 8.449974 | 5.792992 | 7.305504 | 7.767563 | 0.7341 |
| bta-miR-133a | 8.318871 | 7.998035 | 3.227861 | 10.84563 | 5.049467 | 7.009317 | 0.6609 |
| bta-miR-143 | 8.77576 | 7.267372 | 7.321341 | 8.245388 | 7.119818 | 8.74276 | 0.7372 |
| bta-miR-151-5p | 10.35062 | 10.37486 | 8.470007 | 10.4777 | 7.214531 | 12.04701 | 0.9129 |
| bta-miR-154a | 9.988315 | 11.37512 | 11.82846 | 11.8175 | 10.55095 | 12.56194 | 0.5123 |
| bta-miR-154b | 5.980039 | 6.74918 | 6.758263 | 6.357443 | 6.061993 | 6.649214 | 0.6745 |
| bta-miR-154c | 10.90113 | 11.78828 | 11.55069 | 11.66859 | 9.855604 | 9.652596 | 0.2149 |
| bta-miR-148a | 9.413035 | 8.817762 | 8.392921 | 10.3641 | 6.938593 | 11.23001 | 0.6604 |
| bta-miR-148b | 10.44492 | 10.41232 | 9.588634 | 9.470139 | 7.478998 | 10.61174 | 0.3719 |
| bta-miR-149-3p | 6.219905 | 6.900366 | 6.86586 | 6.449944 | 6.691326 | 5.987778 | 0.3986 |
| bta-miR-16a | 9.758548 | 10.25755 | 6.437122 | 10.24496 | 6.488596 | 10.21028 | 0.9292 |
| bta-miR-16b | 8.435746 | 9.341108 | 6.456987 | 8.893066 | 6.70562 | 10.86926 | 0.6398 |
| bta-miR-188 | 9.371188 | 8.609522 | 9.865204 | 8.135046 | 8.646627 | 9.08176 | 0.2211 |
| bta-miR-17-5p | 10.95165 | 9.201868 | 10.92313 | 9.670048 | 9.40737 | 10.14426 | 0.3733 |
| bta-miR-181b | 10.34857 | 8.494308 | 8.665272 | 9.037815 | 8.764345 | 10.68224 | 0.7188 |
| bta-miR-181d | 9.514861 | 7.478064 | 9.386669 | 8.526175 | 8.365341 | 9.664276 | 0.9432 |
| bta-miR-191 | 8.236646 | 5.623587 | 7.533197 | 5.403269 | 7.127773 | 7.627281 | 0.7101 |
| bta-miR-192 | 13.28819 | 10.00078 | 10.57664 | 10.11746 | 11.48944 | 10.23597 | 0.5745 |
| bta-miR-193a-5p | 7.273412 | 5.219772 | 8.860382 | 7.389532 | 6.202488 | 7.43029 | 0.9267 |
| bta-miR-193b | 9.327529 | 8.356787 | 8.443254 | 9.095001 | 9.231794 | 9.243614 | 0.2002 |
| bta-miR-187 | 8.936263 | 7.18799 | 6.952196 | 7.379562 | 7.377857 | 7.671806 | 0.7505 |
| bta-miR-195 | 9.603553 | 8.107912 | 8.190243 | 9.139324 | 6.689001 | 11.3458 | 0.7815 |
| bta-miR-200c | 8.278815 | 8.710485 | 10.4213 | 8.423227 | 7.554483 | 9.194416 | 0.4079 |
| bta-miR-205 | 10.5288 | 10.73453 | 10.24799 | 11.67985 | 11.22398 | 9.861271 | 0.5000 |
| bta-miR-206 | 9.414786 | 8.300842 | 5.936382 | 9.461899 | 7.153978 | 11.42537 | 0.4135 |
| bta-miR-199c | 9.164618 | 8.45962 | 7.685457 | 9.205608 | 6.141253 | 10.23488 | 0.9478 |
| bta-miR-20a | 10.12747 | 12.42266 | 9.699039 | 11.58327 | 8.210178 | 9.391457 | 0.4762 |
| bta-miR-200b | 6.349769 | 6.873535 | 8.477457 | 8.004877 | 6.299713 | 7.595516 | 0.9394 |
| bta-miR-210 | 10.27993 | 9.618109 | 10.60997 | 9.857819 | 9.563421 | 9.118778 | 0.1443 |
| bta-miR-22-5p | 9.667888 | 10.74456 | 6.786339 | 10.24586 | 9.236731 | 8.425941 | 0.8638 |
| bta-miR-212 | 4.554889 | 3.996458 | 8.387453 | 7.927616 | 7.323542 | 5.992317 | 0.3912 |
| bta-miR-214 | 10.33705 | 10.12446 | 9.560478 | 8.898308 | 9.504087 | 9.318687 | 0.0589 |
| bta-miR-222 | 11.41473 | 9.848307 | 9.576859 | 11.38837 | 9.444368 | 11.19868 | 0.6622 |
| bta-miR-23a | 7.311198 | 8.035294 | 5.992023 | 7.781424 | 5.113171 | 7.884476 | 0.8721 |
| bta-miR-23b-3p | 10.369 | 10.79425 | 8.838745 | 11.59378 | 8.239296 | 10.14441 | 0.9946 |
| bta-miR-219 | 7.520541 | 6.112766 | 8.466751 | 7.318523 | 7.122595 | 5.588818 | 0.4747 |
| bta-miR-24-3p | 8.090997 | 7.274107 | 6.042261 | 8.655083 | 5.835579 | 8.499947 | 0.6541 |
| bta-miR-25 | 11.39487 | 9.332655 | 9.385155 | 9.486008 | 8.61886 | 11.10056 | 0.7763 |
| bta-miR-26a | 7.305032 | 8.016808 | 5.378508 | 9.40815 | 5.749537 | 8.901428 | 0.4656 |
| bta-miR-29d-3p | 15.3386 | 9.74296 | 7.279274 | 11.12197 | 8.338264 | 11.00791 | 0.8169 |
| bta-miR-29d-5p | 11.62032 | 11.5936 | 10.4739 | 10.89832 | 12.44872 | 11.67089 | 0.4912 |
| bta-miR-27a-3p | 11.89868 | 8.406197 | 6.556074 | 10.91428 | 7.189367 | 10.89806 | 0.7389 |
| bta-miR-27b | 9.308311 | 11.3959 | 6.856426 | 11.66791 | 6.287188 | 9.26141 | 0.9578 |
| bta-miR-296-3p | 9.304938 | 10.61022 | 11.96944 | 6.49894 | 13.35006 | 10.06803 | 0.7728 |
| bta-miR-296-5p | 9.485057 | 10.40759 | 7.436438 | 12.4138 | 10.35925 | 5.507814 | 0.8936 |
| bta-miR-29c | 7.660571 | 9.130949 | 6.460747 | 9.151276 | 6.013288 | 9.206993 | 0.7896 |
| bta-miR-30b-5p | 9.38911 | 8.542952 | 10.00223 | 11.17567 | 9.496073 | 12.10077 | 0.1380 |
| bta-miR-328 | 10.30239 | 7.964647 | 10.39543 | 8.41931 | 9.974607 | 9.563034 | 0.8111 |
| bta-miR-30c | 9.194003 | 8.337814 | 8.286472 | 8.917786 | 7.15764 | 10.14469 | 0.8908 |
| bta-miR-30f | 11.76166 | 10.43936 | 9.05447 | 11.65874 | 10.00362 | 14.72308 | 0.3422 |
| bta-miR-31 | 8.414468 | 8.910322 | 11.41829 | 10.44111 | 8.955149 | 9.154339 | 0.9538 |
| bta-miR-331-5p | 8.336928 | 6.949476 | 9.375286 | 6.549242 | 8.330029 | 8.095434 | 0.5650 |
| bta-miR-320a | 4.677434 | 4.412218 | 5.337252 | 5.0774 | 4.510035 | 4.231899 | 0.6140 |
| bta-miR-320b | 8.399459 | 8.008684 | 9.556301 | 8.498648 | 8.29949 | 8.270191 | 0.5597 |
| bta-miR-339a | 11.27357 | 8.174273 | 9.689762 | 9.561447 | 9.176149 | 9.185978 | 0.6775 |
| bta-miR-323 | -6.67114 | -6.71724 | -6.07045 | -6.57503 | -7.02365 | -6.90895 | 0.2315 |
| bta-miR-339b | 11.42752 | 7.889271 | 9.523026 | 8.472776 | 10.60036 | 9.325298 | 0.9079 |
| bta-miR-324 | 11.38555 | 11.62117 | 10.5756 | 10.95225 | 10.75658 | 10.54525 | 0.2601 |
| bta-miR-33b | 10.46886 | 9.413832 | 10.44423 | 10.99823 | 9.74122 | 10.15875 | 0.7265 |
| bta-miR-345-3p | 10.83063 | 9.318048 | 11.65296 | 11.39053 | 12.81662 | 11.01578 | 0.2632 |
| bta-miR-345-5p | 9.322089 | 8.777731 | 9.927963 | 8.975727 | 8.988501 | 9.068054 | 0.3760 |
| bta-miR-346 | 9.147067 | 7.926579 | 11.56571 | 8.482477 | 9.291876 | 9.189407 | 0.6381 |
| bta-miR-370 | 11.35029 | 10.2386 | 13.32426 | 10.55026 | 10.38768 | 11.16211 | 0.3715 |
| bta-miR-375 | 8.292018 | 8.440569 | 9.318443 | 7.385071 | 8.726767 | 8.120335 | 0.2945 |
| bta-miR-382 | 6.042964 | 5.542351 | 6.476446 | 5.45953 | 6.28528 | 5.973738 | 0.7676 |
| bta-miR-383 | 10.74668 | 8.913145 | 12.66998 | 8.425671 | 9.737921 | 9.138218 | 0.2184 |
| bta-miR-378 | 9.923236 | 8.331881 | 6.922289 | 12.04178 | 8.320988 | 9.266216 | 0.3531 |
| bta-miR-378b | 11.02169 | 9.854086 | 6.908686 | 12.3183 | 8.283663 | 10.0681 | 0.5999 |
| bta-miR-411a | 8.387261 | 8.349604 | 9.72422 | 8.159906 | 9.032549 | 8.926533 | 0.8399 |
| bta-miR-411b | 10.94478 | 10.04873 | 12.67674 | 11.39021 | 9.926291 | 10.39131 | 0.5004 |
| bta-miR-378d | 9.748084 | 11.64137 | 10.74906 | 9.477818 | 10.73676 | 12.35579 | 0.8921 |
| bta-miR-421 | 6.608041 | 3.407516 | 6.427677 | 5.725368 | 6.358215 | 6.497095 | 0.5401 |
| bta-miR-423-3p | 11.44991 | 8.778803 | 10.01 | 8.941216 | 11.3575 | 10.9641 | 0.7668 |
| bta-miR-423-5p | 9.635451 | 9.441198 | 10.62646 | 8.82563 | 9.256472 | 12.07618 | 0.8954 |
| bta-miR-449c | 11.95242 | 11.50408 | 12.81578 | 12.76463 | 12.56254 | 10.73838 | 0.9313 |
| bta-miR-449d | 9.82054 | 8.586172 | 10.27478 | 9.188492 | 11.34187 | 9.180714 | 0.7159 |
| bta-miR-425-3p | 5.047448 | 4.263044 | 5.525396 | 4.908121 | 5.349876 | 5.052002 | 0.7063 |
| bta-miR-429 | 5.642312 | 5.296574 | 6.211414 | 6.298655 | 5.854845 | 5.5462 | 0.6234 |
| bta-miR-4523 | 9.802554 | 8.812407 | 12.29737 | 4.382282 | 9.263468 | 9.56263 | 0.2631 |
| bta-miR-453 | 9.426059 | 8.645651 | 10.14067 | 8.908218 | 9.33708 | 9.794998 | 0.9145 |
| bta-miR-433 | 6.535562 | 6.608486 | 6.350296 | 5.872278 | 6.361917 | 6.223902 | 0.1041 |
| bta-miR-486 | 6.246661 | 6.298117 | 6.278273 | 7.579851 | 6.005039 | 5.63677 | 0.8345 |
| bta-miR-500 | 2.751954 | 0.354319 | 3.38809 | 0.983736 | 2.018721 | 2.157902 | 0.6781 |
| bta-miR-489 | 8.202521 | 8.306927 | 8.261436 | 7.302591 | 8.309669 | 8.056166 | 0.2932 |
| bta-miR-503-3p | 8.45361 | 8.150631 | 7.863565 | 8.55769 | 8.504259 | 7.847012 | 0.6331 |
| bta-miR-493 | 8.368438 | 6.976126 | 9.392044 | 7.768055 | 7.111837 | 7.35008 | 0.3138 |
| bta-miR-505 | 8.300122 | 6.326246 | 8.039667 | 7.894651 | 6.924662 | 7.414911 | 0.8426 |
| bta-miR-494 | -1.63496 | -1.28054 | -0.56408 | -0.59792 | -0.73111 | -1.97832 | 0.9206 |
| bta-miR-541 | 6.39844 | 6.231714 | 7.097651 | 5.897614 | 6.285688 | 6.27231 | 0.2229 |
| bta-miR-584 | 6.188684 | 5.11237 | 7.426083 | 6.784405 | 6.191706 | 5.968166 | 0.9239 |
| bta-miR-592 | 13.27041 | 9.961813 | 11.36683 | 10.03513 | 11.45333 | 11.29977 | 0.5991 |
| bta-miR-615 | -14.0734 | -13.7512 | -13.8219 | -13.948 | -14.0369 | -14.3961 | 0.2195 |
| bta-miR-631 | -4.26953 | -4.2129 | -4.03086 | -4.24188 | -4.54529 | -4.43034 | 0.1086 |
| bta-miR-652 | 10.92366 | 8.814116 | 9.530449 | 9.725567 | 9.939753 | 11.24534 | 0.5217 |
| bta-miR-574 | 3.279292 | 4.053377 | 3.385927 | 4.628204 | 4.29557 | 3.131907 | 0.4350 |
| bta-miR-656 | 8.329532 | 7.31398 | 7.855247 | 7.364714 | 7.449041 | 7.565872 | 0.2804 |
| bta-miR-658 | 11.35638 | 12.33285 | 12.36686 | 12.62336 | 12.87776 | 11.30797 | 0.6917 |
| bta-miR-664a | 9.328307 | 9.435633 | 10.71014 | 8.443344 | 7.475161 | 9.382137 | 0.1205 |
| bta-miR-760-3p | 11.49194 | 10.5415 | 11.89981 | 10.12498 | 10.53274 | 10.2173 | 0.0726 |
| bta-miR-760-5p | 9.301696 | 9.048285 | 7.322767 | 8.669281 | 7.688808 | 9.12743 | 0.9379 |
| bta-miR-665 | 9.124274 | 5.818836 | 8.439383 | 6.340084 | 7.122225 | 6.874781 | 0.3815 |
| bta-miR-669 | 8.32293 | 7.877956 | 9.371353 | 9.091324 | 8.304583 | 8.315643 | 0.9323 |
| bta-miR-763 | 9.695251 | 9.737562 | 10.12927 | 7.75111 | 10.64444 | 10.15372 | 0.7279 |
| bta-miR-767 | 7.288161 | 7.450369 | 7.400957 | 7.51528 | 7.340191 | 7.24999 | 0.9073 |
| bta-miR-677 | 13.46955 | 9.627939 | 8.509909 | 11.94178 | 8.815424 | 10.03988 | 0.8852 |
| bta-miR-874 | 8.3628 | 6.219395 | 7.516171 | 7.378412 | 8.052797 | 6.950726 | 0.8992 |
| bta-miR-877 | 7.699403 | 6.340729 | 8.071043 | 6.731041 | 7.003788 | 6.628012 | 0.3394 |
| bta-miR-885 | 10.78184 | 4.801031 | 11.17554 | 6.598738 | 8.39591 | 9.161667 | 0.7132 |
| bta-miR-92a | 7.19538 | 5.61788 | 7.395399 | 6.304861 | 6.970467 | 8.036798 | 0.6517 |
| bta-miR-92b | 5.457097 | 4.656933 | 4.59609 | 5.059885 | 5.178087 | 5.000406 | 0.5666 |
| bta-miR-935 | 10.45087 | 8.46699 | 9.623718 | 9.354088 | 9.427416 | 10.55638 | 0.7218 |
| bta-miR-940 | 4.296811 | 3.870539 | 3.744774 | 4.588444 | 4.275368 | 2.202326 | 0.7318 |
| bta-miR-1224 | 3.74479 | 4.25971 | 4.724144 | 3.816919 | 3.030884 | 4.008503 | 0.2041 |
| bta-miR-1225-3p | 6.121905 | 5.651754 | 5.513281 | 5.468018 | 6.255581 | 4.38986 | 0.5311 |
| bta-miR-1246 | -0.79415 | 1.301109 | 0.382721 | 1.356659 | 1.067633 | 0.15473 | 0.4700 |
| bta-miR-1247-5p | 6.256555 | 6.196478 | 7.19758 | 4.696552 | 6.939753 | 6.186398 | 0.4534 |
| bta-miR-1248 | 11.24695 | 10.47711 | 7.387741 | 10.45074 | 10.99259 | 12.8976 | 0.2791 |
| bta-miR-1306 | 11.44998 | 10.18557 | 11.36619 | 11.87962 | 10.45579 | 12.77763 | 0.4232 |
| bta-miR-1260b | 2.665286 | 2.223625 | -1.02544 | 3.029955 | 0.301739 | 2.516743 | 0.6684 |
| bta-miR-1307 | 3.854907 | 3.954506 | 4.820864 | 4.451782 | 5.129292 | 3.894249 | 0.5818 |
| bta-miR-1343-3p | 8.379815 | 6.716177 | 8.659832 | 7.203905 | 7.20858 | 8.25737 | 0.6326 |
| bta-miR-1343-5p | 5.028543 | 5.461735 | 5.332357 | 5.41522 | 4.944231 | 4.916775 | 0.4276 |
| bta-miR-1281 | 6.764709 | 7.178242 | 5.994081 | 7.64258 | 6.95764 | 4.756683 | 0.8466 |
| bta-miR-1388-3p | 8.557364 | 7.022686 | 8.206028 | 7.325203 | 8.648565 | 9.703217 | 0.4899 |
| RNT43 snoRNA | 8.188893 | 6.493582 | 7.319403 | 9.545467 | 6.874199 | 7.577818 | 0.5171 |
| Hm/Ms/Rt T1 snRNA | -2.73956 | -0.90206 | -2.65185 | -2.51664 | -2.46216 | -1.4505 | 0.9509 |
| bta-miR-1287 | 9.786246 | 7.2726 | 8.362364 | 8.50585 | 8.930414 | 9.128262 | 0.6383 |
| bta-miR-99b | -0.26955 | 0.20104 | 0.216698 | -0.14635 | -0.13543 | -0.1005 | 0.3315 |
| ^1^Body energy reserve: MBER: Cows with moderated body energy reserve; HBER: Cows with high body energy reserve; ^2^P-value: P value between animals with different body energy reserve. | | | | | | | |
