# Supplementary material for: The bovine oviductal environment and composition are negatively affected by elevated body energy reserves
Source: PLoS One. 2025 Jun 23;20(6):e0326138. doi: 10.1371/journal.pone.0326138 (PMC12184905; doi:10.1371/journal.pone.0326138)
Supplement: S6 Table — (DOCX) [file pone.0326138.s009.docx]

| **Supplementary table 6.** Biological patwhays predicted as modulated by miRNAs up regulated in ampullary extracellular vesicles (AMP-EVs) in moderated body energy reserve (MBER) group. | | |
| --- | --- | --- |
| **Pathway** | **%^1^** | **BH^2^** |
| bta04360 Axon guidance | 27.53 | 0.0094 |
| bta04664 Fc epsilon RI signaling pathway | 37.14 | 0.0094 |
| bta04728 Dopaminergic synapse | 29.85 | 0.0094 |
| bta04928 Parathyroid hormone synthesis. secretion and action | 32.69 | 0.0094 |
| bta05200 Pathways in cancer | 21.77 | 0.0094 |
| bta05214 Glioma | 36.36 | 0.0094 |
| bta04014 Ras signaling pathway | 24.79 | 0.0121 |
| bta04015 Rap1 signaling pathway | 25.00 | 0.0121 |
| bta04024 cAMP signaling pathway | 24.89 | 0.0121 |
| bta04062 Chemokine signaling pathway | 26.60 | 0.0121 |
| bta04071 Sphingolipid signaling pathway | 29.17 | 0.0121 |
| bta04261 Adrenergic signaling in cardiomyocytes | 27.33 | 0.0121 |
| bta04371 Apelin signaling pathway | 27.86 | 0.0121 |
| bta05225 Hepatocellular carcinoma | 26.44 | 0.0121 |
| bta04012 ErbB signaling pathway | 32.14 | 0.0124 |
| bta04072 Phospholipase D signaling pathway | 26.97 | 0.0124 |
| bta05212 Pancreatic cancer | 32.89 | 0.0126 |
| bta05223 Non-small cell lung cancer | 34.33 | 0.0126 |
| bta04810 Regulation of actin cytoskeleton | 24.64 | 0.0149 |
| bta04660 T cell receptor signaling pathway | 28.97 | 0.017 |
| bta04933 AGE-RAGE signaling pathway in diabetic complications | 29.13 | 0.0175 |
| bta00564 Glycerophospholipid metabolism | 28.85 | 0.0185 |
| bta05160 Hepatitis C | 25.61 | 0.0185 |
| bta04144 Endocytosis | 23.27 | 0.0212 |
| bta04010 MAPK signaling pathway | 22.26 | 0.0232 |
| bta04730 Long-term depression | 33.33 | 0.0232 |
| bta04912 GnRH signaling pathway | 29.03 | 0.0232 |
| bta05205 Proteoglycans in cancer | 23.90 | 0.0232 |
| bta04919 Thyroid hormone signaling pathway | 27.12 | 0.0234 |
| bta04530 Tight junction | 24.29 | 0.0274 |
| bta05211 Renal cell carcinoma | 30.99 | 0.0274 |
| bta04150 mTOR signaling pathway | 24.84 | 0.0283 |
| bta04625 C-type lectin receptor signaling pathway | 27.36 | 0.0283 |
| bta05220 Chronic myeloid leukemia | 29.87 | 0.0283 |
| bta05218 Melanoma | 30.14 | 0.0307 |
| bta04350 TGF-beta signaling pathway | 27.96 | 0.0322 |
| bta01522 Endocrine resistance | 27.66 | 0.0343 |
| bta05210 Colorectal cancer | 28.09 | 0.0343 |
| bta05230 Central carbon metabolism in cancer | 30.30 | 0.0385 |
| bta04925 Aldosterone synthesis and secretion | 27.08 | 0.04 |
| bta05017 Spinocerebellar ataxia | 27.08 | 0.04 |
| bta01100 Metabolic pathways | 17.55 | 0.0412 |
| bta04370 VEGF signaling pathway | 31.03 | 0.0412 |
| bta04916 Melanogenesis | 26.47 | 0.0412 |
| bta04152 AMPK signaling pathway | 25.20 | 0.0421 |
| bta04666 Fc gamma R-mediated phagocytosis | 26.88 | 0.0434 |
| bta05215 Prostate cancer | 26.53 | 0.0434 |
| bta04725 Cholinergic synapse | 25.44 | 0.0454 |
| bta05231 Choline metabolism in cancer | 26.26 | 0.0462 |
| bta05163 Human cytomegalovirus infection | 21.63 | 0.047 |
| bta04921 Oxytocin signaling pathway | 23.68 | 0.0472 |
| bta05219 Bladder cancer | 33.33 | 0.0506 |
| bta04971 Gastric acid secretion | 27.63 | 0.0539 |
| bta04390 Hippo signaling pathway | 23.08 | 0.0561 |
| bta04670 Leukocyte transendothelial migration | 24.78 | 0.0561 |
| bta04724 Glutamatergic synapse | 24.78 | 0.0561 |
| bta04910 Insulin signaling pathway | 23.57 | 0.0561 |
| bta04922 Glucagon signaling pathway | 25.24 | 0.0561 |
| bta04934 Cushing syndrome | 23.08 | 0.0561 |
| bta04935 Growth hormone synthesis. secretion and action | 24.58 | 0.0561 |
| bta04926 Relaxin signaling pathway | 23.85 | 0.0585 |
| bta04962 Vasopressin-regulated water reabsorption | 30.61 | 0.0612 |
| bta04550 Signaling pathways regulating pluripotency of stem cells | 23.24 | 0.0623 |
| bta04720 Long-term potentiation | 27.54 | 0.0623 |
| bta05226 Gastric cancer | 22.88 | 0.0623 |
| bta01521 EGFR tyrosine kinase inhibitor resistance | 26.25 | 0.0672 |
| bta04722 Neurotrophin signaling pathway | 23.77 | 0.0672 |
| bta04972 Pancreatic secretion | 24.51 | 0.0741 |
| bta05032 Morphine addiction | 25.00 | 0.0767 |
| bta04920 Adipocytokine signaling pathway | 26.39 | 0.0786 |
| bta05133 Pertussis | 25.97 | 0.0786 |
| bta05202 Transcriptional misregulation in cancer | 21.47 | 0.0786 |
| bta04022 cGMP-PKG signaling pathway | 21.89 | 0.0787 |
| bta04068 FoxO signaling pathway | 22.90 | 0.0815 |
| bta04070 Phosphatidylinositol signaling system | 24.24 | 0.083 |
| bta04931 Insulin resistance | 23.64 | 0.0838 |
| bta05161 Hepatitis B | 21.64 | 0.0853 |
| bta04713 Circadian entrainment | 24.00 | 0.086 |
| bta04915 Estrogen signaling pathway | 22.46 | 0.086 |
| bta05031 Amphetamine addiction | 26.09 | 0.0891 |
| bta05224 Breast cancer | 22.00 | 0.0891 |
| bta04020 Calcium signaling pathway | 20.79 | 0.0946 |
| bta04750 Inflammatory mediator regulation of TRP channels | 23.30 | 0.106 |
| bta04662 B cell receptor signaling pathway | 24.14 | 0.1061 |
| bta00561 Glycerolipid metabolism | 25.37 | 0.1086 |
| bta04066 HIF-1 signaling pathway | 22.73 | 0.1086 |
| bta04218 Cellular senescence | 21.08 | 0.1086 |
| bta04611 Platelet activation | 22.31 | 0.1086 |
| bta05020 Prion diseases | 31.25 | 0.1086 |
| bta05142 Chagas disease (American trypanosomiasis) | 22.61 | 0.1086 |
| bta05167 Kaposi sarcoma-associated herpesvirus infection | 20.39 | 0.1086 |
| bta05414 Dilated cardiomyopathy (DCM) | 23.23 | 0.1086 |
| bta04726 Serotonergic synapse | 22.41 | 0.1111 |
| bta04270 Vascular smooth muscle contraction | 21.80 | 0.1123 |
| bta04310 Wnt signaling pathway | 20.99 | 0.115 |
| bta05221 Acute myeloid leukemia | 25.00 | 0.115 |
| bta05164 Influenza A | 20.44 | 0.1263 |
| bta00010 Glycolysis Gluconeogenesis | 25.00 | 0.1271 |
| bta04727 GABAergic synapse | 23.08 | 0.1275 |
| bta04140 Autophagy | 21.13 | 0.1307 |
| bta04215 Apoptosis | 29.41 | 0.1307 |
| bta04520 Adherens junction | 24.29 | 0.1307 |
| bta03320 PPAR signaling pathway | 23.46 | 0.131 |
| bta04929 GnRH secretion | 24.62 | 0.131 |
| bta05410 Hypertrophic cardiomyopathy (HCM) | 22.83 | 0.131 |
| bta01524 Platinum drug resistance | 23.08 | 0.1568 |
| bta04151 PI3K-Akt signaling pathway | 18.23 | 0.1568 |
| bta00562 Inositol phosphate metabolism | 23.29 | 0.1587 |
| bta01230 Biosynthesis of amino acids | 23.29 | 0.1587 |
| bta03030 DNA replication | 27.78 | 0.1587 |
| bta04211 Longevity regulating pathway | 22.22 | 0.1615 |
| bta04540 Gap junction | 22.22 | 0.1615 |
| bta01200 Carbon metabolism | 21.24 | 0.1653 |
| bta04911 Insulin secretion | 22.35 | 0.1661 |
| bta00071 Fatty acid degradation | 26.19 | 0.1676 |
| bta05213 Endometrial cancer | 23.73 | 0.1852 |
| bta00340 Histidine metabolism | 30.43 | 0.1887 |
| bta03430 Mismatch repair | 30.43 | 0.1887 |
| bta05418 Fluid shear stress and atherosclerosis | 20.00 | 0.1895 |
| bta04380 Osteoclast differentiation | 20.15 | 0.1944 |
| bta05014 Amyotrophic lateral sclerosis (ALS) | 23.33 | 0.1944 |
| bta00310 Lysine degradation | 22.73 | 0.1979 |
| bta04137 Mitophagy | 22.73 | 0.1979 |
| bta05132 Salmonella infection | 18.75 | 0.1979 |
| bta00410 beta-Alanine metabolism | 26.47 | 0.1996 |
| bta00592 alpha-Linolenic acid metabolism | 27.59 | 0.1996 |
| bta04668 TNF signaling pathway | 20.34 | 0.1996 |
| bta04924 Renin secretion | 22.22 | 0.1996 |
| bta04961 Endocrine and other factor-regulated calcium reabsorption | 24.00 | 0.1996 |
| bta03420 Nucleotide excision repair | 24.44 | 0.204 |
| bta04141 Protein processing in endoplasmic reticulum | 19.28 | 0.2046 |
| bta04510 Focal adhesion | 18.69 | 0.2219 |
| bta00510 N-Glycan biosynthesis | 23.08 | 0.2291 |
| bta00565 Ether lipid metabolism | 23.08 | 0.2291 |
| bta04923 Regulation of lipolysis in adipocytes | 22.41 | 0.2363 |
| bta00380 Tryptophan metabolism | 23.40 | 0.2364 |
| bta05162 Measles | 19.08 | 0.239 |
| bta04950 Maturity onset diabetes of the young | 26.92 | 0.243 |
| bta05412 Arrhythmogenic right ventricular cardiomyopathy (ARVC) | 21.05 | 0.2468 |
| bta00330 Arginine and proline metabolism | 22.92 | 0.2505 |
| bta04927 Cortisol synthesis and secretion | 21.54 | 0.2505 |
| bta05222 Small cell lung cancer | 20.21 | 0.2505 |
| bta05135 Yersinia infection | 19.23 | 0.2539 |
| bta04917 Prolactin signaling pathway | 20.48 | 0.2584 |
| bta00270 Cysteine and methionine metabolism | 22.45 | 0.2628 |
| bta04650 Natural killer cell mediated cytotoxicity | 19.08 | 0.2628 |
| bta04721 Synaptic vesicle cycle | 20.51 | 0.2676 |
| bta03410 Base excision repair | 24.24 | 0.2724 |
| bta04130 SNARE interactions in vesicular transport | 24.24 | 0.2724 |
| bta00230 Purine metabolism | 18.66 | 0.2921 |
| bta00514 Other types of O-glycan biosynthesis | 22.22 | 0.2921 |
| bta00515 Mannose type O-glycan biosynthesis | 26.09 | 0.2921 |
| bta00051 Fructose and mannose metabolism | 23.53 | 0.2932 |
| bta00280 Valine. leucine and isoleucine degradation | 21.57 | 0.2938 |
| bta04621 NOD-like receptor signaling pathway | 17.93 | 0.2953 |
| bta05170 Human immunodeficiency virus 1 infection | 17.52 | 0.2953 |
| bta05235 PD-L1 expression and PD-1 checkpoint pathway in cancer | 19.35 | 0.3039 |
| bta04914 Progesterone-mediated oocyte maturation | 19.32 | 0.3199 |
| bta00220 Arginine biosynthesis | 26.32 | 0.323 |
| bta00770 Pantothenate and CoA biosynthesis | 26.32 | 0.323 |
| bta00020 Citrate cycle (TCA cycle) | 23.33 | 0.3243 |
| bta03008 Ribosome biogenesis in eukaryotes | 19.28 | 0.3287 |
| bta04260 Cardiac muscle contraction | 19.10 | 0.3287 |
| bta00053 Ascorbate and aldarate metabolism | 24.00 | 0.3367 |
| bta00052 Galactose metabolism | 22.58 | 0.3428 |
| bta00100 Steroid biosynthesis | 25.00 | 0.3428 |
| bta00532 Glycosaminoglycan biosynthesis | 25.00 | 0.3428 |
| bta04064 NF-kappa B signaling pathway | 18.35 | 0.3428 |
| bta05166 Human T-cell leukemia virus 1 infection | 17.09 | 0.3428 |
| bta05169 Epstein-Barr virus infection | 17.11 | 0.3428 |
| bta03018 RNA degradation | 18.99 | 0.3514 |
| bta05100 Bacterial invasion of epithelial cells | 19.18 | 0.3514 |
| bta04620 Toll-like receptor signaling pathway | 18.18 | 0.3532 |
| bta04210 Apoptosis | 17.61 | 0.3635 |
| bta04213 Longevity regulating pathway | 19.35 | 0.3669 |
| bta04973 Carbohydrate digestion and absorption | 20.45 | 0.3669 |
| bta00900 Terpenoid backbone biosynthesis | 23.81 | 0.3679 |
| bta04136 Autophagy | 21.21 | 0.3824 |
| bta04216 Ferroptosis | 20.00 | 0.3824 |
| bta04340 Hedgehog signaling pathway | 19.61 | 0.3824 |
| bta04913 Ovarian steroidogenesis | 19.30 | 0.3824 |
| bta03015 mRNA surveillance pathway | 17.89 | 0.3939 |
| bta04061 Viral protein interaction with cytokine and cytokine receptor | 17.89 | 0.3939 |
| bta04514 Cell adhesion molecules (CAMs) | 17.09 | 0.3939 |
| bta04723 Retrograde endocannabinoid signaling | 17.11 | 0.3939 |
| bta04930 Type II diabetes mellitus | 19.57 | 0.3986 |
| bta05152 Tuberculosis | 16.67 | 0.4047 |
| bta04146 Peroxisome | 17.86 | 0.413 |
| bta05144 Malaria | 18.64 | 0.413 |
| bta04217 Necroptosis | 16.67 | 0.4215 |
| bta04640 Hematopoietic cell lineage | 17.27 | 0.4215 |
| bta05146 Amoebiasis | 17.09 | 0.4298 |
| bta04975 Fat digestion and absorption | 18.75 | 0.4344 |
| bta05030 Cocaine addiction | 18.75 | 0.4344 |
| bta00591 Linoleic acid metabolism | 19.44 | 0.441 |
| bta01040 Biosynthesis of unsaturated fatty acids | 20.00 | 0.4418 |
| bta00600 Sphingolipid metabolism | 18.37 | 0.4452 |
| bta04142 Lysosome | 16.67 | 0.4452 |
| bta04918 Thyroid hormone synthesis | 17.57 | 0.4452 |
| bta04932 Non-alcoholic fatty liver disease (NAFLD) | 16.46 | 0.4452 |
| bta04970 Salivary secretion | 17.20 | 0.4452 |
| bta00260 Glycine. serine and threonine metabolism | 18.60 | 0.4471 |
| bta04659 Th17 cell differentiation | 16.81 | 0.4471 |
| bta00512 Mucin type O-glycan biosynthesis | 19.35 | 0.4571 |
| bta04120 Ubiquitin mediated proteolysis | 16.43 | 0.4587 |
| bta04979 Cholesterol metabolism | 18.00 | 0.4597 |
| bta00982 Drug metabolism | 17.46 | 0.46 |
| bta04714 Thermogenesis | 15.90 | 0.46 |
| bta05165 Human papillomavirus infection | 15.65 | 0.46 |
| bta00620 Pyruvate metabolism | 18.42 | 0.4675 |
| bta04976 Bile secretion | 16.87 | 0.4682 |
| bta05321 Inflammatory bowel disease (IBD) | 17.14 | 0.4682 |
| bta05016 Huntington disease | 15.69 | 0.4695 |
| bta05204 Chemical carcinogenesis | 16.88 | 0.4732 |
| bta01212 Fatty acid metabolism | 17.24 | 0.477 |
| bta05134 Legionellosis | 17.24 | 0.477 |
| bta04744 Phototransduction | 18.52 | 0.5035 |
| bta05216 Thyroid cancer | 17.50 | 0.5068 |
| bta00480 Glutathione metabolism | 16.67 | 0.5165 |
| bta05145 Toxoplasmosis | 15.93 | 0.5178 |
| bta00030 Pentose phosphate pathway | 17.86 | 0.5284 |
| bta04080 Neuroactive ligand-receptor interaction | 15.15 | 0.5337 |
| bta04060 Cytokine-cytokine receptor interaction | 15.17 | 0.5361 |
| bta00062 Fatty acid elongation | 17.24 | 0.5434 |
| bta00590 Arachidonic acid metabolism | 15.85 | 0.5434 |
| bta00601 Glycosphingolipid biosynthesis | 17.24 | 0.5434 |
| bta03020 RNA polymerase | 17.24 | 0.5434 |
| bta04392 Hippo signaling pathway | 17.24 | 0.5434 |
| bta04512 ECM-receptor interaction | 15.73 | 0.5434 |
| bta00983 Drug metabolism | 15.79 | 0.5489 |
| bta05217 Basal cell carcinoma | 15.87 | 0.5553 |
| bta00513 Various types of N-glycan biosynthesis | 16.28 | 0.5559 |
| bta00520 Amino sugar and nucleotide sugar metabolism | 16.00 | 0.5608 |
| bta04960 Aldosterone-regulated sodium reabsorption | 16.22 | 0.5706 |
| bta05010 Alzheimer disease | 15.00 | 0.5741 |
| bta04610 Complement and coagulation cascades | 15.22 | 0.5806 |
| bta04710 Circadian rhythm | 16.13 | 0.5858 |
| bta00500 Starch and sucrose metabolism | 15.63 | 0.6133 |
| bta00980 Metabolism of xenobiotics by cytochrome P450 | 14.93 | 0.6199 |
| bta00860 Porphyrin and chlorophyll metabolism | 15.00 | 0.6341 |
| bta05033 Nicotine addiction | 15.00 | 0.6341 |
| bta05340 Primary immunodeficiency | 14.63 | 0.6571 |
| bta00240 Pyrimidine metabolism | 14.29 | 0.6617 |
| bta04114 Oocyte meiosis | 14.29 | 0.6617 |
| bta04115 p53 signaling pathway | 14.29 | 0.6617 |
| bta04658 Th1 and Th2 cell differentiation | 14.29 | 0.6617 |
| bta04672 Intestinal immune network for IgA production | 14.29 | 0.6617 |
| bta03440 Homologous recombination | 14.29 | 0.6656 |
| bta05140 Leishmaniasis | 14.10 | 0.6689 |
| bta00790 Folate biosynthesis | 13.89 | 0.6911 |
| bta04110 Cell cycle | 13.82 | 0.6979 |
| bta05143 African trypanosomiasis | 13.64 | 0.7012 |
| bta04630 JAK-STAT signaling pathway | 13.86 | 0.7015 |
| bta00140 Steroid hormone biosynthesis | 13.43 | 0.7127 |
| bta04623 Cytosolic DNA-sensing pathway | 13.43 | 0.7127 |
| bta05323 Rheumatoid arthritis | 13.46 | 0.7193 |
| bta04330 Notch signaling pathway | 13.21 | 0.7208 |
| bta05206 MicroRNAs in cancer | 13.70 | 0.7292 |
| bta05416 Viral myocarditis | 13.16 | 0.7292 |
| bta00350 Tyrosine metabolism | 12.82 | 0.7308 |
| bta00760 Nicotinate and nicotinamide metabolism | 12.82 | 0.7308 |
| bta04657 IL-17 signaling pathway | 13.04 | 0.7391 |
| bta04978 Mineral absorption | 12.73 | 0.7411 |
| bta03040 Spliceosome | 12.93 | 0.7736 |
| bta03013 RNA transport | 12.92 | 0.7856 |
| bta03460 Fanconi anemia pathway | 11.54 | 0.8118 |
| bta05203 Viral carcinogenesis | 12.86 | 0.8129 |
| bta03050 Proteasome | 10.87 | 0.8379 |
| bta04742 Taste transduction | 11.39 | 0.8441 |
| bta00830 Retinol metabolism | 10.94 | 0.8532 |
| bta00970 Aminoacyl-tRNA biosynthesis | 10.61 | 0.8727 |
| bta04612 Antigen processing and presentation | 10.59 | 0.8937 |
| bta05012 Parkinson disease | 11.33 | 0.8967 |
| bta05034 Alcoholism | 11.79 | 0.9003 |
| bta04622 RIG-I-like receptor signaling pathway | 9.80 | 0.9424 |
| bta04974 Protein digestion and absorption | 9.92 | 0.949 |
| bta04145 Phagosome | 10.00 | 0.97 |
| bta05150 Staphylococcus aureus infection | 8.57 | 0.9762 |
| bta05168 Herpes simplex virus 1 infection | 11.17 | 0.9762 |
| bta00190 Oxidative phosphorylation | 7.14 | 1.0 |
| bta03010 Ribosome | 3.13 | 1.0 |
| bta04740 Olfactory transduction | 1.92 | 1.0 |
| bta05322 Systemic lupus erythematosus | 3.85 | 1.0 |
| ^1^%: Percent of genes predicted to be modulated. ^2^BH: Benjamini – Hochberg | | |
