# Supplementary material for: The bovine oviductal environment and composition are negatively affected by elevated body energy reserves
Source: PLoS One. 2025 Jun 23;20(6):e0326138. doi: 10.1371/journal.pone.0326138 (PMC12184905; doi:10.1371/journal.pone.0326138)
Supplement: S7 Table — (DOCX) [file pone.0326138.s010.docx]

| **Supplementary table 7.** Biological patwhays predicted as modulated by miRNAs up regulated in isthmic extracellular vesicles (IST-EVs) in high body energy reserve (HBER) group. | | |
| --- | --- | --- |
| Pathway | %^1^ | BH^2^ |
| bta04144 Endocytosis | 48.98 | 0.0 |
| bta04360 Axon guidance | 49.44 | 0.0 |
| bta04722 Neurotrophin signaling pathway | 53.28 | 0.0 |
| bta05200 Pathways in cancer | 40.41 | 0.0 |
| bta04010 MAPK signaling pathway | 43.15 | 0.0045 |
| bta04014 Ras signaling pathway | 45.04 | 0.0045 |
| bta05212 Pancreatic cancer | 59.21 | 0.0045 |
| bta04062 Chemokine signaling pathway | 45.74 | 0.0078 |
| bta04390 Hippo signaling pathway | 46.15 | 0.0171 |
| bta04514 Cell adhesion molecules (CAMs) | 45.57 | 0.0171 |
| bta05220 Chronic myeloid leukemia | 54.55 | 0.0171 |
| bta01522 Endocrine resistance | 48.94 | 0.025 |
| bta04072 Phospholipase D signaling pathway | 44.74 | 0.025 |
| bta04150 mTOR signaling pathway | 43.95 | 0.025 |
| bta04310 Wnt signaling pathway | 44.44 | 0.025 |
| bta04550 Signaling pathways regulating pluripotency of stem cells | 45.07 | 0.025 |
| bta04611 Platelet activation | 46.28 | 0.025 |
| bta04810 Regulation of actin cytoskeleton | 42.18 | 0.025 |
| bta04910 Insulin signaling pathway | 45.00 | 0.025 |
| bta04934 Cushing syndrome | 44.23 | 0.025 |
| bta05100 Bacterial invasion of epithelial cells | 52.05 | 0.025 |
| bta05132 Salmonella infection | 41.07 | 0.025 |
| bta05211 Renal cell carcinoma | 53.52 | 0.025 |
| bta05214 Glioma | 51.95 | 0.025 |
| bta05224 Breast cancer | 44.00 | 0.025 |
| bta05205 Proteoglycans in cancer | 41.46 | 0.0253 |
| bta04931 Insulin resistance | 46.36 | 0.0302 |
| bta05223 Non-small cell lung cancer | 52.24 | 0.0302 |
| bta05231 Choline metabolism in cancer | 47.47 | 0.0302 |
| bta04261 Adrenergic signaling in cardiomyocytes | 43.33 | 0.0303 |
| bta04721 Synaptic vesicle cycle | 50.00 | 0.0303 |
| bta05163 Human cytomegalovirus infection | 39.59 | 0.0304 |
| bta05225 Hepatocellular carcinoma | 41.95 | 0.0304 |
| bta05215 Prostate cancer | 46.94 | 0.0322 |
| bta01100 Metabolic pathways | 32.61 | 0.033 |
| bta04710 Circadian rhythm | 64.52 | 0.033 |
| bta04928 Parathyroid hormone synthesis. secretion and action | 46.15 | 0.033 |
| bta04520 Adherens junction | 50.00 | 0.0377 |
| bta05210 Colorectal cancer | 47.19 | 0.0377 |
| bta04012 ErbB signaling pathway | 47.62 | 0.0388 |
| bta04921 Oxytocin signaling pathway | 42.11 | 0.0388 |
| bta05219 Bladder cancer | 57.14 | 0.0388 |
| bta05160 Hepatitis C | 40.85 | 0.0524 |
| bta05226 Gastric cancer | 41.18 | 0.0555 |
| bta05217 Basal cell carcinoma | 49.21 | 0.0612 |
| bta04071 Sphingolipid signaling pathway | 42.50 | 0.0667 |
| bta04750 Inflammatory mediator regulation of TRP channels | 43.69 | 0.0673 |
| bta00562 Inositol phosphate metabolism | 46.58 | 0.0676 |
| bta04015 Rap1 signaling pathway | 38.43 | 0.0676 |
| bta04140 Autophagy | 40.85 | 0.0676 |
| bta04350 TGF-beta signaling pathway | 44.09 | 0.0676 |
| bta04659 Th17 cell differentiation | 42.48 | 0.0676 |
| bta04666 Fc gamma R-mediated phagocytosis | 44.09 | 0.0676 |
| bta05032 Morphine addiction | 44.57 | 0.0676 |
| bta05213 Endometrial cancer | 49.15 | 0.0676 |
| bta05218 Melanoma | 46.58 | 0.0676 |
| bta00600 Sphingolipid metabolism | 51.02 | 0.0679 |
| bta04070 Phosphatidylinositol signaling system | 43.43 | 0.0679 |
| bta04916 Melanogenesis | 43.14 | 0.0679 |
| bta04530 Tight junction | 38.98 | 0.0741 |
| bta05167 Kaposi sarcoma-associated herpesvirus infection | 37.86 | 0.0836 |
| bta05221 Acute myeloid leukemia | 45.59 | 0.0959 |
| bta04727 GABAergic synapse | 42.86 | 0.0964 |
| bta04926 Relaxin signaling pathway | 40.00 | 0.1001 |
| bta05135 Yersinia infection | 40.00 | 0.1001 |
| bta05170 Human immunodeficiency virus 1 infection | 36.75 | 0.1001 |
| bta00564 Glycerophospholipid metabolism | 41.35 | 0.1027 |
| bta04068 FoxO signaling pathway | 39.69 | 0.1027 |
| bta04371 Apelin signaling pathway | 39.29 | 0.1027 |
| bta04660 T cell receptor signaling pathway | 41.12 | 0.1027 |
| bta04670 Leukocyte transendothelial migration | 40.71 | 0.1027 |
| bta04110 Cell cycle | 39.84 | 0.1073 |
| bta04151 PI3K-Akt signaling pathway | 34.58 | 0.1073 |
| bta04152 AMPK signaling pathway | 39.84 | 0.1073 |
| bta04912 GnRH signaling pathway | 41.94 | 0.1073 |
| bta04625 C-type lectin receptor signaling pathway | 40.57 | 0.1125 |
| bta04668 TNF signaling pathway | 39.83 | 0.1125 |
| bta04919 Thyroid hormone signaling pathway | 39.83 | 0.1125 |
| bta05216 Thyroid cancer | 50.00 | 0.1125 |
| bta04024 cAMP signaling pathway | 36.24 | 0.1135 |
| bta01521 EGFR tyrosine kinase inhibitor resistance | 42.50 | 0.1137 |
| bta05133 Pertussis | 42.86 | 0.1137 |
| bta04728 Dopaminergic synapse | 38.81 | 0.1169 |
| bta04370 VEGF signaling pathway | 44.83 | 0.1271 |
| bta04966 Collecting duct acid secretion | 53.57 | 0.1293 |
| bta04664 Fc epsilon RI signaling pathway | 42.86 | 0.1313 |
| bta04961 Endocrine and other factor-regulated calcium reabsorption | 46.00 | 0.1313 |
| bta05412 Arrhythmogenic right ventricular cardiomyopathy (ARVC) | 42.11 | 0.1327 |
| bta05165 Human papillomavirus infection | 34.20 | 0.1354 |
| bta00604 Glycosphingolipid biosynthesis | 62.50 | 0.1436 |
| bta05169 Epstein-Barr virus infection | 35.53 | 0.1438 |
| bta04022 cGMP-PKG signaling pathway | 36.69 | 0.1504 |
| bta04730 Long-term depression | 43.33 | 0.1504 |
| bta04725 Cholinergic synapse | 38.60 | 0.1548 |
| bta05414 Dilated cardiomyopathy (DCM) | 39.39 | 0.1558 |
| bta05166 Human T-cell leukemia virus 1 infection | 35.04 | 0.1637 |
| bta01230 Biosynthesis of amino acids | 41.10 | 0.1666 |
| bta04914 Progesterone-mediated oocyte maturation | 39.77 | 0.1666 |
| bta05222 Small cell lung cancer | 39.36 | 0.1666 |
| bta04130 SNARE interactions in vesicular transport | 48.48 | 0.1693 |
| bta01200 Carbon metabolism | 38.05 | 0.1717 |
| bta04066 HIF-1 signaling pathway | 38.18 | 0.1717 |
| bta04726 Serotonergic synapse | 37.93 | 0.1717 |
| bta04510 Focal adhesion | 35.35 | 0.1735 |
| bta05410 Hypertrophic cardiomyopathy (HCM) | 39.13 | 0.1735 |
| bta00052 Galactose metabolism | 48.39 | 0.1819 |
| bta04137 Mitophagy | 40.91 | 0.1828 |
| bta04215 Apoptosis | 47.06 | 0.1828 |
| bta04720 Long-term potentiation | 40.58 | 0.1828 |
| bta04920 Adipocytokine signaling pathway | 40.28 | 0.1828 |
| bta05017 Spinocerebellar ataxia | 38.54 | 0.1828 |
| bta05230 Central carbon metabolism in cancer | 40.91 | 0.1828 |
| bta01210 2-Oxocarboxylic acid metabolism | 55.56 | 0.1856 |
| bta04933 AGE-RAGE signaling pathway in diabetic complications | 37.86 | 0.1883 |
| bta05202 Transcriptional misregulation in cancer | 35.08 | 0.1883 |
| bta04392 Hippo signaling pathway | 48.28 | 0.1894 |
| bta04911 Insulin secretion | 38.82 | 0.1923 |
| bta03015 mRNA surveillance pathway | 37.89 | 0.2038 |
| bta04658 Th1 and Th2 cell differentiation | 37.76 | 0.2038 |
| bta04217 Necroptosis | 35.06 | 0.2071 |
| bta05161 Hepatitis B | 35.09 | 0.208 |
| bta04211 Longevity regulating pathway | 37.78 | 0.2214 |
| bta01524 Platinum drug resistance | 38.46 | 0.2265 |
| bta04020 Calcium signaling pathway | 34.16 | 0.2274 |
| bta04114 Oocyte meiosis | 36.13 | 0.2274 |
| bta04145 Phagosome | 34.71 | 0.2274 |
| bta04915 Estrogen signaling pathway | 35.51 | 0.2274 |
| bta05014 Amyotrophic lateral sclerosis (ALS) | 40.00 | 0.2274 |
| bta00650 Butanoate metabolism | 46.43 | 0.2277 |
| bta04142 Lysosome | 35.61 | 0.2277 |
| bta04724 Glutamatergic synapse | 36.28 | 0.2277 |
| bta04210 Apoptosis | 35.21 | 0.2333 |
| bta00532 Glycosaminoglycan biosynthesis | 50.00 | 0.2338 |
| bta04270 Vascular smooth muscle contraction | 35.34 | 0.2338 |
| bta04657 IL-17 signaling pathway | 36.96 | 0.2338 |
| bta04923 Regulation of lipolysis in adipocytes | 39.66 | 0.2338 |
| bta04962 Vasopressin-regulated water reabsorption | 40.82 | 0.2338 |
| bta05134 Legionellosis | 39.66 | 0.2338 |
| bta04115 p53 signaling pathway | 37.66 | 0.2443 |
| bta04935 Growth hormone synthesis. secretion and action | 35.59 | 0.245 |
| bta04925 Aldosterone synthesis and secretion | 36.46 | 0.2462 |
| bta00500 Starch and sucrose metabolism | 43.75 | 0.2463 |
| bta04064 NF-kappa B signaling pathway | 35.78 | 0.2463 |
| bta04540 Gap junction | 36.67 | 0.2463 |
| bta05020 Prion diseases | 43.75 | 0.2463 |
| bta04146 Peroxisome | 36.90 | 0.2493 |
| bta05418 Fluid shear stress and atherosclerosis | 34.48 | 0.2564 |
| bta05145 Toxoplasmosis | 35.40 | 0.2567 |
| bta05164 Influenza A | 33.70 | 0.2575 |
| bta05323 Rheumatoid arthritis | 35.58 | 0.2642 |
| bta00280 Valine. leucine and isoleucine degradation | 39.22 | 0.2654 |
| bta04340 Hedgehog signaling pathway | 39.22 | 0.2654 |
| bta00514 Other types of O-glycan biosynthesis | 40.00 | 0.2662 |
| bta04971 Gastric acid secretion | 36.84 | 0.2673 |
| bta04218 Cellular senescence | 33.73 | 0.2674 |
| bta00770 Pantothenate and CoA biosynthesis | 47.37 | 0.2801 |
| bta04978 Mineral absorption | 38.18 | 0.2833 |
| bta05235 PD-L1 expression and PD-1 checkpoint pathway in cancer | 35.48 | 0.2869 |
| bta00030 Pentose phosphate pathway | 42.86 | 0.2892 |
| bta00051 Fructose and mannose metabolism | 41.18 | 0.2893 |
| bta04713 Circadian entrainment | 35.00 | 0.2945 |
| bta04213 Longevity regulating pathway | 37.10 | 0.296 |
| bta00533 Glycosaminoglycan biosynthesis | 50.00 | 0.3003 |
| bta04141 Protein processing in endoplasmic reticulum | 33.13 | 0.3048 |
| bta00310 Lysine degradation | 36.36 | 0.3138 |
| bta00515 Mannose type O-glycan biosynthesis | 43.48 | 0.3152 |
| bta04061 Viral protein interaction with cytokine and cytokine receptor | 34.74 | 0.3152 |
| bta05321 Inflammatory bowel disease (IBD) | 35.71 | 0.3305 |
| bta00330 Arginine and proline metabolism | 37.50 | 0.332 |
| bta00561 Glycerolipid metabolism | 35.82 | 0.332 |
| bta04970 Salivary secretion | 34.41 | 0.3364 |
| bta04922 Glucagon signaling pathway | 33.98 | 0.3399 |
| bta03030 DNA replication | 38.89 | 0.3405 |
| bta00640 Propanoate metabolism | 39.39 | 0.3418 |
| bta00630 Glyoxylate and dicarboxylate metabolism | 40.00 | 0.3423 |
| bta04640 Hematopoietic cell lineage | 33.64 | 0.3423 |
| bta04927 Cortisol synthesis and secretion | 35.38 | 0.3523 |
| bta04924 Renin secretion | 34.72 | 0.3659 |
| bta00240 Pyrimidine metabolism | 35.71 | 0.367 |
| bta05031 Amphetamine addiction | 34.78 | 0.3686 |
| bta05142 Chagas disease (American trypanosomiasis) | 33.04 | 0.3699 |
| bta00130 Ubiquinone and other terpenoid-quinone biosynthesis | 50.00 | 0.3704 |
| bta04723 Retrograde endocannabinoid signaling | 32.24 | 0.3704 |
| bta04972 Pancreatic secretion | 33.33 | 0.3704 |
| bta05162 Measles | 32.24 | 0.3704 |
| bta00220 Arginine biosynthesis | 42.11 | 0.3753 |
| bta04973 Carbohydrate digestion and absorption | 36.36 | 0.3769 |
| bta04913 Ovarian steroidogenesis | 35.09 | 0.3784 |
| bta04976 Bile secretion | 33.73 | 0.3784 |
| bta00010 Glycolysis Gluconeogenesis | 34.38 | 0.3866 |
| bta04120 Ubiquitin mediated proteolysis | 32.14 | 0.3866 |
| bta04621 NOD-like receptor signaling pathway | 31.52 | 0.3866 |
| bta04662 B cell receptor signaling pathway | 33.33 | 0.3866 |
| bta00062 Fatty acid elongation | 37.93 | 0.3898 |
| bta00230 Purine metabolism | 32.09 | 0.3898 |
| bta00592 alpha-Linolenic acid metabolism | 37.93 | 0.3898 |
| bta00601 Glycosphingolipid biosynthesis | 37.93 | 0.3898 |
| bta01212 Fatty acid metabolism | 34.48 | 0.3898 |
| bta04216 Ferroptosis | 35.56 | 0.3898 |
| bta03430 Mismatch repair | 39.13 | 0.3959 |
| bta00565 Ether lipid metabolism | 34.62 | 0.3997 |
| bta00603 Glycosphingolipid biosynthesis | 41.18 | 0.3997 |
| bta00910 Nitrogen metabolism | 41.18 | 0.3997 |
| bta00591 Linoleic acid metabolism | 36.11 | 0.4028 |
| bta00270 Cysteine and methionine metabolism | 34.69 | 0.4029 |
| bta01040 Biosynthesis of unsaturated fatty acids | 36.67 | 0.4157 |
| bta04330 Notch signaling pathway | 33.96 | 0.4231 |
| bta05033 Nicotine addiction | 35.00 | 0.4244 |
| bta00520 Amino sugar and nucleotide sugar metabolism | 34.00 | 0.4281 |
| bta04380 Osteoclast differentiation | 31.34 | 0.4281 |
| bta04960 Aldosterone-regulated sodium reabsorption | 35.14 | 0.4281 |
| bta00900 Terpenoid backbone biosynthesis | 38.10 | 0.4298 |
| bta00380 Tryptophan metabolism | 34.04 | 0.4306 |
| bta00410 beta-Alanine metabolism | 35.29 | 0.4322 |
| bta00053 Ascorbate and aldarate metabolism | 36.00 | 0.4614 |
| bta04612 Antigen processing and presentation | 31.76 | 0.4614 |
| bta03420 Nucleotide excision repair | 33.33 | 0.4689 |
| bta04940 Type I diabetes mellitus | 32.20 | 0.4859 |
| bta05416 Viral myocarditis | 31.58 | 0.4859 |
| bta04917 Prolactin signaling pathway | 31.33 | 0.4888 |
| bta04930 Type II diabetes mellitus | 32.61 | 0.4964 |
| bta04614 Renin-angiotensin system | 34.62 | 0.4967 |
| bta00260 Glycine. serine and threonine metabolism | 32.56 | 0.5057 |
| bta00360 Phenylalanine metabolism | 34.78 | 0.5076 |
| bta00100 Steroid biosynthesis | 35.00 | 0.5224 |
| bta04975 Fat digestion and absorption | 31.25 | 0.5499 |
| bta05030 Cocaine addiction | 31.25 | 0.5499 |
| bta05332 Graft-versus-host disease | 31.25 | 0.5499 |
| bta05340 Primary immunodeficiency | 31.71 | 0.5499 |
| bta00040 Pentose and glucuronate interconversions | 32.26 | 0.5512 |
| bta00512 Mucin type O-glycan biosynthesis | 32.26 | 0.5512 |
| bta00071 Fatty acid degradation | 30.95 | 0.5761 |
| bta04672 Intestinal immune network for IgA production | 30.36 | 0.5768 |
| bta00730 Thiamine metabolism | 33.33 | 0.5782 |
| bta04932 Non-alcoholic fatty liver disease (NAFLD) | 29.11 | 0.5783 |
| bta00511 Other glycan degradation | 31.82 | 0.5804 |
| bta00513 Various types of N-glycan biosynthesis | 30.23 | 0.5804 |
| bta00531 Glycosaminoglycan degradation | 31.82 | 0.5804 |
| bta00760 Nicotinate and nicotinamide metabolism | 30.77 | 0.5804 |
| bta00790 Folate biosynthesis | 30.56 | 0.5804 |
| bta03020 RNA polymerase | 31.03 | 0.5804 |
| bta04650 Natural killer cell mediated cytotoxicity | 29.01 | 0.5804 |
| bta04918 Thyroid hormone synthesis | 29.73 | 0.5804 |
| bta05146 Amoebiasis | 29.06 | 0.5804 |
| bta05152 Tuberculosis | 28.79 | 0.5804 |
| bta05203 Viral carcinogenesis | 28.63 | 0.5804 |
| bta00590 Arachidonic acid metabolism | 29.27 | 0.5844 |
| bta00860 Porphyrin and chlorophyll metabolism | 30.00 | 0.5929 |
| bta04136 Autophagy | 30.30 | 0.5929 |
| bta04714 Thermogenesis | 28.45 | 0.5937 |
| bta04929 GnRH secretion | 29.23 | 0.5937 |
| bta03022 Basal transcription factors | 29.55 | 0.6002 |
| bta00250 Alanine. aspartate and glutamate metabolism | 29.73 | 0.6014 |
| bta00510 N-Glycan biosynthesis | 28.85 | 0.6216 |
| bta03460 Fanconi anemia pathway | 28.85 | 0.6216 |
| bta03320 PPAR signaling pathway | 28.40 | 0.6281 |
| bta05140 Leishmaniasis | 28.21 | 0.6406 |
| bta04260 Cardiac muscle contraction | 28.09 | 0.6427 |
| bta04742 Taste transduction | 27.85 | 0.6608 |
| bta04620 Toll-like receptor signaling pathway | 27.27 | 0.7008 |
| bta05016 Huntington disease | 27.37 | 0.7037 |
| bta04964 Proximal tubule bicarbonate reclamation | 27.27 | 0.7117 |
| bta00980 Metabolism of xenobiotics by cytochrome P450 | 26.87 | 0.7158 |
| bta04950 Maturity onset diabetes of the young | 26.92 | 0.7158 |
| bta00020 Citrate cycle (TCA cycle) | 26.67 | 0.7218 |
| bta00830 Retinol metabolism | 26.56 | 0.7275 |
| bta00340 Histidine metabolism | 26.09 | 0.7361 |
| bta03060 Protein export | 26.09 | 0.7361 |
| bta03440 Homologous recombination | 26.19 | 0.7361 |
| bta04744 Phototransduction | 25.93 | 0.739 |
| bta00534 Glycosaminoglycan biosynthesis | 25.00 | 0.7707 |
| bta04060 Cytokine-cytokine receptor interaction | 26.63 | 0.7764 |
| bta00140 Steroid hormone biosynthesis | 25.37 | 0.7812 |
| bta03008 Ribosome biogenesis in eukaryotes | 25.30 | 0.7891 |
| bta03040 Spliceosome | 25.85 | 0.7891 |
| bta05330 Allograft rejection | 25.00 | 0.7891 |
| bta04512 ECM-receptor interaction | 24.72 | 0.8215 |
| bta04977 Vitamin digestion and absorption | 23.08 | 0.8215 |
| bta05310 Asthma | 23.68 | 0.8215 |
| bta05150 Staphylococcus aureus infection | 24.76 | 0.8293 |
| bta04623 Cytosolic DNA-sensing pathway | 23.88 | 0.8342 |
| bta05144 Malaria | 23.73 | 0.8342 |
| bta05168 Herpes simplex virus 1 infection | 26.05 | 0.8342 |
| bta03013 RNA transport | 24.72 | 0.8704 |
| bta03410 Base excision repair | 21.21 | 0.8775 |
| bta04630 JAK-STAT signaling pathway | 24.75 | 0.8775 |
| bta03050 Proteasome | 21.74 | 0.8806 |
| bta04979 Cholesterol metabolism | 22.00 | 0.8806 |
| bta00620 Pyruvate metabolism | 21.05 | 0.8813 |
| bta00982 Drug metabolism | 22.22 | 0.8813 |
| bta03018 RNA degradation | 22.78 | 0.8813 |
| bta00350 Tyrosine metabolism | 20.51 | 0.8869 |
| bta00480 Glutathione metabolism | 21.67 | 0.8869 |
| bta00983 Drug metabolism | 22.37 | 0.8869 |
| bta01523 Antifolate resistance | 20.93 | 0.8869 |
| bta00563 Glycosylphosphatidylinositol (GPI)-anchor biosynthesis | 19.23 | 0.887 |
| bta05204 Chemical carcinogenesis | 22.08 | 0.8888 |
| bta05206 MicroRNAs in cancer | 24.32 | 0.9106 |
| bta04080 Neuroactive ligand-receptor interaction | 24.52 | 0.9144 |
| bta04610 Complement and coagulation cascades | 21.74 | 0.9144 |
| bta05012 Parkinson disease | 22.67 | 0.9171 |
| bta05034 Alcoholism | 23.58 | 0.9171 |
| bta05010 Alzheimer disease | 22.22 | 0.9507 |
| bta04622 RIG-I-like receptor signaling pathway | 19.61 | 0.9699 |
| bta05320 Autoimmune thyroid disease | 18.31 | 0.9699 |
| bta05143 African trypanosomiasis | 15.91 | 0.9707 |
| bta00970 Aminoacyl-tRNA biosynthesis | 16.67 | 0.9837 |
| bta04974 Protein digestion and absorption | 19.01 | 0.9837 |
| bta00190 Oxidative phosphorylation | 19.29 | 0.9848 |
| bta02010 ABC transporters | 15.00 | 0.9873 |
| bta03010 Ribosome | 10.63 | 1.0 |
| bta04740 Olfactory transduction | 3.49 | 1.0 |
| bta05322 Systemic lupus erythematosus | 13.74 | 1.0 |
| ^1^%: Percent of genes predicted to be modulated. ^2^BH: Benjamini – Hochberg | | |
