# Supplementary material for: The bovine oviductal environment and composition are negatively affected by elevated body energy reserves
Source: PLoS One. 2025 Jun 23;20(6):e0326138. doi: 10.1371/journal.pone.0326138 (PMC12184905; doi:10.1371/journal.pone.0326138)
Supplement: S8 Table — (DOCX) [file pone.0326138.s011.docx]

| **Supplementary Table 8.** Raw cycle threshold levels of the 383 miRNAs profile in ampullary luminal epithelial cells (AMP-Cell) of cows with different body energy reserve. | | | | | | |
| --- | --- | --- | --- | --- | --- | --- |
| **miRNA** | **Body energy reserve^1^** | | | | | |
|  | **MBER** | | | **HBER** | | |
|  | **1** | **2** | **3** | **1** | **2** | **3** |
| bta-let-7a-3p | 28.835 | 27.905 | 28.312 | 25.948 | 26.802 | 27.546 |
| bta-miR-103 | 27.812 | 26.750 | 29.643 | 25.125 | 26.348 | 26.224 |
| bta-let-7a-5p | 22.837 | 21.861 | 22.618 | 19.972 | 21.518 | 21.505 |
| bta-miR-105a | . | . | . | 33.127 | . | 34.920 |
| bta-let-7b | 24.590 | 24.268 | 23.595 | 20.940 | 23.208 | 22.363 |
| bta-miR-105b | 33.512 | 33.868 | 36.658 | 31.140 | 31.606 | 32.872 |
| bta-let-7c | 23.417 | 22.683 | 22.704 | 20.415 | 21.867 | 21.436 |
| bta-miR-106a | 28.883 | 28.003 | 29.743 | 25.633 | 27.330 | 26.732 |
| bta-let-7d | 24.394 | 23.719 | 23.721 | 21.575 | 22.964 | 22.700 |
| bta-miR-106b | 30.825 | 30.006 | . | 27.614 | 29.287 | 28.813 |
| bta-let-7e | 22.094 | 22.168 | 22.372 | 19.524 | 20.673 | 20.859 |
| bta-miR-107 | 31.754 | 31.482 | 32.886 | 29.026 | 30.573 | 30.988 |
| bta-let-7f | 23.724 | 22.874 | 24.335 | 21.619 | 22.299 | 22.780 |
| bta-miR-10a | 26.805 | 25.602 | 26.620 | 24.553 | 25.531 | 26.361 |
| bta-let-7g | 25.214 | 24.478 | 25.771 | 22.793 | 23.713 | 23.792 |
| bta-miR-10b | 27.011 | 25.852 | 27.361 | 24.765 | 25.771 | 26.661 |
| bta-let-7i | 27.645 | 26.284 | 28.800 | 24.358 | 25.833 | 25.575 |
| bta-miR-122 | . | . | . | 33.595 | . | . |
| bta-miR-1 | 29.753 | 28.446 | 33.147 | 27.745 | 27.849 | 29.217 |
| bta-miR-124a | . | . | . | 34.805 | 35.930 | 34.748 |
| bta-miR-100 | 28.730 | 27.774 | 28.272 | 25.067 | 26.785 | 25.748 |
| bta-miR-124b | . | . | . | 33.665 | 35.529 | . |
| bta-miR-101 | 32.592 | 31.466 | 30.767 | 27.666 | 29.578 | 29.209 |
| bta-miR-125a | 24.784 | 23.869 | 24.832 | 22.493 | 23.143 | 23.747 |
| bta-miR-125b | 24.080 | 23.013 | 24.680 | 20.651 | 21.742 | 21.709 |
| bta-miR-133b | . | . | . | 34.926 | . | . |
| bta-miR-126-3p | 33.414 | 32.750 | 33.554 | 29.441 | 32.930 | . |
| bta-miR-133c | 34.550 | . | . | 32.839 | 36.070 | . |
| bta-miR-126-5p | . | 30.810 | . | 28.720 | 30.636 | . |
| bta-miR-134 | 35.056 | . | . | 28.700 | 35.250 | 34.010 |
| bta-miR-127 | 33.307 | 33.842 | 33.644 | 32.041 | 32.896 | 33.192 |
| bta-miR-135a | 26.402 | 24.728 | 25.220 | 22.100 | 24.096 | 23.752 |
| bta-miR-128 | 28.859 | 28.779 | 30.507 | 26.734 | 28.423 | 28.488 |
| bta-miR-135b | 27.902 | 26.433 | 26.624 | 23.815 | 25.702 | 25.244 |
| bta-miR-129 | 31.674 | 32.034 | . | 31.742 | 31.864 | . |
| bta-miR-136 | . | . | . | . | . | . |
| bta-miR-129-3p | . | 35.744 | 33.760 | 30.791 | 35.106 | 32.848 |
| bta-miR-137 | . | . | . | 35.818 | . | . |
| bta-miR-129-5p | 33.022 | 32.341 | 34.813 | 31.803 | 31.792 | 31.725 |
| bta-miR-138 | 35.016 | 32.743 | . | 27.814 | 33.678 | 31.880 |
| bta-miR-130a | 32.313 | 32.528 | 32.820 | 29.783 | 30.976 | 30.824 |
| bta-miR-139 | 31.501 | 29.952 | 32.037 | 28.014 | 30.638 | 29.797 |
| bta-miR-130b | 25.594 | 24.895 | 26.192 | 23.511 | 24.646 | 24.840 |
| bta-miR-140 | 29.823 | 28.927 | 31.548 | 27.449 | 27.975 | 29.328 |
| bta-miR-132 | 31.642 | 31.600 | 33.805 | 28.651 | 30.746 | 31.117 |
| bta-miR-141 | 29.031 | 27.926 | 29.172 | 25.790 | 27.271 | 26.804 |
| bta-miR-133a | 36.909 | 33.939 | 35.422 | 29.166 | 33.570 | 31.756 |
| bta-miR-142-3p | . | . | . | 32.758 | . | . |
| bta-miR-142-5p | 34.961 | 35.636 | 34.315 | 31.234 | 36.592 | . |
| bta-miR-151-3p | 27.805 | 26.687 | 28.651 | 25.495 | 26.320 | 27.273 |
| bta-miR-143 | 31.686 | 32.371 | 30.728 | 29.197 | 31.419 | 30.724 |
| bta-miR-151-5p | 26.272 | 24.914 | 27.127 | 23.602 | 24.557 | 25.122 |
| bta-miR-144 | . | . | . | . | . | . |
| bta-miR-152 | 33.159 | 32.584 | . | 29.687 | 31.876 | 31.904 |
| bta-miR-145 | 31.762 | 31.846 | 31.320 | 27.749 | 30.596 | 29.834 |
| bta-miR-153 | 34.399 | 33.401 | 31.806 | 29.802 | 34.479 | 30.867 |
| bta-miR-146a | 34.960 | 33.890 | 35.063 | 33.883 | 35.807 | 33.491 |
| bta-miR-154a | 35.936 | 36.820 | 34.401 | 29.947 | 34.946 | 35.040 |
| bta-miR-146b | 34.632 | 34.690 | . | 31.843 | 33.390 | 36.375 |
| bta-miR-154b | 30.700 | 32.932 | 31.903 | 27.781 | 31.925 | 31.812 |
| bta-miR-147 | . | 33.405 | . | 33.918 | 33.248 | . |
| bta-miR-154c | 35.103 | . | . | 32.848 | . | 36.964 |
| bta-miR-148a | 24.389 | 22.910 | 23.444 | 20.194 | 21.834 | 21.838 |
| bta-miR-155 | 34.052 | 31.806 | 33.705 | 29.827 | 32.312 | 32.198 |
| bta-miR-148b | 24.995 | 23.991 | 23.896 | 20.644 | 22.812 | 22.580 |
| bta-miR-15a | 30.751 | 29.750 | 32.645 | 27.554 | 29.380 | 28.807 |
| bta-miR-149-3p | 31.909 | 31.799 | . | 28.584 | 30.734 | 29.409 |
| bta-miR-15b | 27.065 | 26.754 | 27.581 | 24.709 | 26.432 | 26.269 |
| bta-miR-149-5p | 32.558 | 34.231 | 36.096 | 28.856 | 32.426 | 31.866 |
| bta-miR-16a | 26.764 | 25.925 | 27.543 | 23.811 | 25.523 | 24.872 |
| bta-miR-150 | 29.708 | 29.241 | 31.672 | 28.154 | 29.265 | 29.766 |
| bta-miR-16b | 25.933 | 24.921 | 26.809 | 23.068 | 24.606 | 24.132 |
| bta-miR-17-3p | 34.500 | 33.246 | . | 31.534 | 33.478 | 32.799 |
| bta-miR-188 | 34.972 | 33.322 | 33.988 | 31.070 | 32.326 | 32.885 |
| bta-miR-17-5p | 33.416 | 31.832 | 33.064 | 29.806 | 30.832 | 30.786 |
| bta-miR-18a | 34.906 | 32.654 | . | 31.415 | 32.135 | 31.831 |
| bta-miR-181a | 33.587 | 33.170 | . | . | 32.883 | 32.293 |
| bta-miR-18b | . | 35.070 | . | 32.947 | 36.846 | 33.759 |
| bta-miR-181b | 31.764 | 30.696 | 31.904 | 29.556 | 30.700 | 31.731 |
| bta-miR-190a | 34.394 | 32.705 | 32.833 | 29.726 | 31.777 | 30.289 |
| bta-miR-181c | . | . | . | 32.692 | . | 33.543 |
| bta-miR-190b | 25.796 | 25.115 | 28.669 | 25.165 | 24.937 | 26.408 |
| bta-miR-181d | 30.807 | 29.738 | 31.808 | 28.025 | 30.100 | 29.310 |
| bta-miR-191 | 25.370 | 24.613 | 26.775 | 23.111 | 23.834 | 24.759 |
| bta-miR-182 | 29.791 | 30.084 | 31.363 | 27.572 | 29.714 | 29.672 |
| bta-miR-192 | 32.004 | 31.491 | 33.972 | 29.769 | 30.531 | 32.207 |
| bta-miR-183 | 30.827 | 30.566 | 31.693 | 28.764 | 30.167 | 30.438 |
| bta-miR-193a | . | . | . | 35.188 | . | . |
| bta-miR-184 | . | . | . | 31.793 | . | 36.414 |
| bta-miR-193a-3p | . | 35.014 | . | . | 36.340 | . |
| bta-miR-185 | 29.644 | 28.796 | 30.671 | 27.251 | 28.716 | 28.708 |
| bta-miR-193a-5p | 30.655 | 30.149 | 30.544 | 27.558 | 30.134 | 29.755 |
| bta-miR-186 | 29.080 | 27.631 | 29.724 | 25.128 | 27.208 | 27.000 |
| bta-miR-193b | . | . | . | 32.825 | 34.998 | . |
| bta-miR-187 | . | 32.502 | 33.116 | 29.305 | . | 30.796 |
| bta-miR-194 | 30.761 | 29.967 | 30.603 | 27.455 | 28.925 | 28.776 |
| bta-miR-195 | 26.657 | 25.709 | 28.131 | 24.318 | 24.966 | 25.005 |
| bta-miR-200c | 21.721 | 20.956 | 22.677 | 19.736 | 20.662 | 21.409 |
| bta-miR-196a | . | . | 35.109 | 30.781 | 35.183 | 36.841 |
| bta-miR-202 | 33.491 | . | . | 31.283 | 31.828 | 32.778 |
| bta-miR-196b | . | . | . | 33.530 | 35.010 | 34.292 |
| bta-miR-204 | 26.846 | 25.974 | 26.055 | 23.693 | 26.473 | 28.142 |
| bta-miR-197 | 27.332 | 26.531 | 27.947 | 25.844 | 26.708 | 27.691 |
| bta-miR-205 | 33.866 | 31.613 | 30.762 | 28.270 | 28.695 | 29.835 |
| bta-miR-199a-3p | 32.917 | 35.011 | 34.318 | 30.784 | 32.895 | 33.034 |
| bta-miR-206 | 34.792 | 33.830 | 34.962 | 29.437 | 34.110 | 34.425 |
| bta-miR-199a-5p | . | . | . | . | . | . |
| bta-miR-208a | 34.497 | 34.456 | . | . | 34.525 | . |
| bta-miR-199b | . | . | . | 32.949 | . | . |
| bta-miR-208b | . | . | . | 36.671 | . | . |
| bta-miR-199c | 31.962 | 30.932 | 32.293 | 29.676 | 30.751 | 32.958 |
| bta-miR-20a | 29.204 | 27.933 | 29.435 | 25.667 | 27.265 | 26.721 |
| bta-miR-19a | 32.147 | 30.673 | 32.467 | 27.365 | 29.809 | 28.222 |
| bta-miR-20b | 30.457 | 29.449 | 31.599 | 26.901 | 28.642 | 28.599 |
| bta-miR-19b | 32.102 | 31.636 | 30.183 | 27.324 | 29.703 | 27.771 |
| bta-miR-21-3p | . | . | . | 32.905 | 33.884 | 33.987 |
| bta-miR-200a | 30.186 | 28.698 | 30.670 | 26.354 | 27.854 | 27.702 |
| bta-miR-21-5p | 32.131 | 29.940 | 29.834 | 27.958 | 28.761 | 28.822 |
| bta-miR-200b | 20.223 | 19.532 | 21.196 | 18.334 | 18.728 | 19.820 |
| bta-miR-210 | 31.818 | 29.791 | 31.889 | 28.218 | 28.792 | 29.306 |
| bta-miR-211 | 27.641 | 26.691 | 26.805 | 24.637 | 26.876 | 28.372 |
| bta-miR-22-5p | 31.126 | 29.986 | 32.133 | 28.048 | 30.635 | 30.055 |
| bta-miR-212 | . | . | . | 34.160 | . | . |
| bta-miR-221 | 28.811 | 27.818 | 30.797 | 26.706 | 27.690 | 27.702 |
| bta-miR-214 | 33.435 | 34.511 | 34.053 | 31.701 | 35.289 | 32.421 |
| bta-miR-222 | 27.784 | 27.437 | . | 25.758 | 26.832 | 26.933 |
| bta-miR-215 | 31.831 | 30.796 | 31.723 | 29.240 | 29.756 | 31.665 |
| bta-miR-223 | 33.788 | 33.279 | 35.684 | 28.814 | 31.614 | 30.789 |
| bta-miR-216a | 36.766 | 34.494 | 34.498 | 31.970 | 33.476 | 33.176 |
| bta-miR-224 | 29.616 | 29.013 | 31.536 | 27.568 | 29.527 | 29.674 |
| bta-miR-216b | 34.293 | 32.585 | . | 32.167 | 32.638 | 33.970 |
| bta-miR-23a | 22.011 | 21.564 | 23.064 | 20.748 | 21.025 | 22.047 |
| bta-miR-217 | . | . | . | . | . | . |
| bta-miR-23b-3p | 24.927 | 24.817 | 26.077 | 23.656 | 23.708 | 24.815 |
| bta-miR-218 | 30.723 | 29.691 | 32.643 | 29.594 | 30.321 | 31.038 |
| bta-miR-23b-5p | 34.004 | 33.238 | . | 32.678 | 34.004 | 36.965 |
| bta-miR-219 | 34.481 | 34.798 | . | 29.768 | 32.951 | 32.606 |
| bta-miR-24 | . | . | . | 36.538 | . | . |
| bta-miR-219-3p | 32.403 | 32.930 | 33.141 | 30.234 | 31.693 | 31.581 |
| bta-miR-24-3p | 25.695 | 24.822 | 26.477 | 22.951 | 24.224 | 24.419 |
| bta-miR-219-5p | . | . | . | . | . | . |
| bta-miR-25 | 25.477 | 24.793 | 26.566 | 23.831 | 24.310 | 25.220 |
| bta-miR-22-3p | 2.426 | 2.748 | 2.817 | 2.779 | 2.272 | 2.824 |
| bta-miR-26a | 22.296 | 21.383 | 22.637 | 19.573 | 20.403 | 21.331 |
| bta-miR-26b | 23.787 | 22.722 | 24.187 | 21.106 | 22.425 | 23.050 |
| bta-miR-29d-3p | 26.838 | 25.756 | 26.265 | 23.757 | 24.800 | 24.739 |
| bta-miR-26c | . | . | . | . | . | . |
| bta-miR-29d-5p | 31.457 | 29.689 | 31.184 | 28.423 | 29.279 | 30.028 |
| bta-miR-27a-3p | 27.001 | 25.765 | 26.328 | 23.690 | 25.366 | 24.769 |
| bta-miR-29e | . | 34.765 | . | 33.198 | . | 34.809 |
| bta-miR-27a-5p | 33.258 | 32.788 | . | 31.417 | 32.716 | 32.869 |
| bta-miR-301a | . | . | . | 33.652 | . | . |
| bta-miR-27b | 26.736 | 25.919 | 26.629 | 23.715 | 24.787 | 25.205 |
| bta-miR-301b | . | . | . | . | . | 36.137 |
| bta-miR-28 | 30.780 | 30.021 | 31.635 | 27.934 | 29.013 | 29.857 |
| bta-miR-302a | . | . | . | 35.965 | . | . |
| bta-miR-296-3p | . | . | 31.529 | 29.507 | 30.258 | 30.581 |
| bta-miR-302b | . | . | . | 32.691 | 34.086 | . |
| bta-miR-296-5p | 33.955 | 32.162 | 32.812 | 28.963 | 31.262 | . |
| bta-miR-302c | 36.755 | . | . | 31.919 | . | 33.931 |
| bta-miR-299 | 35.290 | 35.000 | . | 32.070 | . | . |
| bta-miR-302d | . | 35.783 | . | . | . | 34.683 |
| bta-miR-29a | 24.390 | 23.484 | 24.061 | 21.406 | 22.752 | 22.878 |
| bta-miR-3064 | 34.924 | 33.333 | . | 31.804 | 36.939 | 34.220 |
| bta-miR-29b | 36.727 | 34.860 | 33.639 | 29.961 | 34.999 | 31.695 |
| bta-miR-30a-5p | 29.462 | 28.402 | 29.398 | 25.178 | 27.236 | 26.524 |
| bta-miR-29c | 24.531 | 23.498 | 23.849 | 21.560 | 22.749 | 22.781 |
| bta-miR-30b-3p | 32.126 | 31.335 | 33.585 | 30.268 | 30.855 | 31.989 |
| bta-miR-30b-5p | 27.879 | 26.553 | 26.713 | 23.186 | 25.649 | 24.640 |
| bta-miR-328 | 33.452 | 30.231 | 30.745 | 28.935 | 30.591 | 29.813 |
| bta-miR-30c | 25.861 | 24.721 | 25.570 | 22.164 | 23.815 | 23.703 |
| bta-miR-329a | . | . | . | . | . | . |
| bta-miR-30d | 29.191 | 28.184 | 29.073 | 25.392 | 27.689 | 26.625 |
| bta-miR-329b | . | 36.599 | . | 33.880 | . | 35.698 |
| bta-miR-30e-5p | 29.510 | 28.806 | 29.265 | 25.336 | 27.518 | 26.691 |
| bta-miR-330 | 35.360 | 32.912 | 33.658 | 32.604 | 36.909 | 33.687 |
| bta-miR-30f | 27.767 | 26.589 | 27.257 | 23.781 | 25.788 | 25.486 |
| bta-miR-331-3p | 31.495 | 30.017 | 35.825 | 28.384 | 29.707 | 30.159 |
| bta-miR-31 | 25.694 | 24.773 | 26.884 | 23.463 | 24.245 | 24.853 |
| bta-miR-331-5p | 31.676 | 30.093 | 31.759 | 28.548 | 30.732 | 30.750 |
| bta-miR-32 | . | 35.721 | . | . | . | . |
| bta-miR-335 | 32.824 | 33.905 | 32.646 | 29.548 | 30.733 | 30.568 |
| bta-miR-320a | 27.035 | 26.753 | 28.570 | 24.800 | 26.661 | 26.722 |
| bta-miR-338 | . | 34.444 | 34.839 | 31.796 | 33.689 | 31.697 |
| bta-miR-320b | 36.668 | 35.745 | . | 30.833 | 33.919 | 33.817 |
| bta-miR-339a | 30.081 | 29.411 | 30.795 | 26.760 | 28.460 | 28.690 |
| bta-miR-323 | 17.002 | 17.610 | 17.163 | 17.530 | 17.301 | 17.567 |
| bta-miR-339b | 29.516 | 28.775 | 29.980 | 26.255 | 27.905 | 27.812 |
| bta-miR-324 | 33.974 | 32.930 | 34.575 | 29.170 | . | 32.384 |
| bta-miR-33a | . | . | 36.053 | 33.196 | . | 35.250 |
| bta-miR-326 | 33.450 | 33.690 | 34.390 | 30.716 | 33.025 | 32.868 |
| bta-miR-33b | 35.322 | 34.995 | 35.637 | 33.830 | 34.906 | 36.357 |
| bta-miR-340 | 33.591 | 31.617 | 31.917 | 29.389 | 31.467 | 31.684 |
| bta-miR-365-3p | 29.511 | 28.546 | 29.166 | 25.737 | 27.111 | 27.454 |
| bta-miR-342 | 30.818 | 29.725 | 30.784 | 28.004 | 29.429 | 29.838 |
| bta-miR-365-5p | 34.477 | 36.270 | 36.208 | 31.658 | 32.394 | 30.757 |
| bta-miR-345-3p | 32.174 | 30.720 | 32.769 | 29.837 | 31.870 | . |
| bta-miR-367 | . | . | . | . | . | . |
| bta-miR-345-5p | . | 32.643 | 33.992 | . | 33.970 | 32.129 |
| bta-miR-369-3p | . | . | . | . | . | . |
| bta-miR-346 | 33.183 | 33.227 | 34.958 | 30.469 | 32.055 | 30.545 |
| bta-miR-369-5p | . | . | 35.106 | 31.816 | . | 34.953 |
| bta-miR-34a | 29.152 | 28.654 | 30.835 | 26.658 | 28.177 | 28.560 |
| bta-miR-370 | 32.842 | 34.995 | 33.266 | . | 32.875 | 33.714 |
| bta-miR-34b | 27.224 | 25.684 | 28.835 | 24.836 | 25.094 | 25.802 |
| bta-miR-371 | 35.613 | . | . | . | 35.956 | . |
| bta-miR-34c | 26.951 | 25.733 | 28.755 | 24.820 | 24.810 | 25.786 |
| bta-miR-374a | 28.828 | 27.692 | 28.416 | 26.132 | 27.980 | 26.844 |
| bta-miR-361 | 26.762 | 26.083 | 28.811 | 25.779 | 25.475 | 28.334 |
| bta-miR-374b | 27.182 | 26.167 | 27.199 | 24.608 | 25.373 | 25.841 |
| bta-miR-362-3p | . | 35.325 | . | 31.076 | 34.849 | 33.103 |
| bta-miR-375 | 26.079 | 25.066 | 27.323 | 23.827 | 24.228 | 25.806 |
| bta-miR-362-5p | 34.126 | 32.550 | 36.970 | 30.984 | 32.807 | 32.890 |
| bta-miR-376a | . | . | . | 31.729 | . | . |
| bta-miR-363 | . | 34.602 | . | 32.925 | . | . |
| bta-miR-376b | . | . | . | . | . | 34.535 |
| bta-miR-376c | . | . | . | . | . | . |
| bta-miR-382 | 31.764 | 31.328 | 32.227 | 30.037 | . | 31.861 |
| bta-miR-376d | . | 35.981 | . | 32.290 | . | 33.333 |
| bta-miR-383 | 35.141 | 33.559 | 34.125 | 30.614 | 36.551 | 33.645 |
| bta-miR-376e | . | . | . | 35.506 | . | . |
| bta-miR-409a | . | . | 35.602 | . | 34.721 | 36.084 |
| bta-miR-377 | . | . | . | . | . | . |
| bta-miR-409b | . | . | . | . | . | . |
| bta-miR-378 | 29.716 | 29.678 | 32.337 | 27.724 | 29.970 | 29.140 |
| bta-miR-410 | . | . | . | 36.175 | 36.913 | . |
| bta-miR-378b | 31.109 | 30.521 | 31.674 | 27.725 | 30.321 | 29.319 |
| bta-miR-411a | 32.670 | . | 33.814 | 29.806 | 32.729 | 34.270 |
| bta-miR-378c | 33.248 | 32.861 | 34.888 | 31.891 | 32.884 | 32.719 |
| bta-miR-411b | 33.491 | 33.963 | 34.665 | . | 33.719 | . |
| bta-miR-378d | 35.327 | 34.162 | 36.272 | 30.841 | 33.998 | 33.688 |
| bta-miR-411c-3p | . | . | . | 33.922 | 36.201 | 36.208 |
| bta-miR-379 | . | . | . | 33.855 | . | . |
| bta-miR-411c-5p | . | . | 35.487 | . | . | . |
| bta-miR-380-3p | . | 35.276 | 35.918 | 32.942 | . | 35.238 |
| bta-miR-412 | . | . | 36.377 | . | . | . |
| bta-miR-380-5p | . | 33.087 | 34.060 | 28.800 | 36.685 | 35.113 |
| bta-miR-421 | 30.452 | 29.271 | 30.517 | 27.551 | 28.945 | 29.641 |
| bta-miR-381 | . | 35.702 | . | 34.884 | . | 36.628 |
| bta-miR-423-3p | 29.915 | 28.741 | 30.677 | 26.757 | 28.148 | 28.612 |
| bta-miR-423-5p | 28.294 | 27.919 | 28.555 | 25.766 | 27.315 | 27.534 |
| bta-miR-449c | 30.713 | 31.839 | 34.718 | 28.273 | 30.803 | . |
| bta-miR-424-3p | 33.331 | 34.016 | 34.597 | 32.846 | 32.637 | 33.877 |
| bta-miR-449d | 31.872 | 31.960 | 32.926 | 31.387 | 31.899 | 31.799 |
| bta-miR-424-5p | 29.527 | 27.918 | 29.336 | 25.522 | 27.321 | 26.397 |
| bta-miR-450a | 32.786 | 31.792 | 35.011 | 30.053 | 31.811 | 31.108 |
| bta-miR-425-3p | 27.744 | 27.306 | . | . | 26.527 | . |
| bta-miR-450b | 32.926 | 30.926 | 33.000 | 29.509 | 30.840 | 30.708 |
| bta-miR-425-5p | 31.107 | 29.744 | 30.491 | 26.684 | 28.892 | 27.918 |
| bta-miR-451 | . | 34.841 | 33.943 | 27.833 | 33.327 | 35.041 |
| bta-miR-429 | 27.329 | 26.222 | 27.231 | 24.016 | 25.701 | 25.428 |
| bta-miR-452 | 36.197 | 33.959 | . | 32.952 | 34.206 | 34.657 |
| bta-miR-431 | . | . | . | 35.435 | 34.715 | 35.705 |
| bta-miR-4523 | 34.641 | 34.109 | 34.321 | 32.581 | . | 34.950 |
| bta-miR-432 | . | . | . | 29.622 | 35.152 | 33.214 |
| bta-miR-453 | 34.943 | 34.959 | 35.731 | 36.961 | . | . |
| bta-miR-433 | 30.100 | 30.733 | 29.874 | 28.333 | 29.818 | 30.138 |
| bta-miR-454 | 35.169 | 32.433 | 34.513 | 29.421 | 31.613 | 31.902 |
| bta-miR-448 | . | . | . | 33.636 | . | . |
| bta-miR-455-3p | 32.869 | 32.845 | 34.081 | 29.746 | 30.594 | 31.688 |
| bta-miR-449a | 26.179 | 26.265 | 29.951 | 23.642 | 26.312 | 25.892 |
| bta-miR-455-5p | 34.807 | 34.390 | 34.633 | 32.295 | 32.891 | 32.928 |
| bta-miR-449b | 27.884 | 28.618 | 31.719 | 25.820 | 28.764 | 27.938 |
| bta-miR-483 | 34.663 | . | . | 33.309 | . | 35.610 |
| bta-miR-484 | 31.893 | 31.384 | 31.942 | 28.437 | 29.867 | 30.123 |
| bta-miR-496 | . | . | . | . | . | . |
| bta-miR-485 | 34.167 | . | 36.107 | 33.699 | . | 35.361 |
| bta-miR-497 | 32.337 | 30.921 | 36.436 | 30.479 | 30.687 | 30.800 |
| bta-miR-486 | . | . | . | . | . | . |
| bta-miR-499 | 34.910 | 33.579 | 33.964 | 32.086 | 33.025 | 31.844 |
| bta-miR-487a | . | . | . | 32.918 | . | . |
| bta-miR-500 | 32.120 | 30.968 | 34.942 | 29.218 | 30.692 | 31.051 |
| bta-miR-487b | . | . | . | 34.417 | . | . |
| bta-miR-502a | 34.631 | 33.885 | . | 34.385 | . | 33.387 |
| bta-miR-488 | 36.865 | . | . | 31.938 | . | . |
| bta-miR-502b | 31.518 | 31.795 | 33.148 | 29.735 | 30.910 | 30.837 |
| bta-miR-489 | 34.079 | 33.899 | 34.796 | 34.504 | 33.862 | 36.445 |
| bta-miR-503-3p | . | . | 33.512 | 29.566 | . | 30.768 |
| bta-miR-490 | 36.296 | . | . | 33.243 | 33.052 | 34.042 |
| bta-miR-503-5p | 34.326 | 34.962 | 36.058 | 31.136 | . | 33.898 |
| bta-miR-491 | 29.872 | 29.780 | 30.421 | 26.703 | 28.529 | 27.953 |
| bta-miR-504 | 35.430 | 33.494 | . | 32.628 | 33.326 | 33.358 |
| bta-miR-493 | 32.599 | 31.695 | 36.937 | 30.625 | 32.213 | 30.678 |
| bta-miR-505 | 29.542 | 27.865 | 30.567 | 26.688 | 27.841 | 28.099 |
| bta-miR-494 | 27.511 | 28.261 | . | . | 28.747 | . |
| bta-miR-532 | 34.468 | 33.097 | 32.882 | 30.119 | 32.966 | 33.977 |
| bta-miR-495 | . | . | . | . | . | . |
| bta-miR-539 | . | . | . | 32.287 | . | . |
| bta-miR-541 | 31.961 | 31.662 | 32.882 | 30.619 | 31.648 | 32.453 |
| bta-miR-582 | . | . | 33.985 | 33.214 | . | . |
| bta-miR-542-5p | 35.302 | 32.747 | 35.118 | 31.868 | 32.857 | 31.734 |
| bta-miR-584 | 33.443 | 33.544 | 35.383 | 31.448 | 33.975 | 31.723 |
| bta-miR-543 | . | 34.369 | . | 33.886 | 35.859 | . |
| bta-miR-592 | 34.365 | 35.598 | . | 31.668 | . | 35.295 |
| bta-miR-544a | . | . | . | 33.700 | . | . |
| bta-miR-599 | . | . | . | . | . | . |
| bta-miR-544b | 34.909 | . | . | 33.965 | 34.675 | . |
| bta-miR-615 | . | . | . | . | . | . |
| bta-miR-545-3p | . | 35.430 | . | 33.971 | . | 34.513 |
| bta-miR-628 | 31.721 | 31.835 | 33.753 | 30.212 | 30.804 | 33.936 |
| bta-miR-545-5p | . | . | . | 33.510 | . | . |
| bta-miR-631 | 19.223 | 19.256 | 19.071 | 19.237 | 19.265 | 19.075 |
| bta-miR-551a | . | . | . | . | . | . |
| bta-miR-652 | 28.512 | 28.080 | 29.746 | 26.711 | 27.682 | 28.167 |
| bta-miR-551b | . | . | . | 34.900 | . | 33.889 |
| bta-miR-653 | . | 34.723 | . | . | . | . |
| bta-miR-562 | . | . | . | . | . | . |
| bta-miR-654 | 33.910 | 32.220 | 33.876 | 32.006 | 33.584 | 31.904 |
| bta-miR-568 | . | . | . | . | . | . |
| bta-miR-655 | . | . | . | 33.406 | . | . |
| bta-miR-574 | 28.024 | 27.497 | 28.995 | 26.279 | 26.988 | 26.593 |
| bta-miR-656 | 33.756 | 32.817 | 34.048 | 31.464 | 33.886 | 33.249 |
| bta-miR-658 | . | . | . | 34.583 | 34.326 | 34.895 |
| bta-miR-758 | . | 34.534 | . | 31.638 | . | . |
| bta-miR-660 | 31.819 | 29.208 | 30.743 | 27.314 | 28.815 | 29.168 |
| bta-miR-759 | . | . | . | . | . | . |
| bta-miR-664a | 33.912 | 33.231 | 32.683 | 30.592 | 33.548 | 31.842 |
| bta-miR-760-3p | 34.218 | 34.880 | . | . | 33.409 | 32.840 |
| bta-miR-664b | 26.094 | 25.398 | 27.231 | 23.974 | 24.735 | 25.743 |
| bta-miR-760-5p | 26.565 | 25.881 | 27.841 | 24.685 | 25.621 | 25.624 |
| bta-miR-665 | 32.160 | 30.975 | 32.706 | 29.245 | 29.722 | 29.718 |
| bta-miR-761 | 36.911 | . | . | 33.737 | 36.423 | 35.403 |
| bta-miR-669 | 29.697 | 29.769 | 31.281 | 28.695 | 29.053 | 29.454 |
| bta-miR-763 | 34.394 | 35.194 | 35.377 | 31.805 | 34.820 | 34.199 |
| bta-miR-670 | . | 36.846 | . | 33.905 | . | 34.161 |
| bta-miR-764 | 34.513 | . | 34.129 | 33.938 | 34.444 | 35.484 |
| bta-miR-671 | 34.371 | 34.105 | . | 33.604 | 32.921 | 34.537 |
| bta-miR-767 | 32.941 | 33.419 | 32.860 | 32.480 | 32.898 | 33.614 |
| bta-miR-677 | 36.510 | 31.822 | 32.867 | 30.685 | 33.067 | 31.787 |
| bta-miR-769 | . | 33.714 | . | 32.303 | 32.945 | 33.869 |
| bta-miR-7 | 29.961 | 28.793 | 30.072 | 27.396 | 28.290 | 29.301 |
| bta-miR-873 | 35.734 | 36.633 | . | 32.270 | 35.653 | . |
| bta-miR-708 | 30.485 | 30.214 | 31.399 | 28.216 | 29.779 | 29.709 |
| bta-miR-874 | . | 30.838 | 33.778 | 28.838 | 30.137 | 30.163 |
| bta-miR-744 | 27.715 | 27.171 | 29.264 | 26.635 | 26.975 | 28.533 |
| bta-miR-875 | . | . | . | 35.117 | . | . |
| bta-miR-876 | . | 36.465 | . | 34.560 | . | . |
| bta-miR-98 | 28.116 | 26.973 | 28.706 | 25.939 | 26.757 | 27.274 |
| bta-miR-877 | . | . | 33.890 | . | 31.278 | . |
| bta-miR-99a-3p | 32.901 | 33.773 | . | 29.722 | 31.884 | 33.014 |
| bta-miR-885 | 26.808 | 25.971 | 26.752 | 24.202 | 25.384 | 25.746 |
| bta-miR-99a-5p | 28.859 | 27.684 | 28.020 | 23.985 | 26.406 | 24.944 |
| bta-miR-9-3p | 35.036 | 32.956 | 35.582 | 30.523 | 33.878 | 35.116 |
| bta-miR-99b | 22.763 | 22.870 | 22.371 | 22.761 | 22.832 | 22.684 |
| bta-miR-9-5p | 25.929 | 24.742 | 27.609 | 24.751 | 24.480 | 27.300 |
| bta-miR-1179 | . | 36.693 | . | 33.350 | . | . |
| bta-miR-92a | 26.815 | 25.977 | 26.838 | 24.499 | 25.677 | 25.697 |
| bta-miR-1185 | . | . | . | . | . | . |
| bta-miR-92b | 25.326 | 24.827 | 25.579 | 22.878 | 24.381 | 24.509 |
| bta-miR-1193 | . | . | . | 34.182 | 36.734 | . |
| bta-miR-93 | 28.927 | 28.114 | 30.276 | 26.477 | 27.741 | 28.221 |
| bta-miR-1197 | . | 35.079 | . | . | . | . |
| bta-miR-935 | . | 34.270 | . | 30.976 | 33.736 | 32.567 |
| bta-miR-122 | . | . | . | 36.863 | . | . |
| bta-miR-940 | 27.742 | 26.434 | 28.823 | 25.770 | 26.670 | 26.424 |
| bta-miR-1224 | 29.751 | 30.847 | . | . | . | . |
| bta-miR-95 | 30.819 | 31.715 | 30.379 | 29.644 | 31.645 | 31.885 |
| bta-miR-1225-3p | 28.949 | 27.791 | 30.173 | 26.998 | 27.833 | 27.817 |
| bta-miR-96 | . | 34.891 | . | 31.115 | 35.032 | 33.443 |
| bta-miR-1246 | . | . | . | . | . | . |
| bta-miR-1247-3p | 31.869 | 31.843 | 34.907 | . | 31.622 | 31.877 |
| bta-miR-1296 | 33.883 | 32.446 | 34.593 | 30.244 | 31.743 | . |
| bta-miR-1247-5p | 31.906 | 30.836 | 33.943 | 29.752 | 30.272 | 30.031 |
| bta-miR-1298 | . | . | . | . | . | 35.924 |
| bta-miR-1248 | 32.811 | 32.326 | 32.897 | 30.226 | 32.785 | 30.511 |
| bta-miR-1301 | 34.920 | 35.727 | . | . | 34.066 | . |
| bta-miR-1249 | 29.913 | 28.696 | 29.621 | 27.829 | 28.740 | 29.144 |
| bta-miR-1306 | 29.660 | 29.396 | 30.292 | 27.678 | 28.830 | 28.776 |
| bta-miR-1260b | 24.173 | 23.208 | 24.032 | 22.220 | 23.695 | 23.673 |
| bta-miR-1307 | 29.177 | 28.654 | . | . | 27.997 | 28.237 |
| bta-miR-1271 | 33.649 | 34.548 | . | 31.712 | 35.825 | 32.918 |
| bta-miR-1343-3p | 31.949 | 31.131 | 30.335 | 28.578 | 30.118 | 29.798 |
| bta-miR-1277 | . | . | . | . | . | . |
| bta-miR-1343-5p | 30.119 | 29.557 | 30.922 | 28.653 | 29.330 | 28.967 |
| bta-miR-1281 | 31.735 | 30.904 | . | 29.626 | 30.909 | 30.356 |
| bta-miR-1388-3p | 32.924 | 30.932 | 32.521 | 28.377 | 32.227 | 31.557 |
| bta-miR-1282 | . | . | . | . | . | . |
| RNT43 snoRNA | 28.618 | 27.985 | 26.365 | 24.704 | 27.720 | 24.754 |
| bta-miR-1284 | . | 34.943 | . | . | 33.711 | 34.147 |
| Hm/Ms/Rt T1 snRNA | 20.971 | 19.574 | 19.682 | 16.686 | 19.004 | 17.789 |
| bta-miR-1287 | . | 34.347 | . | 33.926 | . | 34.939 |
| bta-miR-99b | 22.604 | 22.688 | 22.760 | 22.483 | 22.798 | 22.774 |
| bta-miR-1291 | 34.557 | 32.943 | . | . | 35.021 | 32.946 |
| Negative control | . | . | . | . | . | . |
| ^1^Body energy reserve: MBER: Cows with moderated body energy reserve; HBER: Cows with high body energy reserve. | | | | | | |
