# Supplementary material for: The bovine oviductal environment and composition are negatively affected by elevated body energy reserves
Source: PLoS One. 2025 Jun 23;20(6):e0326138. doi: 10.1371/journal.pone.0326138 (PMC12184905; doi:10.1371/journal.pone.0326138)
Supplement: S9 Table — (DOCX) [file pone.0326138.s012.docx]

| **Supplementary Table 9.** Raw cycle threshold levels of the 383 miRNAs profile in isthmic luminal epithelial cells (IST-Cell) of cows with different body energy reserve. | | | | | | |
| --- | --- | --- | --- | --- | --- | --- |
| **miRNA** | **Body energy reserve^1^** | | | | | |
|  | **MBER** | | | **HBER** | | |
|  | **1** | **2** | **3** | **1** | **2** | **3** |
| bta-let-7a-3p | 25.990 | 25.861 | 26.589 | 26.341 | 25.703 | 25.818 |
| bta-miR-103 | 26.724 | 26.018 | 26.196 | 27.613 | 25.873 | 26.133 |
| bta-let-7a-5p | 20.054 | 20.193 | 20.655 | 19.635 | 19.990 | 19.510 |
| bta-miR-105a | . | 34.467 | . | 34.991 | 36.061 | . |
| bta-let-7b | 21.352 | 21.414 | 21.781 | 20.803 | 21.046 | 20.781 |
| bta-miR-105b | 32.580 | . | 33.433 | 34.999 | 33.052 | 32.271 |
| bta-let-7c | 20.133 | 20.319 | 20.795 | 19.782 | 20.108 | 19.663 |
| bta-miR-106a | 26.833 | 26.105 | 26.339 | 27.831 | 25.936 | 25.912 |
| bta-let-7d | 21.181 | 21.247 | 21.813 | 20.775 | 21.361 | 20.730 |
| bta-miR-106b | 28.682 | 28.306 | 27.710 | 30.484 | 27.783 | 27.955 |
| bta-let-7e | 19.902 | 19.786 | 20.549 | 19.455 | 19.799 | 19.273 |
| bta-miR-107 | 30.803 | 29.818 | 29.737 | 31.490 | 30.721 | 29.872 |
| bta-let-7f | 21.254 | 21.633 | . | 21.123 | 21.488 | 20.720 |
| bta-miR-10a | 23.713 | 24.278 | 25.267 | 24.722 | 23.856 | 24.276 |
| bta-let-7g | 22.611 | 22.397 | 23.187 | 23.183 | 22.447 | 22.181 |
| bta-miR-10b | 24.178 | 24.511 | 25.279 | 25.391 | 24.581 | 24.701 |
| bta-let-7i | 25.512 | 24.768 | 24.967 | 25.712 | 24.973 | 24.744 |
| bta-miR-122 | . | 35.070 | . | 35.871 | . | 35.229 |
| bta-miR-1 | 29.811 | 29.135 | 27.477 | 30.174 | 29.256 | 29.464 |
| bta-miR-124a | 35.880 | . | . | . | 35.866 | . |
| bta-miR-100 | 25.112 | 24.993 | 25.732 | 26.396 | 24.593 | 24.665 |
| bta-miR-124b | . | 34.278 | 34.685 | . | 35.929 | . |
| bta-miR-101 | 28.967 | 28.035 | 28.578 | 29.799 | 27.937 | 28.311 |
| bta-miR-125a | 22.391 | 22.390 | 22.741 | 22.934 | 22.147 | 22.090 |
| bta-miR-125b | 22.522 | 21.990 | 21.808 | 23.142 | 21.649 | 21.570 |
| bta-miR-133b | . | 34.838 | 36.832 | . | . | . |
| bta-miR-126-3p | . | . | 30.562 | 30.625 | 28.322 | 29.888 |
| bta-miR-133c | . | 35.158 | . | . | 35.355 | . |
| bta-miR-126-5p | 27.834 | 27.630 | . | 28.636 | 27.074 | 28.683 |
| bta-miR-134 | . | 34.625 | . | . | . | 36.640 |
| bta-miR-127 | 30.601 | 30.003 | 30.395 | 31.418 | 30.730 | 31.724 |
| bta-miR-135a | 22.708 | 21.910 | 22.071 | 22.831 | 21.816 | 21.765 |
| bta-miR-128 | 26.316 | 25.826 | 26.839 | 27.352 | 26.426 | 25.833 |
| bta-miR-135b | 23.775 | 23.217 | 23.354 | 24.275 | 23.511 | 22.952 |
| bta-miR-129 | . | 31.492 | 32.714 | . | 31.769 | 31.887 |
| bta-miR-136 | . | 35.791 | 34.875 | . | . | 34.935 |
| bta-miR-129-3p | 33.941 | 31.524 | 32.862 | 32.715 | 31.312 | 31.527 |
| bta-miR-137 | . | . | . | . | . | . |
| bta-miR-129-5p | 32.118 | 31.390 | 32.647 | . | 31.643 | 32.839 |
| bta-miR-138 | . | . | . | 33.559 | 32.482 | 32.917 |
| bta-miR-130a | 29.769 | 30.011 | 30.061 | 31.151 | 30.816 | 31.582 |
| bta-miR-139 | 29.032 | 28.480 | 29.112 | 28.833 | 28.541 | 27.646 |
| bta-miR-130b | 23.399 | 22.786 | 23.269 | 23.960 | 23.283 | 23.389 |
| bta-miR-140 | 28.801 | 28.318 | 28.471 | 29.566 | 27.936 | 28.587 |
| bta-miR-132 | 29.747 | 29.887 | 31.131 | 30.790 | 30.798 | 29.491 |
| bta-miR-141 | 27.789 | 26.559 | 26.807 | 28.779 | 25.961 | 26.413 |
| bta-miR-133a | 32.859 | 33.287 | 31.095 | 35.314 | 32.928 | 33.836 |
| bta-miR-142-3p | . | 32.537 | . | 33.500 | 34.854 | 34.756 |
| bta-miR-142-5p | 33.958 | 33.910 | 32.668 | 35.567 | 33.270 | 33.641 |
| bta-miR-151-3p | 26.746 | 26.129 | 26.231 | 26.746 | 25.988 | 25.942 |
| bta-miR-143 | 26.369 | 25.403 | 25.827 | 26.520 | 25.808 | 25.470 |
| bta-miR-151-5p | 24.455 | 23.801 | 24.064 | 24.525 | 23.700 | 23.801 |
| bta-miR-144 | . | . | . | . | . | . |
| bta-miR-152 | 31.422 | 31.677 | 31.078 | 32.252 | 31.910 | 30.263 |
| bta-miR-145 | 28.787 | 28.514 | 30.487 | 29.813 | 27.976 | 28.047 |
| bta-miR-153 | 29.636 | 28.890 | 29.667 | 30.742 | 29.742 | 28.635 |
| bta-miR-146a | 31.897 | 33.328 | 31.348 | 32.190 | 31.530 | 36.107 |
| bta-miR-154a | 32.897 | 32.670 | 35.049 | 34.938 | 32.962 | 32.449 |
| bta-miR-146b | 31.151 | 32.254 | 33.893 | . | 31.903 | 31.961 |
| bta-miR-154b | 29.662 | 29.420 | 28.640 | 30.762 | 30.136 | 29.723 |
| bta-miR-147 | 32.934 | 33.209 | 31.827 | . | 31.114 | 32.276 |
| bta-miR-154c | 32.997 | 34.613 | 34.129 | 36.239 | 34.285 | 35.574 |
| bta-miR-148a | 20.977 | 20.355 | 20.486 | 21.057 | 19.956 | 19.812 |
| bta-miR-155 | 29.424 | 29.864 | 31.256 | 29.816 | 31.147 | 30.219 |
| bta-miR-148b | 21.781 | 20.857 | 20.812 | 21.716 | 20.712 | 20.461 |
| bta-miR-15a | 28.822 | 28.348 | 27.878 | 30.287 | 28.190 | 27.929 |
| bta-miR-149-3p | 29.403 | 30.068 | 30.456 | 31.537 | 30.320 | 30.570 |
| bta-miR-15b | 24.793 | 25.190 | 25.308 | 25.231 | 25.120 | 24.534 |
| bta-miR-149-5p | 31.732 | 31.630 | 32.784 | 32.930 | 31.090 | 30.860 |
| bta-miR-16a | 24.768 | 24.352 | 24.053 | 25.689 | 24.059 | 24.015 |
| bta-miR-150 | 26.697 | 27.785 | 28.357 | 28.519 | 27.803 | 27.922 |
| bta-miR-16b | 24.591 | 24.090 | 23.896 | 25.461 | 23.991 | 23.827 |
| bta-miR-17-3p | 33.997 | 32.837 | 32.127 | 33.178 | 31.567 | 33.419 |
| bta-miR-188 | 32.812 | 30.537 | . | 32.338 | . | 31.012 |
| bta-miR-17-5p | 29.763 | 29.463 | 29.562 | 30.436 | 29.432 | 29.293 |
| bta-miR-18a | 31.830 | 31.882 | 32.154 | . | 32.829 | 31.751 |
| bta-miR-181a | 31.571 | 32.116 | 31.819 | 31.856 | 31.437 | 31.591 |
| bta-miR-18b | 33.829 | 33.146 | 33.759 | 35.029 | 33.072 | 33.348 |
| bta-miR-181b | 28.682 | 28.680 | 29.311 | 29.347 | 28.646 | 28.478 |
| bta-miR-190a | 30.571 | 30.163 | 29.826 | 32.503 | 30.665 | 30.617 |
| bta-miR-181c | 32.841 | 33.750 | 31.649 | 34.091 | 33.340 | 32.834 |
| bta-miR-190b | 25.972 | 25.555 | 25.297 | 25.957 | 26.399 | 25.736 |
| bta-miR-181d | 27.827 | 27.797 | 28.438 | 28.071 | 27.699 | 27.637 |
| bta-miR-191 | 23.644 | 22.729 | 23.441 | 23.829 | 23.605 | 23.308 |
| bta-miR-182 | 27.768 | 28.130 | 28.567 | 28.387 | 28.577 | 27.671 |
| bta-miR-192 | 30.493 | 30.428 | 30.275 | 31.066 | 29.465 | 29.829 |
| bta-miR-183 | 28.737 | 29.404 | 29.197 | 28.785 | 29.228 | 29.029 |
| bta-miR-193a | . | . | 38.254 | . | . | . |
| bta-miR-184 | . | 35.337 | 35.518 | 36.988 | 36.418 | 33.993 |
| bta-miR-193a-3p | 35.711 | . | 34.337 | . | 35.308 | . |
| bta-miR-185 | 28.775 | 28.284 | 27.876 | 29.727 | 27.829 | 28.250 |
| bta-miR-193a-5p | 29.145 | 28.684 | 29.447 | 28.735 | 29.503 | 29.489 |
| bta-miR-186 | 26.642 | 25.967 | 26.040 | 27.744 | 25.662 | 25.686 |
| bta-miR-193b | 34.819 | 35.330 | 33.387 | . | 32.593 | 33.618 |
| bta-miR-187 | 31.205 | 30.495 | . | . | 30.706 | 30.785 |
| bta-miR-194 | 28.759 | 27.815 | 28.081 | 29.731 | 27.319 | 28.075 |
| bta-miR-195 | 25.594 | 25.279 | 24.869 | 25.722 | 24.817 | 24.692 |
| bta-miR-200c | 20.501 | 20.443 | 20.744 | 20.705 | 20.473 | 20.067 |
| bta-miR-196a | 31.422 | 35.741 | . | 36.917 | 30.887 | 35.398 |
| bta-miR-202 | 30.811 | 29.627 | 29.758 | 30.603 | 29.359 | 30.332 |
| bta-miR-196b | 30.727 | 32.847 | 32.778 | 32.768 | 31.535 | 31.924 |
| bta-miR-204 | 24.579 | 23.980 | 24.562 | 24.727 | 24.033 | 24.696 |
| bta-miR-197 | 26.459 | 25.760 | 26.813 | 26.548 | 26.008 | 25.894 |
| bta-miR-205 | 28.544 | 28.705 | 29.806 | 30.090 | 26.039 | 27.644 |
| bta-miR-199a-3p | 27.767 | 27.807 | 30.827 | 28.757 | 26.779 | 28.680 |
| bta-miR-206 | 34.021 | 33.390 | 35.000 | 35.028 | 34.011 | 34.760 |
| bta-miR-199a-5p | 34.673 | 33.222 | 35.728 | 35.048 | 31.313 | 34.105 |
| bta-miR-208a | 35.883 | 36.087 | 36.782 | 34.993 | . | 36.831 |
| bta-miR-199b | 32.751 | 32.885 | 34.959 | . | 32.105 | . |
| bta-miR-208b | 35.211 | 35.733 | 36.768 | 34.946 | . | 35.517 |
| bta-miR-199c | 27.312 | 27.054 | 30.146 | 27.964 | 26.123 | 27.692 |
| bta-miR-20a | 26.685 | 26.402 | 26.455 | 27.740 | 26.230 | 25.995 |
| bta-miR-19a | 27.609 | 27.174 | 27.461 | 29.736 | 27.330 | 27.044 |
| bta-miR-20b | 28.566 | 27.758 | 28.079 | 29.751 | 27.807 | 27.784 |
| bta-miR-19b | 27.692 | 27.028 | 27.309 | 29.480 | 27.007 | 26.889 |
| bta-miR-21-3p | 35.028 | 32.738 | 32.819 | 34.177 | 33.885 | 35.258 |
| bta-miR-200a | 27.039 | 26.876 | 26.993 | 28.304 | 26.768 | 26.839 |
| bta-miR-21-5p | 28.081 | 28.447 | 28.006 | 28.767 | 27.564 | 27.780 |
| bta-miR-200b | 18.582 | 18.632 | 18.853 | 18.991 | 18.490 | 18.153 |
| bta-miR-210 | 29.771 | 28.971 | 29.124 | 30.730 | 28.814 | 28.735 |
| bta-miR-211 | 25.427 | 24.672 | 25.429 | 24.798 | 23.983 | 25.469 |
| bta-miR-22-5p | 28.588 | 28.538 | 28.721 | 28.969 | 28.369 | 27.691 |
| bta-miR-212 | . | . | 37.477 | . | 34.772 | . |
| bta-miR-221 | 27.328 | 27.425 | 25.667 | 26.851 | 27.022 | 26.780 |
| bta-miR-214 | . | . | . | 33.951 | . | 31.902 |
| bta-miR-222 | 27.728 | 27.388 | 25.189 | 26.812 | 26.826 | 26.706 |
| bta-miR-215 | 29.815 | 29.406 | 29.610 | 30.534 | 29.409 | 29.594 |
| bta-miR-223 | 30.830 | 31.064 | 30.393 | 30.809 | 29.853 | 30.616 |
| bta-miR-216a | 32.851 | 31.677 | 32.106 | 35.342 | . | . |
| bta-miR-224 | 27.679 | 27.355 | 28.305 | 27.789 | 28.429 | 27.537 |
| bta-miR-216b | . | . | 32.942 | 35.052 | 33.115 | 32.904 |
| bta-miR-23a | 20.746 | 20.722 | 21.084 | 21.153 | 20.823 | 20.559 |
| bta-miR-217 | . | . | . | . | . | . |
| bta-miR-23b-3p | 23.332 | 23.424 | 23.832 | 23.699 | 23.531 | 23.360 |
| bta-miR-218 | 30.233 | 29.785 | 29.589 | 30.759 | 30.649 | 29.908 |
| bta-miR-23b-5p | 32.865 | 33.608 | 32.900 | 33.499 | 32.751 | 33.202 |
| bta-miR-219 | 33.090 | . | 32.608 | 33.931 | 31.870 | . |
| bta-miR-24 | . | . | . | . | . | . |
| bta-miR-219-3p | 29.201 | 29.365 | 29.720 | 30.655 | 29.760 | 30.449 |
| bta-miR-24-3p | 23.799 | 23.313 | 23.607 | 24.690 | 23.216 | 23.386 |
| bta-miR-219-5p | . | . | . | . | . | . |
| bta-miR-25 | 23.740 | 23.708 | 24.152 | 24.413 | 23.837 | 23.631 |
| bta-miR-22-3p | 2.767 | 2.434 | 2.516 | 2.776 | 2.694 | 2.685 |
| bta-miR-26a | 20.554 | 19.983 | 20.324 | 20.860 | 19.919 | 19.960 |
| bta-miR-26b | 21.233 | 20.888 | 21.445 | 21.231 | 20.886 | 20.798 |
| bta-miR-29d-3p | 22.933 | 22.134 | 21.997 | 23.191 | 22.020 | 22.137 |
| bta-miR-26c | . | . | . | . | . | . |
| bta-miR-29d-5p | 30.284 | 28.793 | 28.593 | 29.774 | 28.666 | 28.969 |
| bta-miR-27a-3p | 24.725 | 23.654 | 24.074 | 25.961 | 24.097 | 24.358 |
| bta-miR-29e | 33.631 | 32.911 | 32.107 | 35.478 | 32.102 | 31.861 |
| bta-miR-27a-5p | 36.255 | 32.803 | 31.895 | 33.268 | 32.904 | 33.261 |
| bta-miR-301a | . | 33.974 | . | . | . | . |
| bta-miR-27b | 24.701 | 24.343 | 25.161 | 25.377 | 24.082 | 24.236 |
| bta-miR-301b | . | . | . | . | . | . |
| bta-miR-28 | 29.601 | 28.725 | 29.577 | 29.891 | 28.470 | 28.744 |
| bta-miR-302a | . | . | 34.830 | . | . | 34.350 |
| bta-miR-296-3p | 29.230 | 29.246 | 30.806 | 29.750 | 29.823 | 29.545 |
| bta-miR-302b | . | . | . | 34.768 | . | . |
| bta-miR-296-5p | 31.645 | 30.714 | 31.100 | 31.797 | 30.479 | 30.354 |
| bta-miR-302c | 33.869 | 33.978 | 37.498 | . | 34.691 | 36.281 |
| bta-miR-299 | . | . | . | . | 35.637 | . |
| bta-miR-302d | . | 33.975 | . | . | . | . |
| bta-miR-29a | 20.487 | 20.229 | 20.468 | 20.997 | 19.787 | 20.067 |
| bta-miR-3064 | 35.491 | 32.164 | 31.837 | 32.936 | . | 33.013 |
| bta-miR-29b | 31.246 | 29.701 | 30.208 | 31.044 | 29.756 | 29.846 |
| bta-miR-30a-5p | 27.166 | 26.360 | 26.508 | 27.772 | 26.039 | 26.256 |
| bta-miR-29c | 20.322 | 19.984 | 20.390 | 20.794 | 19.831 | 20.218 |
| bta-miR-30b-3p | 31.221 | 30.995 | 30.820 | 29.827 | 31.121 | 30.486 |
| bta-miR-30b-5p | 24.992 | 24.383 | 24.128 | 25.810 | 23.809 | 24.098 |
| bta-miR-328 | 30.149 | 27.465 | 28.974 | 29.392 | 27.824 | 27.075 |
| bta-miR-30c | 23.710 | 23.360 | 23.275 | 24.381 | 22.941 | 23.220 |
| bta-miR-329a | . | . | . | . | . | . |
| bta-miR-30d | 27.272 | 26.517 | 26.135 | 27.784 | 26.072 | 26.298 |
| bta-miR-329b | . | 36.088 | . | 35.783 | . | . |
| bta-miR-30e-5p | 27.110 | 26.557 | 26.214 | 28.085 | 26.291 | 26.141 |
| bta-miR-330 | 34.091 | 32.715 | 32.878 | 35.654 | 32.661 | 33.965 |
| bta-miR-30f | 25.586 | 25.121 | 24.813 | 26.377 | 24.686 | 24.939 |
| bta-miR-331-3p | 29.742 | 29.677 | 29.418 | 28.571 | 28.797 | 29.374 |
| bta-miR-31 | 23.978 | 23.742 | 23.974 | 25.184 | 23.659 | 23.819 |
| bta-miR-331-5p | 29.499 | 29.412 | 29.667 | 30.337 | 29.745 | 29.762 |
| bta-miR-32 | . | . | . | . | 34.551 | . |
| bta-miR-335 | 29.794 | 30.371 | 28.748 | 33.346 | 28.599 | 29.665 |
| bta-miR-320a | 25.495 | 25.618 | 25.841 | 25.421 | 25.828 | 25.546 |
| bta-miR-338 | 34.167 | 31.586 | 30.734 | 33.193 | 30.916 | 31.468 |
| bta-miR-320b | 33.848 | 33.127 | . | 34.830 | 33.870 | 35.308 |
| bta-miR-339a | 27.925 | 27.575 | 27.663 | 28.699 | 27.478 | 27.712 |
| bta-miR-323 | 15.702 | 15.593 | 15.838 | 16.003 | 16.430 | 16.207 |
| bta-miR-339b | 27.712 | 27.064 | 26.782 | 28.481 | 26.723 | 27.177 |
| bta-miR-324 | 30.804 | 30.081 | 31.366 | 31.581 | 29.989 | 30.513 |
| bta-miR-33a | 35.915 | . | 33.465 | . | 34.320 | 34.877 |
| bta-miR-326 | 33.114 | 31.860 | 35.137 | 32.894 | 32.086 | 32.793 |
| bta-miR-33b | 33.260 | 32.866 | 34.022 | 34.431 | 35.989 | 35.362 |
| bta-miR-340 | 31.070 | 31.267 | 30.927 | 30.985 | 31.744 | 30.278 |
| bta-miR-365-3p | 25.993 | 25.262 | 26.690 | 26.004 | 25.213 | 25.110 |
| bta-miR-342 | 28.199 | 27.831 | 28.649 | 29.246 | 28.100 | 28.115 |
| bta-miR-365-5p | 35.622 | 34.275 | 34.611 | 33.113 | 32.696 | 32.748 |
| bta-miR-345-3p | 30.347 | 30.020 | 30.737 | 30.265 | 30.184 | 29.917 |
| bta-miR-367 | . | . | . | . | . | . |
| bta-miR-345-5p | 31.454 | 31.156 | 31.149 | 32.700 | 31.030 | . |
| bta-miR-369-3p | 32.339 | 33.222 | 34.349 | 33.224 | 33.562 | 33.956 |
| bta-miR-346 | 31.775 | 30.769 | . | 31.432 | 30.926 | 30.835 |
| bta-miR-369-5p | . | . | . | 36.024 | 33.577 | . |
| bta-miR-34a | 26.333 | 25.960 | 26.827 | 27.825 | 26.278 | 26.440 |
| bta-miR-370 | 35.370 | 34.517 | 33.181 | . | . | 32.552 |
| bta-miR-34b | 25.998 | 25.238 | 24.859 | 27.363 | 24.793 | 25.299 |
| bta-miR-371 | 35.132 | 34.350 | 34.222 | . | 34.897 | 36.526 |
| bta-miR-34c | 25.931 | 25.240 | 25.056 | 27.066 | 24.709 | 25.078 |
| bta-miR-374a | 26.394 | 26.357 | 26.343 | 27.480 | 25.949 | 25.767 |
| bta-miR-361 | 26.310 | 26.489 | 26.723 | 24.800 | 26.384 | 26.250 |
| bta-miR-374b | 24.766 | 24.822 | 24.815 | 25.379 | 24.616 | 24.493 |
| bta-miR-362-3p | 32.906 | 31.757 | 31.975 | . | 31.573 | 31.954 |
| bta-miR-375 | 24.371 | 23.595 | 24.394 | 23.761 | 23.494 | 23.824 |
| bta-miR-362-5p | 32.287 | 32.834 | 31.645 | 32.821 | 32.085 | 31.777 |
| bta-miR-376a | . | . | . | . | . | . |
| bta-miR-363 | 36.142 | 34.933 | . | . | . | 33.916 |
| bta-miR-376b | . | . | . | . | . | . |
| bta-miR-376c | . | 32.851 | . | . | 33.998 | 32.935 |
| bta-miR-382 | . | 28.784 | 28.767 | 30.624 | . | 29.300 |
| bta-miR-376d | 34.788 | . | . | . | 36.072 | . |
| bta-miR-383 | . | 35.938 | 33.396 | 34.511 | 33.922 | 33.280 |
| bta-miR-376e | . | . | . | 34.834 | . | . |
| bta-miR-409a | 35.315 | . | 34.646 | 36.539 | 34.408 | 34.664 |
| bta-miR-377 | . | . | . | . | . | . |
| bta-miR-409b | . | 35.525 | . | . | . | . |
| bta-miR-378 | 28.750 | 28.532 | 29.109 | 29.337 | 28.780 | 28.606 |
| bta-miR-410 | 35.344 | . | . | . | . | . |
| bta-miR-378b | 28.803 | 28.560 | 28.791 | 29.819 | 29.485 | 28.609 |
| bta-miR-411a | . | 30.751 | 31.801 | 33.815 | . | 32.860 |
| bta-miR-378c | 31.706 | 30.793 | 31.594 | 31.950 | 32.539 | 31.022 |
| bta-miR-411b | . | 32.556 | 32.881 | . | . | . |
| bta-miR-378d | 32.564 | 31.948 | 32.356 | 34.361 | 33.860 | 33.029 |
| bta-miR-411c-3p | . | . | 34.993 | . | . | 33.966 |
| bta-miR-379 | . | . | 32.839 | . | . | . |
| bta-miR-411c-5p | . | . | . | 34.374 | 35.145 | . |
| bta-miR-380-3p | 34.276 | 33.931 | . | 35.458 | . | . |
| bta-miR-412 | . | 34.923 | 33.826 | . | 34.509 | . |
| bta-miR-380-5p | 36.275 | . | . | . | 32.738 | 32.895 |
| bta-miR-421 | 26.607 | 25.858 | 27.215 | 27.343 | 28.022 | 27.435 |
| bta-miR-381 | 36.259 | 32.912 | 35.137 | . | 35.840 | 34.135 |
| bta-miR-423-3p | 27.148 | 26.889 | 27.532 | 27.930 | 26.973 | 26.773 |
| bta-miR-423-5p | 25.827 | 25.799 | 26.914 | 26.556 | 26.413 | 25.780 |
| bta-miR-449c | 30.707 | 29.816 | 32.139 | 31.688 | 32.102 | 30.985 |
| bta-miR-424-3p | 32.791 | 31.797 | 31.620 | 33.981 | 33.121 | 31.907 |
| bta-miR-449d | 32.533 | 31.884 | 32.728 | 32.930 | 31.204 | 32.755 |
| bta-miR-424-5p | 27.536 | 27.461 | 25.749 | 28.142 | 27.487 | 26.202 |
| bta-miR-450a | 30.779 | 31.632 | 30.078 | 31.893 | 31.625 | 30.699 |
| bta-miR-425-3p | . | . | . | . | . | . |
| bta-miR-450b | 31.279 | 32.286 | 29.888 | 30.616 | 31.869 | 30.706 |
| bta-miR-425-5p | 28.296 | 27.667 | 27.565 | 28.988 | 27.546 | 27.470 |
| bta-miR-451 | 30.730 | 30.405 | 34.523 | 30.845 | 29.676 | 29.643 |
| bta-miR-429 | 24.534 | 24.113 | 24.150 | 25.970 | 24.059 | 23.773 |
| bta-miR-452 | 33.969 | 32.186 | 33.023 | . | 33.198 | 32.888 |
| bta-miR-431 | . | 33.046 | 35.232 | 35.116 | 34.142 | 35.546 |
| bta-miR-4523 | . | . | . | . | 33.793 | 34.745 |
| bta-miR-432 | 35.416 | 33.921 | . | . | . | . |
| bta-miR-453 | 33.014 | 32.799 | 33.392 | 33.917 | 33.769 | 33.506 |
| bta-miR-433 | 28.532 | 28.101 | 28.243 | 29.782 | 29.504 | 29.627 |
| bta-miR-454 | 32.997 | 30.670 | 31.180 | 30.661 | 31.695 | 30.763 |
| bta-miR-448 | . | 36.350 | . | . | . | . |
| bta-miR-455-3p | 29.714 | 29.702 | 30.304 | 30.887 | 29.260 | 30.243 |
| bta-miR-449a | 24.799 | 24.320 | 25.823 | 25.713 | 25.717 | 25.083 |
| bta-miR-455-5p | 31.760 | 31.852 | 31.803 | 34.153 | 31.154 | 33.793 |
| bta-miR-449b | 26.825 | 26.582 | 28.253 | 27.694 | 28.216 | 27.572 |
| bta-miR-483 | . | 32.801 | . | . | . | . |
| bta-miR-484 | 29.356 | 28.738 | 29.210 | 29.820 | 28.778 | 29.280 |
| bta-miR-496 | . | 36.478 | 34.596 | . | . | . |
| bta-miR-485 | 33.852 | 32.851 | 32.795 | 33.981 | 35.433 | 33.893 |
| bta-miR-497 | 29.938 | 29.683 | 29.752 | 30.581 | 30.639 | 30.088 |
| bta-miR-486 | 28.980 | 28.710 | 29.396 | 30.133 | 29.236 | 29.703 |
| bta-miR-499 | 31.895 | 29.853 | 30.470 | 31.010 | 29.812 | 30.434 |
| bta-miR-487a | 34.422 | 35.550 | . | . | . | . |
| bta-miR-500 | 30.692 | 29.784 | 30.081 | 29.782 | 29.473 | 29.692 |
| bta-miR-487b | . | . | . | . | . | . |
| bta-miR-502a | 33.676 | 34.963 | 33.010 | 32.913 | 34.999 | 34.381 |
| bta-miR-488 | . | 35.573 | . | 35.732 | . | 33.887 |
| bta-miR-502b | 29.846 | 30.272 | 30.735 | 30.376 | 30.154 | 29.662 |
| bta-miR-489 | 30.605 | 30.611 | 29.806 | 32.207 | 32.296 | 32.508 |
| bta-miR-503-3p | 31.243 | 30.365 | 31.709 | 31.997 | 31.608 | 31.643 |
| bta-miR-490 | 34.570 | 31.783 | . | 35.204 | 33.783 | . |
| bta-miR-503-5p | . | . | 33.469 | . | 33.541 | 33.919 |
| bta-miR-491 | 28.541 | 28.356 | 28.775 | 27.700 | 28.446 | 27.747 |
| bta-miR-504 | 33.003 | 32.431 | . | 33.933 | 32.048 | 31.855 |
| bta-miR-493 | 32.533 | 31.251 | 32.838 | 34.317 | 30.830 | 32.128 |
| bta-miR-505 | 27.670 | 26.743 | 26.768 | . | . | 27.254 |
| bta-miR-494 | 27.714 | 26.755 | 27.798 | 28.638 | 27.719 | 27.812 |
| bta-miR-532 | 32.071 | 31.212 | 31.882 | 33.137 | 30.681 | 29.974 |
| bta-miR-495 | 32.878 | 33.162 | 33.613 | 34.825 | 32.907 | 34.190 |
| bta-miR-539 | 35.325 | . | . | . | . | . |
| bta-miR-541 | 30.019 | 29.753 | . | 30.828 | 30.784 | 30.261 |
| bta-miR-582 | . | . | . | . | 36.438 | . |
| bta-miR-542-5p | 33.831 | 34.821 | 32.893 | 35.011 | . | 33.812 |
| bta-miR-584 | 32.322 | 31.530 | 32.861 | 33.369 | 33.309 | 34.111 |
| bta-miR-543 | 34.776 | 33.831 | . | . | 34.583 | 35.466 |
| bta-miR-592 | 31.862 | 30.764 | . | 32.311 | 30.472 | 31.697 |
| bta-miR-544a | 36.307 | . | 34.958 | . | 34.244 | . |
| bta-miR-599 | . | . | . | . | . | . |
| bta-miR-544b | . | . | . | . | . | . |
| bta-miR-615 | . | . | . | . | . | . |
| bta-miR-545-3p | 36.064 | 33.731 | 35.041 | 33.935 | 32.219 | . |
| bta-miR-628 | 31.828 | 31.648 | 31.900 | 32.458 | 31.765 | 31.885 |
| bta-miR-545-5p | . | . | . | 34.175 | . | 34.181 |
| bta-miR-631 | 18.456 | 18.015 | 18.360 | 18.707 | 18.774 | 18.645 |
| bta-miR-551a | 37.694 | 38.295 | 36.610 | 40.579 | 38.198 | 40.428 |
| bta-miR-652 | 27.675 | 26.913 | 26.784 | 27.399 | 26.963 | 27.096 |
| bta-miR-551b | 34.789 | 33.056 | . | . | 33.278 | 34.586 |
| bta-miR-653 | . | . | 44.541 | . | 40.601 | 42.456 |
| bta-miR-562 | . | . | . | . | . | . |
| bta-miR-654 | 31.833 | 31.967 | 32.269 | 31.795 | 33.098 | 33.863 |
| bta-miR-568 | . | . | . | . | . | . |
| bta-miR-655 | 36.729 | 34.929 | . | . | 33.566 | . |
| bta-miR-574 | 27.783 | 26.821 | 27.978 | 27.813 | 26.362 | 27.220 |
| bta-miR-656 | 30.265 | 29.720 | 30.339 | 31.103 | 31.577 | 30.780 |
| bta-miR-658 | 36.388 | . | 36.057 | . | . | 36.561 |
| bta-miR-758 | . | 36.062 | . | . | . | . |
| bta-miR-660 | 28.947 | 28.168 | 27.889 | 28.617 | 27.995 | 27.755 |
| bta-miR-759 | . | . | . | . | . | . |
| bta-miR-664a | 31.805 | 30.747 | 31.230 | 32.339 | 31.765 | 31.606 |
| bta-miR-760-3p | 33.315 | 31.911 | 32.936 | 32.717 | 31.516 | 32.667 |
| bta-miR-664b | 24.642 | 24.111 | 24.351 | 24.756 | 23.840 | 23.780 |
| bta-miR-760-5p | 25.769 | . | 25.601 | 26.775 | 25.271 | 24.698 |
| bta-miR-665 | 29.856 | 29.352 | 30.145 | 31.488 | 29.631 | 29.868 |
| bta-miR-761 | 33.776 | 34.561 | 33.735 | 38.253 | 33.829 | 38.139 |
| bta-miR-669 | 30.720 | 29.811 | 29.716 | 30.068 | 28.750 | 30.833 |
| bta-miR-763 | 34.231 | 33.822 | 33.544 | 35.917 | 35.355 | 33.860 |
| bta-miR-670 | 35.027 | 34.811 | . | . | 34.712 | 33.198 |
| bta-miR-764 | 32.838 | 32.228 | 33.327 | 34.064 | 34.124 | 35.476 |
| bta-miR-671 | 33.439 | 33.782 | 35.034 | 33.438 | 32.826 | 33.250 |
| bta-miR-767 | 30.388 | 30.620 | 31.027 | 31.467 | 31.716 | 31.871 |
| bta-miR-677 | 30.625 | 29.894 | 31.012 | 31.485 | 30.532 | 30.678 |
| bta-miR-769 | 31.386 | 32.803 | 32.090 | 32.477 | 31.830 | 31.708 |
| bta-miR-7 | 26.392 | 26.672 | 26.805 | 26.469 | 26.108 | 26.072 |
| bta-miR-873 | . | 33.540 | 34.912 | 35.914 | 34.900 | . |
| bta-miR-708 | 29.234 | 28.390 | 29.585 | 29.213 | 29.151 | 28.525 |
| bta-miR-874 | 31.062 | 30.638 | 30.764 | 31.320 | 30.532 | 30.319 |
| bta-miR-744 | 26.907 | 27.255 | 27.331 | 27.642 | 27.420 | 27.495 |
| bta-miR-875 | 36.641 | . | . | . | . | . |
| bta-miR-876 | . | 35.514 | . | . | . | . |
| bta-miR-98 | 25.433 | 25.547 | 25.754 | 25.658 | 25.701 | 24.716 |
| bta-miR-877 | 30.640 | . | 30.865 | . | . | . |
| bta-miR-99a-3p | 31.752 | 32.599 | 32.732 | 33.492 | 32.025 | 31.880 |
| bta-miR-885 | 24.967 | 24.004 | 24.628 | 24.829 | 23.934 | 24.083 |
| bta-miR-99a-5p | 24.991 | 24.485 | 24.695 | 26.464 | 23.826 | 24.153 |
| bta-miR-9-3p | 32.808 | 31.300 | 32.924 | 35.259 | 32.123 | 33.800 |
| bta-miR-99b | 22.765 | 22.204 | 22.607 | 22.714 | 22.656 | 22.724 |
| bta-miR-9-5p | 25.524 | 24.575 | 25.512 | 25.522 | 24.135 | 26.018 |
| bta-miR-1179 | 33.744 | . | . | . | . | . |
| bta-miR-92a | 23.772 | 23.875 | 24.284 | 24.723 | 23.944 | 23.821 |
| bta-miR-1185 | . | . | . | . | . | . |
| bta-miR-92b | 23.582 | 23.088 | 23.422 | 23.723 | 23.261 | 23.256 |
| bta-miR-1193 | . | . | 38.136 | . | . | 35.056 |
| bta-miR-93 | 26.793 | 26.929 | 26.795 | 27.749 | 26.771 | 26.603 |
| bta-miR-1197 | . | . | . | . | . | . |
| bta-miR-935 | 32.880 | 31.783 | 32.893 | 32.514 | . | 32.517 |
| bta-miR-122 | . | 36.345 | 35.831 | 36.491 | . | . |
| bta-miR-940 | 26.266 | 25.639 | 26.228 | 26.917 | 25.808 | 25.900 |
| bta-miR-1224 | 29.840 | 29.350 | 31.121 | 30.194 | 29.655 | 29.737 |
| bta-miR-95 | 28.255 | 28.758 | 29.833 | 30.319 | 28.836 | 29.583 |
| bta-miR-1225-3p | 28.148 | 27.486 | 27.831 | 28.810 | 27.543 | 28.058 |
| bta-miR-96 | 32.527 | 33.976 | 31.624 | . | 32.795 | 31.940 |
| bta-miR-1246 | . | . | 23.826 | . | . | . |
| bta-miR-1247-3p | 31.781 | 31.060 | . | . | . | . |
| bta-miR-1296 | 31.822 | 32.795 | 33.861 | 32.526 | 31.160 | 32.635 |
| bta-miR-1247-5p | 30.126 | 29.654 | 30.062 | 31.259 | 29.297 | 29.793 |
| bta-miR-1298 | . | . | . | . | . | . |
| bta-miR-1248 | . | 29.024 | 30.086 | 32.160 | 29.708 | 30.373 |
| bta-miR-1301 | . | 33.529 | 35.400 | . | 32.861 | 32.254 |
| bta-miR-1249 | 27.154 | 26.896 | 27.767 | 27.099 | 27.009 | 26.534 |
| bta-miR-1306 | 28.205 | 27.732 | 28.093 | 28.537 | 27.830 | 28.189 |
| bta-miR-1260b | 22.015 | 21.570 | 22.252 | 22.756 | 21.682 | 21.752 |
| bta-miR-1307 | 27.320 | . | 27.532 | 28.207 | 27.236 | 26.978 |
| bta-miR-1271 | 34.748 | 34.026 | 34.777 | 34.396 | 32.280 | 31.857 |
| bta-miR-1343-3p | . | 28.830 | 29.786 | . | 28.709 | 28.830 |
| bta-miR-1277 | . | . | . | . | . | . |
| bta-miR-1343-5p | 28.756 | 28.829 | 29.594 | 29.745 | 28.116 | 28.951 |
| bta-miR-1281 | 30.625 | 29.949 | 30.561 | 31.610 | 29.726 | 29.971 |
| bta-miR-1388-3p | 30.691 | 29.790 | 30.730 | 30.400 | 31.356 | 30.642 |
| bta-miR-1282 | . | . | 34.911 | . | 34.270 | . |
| RNT43 snoRNA | 23.832 | 22.872 | 23.785 | 26.580 | 23.579 | 23.671 |
| bta-miR-1284 | 34.252 | 33.714 | . | 34.702 | 36.830 | 34.160 |
| Hm/Ms/Rt T1 snRNA | 17.126 | 16.434 | 16.607 | 18.796 | 16.172 | 16.331 |
| bta-miR-1287 | . | 33.872 | 34.786 | 36.843 | 34.914 | 34.008 |
| bta-miR-99b | 22.463 | 22.062 | 22.425 | 22.757 | 22.648 | 22.487 |
| bta-miR-1291 | 35.330 | 34.765 | 32.843 | 34.785 | 31.319 | 35.626 |
| Negative control | . | . | . | . | . | . |
| ^1^Body energy reserve: MBER: Cows with moderated body energy reserve; HBER: Cows with high body energy reserve. | | | | | | |
