# Supplementary material for: The bovine oviductal environment and composition are negatively affected by elevated body energy reserves
Source: PLoS One. 2025 Jun 23;20(6):e0326138. doi: 10.1371/journal.pone.0326138 (PMC12184905; doi:10.1371/journal.pone.0326138)
Supplement: S10 Table — (DOCX) [file pone.0326138.s013.docx]

| **Supplementary Table 10.** Normalized data of the 210 miRNAs commonly detected in ampullary luminal epithelial cells (AMP-Cell) of cows with different body energy reserve. | | | | | | | |
| --- | --- | --- | --- | --- | --- | --- | --- |
| **miRNA** | **Body energy reserve^1^** | | | | | | **P – value^2^** |
|  | **MBER** | | | **HBER** | | |  |
|  | **1** | **2** | **3** | **1** | **2** | **3** |  |
| bta-let-7a-3p | 4.957328 | 4.710137 | 5.603642 | 4.900023 | 3.895859 | 5.995809 | 0.8212 |
| bta-miR-103 | 3.934743 | 3.555394 | 6.934411 | 4.077137 | 3.441912 | 4.673582 | 0.5450 |
| bta-let-7a-5p | -1.0407 | -1.33307 | -0.08998 | -1.07646 | -1.3883 | -0.04521 | 0.9791 |
| bta-let-7b | 0.713087 | 1.074027 | 0.886325 | -0.10842 | 0.301775 | 0.812448 | 0.1239 |
| bta-miR-105b | 9.634544 | 10.67383 | 13.94961 | 10.09149 | 8.699806 | 11.32177 | 0.4105 |
| bta-let-7c | -0.4601 | -0.51107 | -0.00443 | -0.6334 | -1.03963 | -0.11397 | 0.4357 |
| bta-miR-106a | 5.005369 | 4.808623 | 7.03427 | 4.585173 | 4.423685 | 5.18202 | 0.3018 |
| bta-let-7d | 0.517088 | 0.524291 | 1.012826 | 0.527407 | 0.057995 | 1.149061 | 0.7796 |
| bta-let-7e | -1.78345 | -1.02627 | -0.33653 | -1.5242 | -2.23294 | -0.69096 | 0.5167 |
| bta-miR-107 | 7.876983 | 8.288043 | 10.17733 | 7.977879 | 7.667219 | 9.437534 | 0.6631 |
| bta-let-7f | -0.15379 | -0.32066 | 1.626803 | 0.571257 | -0.6073 | 1.229589 | 0.9875 |
| bta-miR-10a | 2.927812 | 2.407809 | 3.911635 | 3.504654 | 2.62499 | 4.810312 | 0.5059 |
| bta-let-7g | 1.336505 | 1.283955 | 3.062423 | 1.745238 | 0.807112 | 2.24152 | 0.7017 |
| bta-miR-10b | 3.133373 | 2.658036 | 4.652266 | 3.716582 | 2.864817 | 5.110916 | 0.6640 |
| bta-let-7i | 3.767807 | 3.089242 | 6.092017 | 3.310448 | 2.92713 | 4.02478 | 0.4056 |
| bta-miR-1 | 5.875831 | 5.251748 | 10.43913 | 6.697046 | 4.943197 | 7.666963 | 0.7001 |
| bta-miR-100 | 4.852184 | 4.579281 | 5.564168 | 4.019203 | 3.878679 | 4.197662 | **0.0348** |
| bta-miR-101 | 8.715038 | 8.27199 | 8.058301 | 6.617766 | 6.671478 | 7.658285 | **0.0248** |
| bta-miR-125a | 0.906248 | 0.674683 | 2.123738 | 1.444843 | 0.236759 | 2.196766 | 0.9403 |
| bta-miR-125b | 0.202713 | -0.18106 | 1.971929 | -0.39658 | -1.16439 | 0.158065 | 0.2134 |
| bta-miR-127 | 9.42955 | 10.64718 | 10.93601 | 10.99283 | 9.989996 | 11.64132 | 0.4653 |
| bta-miR-135a | 2.525102 | 1.533659 | 2.511962 | 1.052307 | 1.189773 | 2.201788 | 0.2207 |
| bta-miR-128 | 4.982053 | 5.584651 | 7.7984 | 5.685682 | 5.516405 | 6.937538 | 0.9417 |
| bta-miR-135b | 4.024982 | 3.23815 | 3.915713 | 2.7673 | 2.795528 | 3.693962 | 0.1769 |
| bta-miR-129-5p | 9.144226 | 9.146725 | 12.1047 | 10.75544 | 8.885351 | 10.17447 | 0.8724 |
| bta-miR-130a | 8.436031 | 9.333249 | 10.11151 | 8.734917 | 8.07004 | 9.273494 | 0.3706 |
| bta-miR-139 | 7.624116 | 6.757847 | 9.328551 | 6.966444 | 7.732016 | 8.246996 | 0.7770 |
| bta-miR-130b | 1.716774 | 1.700852 | 3.483785 | 2.462773 | 1.739601 | 3.289281 | 0.8040 |
| bta-miR-140 | 5.945303 | 5.733063 | 8.839791 | 6.400744 | 5.068563 | 7.77746 | 0.7556 |
| bta-miR-132 | 7.764364 | 8.405299 | 11.09631 | 7.60248 | 7.840177 | 9.567003 | 0.5629 |
| bta-miR-141 | 5.154054 | 4.731154 | 6.463277 | 4.742146 | 4.364565 | 5.253402 | 0.3180 |
| bta-miR-133a | 13.03179 | 10.74463 | 12.71399 | 8.118136 | 10.66413 | 10.20535 | 0.0779 |
| bta-miR-151-3p | 3.927322 | 3.492854 | 5.942998 | 4.447006 | 3.413942 | 5.722093 | 0.9455 |
| bta-miR-143 | 7.808214 | 9.176606 | 8.019809 | 8.148466 | 8.512464 | 9.173742 | 0.6232 |
| bta-miR-151-5p | 2.394638 | 1.719218 | 4.418401 | 2.554201 | 1.650949 | 3.571273 | 0.8103 |
| bta-miR-145 | 7.884601 | 8.65127 | 8.611838 | 6.700665 | 7.689562 | 8.283195 | 0.1910 |
| bta-miR-153 | 10.52148 | 10.20687 | 9.097773 | 8.75362 | 11.57278 | 9.316486 | 0.9525 |
| bta-miR-146a | 11.08276 | 10.69557 | 12.35482 | 12.83467 | 12.90081 | 11.94099 | 0.1154 |
| bta-miR-154a | 12.0584 | 13.62574 | 11.69236 | 8.898516 | 12.04021 | 13.48956 | 0.5428 |
| bta-miR-154b | 6.822372 | 9.738045 | 9.194911 | 6.733311 | 9.018598 | 10.26114 | 0.9529 |
| bta-miR-148a | 0.511392 | -0.28448 | 0.735795 | -0.85391 | -1.07194 | 0.287237 | 0.1726 |
| bta-miR-155 | 10.17506 | 8.611487 | 10.99639 | 8.778744 | 9.405512 | 10.6473 | 0.7394 |
| bta-miR-148b | 1.117612 | 0.797009 | 1.187858 | -0.40383 | -0.0945 | 1.029152 | 0.1306 |
| bta-miR-15a | 6.873283 | 6.555979 | 9.937133 | 6.505936 | 6.474205 | 7.256885 | 0.3998 |
| bta-miR-15b | 3.18791 | 3.559334 | 4.872838 | 3.660479 | 3.525618 | 4.718773 | 0.8884 |
| bta-miR-149-5p | 8.680679 | 11.03685 | 13.38745 | 7.807711 | 9.51959 | 10.31572 | 0.3045 |
| bta-miR-16a | 2.886583 | 2.730166 | 4.834689 | 2.763123 | 2.616796 | 3.32142 | 0.4575 |
| bta-miR-150 | 5.830991 | 6.046217 | 8.963254 | 7.106392 | 6.35895 | 8.215044 | 0.8189 |
| bta-miR-16b | 2.055996 | 1.72654 | 4.100799 | 2.019779 | 1.699417 | 2.581775 | 0.5390 |
| bta-miR-188 | 11.09493 | 10.12727 | 11.27974 | 10.0219 | 9.420259 | 11.3343 | 0.4382 |
| bta-miR-17-5p | 9.538467 | 8.637652 | 10.35567 | 8.758341 | 7.925947 | 9.236011 | 0.2371 |
| bta-miR-181b | 7.88706 | 7.501322 | 9.195849 | 8.507481 | 7.793415 | 10.18061 | 0.5092 |
| bta-miR-190a | 10.51693 | 9.510144 | 10.12479 | 8.677942 | 8.870993 | 8.738355 | **0.0125** |
| bta-miR-190b | 1.918253 | 1.920249 | 5.960677 | 4.116883 | 2.030336 | 4.857496 | 0.8130 |
| bta-miR-181d | 6.929351 | 6.54324 | 9.099317 | 6.977419 | 7.193397 | 7.759197 | 0.8091 |
| bta-miR-191 | 1.492349 | 1.418519 | 4.067147 | 2.063374 | 0.92824 | 3.208051 | 0.8238 |
| bta-miR-182 | 5.913456 | 6.889178 | 8.654361 | 6.524439 | 6.80759 | 8.121711 | 0.9991 |
| bta-miR-192 | 8.126216 | 8.296093 | 11.26366 | 8.720597 | 7.624346 | 10.65624 | 0.8740 |
| bta-miR-183 | 6.949541 | 7.371787 | 8.985028 | 7.715531 | 7.26104 | 8.887725 | 0.8248 |
| bta-miR-185 | 5.766346 | 5.601605 | 7.962577 | 6.203346 | 5.809851 | 7.157741 | 0.9536 |
| bta-miR-193a-5p | 6.778129 | 6.954251 | 7.835959 | 6.50992 | 7.22813 | 8.204071 | 0.8431 |
| bta-miR-186 | 5.20302 | 4.436335 | 7.015626 | 4.07954 | 4.301729 | 5.449216 | 0.3423 |
| bta-miR-194 | 6.883619 | 6.772456 | 7.894576 | 6.40671 | 6.019218 | 7.22571 | 0.2774 |
| bta-miR-195 | 2.779762 | 2.514582 | 5.422271 | 3.270064 | 2.059559 | 3.455003 | 0.5643 |
| bta-miR-200c | -2.15609 | -2.2389 | -0.03099 | -1.31245 | -2.24428 | -0.14123 | 0.8099 |
| bta-miR-204 | 2.968595 | 2.779143 | 3.346501 | 2.644581 | 3.567303 | 6.591396 | 0.3624 |
| bta-miR-197 | 3.455089 | 3.336913 | 5.238709 | 4.795603 | 3.802135 | 6.14084 | 0.3799 |
| bta-miR-205 | 9.989063 | 8.418688 | 8.053657 | 7.222338 | 5.788675 | 8.284412 | 0.1395 |
| bta-miR-199a-3p | 9.039775 | 11.81688 | 11.61018 | 9.735724 | 9.989141 | 11.48393 | 0.7091 |
| bta-miR-206 | 10.91479 | 10.63577 | 12.25337 | 8.388529 | 11.20376 | 12.87485 | 0.7664 |
| bta-miR-199c | 8.084816 | 7.737692 | 9.584502 | 8.628023 | 7.844433 | 11.40716 | 0.5365 |
| bta-miR-20a | 5.327055 | 4.73905 | 6.726535 | 4.618719 | 4.359238 | 5.170383 | 0.2381 |
| bta-miR-19a | 8.269191 | 7.478623 | 9.759181 | 6.316694 | 6.902447 | 6.671137 | 0.0533 |
| bta-miR-20b | 6.57929 | 6.254099 | 8.891171 | 5.853029 | 5.735495 | 7.04841 | 0.3305 |
| bta-miR-19b | 8.224975 | 8.441882 | 7.474994 | 6.276274 | 6.797153 | 6.2201 | **0.0095** |
| bta-miR-200a | 6.308195 | 5.503607 | 7.96161 | 5.305886 | 4.947872 | 6.151333 | 0.2364 |
| bta-miR-21-5p | 8.253513 | 6.745862 | 7.125411 | 6.910181 | 5.854633 | 7.271232 | 0.3250 |
| bta-miR-200b | -3.65475 | -3.66191 | -1.51197 | -2.71407 | -4.1783 | -1.72997 | 0.9489 |
| bta-miR-210 | 7.940921 | 6.596594 | 9.181121 | 7.17041 | 5.886116 | 7.755981 | 0.3557 |
| bta-miR-211 | 3.763914 | 3.496691 | 4.096919 | 3.588801 | 3.969715 | 6.821828 | 0.3853 |
| bta-miR-22-5p | 7.248733 | 6.791432 | 9.424888 | 6.999909 | 7.728886 | 8.504377 | 0.9372 |
| bta-miR-221 | 4.933702 | 4.623969 | 8.088994 | 5.657812 | 4.783888 | 6.151453 | 0.7803 |
| bta-miR-214 | 9.557331 | 11.31614 | 11.34486 | 10.65302 | 12.38289 | 10.87007 | 0.5224 |
| bta-miR-215 | 7.954111 | 7.6013 | 9.014977 | 8.191481 | 6.849884 | 10.11438 | 0.8601 |
| bta-miR-223 | 9.911121 | 10.08429 | 12.97595 | 7.765795 | 8.707937 | 9.238763 | 0.0893 |
| bta-miR-216a | 12.88902 | 11.29924 | 11.79018 | 10.92185 | 10.57015 | 11.62514 | 0.1656 |
| bta-miR-224 | 5.738582 | 5.818274 | 8.82767 | 6.519949 | 6.620836 | 8.123159 | 0.8100 |
| bta-miR-23a | -1.86661 | -1.63058 | 0.355559 | -0.30047 | -1.88067 | 0.49636 | 0.6502 |
| bta-miR-23b-3p | 1.049789 | 1.622374 | 3.368932 | 2.608238 | 0.801729 | 3.264572 | 0.8453 |
| bta-miR-218 | 6.84526 | 6.496445 | 9.934554 | 8.545811 | 7.414841 | 9.48776 | 0.5924 |
| bta-miR-219-3p | 8.526126 | 9.735291 | 10.43225 | 9.18644 | 8.787033 | 10.03008 | 0.7474 |
| bta-miR-24-3p | 1.817974 | 1.627251 | 3.768222 | 1.903317 | 1.318138 | 2.868271 | 0.6715 |
| bta-miR-25 | 1.599582 | 1.598759 | 3.858111 | 2.783463 | 1.403734 | 3.66918 | 0.8031 |
| bta-miR-22-3p | -21.4511 | -20.446 | -19.8915 | -18.2688 | -20.6345 | -18.7262 | 0.1807 |
| bta-miR-26a | -1.58122 | -1.81146 | -0.07098 | -1.47496 | -2.50348 | -0.21981 | 0.7892 |
| bta-miR-26b | -0.0908 | -0.47214 | 1.479104 | 0.057619 | -0.48149 | 1.499914 | 0.9525 |
| bta-miR-29d-3p | 2.960552 | 2.561276 | 3.557193 | 2.708985 | 1.894112 | 3.188808 | 0.4183 |
| bta-miR-29d-5p | 7.57998 | 6.494301 | 8.475402 | 7.375174 | 6.372974 | 8.477535 | 0.9033 |
| bta-miR-27a-3p | 3.123663 | 2.570502 | 3.619794 | 2.641772 | 2.459412 | 3.218078 | 0.4317 |
| bta-miR-27b | 2.858566 | 2.725079 | 3.920865 | 2.666798 | 1.881233 | 3.654609 | 0.5334 |
| bta-miR-28 | 6.90302 | 6.826672 | 8.92665 | 6.88584 | 6.106817 | 8.306438 | 0.6561 |
| bta-miR-29a | 0.513004 | 0.289727 | 1.352984 | 0.358208 | -0.15441 | 1.327531 | 0.7204 |
| bta-miR-29b | 12.84971 | 11.66599 | 10.93024 | 8.912522 | 12.09274 | 10.14478 | 0.2561 |
| bta-miR-30a-5p | 5.584798 | 5.207531 | 6.689511 | 4.130207 | 4.329611 | 4.974004 | 0.0580 |
| bta-miR-29c | 0.65352 | 0.303526 | 1.140702 | 0.511557 | -0.15671 | 1.230138 | 0.7336 |
| bta-miR-30b-3p | 8.248336 | 8.140564 | 10.8772 | 9.220459 | 7.948723 | 10.43823 | 0.9258 |
| bta-miR-30b-5p | 4.001749 | 3.358878 | 4.005103 | 2.137993 | 2.743048 | 3.09003 | **0.0323** |
| bta-miR-328 | 9.574749 | 7.036929 | 8.036223 | 7.887081 | 7.685032 | 8.262836 | 0.7385 |
| bta-miR-30c | 1.983312 | 1.526789 | 2.861464 | 1.116154 | 0.909161 | 2.152183 | 0.2535 |
| bta-miR-30d | 5.313299 | 4.989874 | 6.364535 | 4.343756 | 4.782478 | 5.074335 | 0.1524 |
| bta-miR-30e-5p | 5.632255 | 5.611674 | 6.556521 | 4.287729 | 4.611883 | 5.140152 | **0.0347** |
| bta-miR-330 | 11.4823 | 9.717606 | 10.94924 | 11.55547 | 14.00247 | 12.13604 | 0.1105 |
| bta-miR-30f | 3.889562 | 3.394658 | 4.548922 | 2.733134 | 2.881733 | 3.935281 | 0.2063 |
| bta-miR-331-3p | 7.617387 | 6.822918 | 13.11705 | 7.336321 | 6.801004 | 8.608095 | 0.4777 |
| bta-miR-31 | 1.817062 | 1.578612 | 4.176082 | 2.414753 | 1.33897 | 3.302107 | 0.8724 |
| bta-miR-331-5p | 7.798816 | 6.898755 | 9.051114 | 7.499821 | 7.825375 | 9.199062 | 0.7663 |
| bta-miR-335 | 8.946167 | 10.71075 | 9.938197 | 8.499825 | 7.827169 | 9.017814 | 0.0830 |
| bta-miR-320a | 3.157922 | 3.558272 | 5.8616 | 3.752091 | 3.754589 | 5.171535 | 0.9740 |
| bta-miR-339a | 6.203893 | 6.216353 | 8.086615 | 5.712172 | 5.554058 | 7.139061 | 0.4324 |
| bta-miR-323 | -6.87547 | -5.58457 | -5.5455 | -3.51853 | -5.60501 | -3.98383 | 0.1009 |
| bta-miR-339b | 5.638906 | 5.580695 | 7.272023 | 5.207388 | 4.999304 | 6.261123 | 0.3761 |
| bta-miR-326 | 9.572479 | 10.49514 | 11.68122 | 9.667879 | 10.11928 | 11.31741 | 0.7977 |
| bta-miR-33b | 11.44429 | 11.80042 | 12.92859 | 12.78211 | 12.00009 | 14.80659 | 0.2962 |
| bta-miR-340 | 9.713878 | 8.42305 | 9.208716 | 8.340616 | 8.561211 | 10.13351 | 0.8861 |
| bta-miR-365-3p | 5.63322 | 5.3513 | 6.457684 | 4.689039 | 4.204908 | 5.90361 | 0.2185 |
| bta-miR-342 | 6.940176 | 6.530652 | 8.07594 | 6.956169 | 6.522608 | 8.287063 | 0.9224 |
| bta-miR-365-5p | 10.59943 | 13.07515 | 13.49961 | 10.60972 | 9.487909 | 9.207008 | 0.0587 |
| bta-miR-346 | 9.305862 | 10.03293 | 12.24984 | 9.42115 | 9.148424 | 8.994718 | 0.2079 |
| bta-miR-34a | 5.275007 | 5.459939 | 8.126225 | 5.6096 | 5.270756 | 7.00909 | 0.7759 |
| bta-miR-34b | 3.346759 | 2.489499 | 6.126975 | 3.788027 | 2.187342 | 4.251066 | 0.6706 |
| bta-miR-34c | 3.073589 | 2.538954 | 6.046487 | 3.772135 | 1.904272 | 4.235586 | 0.6781 |
| bta-miR-374a | 4.951097 | 4.497391 | 5.707644 | 5.084009 | 5.073393 | 5.29326 | 0.7987 |
| bta-miR-361 | 2.884279 | 2.8886 | 6.10241 | 4.730881 | 2.569126 | 6.784 | 0.6734 |
| bta-miR-374b | 3.304306 | 2.972098 | 4.490649 | 3.559471 | 2.466431 | 4.29009 | 0.8409 |
| bta-miR-375 | 2.202114 | 1.871428 | 4.614825 | 2.779177 | 1.322126 | 4.255132 | 0.9315 |
| bta-miR-362-5p | 10.24869 | 9.355896 | 14.26163 | 9.936245 | 9.90101 | 11.33936 | 0.6010 |
| bta-miR-383 | 11.26408 | 10.36483 | 11.41637 | 9.565891 | 13.64528 | 12.09446 | 0.5743 |
| bta-miR-378 | 5.838514 | 6.483336 | 9.628344 | 6.675989 | 7.063993 | 7.589452 | 0.8715 |
| bta-miR-378b | 7.231424 | 7.326777 | 8.965785 | 6.676981 | 7.41473 | 7.768686 | 0.4405 |
| bta-miR-378c | 9.37022 | 9.666203 | 12.17972 | 10.84306 | 9.977945 | 11.16837 | 0.8015 |
| bta-miR-378d | 11.44958 | 10.96785 | 13.56378 | 9.792742 | 11.09139 | 12.1375 | 0.3993 |
| bta-miR-421 | 6.574243 | 6.076994 | 7.808314 | 6.502897 | 6.038385 | 8.09057 | 0.9467 |
| bta-miR-423-3p | 6.037948 | 5.546311 | 7.969039 | 5.708933 | 5.241442 | 7.061456 | 0.6059 |
| bta-miR-423-5p | 4.416965 | 4.724724 | 5.846557 | 4.718205 | 4.40864 | 5.983078 | 0.9531 |
| bta-miR-424-3p | 9.453476 | 10.82149 | 11.88903 | 11.79803 | 9.730635 | 12.32632 | 0.6231 |
| bta-miR-449d | 7.994285 | 8.765257 | 10.2173 | 10.33865 | 8.993185 | 10.24875 | 0.3299 |
| bta-miR-424-5p | 5.649225 | 4.723309 | 6.627332 | 4.4744 | 4.414358 | 4.846441 | 0.1269 |
| bta-miR-450a | 8.908485 | 8.598023 | 12.30236 | 9.004648 | 8.904821 | 9.557563 | 0.5520 |
| bta-miR-450b | 9.048915 | 7.731995 | 10.29144 | 8.460501 | 7.933653 | 9.15736 | 0.5697 |
| bta-miR-425-5p | 7.230026 | 6.54917 | 7.782964 | 5.635527 | 5.985878 | 6.367429 | **0.0454** |
| bta-miR-429 | 3.451511 | 3.027434 | 4.522788 | 2.96813 | 2.795107 | 3.877306 | 0.4613 |
| bta-miR-433 | 6.222456 | 7.538983 | 7.165563 | 7.28479 | 6.911556 | 8.58745 | 0.3893 |
| bta-miR-454 | 11.29195 | 9.238556 | 11.80501 | 8.372565 | 8.70669 | 10.35154 | 0.1755 |
| bta-miR-455-3p | 8.991249 | 9.650998 | 11.37304 | 8.697527 | 7.687487 | 10.1378 | 0.3111 |
| bta-miR-449a | 2.301609 | 3.070122 | 7.242812 | 2.593554 | 3.40563 | 4.341501 | 0.6635 |
| bta-miR-455-5p | 10.92947 | 11.19606 | 11.92483 | 11.24675 | 9.984358 | 11.37715 | 0.4194 |
| bta-miR-449b | 4.006616 | 5.423253 | 9.010396 | 4.771773 | 5.857822 | 6.38756 | 0.7767 |
| bta-miR-484 | 8.015934 | 8.189942 | 9.233613 | 7.389044 | 6.961212 | 8.572319 | 0.2434 |
| bta-miR-497 | 8.459186 | 7.726315 | 13.72761 | 9.431275 | 7.781032 | 9.249073 | 0.5889 |
| bta-miR-499 | 11.0326 | 10.38487 | 11.25529 | 11.03837 | 10.11902 | 10.29397 | 0.3490 |
| bta-miR-500 | 8.242279 | 7.773655 | 12.23378 | 8.169827 | 7.785417 | 9.500553 | 0.5701 |
| bta-miR-502b | 7.640432 | 8.60034 | 10.43956 | 8.686476 | 8.003576 | 9.286436 | 0.8074 |
| bta-miR-489 | 10.20182 | 10.7048 | 12.08776 | 13.45629 | 10.95536 | 14.89445 | 0.1760 |
| bta-miR-491 | 5.994773 | 6.585576 | 7.712851 | 5.654963 | 5.622787 | 6.402773 | 0.1979 |
| bta-miR-493 | 8.721435 | 8.500248 | 14.22863 | 9.576653 | 9.306368 | 9.127399 | 0.5745 |
| bta-miR-505 | 5.664777 | 4.670451 | 7.858706 | 5.639826 | 4.934887 | 6.548732 | 0.7514 |
| bta-miR-532 | 10.59041 | 9.903054 | 10.17387 | 9.071078 | 10.0598 | 12.42695 | 0.7846 |
| bta-miR-541 | 8.084024 | 8.467709 | 10.17366 | 9.570917 | 8.742039 | 10.90252 | 0.4082 |
| bta-miR-542-5p | 11.42513 | 9.552121 | 12.40988 | 10.81999 | 9.950926 | 10.18377 | 0.4078 |
| bta-miR-584 | 9.565757 | 10.3498 | 12.67492 | 10.40006 | 11.0692 | 10.17248 | 0.7611 |
| bta-miR-628 | 7.843614 | 8.640168 | 11.04425 | 9.164461 | 7.897455 | 12.38596 | 0.7173 |
| bta-miR-631 | -4.65467 | -3.93848 | -3.63716 | -1.81055 | -3.64133 | -2.47585 | 0.0798 |
| bta-miR-652 | 4.634822 | 4.885613 | 7.037564 | 5.663447 | 4.776258 | 6.61639 | 0.8669 |
| bta-miR-654 | 10.03233 | 9.025322 | 11.16792 | 10.95773 | 10.67788 | 10.35334 | 0.4124 |
| bta-miR-574 | 4.147062 | 4.30226 | 6.286393 | 5.230932 | 4.081882 | 5.042827 | 0.8781 |
| bta-miR-656 | 9.878585 | 9.622822 | 11.33995 | 10.41637 | 10.97996 | 11.6982 | 0.3128 |
| bta-miR-660 | 7.941553 | 6.01377 | 8.034806 | 6.265495 | 5.908876 | 7.617126 | 0.4319 |
| bta-miR-664a | 10.03448 | 10.03658 | 9.974559 | 9.544131 | 10.642 | 10.29162 | 0.6800 |
| bta-miR-664b | 2.216659 | 2.203379 | 4.522262 | 2.926233 | 1.829159 | 4.192325 | 0.9987 |
| bta-miR-760-5p | 2.687721 | 2.686995 | 5.132523 | 3.637402 | 2.71516 | 4.073179 | 0.9776 |
| bta-miR-665 | 8.282791 | 7.780324 | 9.998149 | 8.196509 | 6.815786 | 8.16773 | 0.3021 |
| bta-miR-669 | 5.820089 | 6.574873 | 8.57245 | 7.647051 | 6.1469 | 7.903379 | 0.8174 |
| bta-miR-763 | 10.51684 | 11.99927 | 12.66828 | 10.75734 | 11.91386 | 12.64857 | 0.9598 |
| bta-miR-767 | 9.063957 | 10.22474 | 10.15205 | 11.43194 | 9.991815 | 12.06306 | 0.1338 |
| bta-miR-677 | 12.63295 | 8.627518 | 10.1587 | 9.636494 | 10.16105 | 10.23672 | 0.7160 |
| bta-miR-7 | 6.08333 | 5.598288 | 7.3642 | 6.348009 | 5.383436 | 7.750675 | 0.8748 |
| bta-miR-708 | 6.607313 | 7.019991 | 8.690469 | 7.16761 | 6.872496 | 8.158764 | 0.9602 |
| bta-miR-744 | 3.837909 | 3.976883 | 6.555474 | 5.587012 | 4.068935 | 6.982872 | 0.5690 |
| bta-miR-98 | 4.239114 | 3.778428 | 5.997329 | 4.890709 | 3.850599 | 5.723762 | 0.8709 |
| bta-miR-885 | 2.930585 | 2.776177 | 4.043368 | 3.154339 | 2.477434 | 4.195877 | 0.9697 |
| bta-miR-99a-5p | 4.982006 | 4.489634 | 5.311686 | 2.936751 | 3.50029 | 3.393957 | **0.0050** |
| bta-miR-9-3p | 11.15855 | 9.761532 | 12.87353 | 9.474606 | 10.97231 | 13.56591 | 0.9634 |
| bta-miR-9-5p | 2.051412 | 1.547396 | 4.900798 | 3.703411 | 1.574169 | 5.749338 | 0.6252 |
| bta-miR-92a | 2.937822 | 2.782414 | 4.129571 | 3.45143 | 2.770748 | 4.146324 | 0.7812 |
| bta-miR-92b | 1.448965 | 1.63283 | 2.870503 | 1.829718 | 1.474815 | 2.958782 | 0.8777 |
| bta-miR-93 | 5.049568 | 4.919138 | 7.567954 | 5.42902 | 4.835024 | 6.670244 | 0.8532 |
| bta-miR-940 | 3.864715 | 3.23975 | 6.114692 | 4.721945 | 3.764242 | 4.873165 | 0.9627 |
| bta-miR-95 | 6.941335 | 8.520415 | 7.670815 | 8.596195 | 8.738373 | 10.33501 | 0.1037 |
| bta-miR-1225-3p | 5.072008 | 4.596341 | 7.464915 | 5.95036 | 4.927195 | 6.266348 | 0.9973 |
| bta-miR-1247-5p | 8.028785 | 7.641915 | 11.23444 | 8.70414 | 7.365669 | 8.48081 | 0.5524 |
| bta-miR-1248 | 8.933826 | 9.131554 | 10.18869 | 9.178377 | 9.878683 | 8.960094 | 0.8768 |
| bta-miR-1249 | 6.035329 | 5.501704 | 6.912585 | 6.781233 | 5.834093 | 7.593181 | 0.4206 |
| bta-miR-1306 | 5.782592 | 6.201235 | 7.5836 | 6.629732 | 5.923698 | 7.225586 | 0.9202 |
| bta-miR-1260b | 0.295866 | 0.014042 | 1.323609 | 1.17152 | 0.788597 | 2.122493 | 0.2198 |
| bta-miR-1343-3p | 8.071203 | 7.936371 | 7.626569 | 7.530056 | 7.211784 | 8.247701 | 0.5544 |
| bta-miR-1343-5p | 6.241722 | 6.36274 | 8.213984 | 7.604767 | 6.424332 | 7.417011 | 0.7903 |
| bta-miR-1388-3p | 9.046562 | 7.737687 | 9.812991 | 7.328572 | 9.320352 | 10.00703 | 0.9854 |
| RNT43 snoRNA | 4.740491 | 4.790825 | 3.656894 | 3.655661 | 4.813814 | 3.203348 | 0.4512 |
| Hm/Ms/Rt T1 snRNA | -2.90639 | -3.62009 | -3.0259 | -4.36248 | -3.90197 | -3.76165 | **0.0447** |
| bta-miR-99b | -1.27366 | -0.50674 | 0.051812 | 1.434611 | -0.10847 | 1.22393 | 0.0820 |
| ^1^Body energy reserve: MBER: Cows with moderated body energy reserve; HBER: Cows with high body energy reserve; ^2^P-value: P value between animals with different body energy reserve. | | | | | | | |
