# Supplementary material for: The bovine oviductal environment and composition are negatively affected by elevated body energy reserves
Source: PLoS One. 2025 Jun 23;20(6):e0326138. doi: 10.1371/journal.pone.0326138 (PMC12184905; doi:10.1371/journal.pone.0326138)
Supplement: S11 Table — (DOCX) [file pone.0326138.s014.docx]

| **Supplementary Table 11.** Normalized data of the 242 miRNAs commonly detected in isthmic luminal epithelial cells (IST-Cell) of cows with different body energy reserve. | | | | | | | |
| --- | --- | --- | --- | --- | --- | --- | --- |
| **miRNA** | **Body energy reserve** | | | | | | **P - value** |
|  | **MBER** | | | **HBER** | | |  |
|  | **1** | **2** | **3** | **1** | **2** | **3** |  |
| bta-let-7a-3p | 5.014058 | 5.598746 | 5.870683 | 3.862506 | 5.185162 | 5.219811 | 0.2235 |
| bta-miR-103 | 5.7476 | 5.75528 | 5.477537 | 5.133969 | 5.354506 | 5.534808 | 0.0967 |
| bta-let-7a-5p | -0.92236 | -0.06985 | -0.06351 | -2.84431 | -0.52793 | -1.08804 | 0.2067 |
| bta-let-7b | 0.375876 | 1.151021 | 1.062555 | -1.67577 | 0.527716 | 0.183511 | 0.1785 |
| bta-let-7c | -0.84358 | 0.056178 | 0.07607 | -2.69746 | -0.4098 | -0.93443 | 0.2156 |
| bta-miR-106a | 5.857308 | 5.842846 | 5.620696 | 5.35158 | 5.418284 | 5.314286 | **0.0075** |
| bta-let-7d | 0.205119 | 0.984046 | 1.093778 | -1.70406 | 0.843077 | 0.132378 | 0.2823 |
| bta-miR-106b | 7.706094 | 8.043633 | 6.991632 | 8.005068 | 7.26532 | 7.357137 | 0.9268 |
| bta-let-7e | -1.07442 | -0.47689 | -0.16977 | -3.02424 | -0.7187 | -1.32474 | 0.2059 |
| bta-miR-107 | 9.826512 | 9.555918 | 9.018369 | 9.011109 | 10.20278 | 9.274215 | 0.9496 |
| bta-miR-10a | 2.736472 | 4.015843 | 4.548503 | 2.242858 | 3.337664 | 3.678697 | 0.3801 |
| bta-let-7g | 1.635184 | 2.134858 | 2.468021 | 0.703808 | 1.929216 | 1.583384 | 0.1985 |
| bta-miR-10b | 3.202386 | 4.248843 | 4.560674 | 2.912436 | 4.063286 | 4.103265 | 0.6124 |
| bta-let-7i | 4.536073 | 4.505307 | 4.247873 | 3.233325 | 4.454567 | 4.14589 | 0.2685 |
| bta-miR-1 | 8.834472 | 8.872365 | 6.758165 | 7.694642 | 8.737869 | 8.865985 | 0.7431 |
| bta-miR-100 | 4.135989 | 4.729983 | 5.013723 | 3.916819 | 4.074991 | 4.067153 | 0.0828 |
| bta-miR-101 | 7.990672 | 7.772967 | 7.859718 | 7.320513 | 7.419317 | 7.71271 | **0.0433** |
| bta-miR-125a | 1.414516 | 2.127108 | 2.022575 | 0.454739 | 1.62866 | 1.49235 | 0.1999 |
| bta-miR-125b | 1.545478 | 1.7272 | 1.089355 | 0.662634 | 1.13051 | 0.972049 | 0.0855 |
| bta-miR-127 | 9.625056 | 9.740813 | 9.676689 | 8.93908 | 10.21166 | 11.12668 | 0.5523 |
| bta-miR-135a | 1.731618 | 1.647423 | 1.351795 | 0.352223 | 1.297976 | 1.166995 | 0.1149 |
| bta-miR-128 | 5.33953 | 5.563132 | 6.120567 | 4.872746 | 5.908394 | 5.234996 | 0.4292 |
| bta-miR-135b | 2.798474 | 2.954587 | 2.634874 | 1.796271 | 2.992836 | 2.354432 | 0.3108 |
| bta-miR-129-3p | 12.96511 | 11.26148 | 12.14277 | 10.23562 | 10.79368 | 10.92876 | 0.0516 |
| bta-miR-130a | 8.793391 | 9.748334 | 9.341924 | 8.671897 | 10.29843 | 10.9843 | 0.4034 |
| bta-miR-139 | 8.055615 | 8.217722 | 8.392846 | 6.3539 | 8.023321 | 7.048568 | 0.0940 |
| bta-miR-130b | 2.422862 | 2.523005 | 2.549922 | 1.480776 | 2.764788 | 2.791217 | 0.7423 |
| bta-miR-140 | 7.825021 | 8.054993 | 7.752752 | 7.086857 | 7.418432 | 7.988973 | 0.2450 |
| bta-miR-132 | 8.770837 | 9.624299 | 10.41184 | 8.310572 | 10.27967 | 8.892789 | 0.5889 |
| bta-miR-141 | 6.812951 | 6.296645 | 6.087903 | 6.299546 | 5.443306 | 5.81483 | 0.1714 |
| bta-miR-133a | 11.88279 | 13.02466 | 10.37612 | 12.83485 | 12.40954 | 13.2381 | 0.2551 |
| bta-miR-142-5p | 12.98166 | 13.64743 | 11.94916 | 13.08802 | 12.75187 | 13.04353 | 0.8502 |
| bta-miR-151-3p | 5.769769 | 5.866543 | 5.511983 | 4.267396 | 5.470459 | 5.343924 | 0.1570 |
| bta-miR-143 | 5.393212 | 5.140639 | 5.108753 | 4.041085 | 5.289835 | 4.87206 | 0.2730 |
| bta-miR-151-5p | 3.478557 | 3.538138 | 3.34563 | 2.045605 | 3.18154 | 3.203078 | 0.1710 |
| bta-miR-152 | 10.4461 | 11.41471 | 10.35937 | 9.773167 | 11.39149 | 9.665105 | 0.5169 |
| bta-miR-145 | 7.810885 | 8.250996 | 9.767846 | 7.333567 | 7.457817 | 7.449572 | 0.1143 |
| bta-miR-153 | 8.660161 | 8.627259 | 8.94827 | 8.263373 | 9.223749 | 8.037099 | 0.5642 |
| bta-miR-146a | 10.92113 | 13.06501 | 10.62962 | 9.71069 | 11.01191 | 15.50878 | 0.7927 |
| bta-miR-154a | 11.92101 | 12.40761 | 14.3301 | 12.45866 | 12.44417 | 11.85169 | 0.4518 |
| bta-miR-154b | 8.686307 | 9.157603 | 7.92126 | 8.283393 | 9.617508 | 9.124993 | 0.4726 |
| bta-miR-154c | 12.02114 | 14.35039 | 13.40978 | 13.76032 | 13.76711 | 14.97584 | 0.3137 |
| bta-miR-148a | 0.000928 | 0.092606 | -0.23298 | -1.42246 | -0.562 | -0.78548 | **0.0334** |
| bta-miR-155 | 8.448035 | 9.601348 | 10.5373 | 7.337124 | 10.6291 | 9.620753 | 0.7857 |
| bta-miR-148b | 0.805135 | 0.594369 | 0.093545 | -0.76306 | 0.193863 | -0.13647 | 0.1051 |
| bta-miR-15a | 7.845773 | 8.085436 | 7.159084 | 7.808217 | 7.671515 | 7.331668 | 0.7803 |
| bta-miR-149-3p | 8.426673 | 9.8053 | 9.737117 | 9.05818 | 9.801554 | 9.971974 | 0.6157 |
| bta-miR-15b | 3.817199 | 4.927165 | 4.589036 | 2.752047 | 4.601879 | 3.935864 | 0.3423 |
| bta-miR-149-5p | 10.75598 | 11.3678 | 12.06535 | 10.45097 | 10.57228 | 10.26221 | 0.0676 |
| bta-miR-16a | 3.791547 | 4.08923 | 3.334339 | 3.20999 | 3.541458 | 3.417693 | 0.2198 |
| bta-miR-150 | 5.720658 | 7.522349 | 7.638507 | 6.040198 | 7.284548 | 7.32463 | 0.9228 |
| bta-miR-16b | 3.615251 | 3.827019 | 3.177383 | 2.981677 | 3.472607 | 3.228825 | 0.2599 |
| bta-miR-17-3p | 13.02121 | 12.5747 | 11.40833 | 10.69931 | 11.04911 | 12.82093 | 0.3751 |
| bta-miR-17-5p | 8.787074 | 9.200785 | 8.843517 | 7.957453 | 8.913721 | 8.695012 | 0.2541 |
| bta-miR-181a | 10.59502 | 11.85379 | 11.10004 | 9.377148 | 10.91944 | 10.99276 | 0.3053 |
| bta-miR-18b | 12.85321 | 12.88397 | 13.03988 | 12.55037 | 12.55393 | 12.74977 | **0.0247** |
| bta-miR-181b | 7.705634 | 8.417124 | 8.5927 | 6.868292 | 8.12777 | 7.880553 | 0.2632 |
| bta-miR-190a | 9.595342 | 9.900099 | 9.107391 | 10.02386 | 10.14735 | 10.01969 | 0.0871 |
| bta-miR-181c | 11.86494 | 13.48751 | 10.93024 | 11.61166 | 12.82214 | 12.23603 | 0.8833 |
| bta-miR-190b | 4.996127 | 5.292887 | 4.577983 | 3.478425 | 5.880549 | 5.137879 | 0.8756 |
| bta-miR-181d | 6.850434 | 7.534533 | 7.718877 | 5.591824 | 7.181168 | 7.038709 | 0.2528 |
| bta-miR-191 | 2.667972 | 2.465989 | 2.722373 | 1.349711 | 3.086868 | 2.710254 | 0.6804 |
| bta-miR-182 | 6.791671 | 7.866995 | 7.84872 | 5.907662 | 8.059439 | 7.073035 | 0.5322 |
| bta-miR-192 | 9.516469 | 10.16513 | 9.556138 | 8.587098 | 8.946852 | 9.230847 | **0.0425** |
| bta-miR-183 | 7.7609 | 9.141015 | 8.477947 | 6.305752 | 8.710102 | 8.431201 | 0.4942 |
| bta-miR-185 | 7.798493 | 8.021853 | 7.157155 | 7.248461 | 7.310776 | 7.652276 | 0.4253 |
| bta-miR-193a-5p | 8.16892 | 8.421969 | 8.72787 | 6.256016 | 8.984762 | 8.891351 | 0.6859 |
| bta-miR-186 | 5.666333 | 5.704051 | 5.321752 | 5.265299 | 5.144419 | 5.088349 | **0.0397** |
| bta-miR-194 | 7.782418 | 7.552685 | 7.362674 | 7.252328 | 6.801315 | 7.477075 | 0.1701 |
| bta-miR-195 | 4.617475 | 5.016005 | 4.150314 | 3.242531 | 4.299162 | 4.094369 | 0.1549 |
| bta-miR-200c | -0.47534 | 0.180125 | 0.02494 | -1.77444 | -0.04471 | -0.53054 | 0.2774 |
| bta-miR-202 | 9.835101 | 9.364703 | 9.039592 | 8.123553 | 8.841034 | 9.734458 | 0.3794 |
| bta-miR-196b | 9.75085 | 12.58412 | 12.0594 | 10.2894 | 11.01683 | 11.32587 | 0.5590 |
| bta-miR-204 | 3.602443 | 3.717791 | 3.843686 | 2.248456 | 3.514757 | 4.098376 | 0.4744 |
| bta-miR-197 | 5.483346 | 5.49701 | 6.093912 | 4.068701 | 5.489664 | 5.296071 | 0.2043 |
| bta-miR-205 | 7.568263 | 8.442936 | 9.087009 | 7.610861 | 5.521452 | 7.04608 | 0.0982 |
| bta-miR-199a-3p | 6.791068 | 7.5448 | 10.10825 | 6.277922 | 6.261404 | 8.0822 | 0.3380 |
| bta-miR-206 | 13.04464 | 13.12702 | 14.28164 | 12.54882 | 13.49288 | 14.16206 | 0.8990 |
| bta-miR-199a-5p | 13.69724 | 12.95925 | 15.00907 | 12.56866 | 10.79499 | 13.50682 | 0.1837 |
| bta-miR-199c | 6.336116 | 6.791163 | 9.427719 | 5.485499 | 5.604649 | 7.094384 | 0.2538 |
| bta-miR-20a | 5.709094 | 6.139279 | 5.736732 | 5.260811 | 5.712469 | 5.396968 | 0.1038 |
| bta-miR-19a | 6.633136 | 6.910985 | 6.742391 | 7.257266 | 6.812296 | 6.446612 | 0.7729 |
| bta-miR-20b | 7.590354 | 7.495923 | 7.360698 | 7.272442 | 7.288988 | 7.186023 | **0.0343** |
| bta-miR-19b | 6.715774 | 6.765019 | 6.590709 | 7.000804 | 6.488607 | 6.290944 | 0.6789 |
| bta-miR-21-3p | 14.05192 | 12.475 | 12.10068 | 11.69803 | 13.36714 | 14.66014 | 0.7439 |
| bta-miR-200a | 6.063376 | 6.613152 | 6.273824 | 5.824778 | 6.249947 | 6.241435 | 0.3770 |
| bta-miR-21-5p | 7.105353 | 8.184441 | 7.287528 | 6.287842 | 7.046015 | 7.1822 | 0.1888 |
| bta-miR-200b | -2.39428 | -1.63084 | -1.86586 | -3.48785 | -2.02786 | -2.44529 | 0.2313 |
| bta-miR-210 | 8.794599 | 8.70843 | 8.405443 | 8.250804 | 8.295727 | 8.136901 | **0.0325** |
| bta-miR-211 | 4.451064 | 4.409524 | 4.709934 | 2.319464 | 3.465157 | 4.871602 | 0.2617 |
| bta-miR-22-5p | 7.611852 | 8.275225 | 8.00225 | 6.489533 | 7.851028 | 7.093697 | 0.1353 |
| bta-miR-221 | 6.351449 | 7.162781 | 4.948736 | 4.372502 | 6.503683 | 6.18191 | 0.6398 |
| bta-miR-222 | 6.75174 | 7.125237 | 4.469825 | 4.332827 | 6.308071 | 6.108096 | 0.6357 |
| bta-miR-215 | 8.83846 | 9.14322 | 8.891467 | 8.054566 | 8.890598 | 8.996377 | 0.3764 |
| bta-miR-223 | 9.853538 | 10.80155 | 9.674573 | 8.329907 | 9.334555 | 10.01868 | 0.2169 |
| bta-miR-224 | 6.702703 | 7.092955 | 7.586704 | 5.309844 | 7.91057 | 6.939475 | 0.6376 |
| bta-miR-23a | -0.23056 | 0.459809 | 0.365154 | -1.32644 | 0.305039 | -0.03862 | 0.3661 |
| bta-miR-23b-3p | 2.356233 | 3.161372 | 3.113101 | 1.220069 | 3.013449 | 2.762584 | 0.4280 |
| bta-miR-218 | 9.257287 | 9.522938 | 8.87046 | 8.279776 | 10.13095 | 9.309901 | 0.9692 |
| bta-miR-23b-5p | 11.88884 | 13.34525 | 12.18149 | 11.01982 | 12.23339 | 12.60382 | 0.4709 |
| bta-miR-219-3p | 8.224544 | 9.102631 | 9.00077 | 8.176404 | 9.24214 | 9.85123 | 0.6065 |
| bta-miR-24-3p | 2.822959 | 3.05058 | 2.888036 | 2.211421 | 2.697897 | 2.787946 | 0.1375 |
| bta-miR-25 | 2.763907 | 3.445628 | 3.432813 | 1.933774 | 3.319048 | 3.03291 | 0.3981 |
| bta-miR-22-3p | -18.2088 | -17.8284 | -18.2024 | -19.7035 | -17.8238 | -17.9125 | 0.5570 |
| bta-miR-26a | -0.42233 | -0.27994 | -0.39492 | -1.61929 | -0.59868 | -0.63789 | 0.1566 |
| bta-miR-26b | 0.257333 | 0.625229 | 0.72619 | -1.24749 | 0.367643 | 0.200356 | 0.2251 |
| bta-miR-29d-3p | 1.957015 | 1.871808 | 1.278142 | 0.712258 | 1.501602 | 1.539622 | 0.2599 |
| bta-miR-29d-5p | 9.308381 | 8.530068 | 7.87449 | 7.295355 | 8.147854 | 8.371676 | 0.2973 |
| bta-miR-27a-3p | 3.748609 | 3.391341 | 3.355571 | 3.482306 | 3.57897 | 3.760354 | 0.5078 |
| bta-miR-29e | 12.65453 | 12.64819 | 11.38868 | 12.99933 | 11.58405 | 11.26307 | 0.6998 |
| bta-miR-27a-5p | 15.27923 | 12.54086 | 11.17645 | 10.789 | 12.38586 | 12.66342 | 0.4761 |
| bta-miR-27b | 3.725251 | 4.080929 | 4.442474 | 2.898156 | 3.56428 | 3.638701 | 0.0845 |
| bta-miR-28 | 8.625113 | 8.462901 | 8.858301 | 7.411669 | 7.951702 | 8.146514 | **0.0306** |
| bta-miR-296-3p | 8.253835 | 8.983847 | 10.08742 | 7.270764 | 9.305233 | 8.94756 | 0.5060 |
| bta-miR-296-5p | 10.66868 | 10.45125 | 10.38125 | 9.317898 | 9.960854 | 9.756024 | **0.0169** |
| bta-miR-29a | -0.48915 | -0.03382 | -0.25121 | -1.48166 | -0.7311 | -0.53071 | 0.1078 |
| bta-miR-29b | 10.26993 | 9.438336 | 9.489019 | 8.564574 | 9.23785 | 9.247874 | 0.1115 |
| bta-miR-30a-5p | 6.189916 | 6.097872 | 5.789558 | 5.292966 | 5.520737 | 5.658364 | **0.0294** |
| bta-miR-29c | -0.65381 | -0.27892 | -0.32911 | -1.68458 | -0.68671 | -0.37945 | 0.2938 |
| bta-miR-30b-3p | 10.2445 | 10.73278 | 10.10119 | 7.34784 | 10.60309 | 9.888661 | 0.3437 |
| bta-miR-30b-5p | 4.016 | 4.120818 | 3.40973 | 3.330708 | 3.290714 | 3.500241 | 0.1087 |
| bta-miR-328 | 9.173272 | 7.202376 | 8.255618 | 6.912718 | 7.305767 | 6.476725 | 0.1009 |
| bta-miR-30c | 2.734138 | 3.097594 | 2.556579 | 1.901742 | 2.422821 | 2.621713 | 0.1465 |
| bta-miR-30d | 6.296201 | 6.254281 | 5.416284 | 5.305119 | 5.553543 | 5.700114 | 0.2033 |
| bta-miR-30e-5p | 6.133832 | 6.294758 | 5.494893 | 5.605797 | 5.77277 | 5.542972 | 0.2584 |
| bta-miR-330 | 13.11448 | 12.45263 | 12.15894 | 13.17513 | 12.14251 | 13.36757 | 0.5368 |
| bta-miR-30f | 4.610293 | 4.858435 | 4.09418 | 3.897696 | 4.168215 | 4.340947 | 0.2116 |
| bta-miR-331-3p | 8.766389 | 9.414858 | 8.699653 | 6.091933 | 8.279287 | 8.775842 | 0.2194 |
| bta-miR-31 | 3.002374 | 3.479563 | 3.255655 | 2.705102 | 3.141035 | 3.221026 | 0.3501 |
| bta-miR-331-5p | 8.522555 | 9.149098 | 8.948512 | 7.857825 | 9.227329 | 9.164395 | 0.8108 |
| bta-miR-335 | 8.817826 | 10.10871 | 8.029214 | 10.86719 | 8.080793 | 9.06729 | 0.7457 |
| bta-miR-320a | 4.518819 | 5.355746 | 5.12185 | 2.941613 | 5.309673 | 4.94847 | 0.4842 |
| bta-miR-338 | 13.19093 | 11.32336 | 10.01563 | 10.71396 | 10.39749 | 10.86987 | 0.4135 |
| bta-miR-339a | 6.948821 | 7.312199 | 6.944728 | 6.219826 | 6.959591 | 7.114495 | 0.3708 |
| bta-miR-323 | -5.2744 | -4.6691 | -4.881 | -6.47588 | -4.08838 | -4.39126 | 0.9575 |
| bta-miR-339b | 6.736081 | 6.801528 | 6.063487 | 6.002049 | 6.204981 | 6.578816 | 0.4019 |
| bta-miR-324 | 9.828362 | 9.818715 | 10.64694 | 9.10217 | 9.471275 | 9.915244 | 0.1712 |
| bta-miR-326 | 12.13818 | 11.59761 | 14.41823 | 10.41459 | 11.56798 | 12.19566 | 0.2595 |
| bta-miR-33b | 12.28352 | 12.60393 | 13.30357 | 11.9516 | 15.47146 | 14.76386 | 0.2987 |
| bta-miR-340 | 10.09411 | 11.00488 | 10.20856 | 8.506339 | 11.22563 | 9.680531 | 0.4929 |
| bta-miR-365-3p | 5.017211 | 4.999428 | 5.971593 | 3.525033 | 4.695229 | 4.512545 | 0.0889 |
| bta-miR-342 | 7.223161 | 7.568062 | 7.930648 | 6.766715 | 7.581707 | 7.51769 | 0.4386 |
| bta-miR-365-5p | 14.64637 | 14.01295 | 13.89201 | 10.63417 | 12.17808 | 12.15036 | **0.0108** |
| bta-miR-345-3p | 9.371299 | 9.757783 | 10.01872 | 7.785796 | 9.66641 | 9.319592 | 0.2624 |
| bta-miR-369-3p | 11.36245 | 12.95933 | 13.63027 | 10.74481 | 13.04416 | 13.3579 | 0.8132 |
| bta-miR-34a | 5.357274 | 5.697754 | 6.107812 | 5.346141 | 5.760262 | 5.84213 | 0.8014 |
| bta-miR-34b | 5.021716 | 4.975718 | 4.140537 | 4.884041 | 4.275307 | 4.701659 | 0.7985 |
| bta-miR-34c | 4.955005 | 4.977719 | 4.337448 | 4.586949 | 4.190998 | 4.480565 | 0.2340 |
| bta-miR-374a | 5.418345 | 6.094426 | 5.623819 | 5.000966 | 5.430847 | 5.169423 | 0.0959 |
| bta-miR-361 | 5.334367 | 6.226658 | 6.003921 | 2.321213 | 5.865725 | 5.652604 | 0.3515 |
| bta-miR-374b | 3.789855 | 4.559187 | 4.096612 | 2.900077 | 4.098002 | 3.895469 | 0.2977 |
| bta-miR-375 | 3.394494 | 3.3322 | 3.675582 | 1.281767 | 2.976419 | 3.226195 | 0.1917 |
| bta-miR-362-5p | 11.31131 | 12.57104 | 10.92657 | 10.34203 | 11.56742 | 11.17929 | 0.4034 |
| bta-miR-378 | 7.773622 | 8.269103 | 8.389985 | 6.857569 | 8.262306 | 8.008439 | 0.4087 |
| bta-miR-378b | 7.82738 | 8.297679 | 8.072358 | 7.339795 | 8.967104 | 8.011575 | 0.9385 |
| bta-miR-378c | 10.72954 | 10.53061 | 10.87492 | 9.471051 | 12.02069 | 10.42391 | 0.9270 |
| bta-miR-378d | 11.58809 | 11.68516 | 11.63767 | 11.88221 | 13.34205 | 12.43081 | 0.0987 |
| bta-miR-421 | 5.631049 | 5.595049 | 6.496208 | 4.86444 | 7.503883 | 6.837678 | 0.5900 |
| bta-miR-423-3p | 6.171523 | 6.626698 | 6.813514 | 5.451205 | 6.454752 | 6.175279 | 0.2237 |
| bta-miR-423-5p | 4.850791 | 5.536217 | 6.195455 | 4.077311 | 5.895271 | 5.182692 | 0.5085 |
| bta-miR-449c | 9.731004 | 9.55341 | 11.42038 | 9.209319 | 11.58352 | 10.38735 | 0.8699 |
| bta-miR-424-3p | 11.81463 | 11.53438 | 10.90143 | 11.50197 | 12.60313 | 11.30946 | 0.4686 |
| bta-miR-449d | 11.5567 | 11.62142 | 12.0094 | 10.45136 | 10.6855 | 12.15702 | 0.3168 |
| bta-miR-424-5p | 6.55956 | 7.198752 | 5.030576 | 5.663058 | 6.969181 | 5.604167 | 0.8255 |
| bta-miR-450a | 9.802566 | 11.36946 | 9.359458 | 9.413947 | 11.10705 | 10.10073 | 0.9712 |
| bta-miR-450b | 10.30254 | 12.02315 | 9.169435 | 8.137387 | 11.3507 | 10.1086 | 0.6394 |
| bta-miR-425-5p | 7.320157 | 7.404872 | 6.846038 | 6.508801 | 7.027506 | 6.872512 | 0.1704 |
| bta-miR-451 | 9.754111 | 10.14227 | 13.80425 | 8.365565 | 9.157499 | 9.045533 | 0.1446 |
| bta-miR-429 | 3.558394 | 3.850819 | 3.431362 | 3.491009 | 3.540848 | 3.175057 | 0.2793 |
| bta-miR-453 | 12.03793 | 12.53639 | 12.67309 | 11.43785 | 13.25052 | 12.90821 | 0.8529 |
| bta-miR-433 | 7.556252 | 7.837981 | 7.524665 | 7.302979 | 8.986443 | 9.0292 | 0.2380 |
| bta-miR-454 | 12.02074 | 10.40735 | 10.46161 | 8.181786 | 11.17678 | 10.16509 | 0.3358 |
| bta-miR-455-3p | 8.738175 | 9.439183 | 9.585046 | 8.40815 | 8.741982 | 9.645663 | 0.5160 |
| bta-miR-449a | 3.822822 | 4.057034 | 5.104501 | 3.234034 | 5.199415 | 4.484771 | 0.9763 |
| bta-miR-455-5p | 10.78429 | 11.5894 | 11.08457 | 11.67449 | 10.63552 | 13.19529 | 0.4308 |
| bta-miR-449b | 5.848422 | 6.319052 | 7.534467 | 5.214974 | 7.697748 | 6.973973 | 0.9483 |
| bta-miR-484 | 8.379521 | 8.475962 | 8.490985 | 7.341483 | 8.260044 | 8.682364 | 0.4231 |
| bta-miR-485 | 12.87615 | 12.58848 | 12.07602 | 11.50171 | 14.9145 | 13.29554 | 0.5145 |
| bta-miR-497 | 8.962152 | 9.420386 | 9.033563 | 8.10172 | 10.12062 | 9.4903 | 0.8797 |
| bta-miR-486 | 8.004127 | 8.447638 | 8.677207 | 7.654208 | 8.718218 | 9.10533 | 0.8193 |
| bta-miR-499 | 10.91901 | 9.590022 | 9.751634 | 8.531211 | 9.294228 | 9.836017 | 0.1995 |
| bta-miR-500 | 9.715467 | 9.521489 | 9.36214 | 7.302811 | 8.954811 | 9.093882 | 0.1375 |
| bta-miR-502a | 12.70024 | 14.70059 | 12.29136 | 10.43428 | 14.48105 | 13.78278 | 0.8309 |
| bta-miR-502b | 8.869939 | 10.00977 | 10.01663 | 7.897471 | 9.635557 | 9.063834 | 0.2957 |
| bta-miR-489 | 9.628812 | 10.34882 | 9.087676 | 9.727723 | 11.7776 | 11.90982 | 0.1423 |
| bta-miR-503-3p | 10.26737 | 10.10245 | 10.98999 | 9.518477 | 11.08982 | 11.04549 | 0.8749 |
| bta-miR-491 | 7.564424 | 8.093924 | 8.056672 | 5.221011 | 7.928236 | 7.14943 | 0.2384 |
| bta-miR-493 | 11.55699 | 10.9886 | 12.1195 | 11.83807 | 10.31236 | 11.53042 | 0.5950 |
| bta-miR-494 | 6.738244 | 6.492917 | 7.079395 | 6.159207 | 7.201214 | 7.214686 | 0.8317 |
| bta-miR-532 | 11.09519 | 10.94959 | 11.16328 | 10.65834 | 10.16268 | 9.376082 | 0.0569 |
| bta-miR-495 | 11.90181 | 12.89914 | 12.89399 | 12.34646 | 12.38926 | 13.59249 | 0.7087 |
| bta-miR-584 | 11.34574 | 11.26745 | 12.14259 | 10.88976 | 12.79128 | 13.5135 | 0.3833 |
| bta-miR-628 | 10.85224 | 11.38554 | 11.18168 | 9.979291 | 11.24648 | 11.28718 | 0.5443 |
| bta-miR-631 | -2.52009 | -2.24706 | -2.35921 | -3.77232 | -1.74366 | -1.95297 | 0.8689 |
| bta-miR-551a | 16.718 | 18.03275 | 15.89081 | 18.10016 | 17.67995 | 19.83067 | 0.1417 |
| bta-miR-652 | 6.699313 | 6.650698 | 6.065015 | 4.920191 | 6.445282 | 6.498006 | 0.4050 |
| bta-miR-654 | 10.85642 | 11.70439 | 11.55074 | 9.316033 | 12.58001 | 13.26485 | 0.7928 |
| bta-miR-574 | 6.807334 | 6.558814 | 7.259492 | 5.333838 | 5.843565 | 6.621928 | 0.0920 |
| bta-miR-656 | 9.2888 | 9.457944 | 9.620752 | 8.62435 | 11.05886 | 10.18243 | 0.5252 |
| bta-miR-660 | 7.971335 | 7.90569 | 7.169874 | 6.138308 | 7.477373 | 7.157269 | 0.1884 |
| bta-miR-664a | 10.82934 | 10.48487 | 10.51131 | 9.859707 | 11.24731 | 11.00771 | 0.8381 |
| bta-miR-760-3p | 12.339 | 11.64805 | 12.21755 | 10.23851 | 10.99815 | 12.06922 | 0.1665 |
| bta-miR-664b | 3.665677 | 3.848155 | 3.63243 | 2.277445 | 3.322199 | 3.182126 | 0.0778 |
| bta-miR-665 | 8.879533 | 9.089121 | 9.425968 | 9.008702 | 9.112637 | 9.270551 | 0.9961 |
| bta-miR-761 | 12.80004 | 14.29878 | 13.01652 | 15.77387 | 13.31119 | 17.54101 | 0.1736 |
| bta-miR-669 | 9.744294 | 9.548213 | 8.997495 | 7.589114 | 8.231897 | 10.23474 | 0.4190 |
| bta-miR-763 | 13.25446 | 13.55987 | 12.82568 | 13.43759 | 14.83698 | 13.26247 | 0.3080 |
| bta-miR-764 | 11.86199 | 11.96511 | 12.60858 | 11.58466 | 13.60625 | 14.8785 | 0.2871 |
| bta-miR-671 | 12.46265 | 13.5199 | 14.31529 | 10.9595 | 12.30797 | 12.65171 | 0.1216 |
| bta-miR-767 | 9.41221 | 10.35706 | 10.30791 | 8.987918 | 11.19835 | 11.27352 | 0.5998 |
| bta-miR-677 | 9.648664 | 9.631325 | 10.29298 | 9.006171 | 10.01351 | 10.0807 | 0.7204 |
| bta-miR-769 | 10.40995 | 12.54076 | 11.37106 | 9.998288 | 11.31208 | 11.1102 | 0.4396 |
| bta-miR-7 | 5.41571 | 6.409682 | 6.08649 | 3.989897 | 5.590059 | 5.473842 | 0.1831 |
| bta-miR-708 | 8.257844 | 8.127245 | 8.865819 | 6.734326 | 8.633329 | 7.927642 | 0.3377 |
| bta-miR-874 | 10.08578 | 10.37512 | 10.04527 | 8.84089 | 10.01436 | 9.721263 | 0.1550 |
| bta-miR-744 | 5.930629 | 6.992056 | 6.611841 | 5.162875 | 6.902435 | 6.897639 | 0.7862 |
| bta-miR-98 | 4.456887 | 5.284974 | 5.034805 | 3.179478 | 5.183373 | 4.118491 | 0.2905 |
| bta-miR-99a-3p | 10.77608 | 12.33601 | 12.01367 | 11.0135 | 11.5072 | 11.28267 | 0.4247 |
| bta-miR-885 | 3.99133 | 3.741908 | 3.909133 | 2.350375 | 3.41563 | 3.485291 | 0.1004 |
| bta-miR-99a-5p | 4.014935 | 4.222853 | 3.976694 | 3.985146 | 3.307646 | 3.555118 | 0.0983 |
| bta-miR-9-3p | 11.83178 | 11.03754 | 12.20527 | 12.77955 | 11.60473 | 13.20247 | 0.2282 |
| bta-miR-9-5p | 4.548188 | 4.312962 | 4.793363 | 3.0427 | 3.617445 | 5.420522 | 0.5119 |
| bta-miR-92a | 2.796199 | 3.612198 | 3.565219 | 2.244271 | 3.426226 | 3.223222 | 0.4692 |
| bta-miR-92b | 2.606015 | 2.825274 | 2.703485 | 1.244496 | 2.743364 | 2.658521 | 0.3688 |
| bta-miR-93 | 5.81737 | 6.666306 | 6.07596 | 5.269581 | 6.252794 | 6.005376 | 0.4251 |
| bta-miR-940 | 5.289645 | 5.376177 | 5.508874 | 4.438143 | 5.28981 | 5.302211 | 0.2628 |
| bta-miR-1224 | 8.863509 | 9.087712 | 10.40271 | 7.714696 | 9.13662 | 9.139422 | 0.3080 |
| bta-miR-95 | 7.27937 | 8.49578 | 9.114004 | 7.839528 | 8.317492 | 8.9849 | 0.9005 |
| bta-miR-1225-3p | 7.171399 | 7.223744 | 7.112594 | 6.330517 | 7.02466 | 7.460498 | 0.5237 |
| bta-miR-1296 | 10.84594 | 12.53263 | 13.14261 | 10.04705 | 10.64211 | 12.03753 | 0.2349 |
| bta-miR-1247-5p | 9.149631 | 9.391488 | 9.343318 | 8.779523 | 8.77894 | 9.195201 | 0.0744 |
| bta-miR-1249 | 6.178208 | 6.633202 | 7.048063 | 4.62023 | 6.491089 | 5.936585 | 0.1987 |
| bta-miR-1306 | 7.228688 | 7.46913 | 7.374322 | 6.058217 | 7.311533 | 7.590869 | 0.4802 |
| bta-miR-1260b | 1.039008 | 1.307196 | 1.533065 | 0.276944 | 1.164446 | 1.15422 | 0.2608 |
| bta-miR-1271 | 13.77235 | 13.7635 | 14.05845 | 11.91673 | 11.76174 | 11.25932 | **0.0006** |
| bta-miR-1343-5p | 7.779841 | 8.566683 | 8.875038 | 7.266026 | 7.598217 | 8.35278 | 0.2183 |
| bta-miR-1281 | 9.648555 | 9.686188 | 9.842166 | 9.131419 | 9.207657 | 9.373164 | **0.0062** |
| bta-miR-1388-3p | 9.715383 | 9.527745 | 10.01165 | 7.921414 | 10.8377 | 10.044 | 0.8727 |
| RNT43 snoRNA | 2.855496 | 2.609049 | 3.066083 | 4.101084 | 3.060717 | 3.07343 | 0.1986 |
| Hm/Ms/Rt T1 snRNA | -3.85053 | -3.8285 | -4.11174 | -3.68292 | -4.34564 | -4.26635 | 0.5018 |
| bta-miR-99b | 1.487073 | 1.799285 | 1.706603 | 0.278105 | 2.130096 | 1.889638 | 0.7140 |
| bta-miR-1291 | 14.35359 | 14.50239 | 12.12416 | 12.3064 | 10.80065 | 15.02783 | 0.5505 |
| ^1^Body energy reserve: MBER: Cows with moderated body energy reserve; HBER: Cows with high body energy reserve; ^2^P-value: P value between animals with different body energy reserve. | | | | | | | |
