# Supplementary material for: The bovine oviductal environment and composition are negatively affected by elevated body energy reserves
Source: PLoS One. 2025 Jun 23;20(6):e0326138. doi: 10.1371/journal.pone.0326138 (PMC12184905; doi:10.1371/journal.pone.0326138)
Supplement: S12 Table — (DOCX) [file pone.0326138.s015.docx]

| **Supplementary table 12.** Biological patwhays predicted as modulated by exclusive miRNAs in ampullary luminal epithelial cells (AMP-Cell) in high body energy reserve (HBER) group. | | |
| --- | --- | --- |
| **Pathway** | **%^1^** | **BH^2^** |
| bta04144 Endocytosis | 52.65 | 0.0 |
| bta05200 Pathways in cancer | 44.28 | 0.0 |
| bta04010 MAPK signaling pathway | 47.26 | 0.0052 |
| bta04014 Ras signaling pathway | 50.00 | 0.0052 |
| bta04514 Cell adhesion molecules (CAMs) | 53.16 | 0.0052 |
| bta05205 Proteoglycans in cancer | 50.24 | 0.0052 |
| bta04810 Regulation of actin cytoskeleton | 48.34 | 0.018 |
| bta01100 Metabolic pathways | 37.46 | 0.0315 |
| bta04360 Axon guidance | 48.88 | 0.0315 |
| bta04310 Wnt signaling pathway | 48.15 | 0.0492 |
| bta04390 Hippo signaling pathway | 48.08 | 0.0492 |
| bta04659 Th17 cell differentiation | 52.21 | 0.0492 |
| bta04910 Insulin signaling pathway | 49.29 | 0.0492 |
| bta04921 Oxytocin signaling pathway | 48.68 | 0.0492 |
| bta04922 Glucagon signaling pathway | 52.43 | 0.0492 |
| bta05212 Pancreatic cancer | 56.58 | 0.0492 |
| bta04015 Rap1 signaling pathway | 44.91 | 0.0501 |
| bta04120 Ubiquitin mediated proteolysis | 48.57 | 0.0501 |
| bta04530 Tight junction | 46.33 | 0.0501 |
| bta04658 Th1 and Th2 cell differentiation | 52.04 | 0.0501 |
| bta04670 Leukocyte transendothelial migration | 50.44 | 0.0501 |
| bta05163 Human cytomegalovirus infection | 44.08 | 0.0501 |
| bta00564 Glycerophospholipid metabolism | 50.96 | 0.0525 |
| bta01212 Fatty acid metabolism | 58.62 | 0.0525 |
| bta04062 Chemokine signaling pathway | 45.21 | 0.0572 |
| bta04270 Vascular smooth muscle contraction | 48.12 | 0.0572 |
| bta04750 Inflammatory mediator regulation of TRP channels | 50.49 | 0.0572 |
| bta04022 cGMP-PKG signaling pathway | 45.56 | 0.0663 |
| bta04371 Apelin signaling pathway | 47.14 | 0.0663 |
| bta04024 cAMP signaling pathway | 43.23 | 0.0699 |
| bta04668 TNF signaling pathway | 48.31 | 0.0699 |
| bta04912 GnRH signaling pathway | 50.54 | 0.0699 |
| bta04925 Aldosterone synthesis and secretion | 50.00 | 0.0725 |
| bta04068 FoxO signaling pathway | 46.56 | 0.0767 |
| bta04072 Phospholipase D signaling pathway | 45.39 | 0.0767 |
| bta05132 Salmonella infection | 42.86 | 0.0767 |
| bta05160 Hepatitis C | 45.12 | 0.0767 |
| bta05214 Glioma | 51.95 | 0.0767 |
| bta05220 Chronic myeloid leukemia | 51.95 | 0.0767 |
| bta01522 Endocrine resistance | 48.94 | 0.0784 |
| bta04070 Phosphatidylinositol signaling system | 48.48 | 0.0784 |
| bta04261 Adrenergic signaling in cardiomyocytes | 45.33 | 0.0784 |
| bta04625 C-type lectin receptor signaling pathway | 48.11 | 0.0784 |
| bta04722 Neurotrophin signaling pathway | 46.72 | 0.0784 |
| bta05211 Renal cell carcinoma | 52.11 | 0.0784 |
| bta05235 PD-L1 expression and PD-1 checkpoint pathway in cancer | 49.46 | 0.0784 |
| bta05414 Dilated cardiomyopathy (DCM) | 48.48 | 0.0784 |
| bta04660 T cell receptor signaling pathway | 47.66 | 0.0788 |
| bta04931 Insulin resistance | 47.27 | 0.081 |
| bta05167 Kaposi sarcoma-associated herpesvirus infection | 42.72 | 0.0813 |
| bta00562 Inositol phosphate metabolism | 50.68 | 0.0886 |
| bta04934 Cushing syndrome | 44.23 | 0.0886 |
| bta05100 Bacterial invasion of epithelial cells | 50.68 | 0.0886 |
| bta00071 Fatty acid degradation | 57.14 | 0.0901 |
| bta01200 Carbon metabolism | 46.02 | 0.0901 |
| bta04012 ErbB signaling pathway | 48.81 | 0.0901 |
| bta04020 Calcium signaling pathway | 42.08 | 0.0901 |
| bta04066 HIF-1 signaling pathway | 46.36 | 0.0901 |
| bta04130 SNARE interactions in vesicular transport | 60.61 | 0.0901 |
| bta04728 Dopaminergic synapse | 44.78 | 0.0901 |
| bta04916 Melanogenesis | 47.06 | 0.0901 |
| bta04924 Renin secretion | 50.00 | 0.0901 |
| bta05170 Human immunodeficiency virus 1 infection | 41.45 | 0.0901 |
| bta05410 Hypertrophic cardiomyopathy (HCM) | 47.83 | 0.0901 |
| bta05418 Fluid shear stress and atherosclerosis | 44.14 | 0.0901 |
| bta04142 Lysosome | 44.70 | 0.0903 |
| bta04710 Circadian rhythm | 61.29 | 0.0903 |
| bta04911 Insulin secretion | 48.24 | 0.0903 |
| bta04137 Mitophagy | 50.00 | 0.0996 |
| bta04218 Cellular senescence | 42.77 | 0.0996 |
| bta04550 Signaling pathways regulating pluripotency of stem cells | 43.66 | 0.0996 |
| bta04971 Gastric acid secretion | 48.68 | 0.0996 |
| bta05202 Transcriptional misregulation in cancer | 41.88 | 0.0996 |
| bta05412 Arrhythmogenic right ventricular cardiomyopathy (ARVC) | 48.68 | 0.0996 |
| bta04611 Platelet activation | 44.63 | 0.1025 |
| bta00010 Glycolysis Gluconeogenesis | 50.00 | 0.1035 |
| bta00600 Sphingolipid metabolism | 53.06 | 0.1035 |
| bta05219 Bladder cancer | 54.76 | 0.1074 |
| bta04064 NF-kappa B signaling pathway | 44.95 | 0.1114 |
| bta05225 Hepatocellular carcinoma | 41.95 | 0.1114 |
| bta05321 Inflammatory bowel disease (IBD) | 48.57 | 0.112 |
| bta05224 Breast cancer | 42.67 | 0.1149 |
| bta01230 Biosynthesis of amino acids | 47.95 | 0.1158 |
| bta04510 Focal adhesion | 40.91 | 0.1241 |
| bta05230 Central carbon metabolism in cancer | 48.48 | 0.1249 |
| bta04926 Relaxin signaling pathway | 43.08 | 0.1274 |
| bta05161 Hepatitis B | 41.52 | 0.1274 |
| bta04928 Parathyroid hormone synthesis. secretion and action | 44.23 | 0.1392 |
| bta05218 Melanoma | 46.58 | 0.1511 |
| bta04150 mTOR signaling pathway | 41.40 | 0.1522 |
| bta04961 Endocrine and other factor-regulated calcium reabsorption | 50.00 | 0.1523 |
| bta04713 Circadian entrainment | 44.00 | 0.1537 |
| bta00310 Lysine degradation | 46.97 | 0.1585 |
| bta00640 Propanoate metabolism | 54.55 | 0.1585 |
| bta05166 Human T-cell leukemia virus 1 infection | 39.32 | 0.1585 |
| bta05215 Prostate cancer | 43.88 | 0.1585 |
| bta04115 p53 signaling pathway | 45.45 | 0.162 |
| bta04152 AMPK signaling pathway | 42.28 | 0.162 |
| bta04720 Long-term potentiation | 46.38 | 0.162 |
| bta01521 EGFR tyrosine kinase inhibitor resistance | 45.00 | 0.1634 |
| bta04920 Adipocytokine signaling pathway | 45.83 | 0.1634 |
| bta04215 Apoptosis | 52.94 | 0.1711 |
| bta04392 Hippo signaling pathway | 55.17 | 0.1711 |
| bta04520 Adherens junction | 45.71 | 0.1711 |
| bta05135 Yersinia infection | 41.54 | 0.1746 |
| bta05221 Acute myeloid leukemia | 45.59 | 0.181 |
| bta04071 Sphingolipid signaling pathway | 41.67 | 0.1878 |
| bta04330 Notch signaling pathway | 47.17 | 0.1963 |
| bta04919 Thyroid hormone signaling pathway | 41.53 | 0.1964 |
| bta05031 Amphetamine addiction | 44.93 | 0.1964 |
| bta05231 Choline metabolism in cancer | 42.42 | 0.1989 |
| bta00280 Valine. leucine and isoleucine degradation | 47.06 | 0.2047 |
| bta00561 Glycerolipid metabolism | 44.78 | 0.2047 |
| bta05223 Non-small cell lung cancer | 44.78 | 0.2047 |
| bta05226 Gastric cancer | 39.87 | 0.2104 |
| bta00512 Mucin type O-glycan biosynthesis | 51.61 | 0.2138 |
| bta05032 Morphine addiction | 42.39 | 0.2138 |
| bta00230 Purine metabolism | 40.30 | 0.2147 |
| bta04061 Viral protein interaction with cytokine and cytokine receptor | 42.11 | 0.2147 |
| bta04621 NOD-like receptor signaling pathway | 38.59 | 0.2371 |
| bta04726 Serotonergic synapse | 40.52 | 0.2371 |
| bta05133 Pertussis | 42.86 | 0.2371 |
| bta05145 Toxoplasmosis | 40.71 | 0.2371 |
| bta04140 Autophagy | 39.44 | 0.2467 |
| bta04340 Hedgehog signaling pathway | 45.10 | 0.25 |
| bta04380 Osteoclast differentiation | 39.55 | 0.25 |
| bta04657 IL-17 signaling pathway | 41.30 | 0.25 |
| bta04933 AGE-RAGE signaling pathway in diabetic complications | 40.78 | 0.25 |
| bta05210 Colorectal cancer | 41.57 | 0.25 |
| bta04151 PI3K-Akt signaling pathway | 36.19 | 0.2518 |
| bta00270 Cysteine and methionine metabolism | 44.90 | 0.2589 |
| bta00650 Butanoate metabolism | 50.00 | 0.2589 |
| bta04350 TGF-beta signaling pathway | 40.86 | 0.2589 |
| bta04540 Gap junction | 41.11 | 0.2589 |
| bta04666 Fc gamma R-mediated phagocytosis | 40.86 | 0.2589 |
| bta04927 Cortisol synthesis and secretion | 43.08 | 0.2589 |
| bta04640 Hematopoietic cell lineage | 40.00 | 0.2598 |
| bta05217 Basal cell carcinoma | 42.86 | 0.2668 |
| bta04727 GABAergic synapse | 40.66 | 0.2693 |
| bta04978 Mineral absorption | 43.64 | 0.2693 |
| bta04614 Renin-angiotensin system | 50.00 | 0.2737 |
| bta05165 Human papillomavirus infection | 35.94 | 0.2806 |
| bta00062 Fatty acid elongation | 48.28 | 0.2859 |
| bta05164 Influenza A | 37.57 | 0.2859 |
| bta04940 Type I diabetes mellitus | 42.37 | 0.297 |
| bta04662 B cell receptor signaling pathway | 40.23 | 0.2971 |
| bta04724 Glutamatergic synapse | 38.94 | 0.3006 |
| bta04970 Salivary secretion | 39.78 | 0.3006 |
| bta05416 Viral myocarditis | 40.79 | 0.3006 |
| bta04114 Oocyte meiosis | 38.66 | 0.3045 |
| bta00620 Pyruvate metabolism | 44.74 | 0.3075 |
| bta01040 Biosynthesis of unsaturated fatty acids | 46.67 | 0.3075 |
| bta04141 Protein processing in endoplasmic reticulum | 37.35 | 0.3075 |
| bta04730 Long-term depression | 41.67 | 0.3075 |
| bta04930 Type II diabetes mellitus | 43.48 | 0.3075 |
| bta03013 RNA transport | 37.08 | 0.311 |
| bta04973 Carbohydrate digestion and absorption | 43.18 | 0.3228 |
| bta00100 Steroid biosynthesis | 50.00 | 0.3229 |
| bta04370 VEGF signaling pathway | 41.38 | 0.3229 |
| bta05142 Chagas disease (American trypanosomiasis) | 38.26 | 0.3229 |
| bta05140 Leishmaniasis | 39.74 | 0.3277 |
| bta00052 Galactose metabolism | 45.16 | 0.3372 |
| bta00514 Other types of O-glycan biosynthesis | 42.22 | 0.3382 |
| bta01210 2-Oxocarboxylic acid metabolism | 50.00 | 0.3382 |
| bta03420 Nucleotide excision repair | 42.22 | 0.3382 |
| bta04211 Longevity regulating pathway | 38.89 | 0.3382 |
| bta05169 Epstein-Barr virus infection | 35.96 | 0.3382 |
| bta05213 Endometrial cancer | 40.68 | 0.3382 |
| bta04950 Maturity onset diabetes of the young | 46.15 | 0.3403 |
| bta04725 Cholinergic synapse | 37.72 | 0.3458 |
| bta00601 Glycosphingolipid biosynthesis | 44.83 | 0.3476 |
| bta03020 RNA polymerase | 44.83 | 0.3476 |
| bta05216 Thyroid cancer | 42.50 | 0.3476 |
| bta01523 Antifolate resistance | 41.86 | 0.3539 |
| bta00500 Starch and sucrose metabolism | 43.75 | 0.3548 |
| bta00534 Glycosaminoglycan biosynthesis | 45.83 | 0.3601 |
| bta04962 Vasopressin-regulated water reabsorption | 40.82 | 0.3601 |
| bta05152 Tuberculosis | 35.86 | 0.3601 |
| bta04744 Phototransduction | 44.44 | 0.3683 |
| bta01524 Platinum drug resistance | 38.46 | 0.3689 |
| bta00220 Arginine biosynthesis | 47.37 | 0.3696 |
| bta00380 Tryptophan metabolism | 40.43 | 0.3782 |
| bta04623 Cytosolic DNA-sensing pathway | 38.81 | 0.3782 |
| bta04664 Fc epsilon RI signaling pathway | 38.57 | 0.3782 |
| bta04972 Pancreatic secretion | 37.25 | 0.3782 |
| bta00760 Nicotinate and nicotinamide metabolism | 41.03 | 0.3905 |
| bta04914 Progesterone-mediated oocyte maturation | 37.50 | 0.3905 |
| bta05222 Small cell lung cancer | 37.23 | 0.3912 |
| bta00603 Glycosphingolipid biosynthesis | 47.06 | 0.3914 |
| bta00910 Nitrogen metabolism | 47.06 | 0.3914 |
| bta00030 Pentose phosphate pathway | 42.86 | 0.3917 |
| bta04975 Fat digestion and absorption | 39.58 | 0.3939 |
| bta05030 Cocaine addiction | 39.58 | 0.3939 |
| bta05332 Graft-versus-host disease | 39.58 | 0.3939 |
| bta04976 Bile secretion | 37.35 | 0.3966 |
| bta00051 Fructose and mannose metabolism | 41.18 | 0.3986 |
| bta03015 mRNA surveillance pathway | 36.84 | 0.3986 |
| bta05162 Measles | 35.53 | 0.4071 |
| bta00592 alpha-Linolenic acid metabolism | 41.38 | 0.4228 |
| bta00061 Fatty acid biosynthesis | 44.44 | 0.4252 |
| bta00730 Thiamine metabolism | 44.44 | 0.4252 |
| bta05020 Prion diseases | 40.63 | 0.4252 |
| bta05134 Legionellosis | 37.93 | 0.4252 |
| bta00900 Terpenoid backbone biosynthesis | 42.86 | 0.4332 |
| bta04210 Apoptosis | 35.21 | 0.4332 |
| bta04935 Growth hormone synthesis. secretion and action | 35.59 | 0.4332 |
| bta00630 Glyoxylate and dicarboxylate metabolism | 40.00 | 0.453 |
| bta04620 Toll-like receptor signaling pathway | 35.45 | 0.453 |
| bta03410 Base excision repair | 39.39 | 0.454 |
| bta00591 Linoleic acid metabolism | 38.89 | 0.4561 |
| bta04216 Ferroptosis | 37.78 | 0.4631 |
| bta00511 Other glycan degradation | 40.91 | 0.4696 |
| bta00531 Glycosaminoglycan degradation | 40.91 | 0.4696 |
| bta04721 Synaptic vesicle cycle | 35.90 | 0.4696 |
| bta04060 Cytokine-cytokine receptor interaction | 33.44 | 0.4806 |
| bta00410 beta-Alanine metabolism | 38.24 | 0.4834 |
| bta00250 Alanine. aspartate and glutamate metabolism | 37.84 | 0.4837 |
| bta00072 Synthesis and degradation of ketone bodies | 45.45 | 0.484 |
| bta00565 Ether lipid metabolism | 36.54 | 0.484 |
| bta04923 Regulation of lipolysis in adipocytes | 36.21 | 0.484 |
| bta00533 Glycosaminoglycan biosynthesis | 42.86 | 0.49 |
| bta00532 Glycosaminoglycan biosynthesis | 40.00 | 0.5022 |
| bta03430 Mismatch repair | 39.13 | 0.5041 |
| bta00563 Glycosylphosphatidylinositol (GPI)-anchor biosynthesis | 38.46 | 0.5048 |
| bta00520 Amino sugar and nucleotide sugar metabolism | 36.00 | 0.5061 |
| bta04110 Cell cycle | 34.15 | 0.5061 |
| bta04146 Peroxisome | 34.52 | 0.5231 |
| bta00330 Arginine and proline metabolism | 35.42 | 0.5371 |
| bta00670 One carbon pool by folate | 38.89 | 0.5371 |
| bta03030 DNA replication | 36.11 | 0.5371 |
| bta04650 Natural killer cell mediated cytotoxicity | 33.59 | 0.5371 |
| bta03018 RNA degradation | 34.18 | 0.5436 |
| bta04915 Estrogen signaling pathway | 33.33 | 0.55 |
| bta00510 N-Glycan biosynthesis | 34.62 | 0.5592 |
| bta00770 Pantothenate and CoA biosynthesis | 36.84 | 0.5766 |
| bta04217 Necroptosis | 32.76 | 0.5766 |
| bta04672 Intestinal immune network for IgA production | 33.93 | 0.5766 |
| bta04964 Proximal tubule bicarbonate reclamation | 36.36 | 0.5766 |
| bta05017 Spinocerebellar ataxia | 33.33 | 0.5766 |
| bta05330 Allograft rejection | 33.93 | 0.5766 |
| bta05014 Amyotrophic lateral sclerosis (ALS) | 33.33 | 0.6037 |
| bta04913 Ovarian steroidogenesis | 33.33 | 0.6053 |
| bta00340 Histidine metabolism | 34.78 | 0.6102 |
| bta00360 Phenylalanine metabolism | 34.78 | 0.6102 |
| bta00515 Mannose type O-glycan biosynthesis | 34.78 | 0.6102 |
| bta03060 Protein export | 34.78 | 0.6102 |
| bta00350 Tyrosine metabolism | 33.33 | 0.6195 |
| bta04917 Prolactin signaling pathway | 32.53 | 0.6195 |
| bta05203 Viral carcinogenesis | 31.95 | 0.6201 |
| bta04918 Thyroid hormone synthesis | 32.43 | 0.6248 |
| bta00020 Citrate cycle (TCA cycle) | 33.33 | 0.6315 |
| bta00260 Glycine. serine and threonine metabolism | 32.56 | 0.6414 |
| bta00240 Pyrimidine metabolism | 32.14 | 0.6482 |
| bta04960 Aldosterone-regulated sodium reabsorption | 32.43 | 0.6505 |
| bta04612 Antigen processing and presentation | 31.76 | 0.6524 |
| bta05143 African trypanosomiasis | 31.82 | 0.6674 |
| bta04966 Collecting duct acid secretion | 32.14 | 0.6693 |
| bta03440 Homologous recombination | 30.95 | 0.7052 |
| bta04723 Retrograde endocannabinoid signaling | 30.92 | 0.7052 |
| bta03460 Fanconi anemia pathway | 30.77 | 0.7074 |
| bta05323 Rheumatoid arthritis | 30.77 | 0.7074 |
| bta04213 Longevity regulating pathway | 30.65 | 0.7091 |
| bta00604 Glycosphingolipid biosynthesis | 31.25 | 0.7099 |
| bta00513 Various types of N-glycan biosynthesis | 30.23 | 0.7218 |
| bta04136 Autophagy | 30.30 | 0.7218 |
| bta00860 Porphyrin and chlorophyll metabolism | 30.00 | 0.7296 |
| bta05168 Herpes simplex virus 1 infection | 30.77 | 0.7299 |
| bta05204 Chemical carcinogenesis | 29.87 | 0.7428 |
| bta03022 Basal transcription factors | 29.55 | 0.7436 |
| bta03320 PPAR signaling pathway | 29.63 | 0.7512 |
| bta05340 Primary immunodeficiency | 29.27 | 0.7512 |
| bta00040 Pentose and glucuronate interconversions | 29.03 | 0.7513 |
| bta04512 ECM-receptor interaction | 29.21 | 0.7761 |
| bta00790 Folate biosynthesis | 27.78 | 0.7975 |
| bta05146 Amoebiasis | 29.06 | 0.7975 |
| bta00140 Steroid hormone biosynthesis | 28.36 | 0.7977 |
| bta04979 Cholesterol metabolism | 28.00 | 0.7977 |
| bta00830 Retinol metabolism | 28.13 | 0.8025 |
| bta04977 Vitamin digestion and absorption | 26.92 | 0.8025 |
| bta04630 JAK-STAT signaling pathway | 29.21 | 0.8143 |
| bta04929 GnRH secretion | 27.69 | 0.8175 |
| bta04145 Phagosome | 28.82 | 0.8241 |
| bta05310 Asthma | 26.32 | 0.8327 |
| bta04622 RIG-I-like receptor signaling pathway | 27.45 | 0.8593 |
| bta00053 Ascorbate and aldarate metabolism | 24.00 | 0.8655 |
| bta03040 Spliceosome | 27.89 | 0.8655 |
| bta04260 Cardiac muscle contraction | 26.97 | 0.8655 |
| bta04742 Taste transduction | 26.58 | 0.8655 |
| bta05033 Nicotine addiction | 25.00 | 0.8655 |
| bta05144 Malaria | 25.42 | 0.8818 |
| bta00982 Drug metabolism | 25.40 | 0.886 |
| bta04080 Neuroactive ligand-receptor interaction | 28.65 | 0.8865 |
| bta05320 Autoimmune thyroid disease | 25.35 | 0.8935 |
| bta00590 Arachidonic acid metabolism | 25.61 | 0.8948 |
| bta00983 Drug metabolism | 25.00 | 0.9046 |
| bta04714 Thermogenesis | 27.62 | 0.9046 |
| bta00980 Metabolism of xenobiotics by cytochrome P450 | 23.88 | 0.9269 |
| bta00480 Glutathione metabolism | 23.33 | 0.9294 |
| bta04932 Non-alcoholic fatty liver disease (NAFLD) | 25.95 | 0.9344 |
| bta00970 Aminoacyl-tRNA biosynthesis | 22.73 | 0.9427 |
| bta04610 Complement and coagulation cascades | 23.91 | 0.9427 |
| bta05206 MicroRNAs in cancer | 27.05 | 0.9427 |
| bta05010 Alzheimer disease | 25.56 | 0.9477 |
| bta05034 Alcoholism | 26.20 | 0.9477 |
| bta03008 Ribosome biogenesis in eukaryotes | 22.89 | 0.9507 |
| bta05150 Staphylococcus aureus infection | 22.86 | 0.9689 |
| bta03050 Proteasome | 17.39 | 0.9904 |
| bta02010 ABC transporters | 18.33 | 0.9926 |
| bta00190 Oxidative phosphorylation | 12.14 | 1.0 |
| bta03010 Ribosome | 13.13 | 1.0 |
| bta04740 Olfactory transduction | 4.63 | 1.0 |
| bta04974 Protein digestion and absorption | 17.36 | 1.0 |
| bta05012 Parkinson disease | 17.33 | 1.0 |
| bta05016 Huntington disease | 21.53 | 1.0 |
| bta05322 Systemic lupus erythematosus | 14.29 | 1.0 |
| ^1^%: Percent of genes predicted to be modulated. ^2^BH: Benjamini – Hochberg | | |
