# Supplementary material for: The bovine oviductal environment and composition are negatively affected by elevated body energy reserves
Source: PLoS One. 2025 Jun 23;20(6):e0326138. doi: 10.1371/journal.pone.0326138 (PMC12184905; doi:10.1371/journal.pone.0326138)
Supplement: S13 Table — (DOCX) [file pone.0326138.s016.docx]

| **Supplementary table 13.** Biological patwhays predicted as modulated by miRNAs up regulated in ampullary luminal epithelial cells (AMP-Cell) in high body energy reserve (HBER) group. | | |
| --- | --- | --- |
| **Pathway** | **%^1^** | **BH^2^** |
| bta05205 Proteoglycans in cancer | 18.05 | 0.0 |
| bta04810 Regulation of actin cytoskeleton | 16.59 | 0.0066 |
| bta05211 Renal cell carcinoma | 23.94 | 0.0066 |
| bta04014 Ras signaling pathway | 14.46 | 0.0239 |
| bta04360 Axon guidance | 15.73 | 0.0239 |
| bta04012 ErbB signaling pathway | 19.05 | 0.0318 |
| bta04144 Endocytosis | 13.88 | 0.0318 |
| bta04660 T cell receptor signaling pathway | 17.76 | 0.0318 |
| bta04722 Neurotrophin signaling pathway | 17.21 | 0.0318 |
| bta05212 Pancreatic cancer | 19.74 | 0.0318 |
| bta05214 Glioma | 19.48 | 0.0326 |
| bta04550 Signaling pathways regulating pluripotency of stem cells | 15.49 | 0.0398 |
| bta04625 C-type lectin receptor signaling pathway | 16.98 | 0.0398 |
| bta04370 VEGF signaling pathway | 20.69 | 0.0455 |
| bta05223 Non-small cell lung cancer | 19.40 | 0.0478 |
| bta01522 Endocrine resistance | 17.02 | 0.0492 |
| bta04022 cGMP-PKG signaling pathway | 14.20 | 0.0492 |
| bta05200 Pathways in cancer | 11.07 | 0.0492 |
| bta05220 Chronic myeloid leukemia | 18.18 | 0.0492 |
| bta05225 Hepatocellular carcinoma | 13.79 | 0.0637 |
| bta04919 Thyroid hormone signaling pathway | 15.25 | 0.0654 |
| bta00564 Glycerophospholipid metabolism | 15.38 | 0.0812 |
| bta04010 MAPK signaling pathway | 11.99 | 0.0812 |
| bta04020 Calcium signaling pathway | 12.87 | 0.0812 |
| bta04921 Oxytocin signaling pathway | 13.82 | 0.0812 |
| bta04664 Fc epsilon RI signaling pathway | 17.14 | 0.088 |
| bta05210 Colorectal cancer | 15.73 | 0.0929 |
| bta05414 Dilated cardiomyopathy (DCM) | 15.15 | 0.0952 |
| bta04510 Focal adhesion | 12.63 | 0.0961 |
| bta05100 Bacterial invasion of epithelial cells | 16.44 | 0.0995 |
| bta05410 Hypertrophic cardiomyopathy (HCM) | 15.22 | 0.1021 |
| bta04666 Fc gamma R-mediated phagocytosis | 15.05 | 0.107 |
| bta05226 Gastric cancer | 13.07 | 0.1135 |
| bta05412 Arrhythmogenic right ventricular cardiomyopathy (ARVC) | 15.79 | 0.1135 |
| bta04150 mTOR signaling pathway | 12.74 | 0.1342 |
| bta01521 EGFR tyrosine kinase inhibitor resistance | 15.00 | 0.135 |
| bta04071 Sphingolipid signaling pathway | 13.33 | 0.135 |
| bta04151 PI3K-Akt signaling pathway | 10.72 | 0.135 |
| bta04261 Adrenergic signaling in cardiomyocytes | 12.67 | 0.135 |
| bta05135 Yersinia infection | 13.08 | 0.135 |
| bta05163 Human cytomegalovirus infection | 11.43 | 0.135 |
| bta05202 Transcriptional misregulation in cancer | 12.04 | 0.135 |
| bta04611 Platelet activation | 13.22 | 0.1402 |
| bta04140 Autophagy | 12.68 | 0.1406 |
| bta05218 Melanoma | 15.07 | 0.1406 |
| bta04270 Vascular smooth muscle contraction | 12.78 | 0.1427 |
| bta05235 PD-L1 expression and PD-1 checkpoint pathway in cancer | 13.98 | 0.1427 |
| bta04925 Aldosterone synthesis and secretion | 13.54 | 0.1696 |
| bta04915 Estrogen signaling pathway | 12.32 | 0.1779 |
| bta04961 Endocrine and other factor-regulated calcium reabsorption | 16.00 | 0.1814 |
| bta05213 Endometrial cancer | 15.25 | 0.1814 |
| bta05224 Breast cancer | 12.00 | 0.1814 |
| bta05231 Choline metabolism in cancer | 13.13 | 0.184 |
| bta04068 FoxO signaling pathway | 12.21 | 0.1885 |
| bta04072 Phospholipase D signaling pathway | 11.84 | 0.1885 |
| bta04380 Osteoclast differentiation | 11.94 | 0.2072 |
| bta04724 Glutamatergic synapse | 12.39 | 0.2072 |
| bta04924 Renin secretion | 13.89 | 0.2072 |
| bta04390 Hippo signaling pathway | 11.54 | 0.2076 |
| bta04912 GnRH signaling pathway | 12.90 | 0.2076 |
| bta04630 JAK-STAT signaling pathway | 10.89 | 0.2222 |
| bta04015 Rap1 signaling pathway | 10.65 | 0.2388 |
| bta04960 Aldosterone-regulated sodium reabsorption | 16.22 | 0.2388 |
| bta05230 Central carbon metabolism in cancer | 13.64 | 0.2388 |
| bta04926 Relaxin signaling pathway | 11.54 | 0.2484 |
| bta05215 Prostate cancer | 12.24 | 0.2484 |
| bta01524 Platinum drug resistance | 12.82 | 0.256 |
| bta04066 HIF-1 signaling pathway | 11.82 | 0.2587 |
| bta04062 Chemokine signaling pathway | 10.64 | 0.2658 |
| bta04141 Protein processing in endoplasmic reticulum | 10.84 | 0.2658 |
| bta05206 MicroRNAs in cancer | 9.93 | 0.2679 |
| bta04728 Dopaminergic synapse | 11.19 | 0.2711 |
| bta04916 Melanogenesis | 11.76 | 0.2748 |
| bta00512 Mucin type O-glycan biosynthesis | 16.13 | 0.2759 |
| bta04917 Prolactin signaling pathway | 12.05 | 0.2936 |
| bta04976 Bile secretion | 12.05 | 0.2936 |
| bta05165 Human papillomavirus infection | 9.57 | 0.2936 |
| bta05219 Bladder cancer | 14.29 | 0.2939 |
| bta03015 mRNA surveillance pathway | 11.58 | 0.3045 |
| bta04910 Insulin signaling pathway | 10.71 | 0.3075 |
| bta04911 Insulin secretion | 11.76 | 0.3075 |
| bta05017 Spinocerebellar ataxia | 11.46 | 0.3075 |
| bta00514 Other types of O-glycan biosynthesis | 13.33 | 0.308 |
| bta04114 Oocyte meiosis | 10.92 | 0.308 |
| bta04142 Lysosome | 10.61 | 0.308 |
| bta04210 Apoptosis | 10.56 | 0.308 |
| bta04218 Cellular senescence | 10.24 | 0.308 |
| bta04650 Natural killer cell mediated cytotoxicity | 10.69 | 0.308 |
| bta04662 B cell receptor signaling pathway | 11.49 | 0.308 |
| bta04978 Mineral absorption | 12.73 | 0.308 |
| bta05160 Hepatitis C | 10.37 | 0.308 |
| bta05166 Human T-cell leukemia virus 1 infection | 9.83 | 0.308 |
| bta04934 Cushing syndrome | 10.26 | 0.316 |
| bta05418 Fluid shear stress and atherosclerosis | 10.34 | 0.3178 |
| bta00790 Folate biosynthesis | 13.89 | 0.3236 |
| bta04913 Ovarian steroidogenesis | 12.28 | 0.3248 |
| bta04670 Leukocyte transendothelial migration | 10.62 | 0.3334 |
| bta05167 Kaposi sarcoma-associated herpesvirus infection | 9.71 | 0.3334 |
| bta04720 Long-term potentiation | 11.59 | 0.3361 |
| bta04750 Inflammatory mediator regulation of TRP channels | 10.68 | 0.3415 |
| bta04520 Adherens junction | 11.43 | 0.3462 |
| bta04310 Wnt signaling pathway | 9.88 | 0.347 |
| bta04928 Parathyroid hormone synthesis. secretion and action | 10.58 | 0.347 |
| bta00600 Sphingolipid metabolism | 12.24 | 0.3496 |
| bta04371 Apelin signaling pathway | 10.00 | 0.3541 |
| bta05222 Small cell lung cancer | 10.64 | 0.3541 |
| bta04530 Tight junction | 9.60 | 0.3641 |
| bta04935 Growth hormone synthesis. secretion and action | 10.17 | 0.3641 |
| bta04340 Hedgehog signaling pathway | 11.76 | 0.3732 |
| bta04918 Thyroid hormone synthesis | 10.81 | 0.3824 |
| bta04064 NF-kappa B signaling pathway | 10.09 | 0.3825 |
| bta05340 Primary immunodeficiency | 12.20 | 0.3825 |
| bta04024 cAMP signaling pathway | 9.17 | 0.3853 |
| bta00230 Purine metabolism | 9.70 | 0.3943 |
| bta04110 Cell cycle | 9.76 | 0.3958 |
| bta04152 AMPK signaling pathway | 9.76 | 0.3958 |
| bta04927 Cortisol synthesis and secretion | 10.77 | 0.3958 |
| bta04929 GnRH secretion | 10.77 | 0.3958 |
| bta04137 Mitophagy | 10.61 | 0.4076 |
| bta04659 Th17 cell differentiation | 9.73 | 0.4076 |
| bta04721 Synaptic vesicle cycle | 10.26 | 0.4076 |
| bta05152 Tuberculosis | 9.09 | 0.4076 |
| bta04540 Gap junction | 10.00 | 0.4082 |
| bta00561 Glycerolipid metabolism | 10.45 | 0.4108 |
| bta00240 Pyrimidine metabolism | 10.71 | 0.4173 |
| bta04216 Ferroptosis | 11.11 | 0.4225 |
| bta04350 TGF-beta signaling pathway | 9.68 | 0.4392 |
| bta04668 TNF signaling pathway | 9.32 | 0.4443 |
| bta04923 Regulation of lipolysis in adipocytes | 10.34 | 0.4443 |
| bta04061 Viral protein interaction with cytokine and cytokine receptor | 9.47 | 0.4583 |
| bta05161 Hepatitis B | 8.77 | 0.4809 |
| bta04060 Cytokine-cytokine receptor interaction | 8.36 | 0.4825 |
| bta04962 Vasopressin-regulated water reabsorption | 10.20 | 0.4825 |
| bta04213 Longevity regulating pathway | 9.68 | 0.4987 |
| bta05217 Basal cell carcinoma | 9.52 | 0.5134 |
| bta04914 Progesterone-mediated oocyte maturation | 9.09 | 0.5159 |
| bta04971 Gastric acid secretion | 9.21 | 0.5183 |
| bta00510 N-Glycan biosynthesis | 9.62 | 0.522 |
| bta04725 Cholinergic synapse | 8.77 | 0.522 |
| bta04115 p53 signaling pathway | 9.09 | 0.5235 |
| bta00310 Lysine degradation | 9.09 | 0.5449 |
| bta04970 Salivary secretion | 8.60 | 0.5688 |
| bta04672 Intestinal immune network for IgA production | 8.93 | 0.5854 |
| bta04640 Hematopoietic cell lineage | 8.18 | 0.6192 |
| bta04730 Long-term depression | 8.33 | 0.6485 |
| bta05014 Amyotrophic lateral sclerosis (ALS) | 8.33 | 0.6485 |
| bta05170 Human immunodeficiency virus 1 infection | 7.69 | 0.6621 |
| bta04211 Longevity regulating pathway | 7.78 | 0.6759 |
| bta04260 Cardiac muscle contraction | 7.87 | 0.6759 |
| bta04621 NOD-like receptor signaling pathway | 7.61 | 0.6759 |
| bta04922 Glucagon signaling pathway | 7.77 | 0.6759 |
| bta04933 AGE-RAGE signaling pathway in diabetic complications | 7.77 | 0.6759 |
| bta05133 Pertussis | 7.79 | 0.6791 |
| bta05169 Epstein-Barr virus infection | 7.46 | 0.6975 |
| bta04620 Toll-like receptor signaling pathway | 7.27 | 0.7417 |
| bta04931 Insulin resistance | 7.27 | 0.7417 |
| bta03008 Ribosome biogenesis in eukaryotes | 7.23 | 0.7418 |
| bta04120 Ubiquitin mediated proteolysis | 7.14 | 0.7418 |
| bta04658 Th1 and Th2 cell differentiation | 7.14 | 0.7418 |
| bta05031 Amphetamine addiction | 7.25 | 0.7418 |
| bta05168 Herpes simplex virus 1 infection | 7.20 | 0.7418 |
| bta05321 Inflammatory bowel disease (IBD) | 7.14 | 0.7418 |
| bta04713 Circadian entrainment | 7.00 | 0.7579 |
| bta04920 Adipocytokine signaling pathway | 6.94 | 0.7612 |
| bta04932 Non-alcoholic fatty liver disease (NAFLD) | 6.96 | 0.7632 |
| bta04217 Necroptosis | 6.90 | 0.7753 |
| bta04512 ECM-receptor interaction | 6.74 | 0.7796 |
| bta00983 Drug metabolism | 6.58 | 0.7817 |
| bta03013 RNA transport | 6.74 | 0.7817 |
| bta04080 Neuroactive ligand-receptor interaction | 6.89 | 0.7817 |
| bta04657 IL-17 signaling pathway | 6.52 | 0.7817 |
| bta04727 GABAergic synapse | 6.59 | 0.7817 |
| bta05032 Morphine addiction | 6.52 | 0.7817 |
| bta05416 Viral myocarditis | 6.58 | 0.7817 |
| bta05140 Leishmaniasis | 6.41 | 0.7862 |
| bta05164 Influenza A | 6.63 | 0.7862 |
| bta03018 RNA degradation | 6.33 | 0.7895 |
| bta05203 Viral carcinogenesis | 6.64 | 0.7935 |
| bta04145 Phagosome | 6.47 | 0.7991 |
| bta04514 Cell adhesion molecules (CAMs) | 6.33 | 0.8101 |
| bta05145 Toxoplasmosis | 6.19 | 0.8101 |
| bta04070 Phosphatidylinositol signaling system | 6.06 | 0.8143 |
| bta04146 Peroxisome | 5.95 | 0.8143 |
| bta01100 Metabolic pathways | 6.94 | 0.8181 |
| bta03040 Spliceosome | 6.12 | 0.8181 |
| bta04612 Antigen processing and presentation | 5.88 | 0.8181 |
| bta04972 Pancreatic secretion | 5.88 | 0.8181 |
| bta05132 Salmonella infection | 6.25 | 0.8206 |
| bta04723 Retrograde endocannabinoid signaling | 5.92 | 0.8221 |
| bta04974 Protein digestion and absorption | 5.79 | 0.8221 |
| bta05162 Measles | 5.92 | 0.8221 |
| bta05323 Rheumatoid arthritis | 5.77 | 0.8221 |
| bta05142 Chagas disease (American trypanosomiasis) | 5.22 | 0.8788 |
| bta04726 Serotonergic synapse | 5.17 | 0.8796 |
| bta04714 Thermogenesis | 5.44 | 0.9138 |
| bta05010 Alzheimer disease | 5.00 | 0.9221 |
| bta05034 Alcoholism | 5.24 | 0.9221 |
| bta05016 Huntington disease | 5.11 | 0.9437 |
| bta04740 Olfactory transduction | 0.96 | 1.0 |
| ^1^%: Percent of genes predicted to be modulated. ^2^BH: Benjamini – Hochberg | | |
