# Supplementary material for: The bovine oviductal environment and composition are negatively affected by elevated body energy reserves
Source: PLoS One. 2025 Jun 23;20(6):e0326138. doi: 10.1371/journal.pone.0326138 (PMC12184905; doi:10.1371/journal.pone.0326138)
Supplement: S14 Table — (DOCX) [file pone.0326138.s017.docx]

| **Supplementary table 14.** Biological patwhays predicted as modulated by miRNAs up regulated in isthmic luminal epithelial cells (IST-Cell) in high body energy reserve (HBER) group. | | |
| --- | --- | --- |
| **Pathway** | **%^1^** | **BH^2^** |
| bta04010 MAPK signaling pathway | 50.34 | 0.0 |
| bta04014 Ras signaling pathway | 53.31 | 0.0 |
| bta04015 Rap1 signaling pathway | 53.24 | 0.0 |
| bta04360 Axon guidance | 55.62 | 0.0 |
| bta05200 Pathways in cancer | 47.79 | 0.0 |
| bta04144 Endocytosis | 50.61 | 0.0039 |
| bta04810 Regulation of actin cytoskeleton | 52.13 | 0.0039 |
| bta04934 Cushing syndrome | 55.13 | 0.0039 |
| bta04072 Phospholipase D signaling pathway | 53.95 | 0.0105 |
| bta04062 Chemokine signaling pathway | 50.53 | 0.0126 |
| bta04140 Autophagy | 54.23 | 0.0126 |
| bta04150 mTOR signaling pathway | 52.87 | 0.0126 |
| bta04390 Hippo signaling pathway | 52.56 | 0.0126 |
| bta04910 Insulin signaling pathway | 54.29 | 0.0126 |
| bta05205 Proteoglycans in cancer | 49.76 | 0.0126 |
| bta01522 Endocrine resistance | 57.45 | 0.0192 |
| bta05212 Pancreatic cancer | 60.53 | 0.0192 |
| bta05215 Prostate cancer | 57.14 | 0.0192 |
| bta05224 Breast cancer | 51.33 | 0.0231 |
| bta04550 Signaling pathways regulating pluripotency of stem cells | 51.41 | 0.0267 |
| bta04611 Platelet activation | 52.89 | 0.0284 |
| bta04520 Adherens junction | 60.00 | 0.0285 |
| bta04012 ErbB signaling pathway | 57.14 | 0.0287 |
| bta04931 Insulin resistance | 53.64 | 0.0288 |
| bta01100 Metabolic pathways | 38.18 | 0.0289 |
| bta04020 Calcium signaling pathway | 47.03 | 0.0289 |
| bta04310 Wnt signaling pathway | 48.77 | 0.0289 |
| bta04510 Focal adhesion | 47.47 | 0.0289 |
| bta04659 Th17 cell differentiation | 52.21 | 0.0289 |
| bta04660 T cell receptor signaling pathway | 53.27 | 0.0289 |
| bta04668 TNF signaling pathway | 51.69 | 0.0289 |
| bta04722 Neurotrophin signaling pathway | 51.64 | 0.0289 |
| bta04922 Glucagon signaling pathway | 53.40 | 0.0289 |
| bta05100 Bacterial invasion of epithelial cells | 57.53 | 0.0289 |
| bta05135 Yersinia infection | 50.77 | 0.0289 |
| bta05210 Colorectal cancer | 55.06 | 0.0289 |
| bta05211 Renal cell carcinoma | 59.15 | 0.0289 |
| bta05230 Central carbon metabolism in cancer | 59.09 | 0.0289 |
| bta05226 Gastric cancer | 49.02 | 0.0306 |
| bta00514 Other types of O-glycan biosynthesis | 64.44 | 0.0345 |
| bta05214 Glioma | 55.84 | 0.036 |
| bta04928 Parathyroid hormone synthesis. secretion and action | 51.92 | 0.0396 |
| bta04916 Melanogenesis | 51.96 | 0.0416 |
| bta04530 Tight junction | 46.89 | 0.0428 |
| bta04071 Sphingolipid signaling pathway | 50.00 | 0.0447 |
| bta04514 Cell adhesion molecules (CAMs) | 47.47 | 0.0451 |
| bta04919 Thyroid hormone signaling pathway | 50.00 | 0.0451 |
| bta05220 Chronic myeloid leukemia | 54.55 | 0.0451 |
| bta04024 cAMP signaling pathway | 44.54 | 0.0521 |
| bta04142 Lysosome | 48.48 | 0.0521 |
| bta04625 C-type lectin receptor signaling pathway | 50.00 | 0.0598 |
| bta05225 Hepatocellular carcinoma | 45.98 | 0.0598 |
| bta04664 Fc epsilon RI signaling pathway | 54.29 | 0.0599 |
| bta05163 Human cytomegalovirus infection | 43.67 | 0.0599 |
| bta05231 Choline metabolism in cancer | 50.51 | 0.0599 |
| bta01521 EGFR tyrosine kinase inhibitor resistance | 52.50 | 0.0611 |
| bta04921 Oxytocin signaling pathway | 46.71 | 0.0611 |
| bta04270 Vascular smooth muscle contraction | 47.37 | 0.0664 |
| bta04720 Long-term potentiation | 53.62 | 0.0664 |
| bta04935 Growth hormone synthesis. secretion and action | 48.31 | 0.0664 |
| bta04961 Endocrine and other factor-regulated calcium reabsorption | 58.00 | 0.0664 |
| bta04925 Aldosterone synthesis and secretion | 50.00 | 0.0679 |
| bta04728 Dopaminergic synapse | 47.01 | 0.0703 |
| bta04371 Apelin signaling pathway | 46.43 | 0.0754 |
| bta05217 Basal cell carcinoma | 53.97 | 0.0754 |
| bta04926 Relaxin signaling pathway | 46.92 | 0.0756 |
| bta04658 Th1 and Th2 cell differentiation | 48.98 | 0.0803 |
| bta04912 GnRH signaling pathway | 49.46 | 0.0803 |
| bta05017 Spinocerebellar ataxia | 48.96 | 0.0846 |
| bta00564 Glycerophospholipid metabolism | 48.08 | 0.0884 |
| bta04920 Adipocytokine signaling pathway | 51.39 | 0.0902 |
| bta04915 Estrogen signaling pathway | 45.65 | 0.0946 |
| bta01212 Fatty acid metabolism | 53.45 | 0.0951 |
| bta04152 AMPK signaling pathway | 46.34 | 0.0963 |
| bta05221 Acute myeloid leukemia | 51.47 | 0.0971 |
| bta05235 PD-L1 expression and PD-1 checkpoint pathway in cancer | 48.39 | 0.0987 |
| bta05132 Salmonella infection | 42.41 | 0.1036 |
| bta00512 Mucin type O-glycan biosynthesis | 61.29 | 0.1053 |
| bta04120 Ubiquitin mediated proteolysis | 45.00 | 0.1053 |
| bta00533 Glycosaminoglycan biosynthesis | 78.57 | 0.1082 |
| bta04392 Hippo signaling pathway | 62.07 | 0.1082 |
| bta04924 Renin secretion | 50.00 | 0.1107 |
| bta00562 Inositol phosphate metabolism | 49.32 | 0.1139 |
| bta04215 Apoptosis | 58.82 | 0.1139 |
| bta04261 Adrenergic signaling in cardiomyocytes | 44.00 | 0.1139 |
| bta04666 Fc gamma R-mediated phagocytosis | 47.31 | 0.1139 |
| bta04730 Long-term depression | 51.67 | 0.1139 |
| bta04750 Inflammatory mediator regulation of TRP channels | 46.60 | 0.1139 |
| bta04911 Insulin secretion | 48.24 | 0.1139 |
| bta04914 Progesterone-mediated oocyte maturation | 47.73 | 0.1139 |
| bta04917 Prolactin signaling pathway | 48.19 | 0.1139 |
| bta00310 Lysine degradation | 50.00 | 0.1143 |
| bta04022 cGMP-PKG signaling pathway | 43.20 | 0.1143 |
| bta04064 NF-kappa B signaling pathway | 45.87 | 0.1143 |
| bta04137 Mitophagy | 50.00 | 0.1143 |
| bta04340 Hedgehog signaling pathway | 52.94 | 0.1143 |
| bta05412 Arrhythmogenic right ventricular cardiomyopathy (ARVC) | 48.68 | 0.1143 |
| bta04070 Phosphatidylinositol signaling system | 46.46 | 0.1153 |
| bta05213 Endometrial cancer | 50.85 | 0.1168 |
| bta05219 Bladder cancer | 54.76 | 0.1168 |
| bta00510 N-Glycan biosynthesis | 51.92 | 0.1219 |
| bta04670 Leukocyte transendothelial migration | 45.13 | 0.122 |
| bta05165 Human papillomavirus infection | 39.71 | 0.122 |
| bta05223 Non-small cell lung cancer | 49.25 | 0.122 |
| bta04927 Cortisol synthesis and secretion | 49.23 | 0.1274 |
| bta05014 Amyotrophic lateral sclerosis (ALS) | 50.00 | 0.1274 |
| bta04350 TGF-beta signaling pathway | 46.24 | 0.1277 |
| bta05202 Transcriptional misregulation in cancer | 41.88 | 0.1294 |
| bta04330 Notch signaling pathway | 50.94 | 0.131 |
| bta04370 VEGF signaling pathway | 50.00 | 0.131 |
| bta04066 HIF-1 signaling pathway | 44.55 | 0.1403 |
| bta04068 FoxO signaling pathway | 43.51 | 0.1403 |
| bta05161 Hepatitis B | 42.11 | 0.1403 |
| bta00230 Purine metabolism | 43.28 | 0.1427 |
| bta05166 Human T-cell leukemia virus 1 infection | 40.60 | 0.1431 |
| bta01040 Biosynthesis of unsaturated fatty acids | 56.67 | 0.1442 |
| bta04151 PI3K-Akt signaling pathway | 38.87 | 0.1442 |
| bta04211 Longevity regulating pathway | 45.56 | 0.1442 |
| bta04726 Serotonergic synapse | 43.97 | 0.1442 |
| bta05160 Hepatitis C | 42.07 | 0.1442 |
| bta00770 Pantothenate and CoA biosynthesis | 63.16 | 0.156 |
| bta01524 Platinum drug resistance | 46.15 | 0.156 |
| bta04978 Mineral absorption | 49.09 | 0.156 |
| bta05170 Human immunodeficiency virus 1 infection | 40.17 | 0.1588 |
| bta00513 Various types of N-glycan biosynthesis | 51.16 | 0.1615 |
| bta04971 Gastric acid secretion | 46.05 | 0.1625 |
| bta04713 Circadian entrainment | 44.00 | 0.1699 |
| bta04930 Type II diabetes mellitus | 50.00 | 0.1702 |
| bta04540 Gap junction | 44.44 | 0.1749 |
| bta05167 Kaposi sarcoma-associated herpesvirus infection | 40.29 | 0.1749 |
| bta04725 Cholinergic synapse | 42.98 | 0.1764 |
| bta00100 Steroid biosynthesis | 60.00 | 0.1808 |
| bta00071 Fatty acid degradation | 50.00 | 0.1891 |
| bta04721 Synaptic vesicle cycle | 44.87 | 0.1903 |
| bta05218 Melanoma | 45.21 | 0.1935 |
| bta04923 Regulation of lipolysis in adipocytes | 46.55 | 0.2048 |
| bta04114 Oocyte meiosis | 42.02 | 0.206 |
| bta04061 Viral protein interaction with cytokine and cytokine receptor | 43.16 | 0.2071 |
| bta00240 Pyrimidine metabolism | 46.43 | 0.2148 |
| bta04210 Apoptosis | 40.85 | 0.2227 |
| bta05414 Dilated cardiomyopathy (DCM) | 42.42 | 0.2285 |
| bta00592 alpha-Linolenic acid metabolism | 51.72 | 0.235 |
| bta04724 Glutamatergic synapse | 41.59 | 0.235 |
| bta00565 Ether lipid metabolism | 46.15 | 0.2388 |
| bta04146 Peroxisome | 42.86 | 0.2439 |
| bta05032 Morphine addiction | 42.39 | 0.2439 |
| bta04662 B cell receptor signaling pathway | 42.53 | 0.2493 |
| bta05216 Thyroid cancer | 47.50 | 0.2599 |
| bta00020 Citrate cycle (TCA cycle) | 50.00 | 0.2638 |
| bta00010 Glycolysis Gluconeogenesis | 43.75 | 0.2707 |
| bta01200 Carbon metabolism | 40.71 | 0.2759 |
| bta05340 Primary immunodeficiency | 46.34 | 0.2882 |
| bta04657 IL-17 signaling pathway | 41.30 | 0.2899 |
| bta04933 AGE-RAGE signaling pathway in diabetic complications | 40.78 | 0.2899 |
| bta05410 Hypertrophic cardiomyopathy (HCM) | 41.30 | 0.2899 |
| bta04710 Circadian rhythm | 48.39 | 0.2923 |
| bta05418 Fluid shear stress and atherosclerosis | 39.31 | 0.2962 |
| bta04380 Osteoclast differentiation | 39.55 | 0.2969 |
| bta00760 Nicotinate and nicotinamide metabolism | 46.15 | 0.297 |
| bta05142 Chagas disease (American trypanosomiasis) | 40.00 | 0.302 |
| bta00534 Glycosaminoglycan biosynthesis | 50.00 | 0.3086 |
| bta05169 Epstein-Barr virus infection | 37.72 | 0.3086 |
| bta04141 Protein processing in endoplasmic reticulum | 38.55 | 0.31 |
| bta05031 Amphetamine addiction | 42.03 | 0.31 |
| bta05222 Small cell lung cancer | 40.43 | 0.318 |
| bta04964 Proximal tubule bicarbonate reclamation | 50.00 | 0.3272 |
| bta04650 Natural killer cell mediated cytotoxicity | 38.93 | 0.3312 |
| bta05321 Inflammatory bowel disease (IBD) | 41.43 | 0.3312 |
| bta00620 Pyruvate metabolism | 44.74 | 0.3408 |
| bta00640 Propanoate metabolism | 45.45 | 0.3465 |
| bta04130 SNARE interactions in vesicular transport | 45.45 | 0.3465 |
| bta04962 Vasopressin-regulated water reabsorption | 42.86 | 0.3465 |
| bta05162 Measles | 38.16 | 0.3472 |
| bta05145 Toxoplasmosis | 38.94 | 0.3555 |
| bta00515 Mannose type O-glycan biosynthesis | 47.83 | 0.3556 |
| bta04115 p53 signaling pathway | 40.26 | 0.3556 |
| bta04621 NOD-like receptor signaling pathway | 37.50 | 0.3556 |
| bta04972 Pancreatic secretion | 39.22 | 0.3556 |
| bta00052 Galactose metabolism | 45.16 | 0.3592 |
| bta00061 Fatty acid biosynthesis | 50.00 | 0.3592 |
| bta04976 Bile secretion | 39.76 | 0.3608 |
| bta00410 beta-Alanine metabolism | 44.12 | 0.3697 |
| bta04216 Ferroptosis | 42.22 | 0.3759 |
| bta00062 Fatty acid elongation | 44.83 | 0.3787 |
| bta04960 Aldosterone-regulated sodium reabsorption | 43.24 | 0.3787 |
| bta01230 Biosynthesis of amino acids | 39.73 | 0.3836 |
| bta05020 Prion diseases | 43.75 | 0.3896 |
| bta00260 Glycine. serine and threonine metabolism | 41.86 | 0.3937 |
| bta00600 Sphingolipid metabolism | 40.82 | 0.4066 |
| bta04727 GABAergic synapse | 38.46 | 0.4066 |
| bta04918 Thyroid hormone synthesis | 39.19 | 0.4066 |
| bta05133 Pertussis | 38.96 | 0.4066 |
| bta00531 Glycosaminoglycan degradation | 45.45 | 0.4123 |
| bta04136 Autophagy | 42.42 | 0.4183 |
| bta04973 Carbohydrate digestion and absorption | 40.91 | 0.4183 |
| bta05134 Legionellosis | 39.66 | 0.4189 |
| bta00340 Histidine metabolism | 43.48 | 0.4539 |
| bta04213 Longevity regulating pathway | 38.71 | 0.4539 |
| bta04218 Cellular senescence | 36.14 | 0.4539 |
| bta04929 GnRH secretion | 38.46 | 0.4539 |
| bta05030 Cocaine addiction | 39.58 | 0.4539 |
| bta05152 Tuberculosis | 35.86 | 0.4539 |
| bta00280 Valine. leucine and isoleucine degradation | 39.22 | 0.4549 |
| bta05164 Influenza A | 35.91 | 0.4579 |
| bta04913 Ovarian steroidogenesis | 38.60 | 0.4626 |
| bta00601 Glycosphingolipid biosynthesis | 41.38 | 0.4634 |
| bta00730 Thiamine metabolism | 44.44 | 0.4634 |
| bta00500 Starch and sucrose metabolism | 40.63 | 0.4715 |
| bta03320 PPAR signaling pathway | 37.04 | 0.4878 |
| bta00561 Glycerolipid metabolism | 37.31 | 0.4969 |
| bta04970 Salivary secretion | 36.56 | 0.4969 |
| bta00380 Tryptophan metabolism | 38.30 | 0.4985 |
| bta05416 Viral myocarditis | 36.84 | 0.5024 |
| bta00591 Linoleic acid metabolism | 38.89 | 0.5094 |
| bta03030 DNA replication | 38.89 | 0.5094 |
| bta03440 Homologous recombination | 38.10 | 0.5188 |
| bta03420 Nucleotide excision repair | 37.78 | 0.5214 |
| bta04966 Collecting duct acid secretion | 39.29 | 0.5289 |
| bta00051 Fructose and mannose metabolism | 38.24 | 0.5392 |
| bta04080 Neuroactive ligand-receptor interaction | 34.16 | 0.5425 |
| bta00270 Cysteine and methionine metabolism | 36.73 | 0.5548 |
| bta03460 Fanconi anemia pathway | 36.54 | 0.5553 |
| bta03430 Mismatch repair | 39.13 | 0.5556 |
| bta04672 Intestinal immune network for IgA production | 35.71 | 0.5892 |
| bta00670 One carbon pool by folate | 38.89 | 0.5906 |
| bta01210 2-Oxocarboxylic acid metabolism | 38.89 | 0.5906 |
| bta04060 Cytokine-cytokine receptor interaction | 33.75 | 0.5906 |
| bta04620 Toll-like receptor signaling pathway | 34.55 | 0.5906 |
| bta04640 Hematopoietic cell lineage | 34.55 | 0.5906 |
| bta04744 Phototransduction | 37.04 | 0.5961 |
| bta04975 Fat digestion and absorption | 35.42 | 0.6045 |
| bta05146 Amoebiasis | 34.19 | 0.6074 |
| bta00604 Glycosphingolipid biosynthesis | 37.50 | 0.6351 |
| bta01523 Antifolate resistance | 34.88 | 0.6351 |
| bta03015 mRNA surveillance pathway | 33.68 | 0.6537 |
| bta03008 Ribosome biogenesis in eukaryotes | 33.73 | 0.6542 |
| bta00520 Amino sugar and nucleotide sugar metabolism | 34.00 | 0.6626 |
| bta04110 Cell cycle | 33.33 | 0.6626 |
| bta00563 Glycosylphosphatidylinositol (GPI)-anchor biosynthesis | 34.62 | 0.6654 |
| bta03020 RNA polymerase | 34.48 | 0.6654 |
| bta04950 Maturity onset diabetes of the young | 34.62 | 0.6654 |
| bta04977 Vitamin digestion and absorption | 34.62 | 0.6654 |
| bta00532 Glycosaminoglycan biosynthesis | 35.00 | 0.6691 |
| bta00910 Nitrogen metabolism | 35.29 | 0.6705 |
| bta00480 Glutathione metabolism | 33.33 | 0.6708 |
| bta00330 Arginine and proline metabolism | 33.33 | 0.6771 |
| bta00590 Arachidonic acid metabolism | 32.93 | 0.6772 |
| bta00983 Drug metabolism | 32.89 | 0.6772 |
| bta04612 Antigen processing and presentation | 32.94 | 0.6772 |
| bta04742 Taste transduction | 32.91 | 0.6772 |
| bta00900 Terpenoid backbone biosynthesis | 33.33 | 0.7014 |
| bta00250 Alanine. aspartate and glutamate metabolism | 32.43 | 0.7131 |
| bta04723 Retrograde endocannabinoid signaling | 32.24 | 0.716 |
| bta05140 Leishmaniasis | 32.05 | 0.7212 |
| bta00053 Ascorbate and aldarate metabolism | 32.00 | 0.7286 |
| bta03022 Basal transcription factors | 31.82 | 0.7286 |
| bta05323 Rheumatoid arthritis | 31.73 | 0.7388 |
| bta00140 Steroid hormone biosynthesis | 31.34 | 0.7517 |
| bta00350 Tyrosine metabolism | 30.77 | 0.768 |
| bta00360 Phenylalanine metabolism | 30.43 | 0.7709 |
| bta03013 RNA transport | 31.46 | 0.7709 |
| bta03410 Base excision repair | 30.30 | 0.7763 |
| bta00630 Glyoxylate and dicarboxylate metabolism | 30.00 | 0.7834 |
| bta00603 Glycosphingolipid biosynthesis | 29.41 | 0.7894 |
| bta00982 Drug metabolism | 30.16 | 0.7894 |
| bta04217 Necroptosis | 31.03 | 0.7894 |
| bta04979 Cholesterol metabolism | 30.00 | 0.7894 |
| bta04512 ECM-receptor interaction | 30.34 | 0.7946 |
| bta00980 Metabolism of xenobiotics by cytochrome P450 | 29.85 | 0.7993 |
| bta03040 Spliceosome | 30.61 | 0.7993 |
| bta04623 Cytosolic DNA-sensing pathway | 29.85 | 0.7993 |
| bta00030 Pentose phosphate pathway | 28.57 | 0.8039 |
| bta00650 Butanoate metabolism | 28.57 | 0.8039 |
| bta04630 JAK-STAT signaling pathway | 30.69 | 0.8079 |
| bta00511 Other glycan degradation | 27.27 | 0.8296 |
| bta00790 Folate biosynthesis | 27.78 | 0.8339 |
| bta04260 Cardiac muscle contraction | 29.21 | 0.8339 |
| bta00220 Arginine biosynthesis | 26.32 | 0.8382 |
| bta04614 Renin-angiotensin system | 26.92 | 0.8382 |
| bta04932 Non-alcoholic fatty liver disease (NAFLD) | 29.75 | 0.8392 |
| bta05033 Nicotine addiction | 27.50 | 0.8392 |
| bta00040 Pentose and glucuronate interconversions | 25.81 | 0.8704 |
| bta05310 Asthma | 26.32 | 0.8704 |
| bta03018 RNA degradation | 27.85 | 0.8712 |
| bta04714 Thermogenesis | 29.71 | 0.8719 |
| bta05206 MicroRNAs in cancer | 29.79 | 0.8839 |
| bta05204 Chemical carcinogenesis | 27.27 | 0.8852 |
| bta00830 Retinol metabolism | 26.56 | 0.8925 |
| bta05143 African trypanosomiasis | 25.00 | 0.9067 |
| bta04145 Phagosome | 28.24 | 0.9135 |
| bta04940 Type I diabetes mellitus | 25.42 | 0.9155 |
| bta03060 Protein export | 21.74 | 0.9209 |
| bta05016 Huntington disease | 28.83 | 0.9209 |
| bta05150 Staphylococcus aureus infection | 26.67 | 0.9209 |
| bta04610 Complement and coagulation cascades | 26.09 | 0.9257 |
| bta00860 Porphyrin and chlorophyll metabolism | 22.50 | 0.9421 |
| bta05144 Malaria | 23.73 | 0.9432 |
| bta05332 Graft-versus-host disease | 22.92 | 0.9432 |
| bta04622 RIG-I-like receptor signaling pathway | 25.49 | 0.944 |
| bta05010 Alzheimer disease | 26.67 | 0.9582 |
| bta05168 Herpes simplex virus 1 infection | 28.29 | 0.9681 |
| bta05330 Allograft rejection | 21.43 | 0.9746 |
| bta05203 Viral carcinogenesis | 26.56 | 0.979 |
| bta00190 Oxidative phosphorylation | 12.86 | 1.0 |
| bta00970 Aminoacyl-tRNA biosynthesis | 16.67 | 1.0 |
| bta02010 ABC transporters | 16.67 | 1.0 |
| bta03010 Ribosome | 11.88 | 1.0 |
| bta03050 Proteasome | 15.22 | 1.0 |
| bta04740 Olfactory transduction | 3.84 | 1.0 |
| bta04974 Protein digestion and absorption | 22.31 | 1.0 |
| bta05012 Parkinson disease | 20.67 | 1.0 |
| bta05034 Alcoholism | 24.89 | 1.0 |
| bta05320 Autoimmune thyroid disease | 16.90 | 1.0 |
| bta05322 Systemic lupus erythematosus | 11.54 | 1.0 |
| ^1^%: Percent of genes predicted to be modulated. ^2^BH: Benjamini – Hochberg | | |
